# Organocatalytic Asymmetric Conjugate Addition of Fluorooxindoles to Quinone Methides

**DOI:** 10.1021/acs.joc.4c00062

**Published:** 2024-04-15

**Authors:** Maria Bouda, Jeffery A. Bertke, Christian Wolf

**Affiliations:** Chemistry Department, Georgetown University, Washington, D.C. 20057, United States

## Abstract

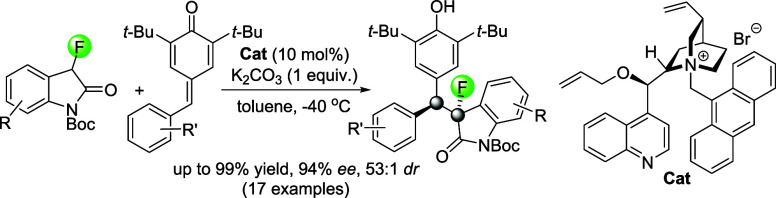

Fluorooxindoles undergo asymmetric Michael addition to *para*-quinone methides under phase-transfer conditions with 10 mol% of a readily available cinchona alkaloid ammonium catalyst. This reaction affords sterically encumbered, multifunctional fluorinated organic compounds displaying two adjacent chirality centers with high yields, *ee*’s and *dr*’s.

The widespread applications and medicinal importance of fluorinated compounds continue to inspire the search for new multifunctional structures that contain a C–F bond and methods to make them.^1−3^ Several advantageous physiochemical properties of organofluorines, including improved lipophilicity, bioavailability, oxidative and thermal stability, and resistance to metabolic degradation in comparison with their nonfluorinated parent molecules, have received considerable attention in modern drug discovery projects. The resultant steady demand for new drug candidates and the general value of chiral compounds in the health sciences have nurtured the development of many asymmetric catalytic procedures that utilize readily available fluorinated building blocks or fluorinating agents.^4−9^ The strong electron-withdrawing and stereoelectronic effects of fluorine, however, often cause unexpected problems with the generation and successive stereocontrolled use of fluorinated reaction intermediates and thus change the outcome of well-established protocols. As a result, asymmetric catalysis with fluorinated species remains challenging, trailing behind the synthetic opportunities available for nonfluorinated compounds. The wide-ranging implications of the multifunctional fluorinated compounds mentioned above call for alternative approaches that resolve these drawbacks.

During recent years, our laboratory has developed various (organo)catalytic methods that produce multifunctional compounds with high enantio- and diastereoselectivities using fluorinated substrates including fluorooxindoles.^10−18^ The medicinal value and prospects of Maxipost and other chiral 3-fluorooxindoles,^19−21^ have received increasing attention from the synthetic community and intriguing procedures that achieve asymmetric C–C bond construction by incorporating the sought-after fluorooxindole motif into versatile scaffolds have been reported.^22−36^

We now report the first catalytic asymmetric 1,6-Michael addition of fluorooxindoles to *para-*quinone methides. This reaction proceeds with excellent yields and installs two contiguous chirality centers, of which one exhibits a C–F moiety, with good to high enantio- and diastereoselectivities in the presence of a cinchona alkaloid derived phase-transfer catalyst (Scheme 1).

**Scheme 1:**
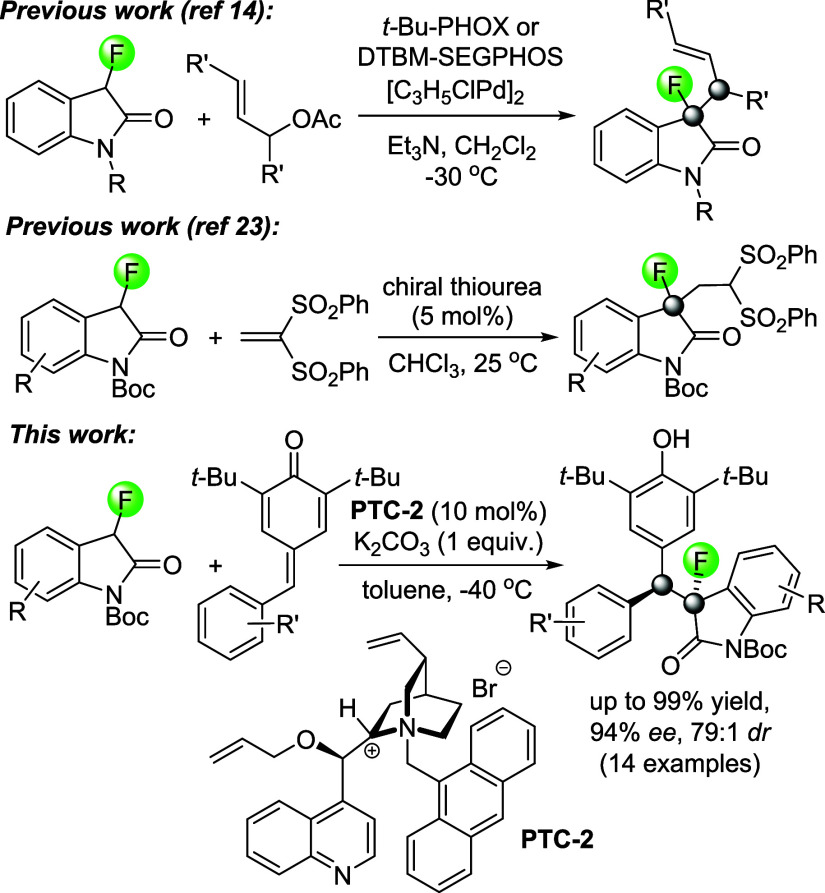
Catalytic Asymmetric Allylic Alkylation and 1,6-Michael Addition with Fluorooxindole Pronucleophiles Generating Sterically Encumbered Scaffolds with Two Adjacent Chirality Centers

## Results and Discussion

To date, several groups have investigated stereoselective carbon–carbon bond formation with indoles, isatins, or oxindole derivatives, and quinone methides as Michael acceptors, giving access to important chiral diarylmethines.^37−43^ Asymmetric conjugate additions of 3-aryloxindoles to quinone methides have been reported by Enders and Fan using squaramide catalysts.^44,45^ Based on our experience with 3-fluorooxindoles we expected that the replacement of the aryl group located at the pronucleophilic carbon center by fluoride would largely alter its reactivity.^46^ Indeed, we found that the squaramide protocol does not give any product when 3-fluorooxindoles are employed, which highlights the strikingly different reactivity of organofluorines compared to their nonfluorinated analogs and the necessity to develop new catalytic methods that address this (Table 1, entry 1). We therefore initiated the search for suitable conditions with the comprehensive screening of organocatalysts, oxindole protecting groups, solvents, and base additives and by varying the reaction temperature (see Table 1 and SI). We were pleased to find that the use of the *N*-Boc oxindole **1** in addition to *para*-quinone methide **2** gives superior results compared to *N*-methyl or *N*-phenyl derived substrates as this was envisioned to greatly facilitate deprotection of the reaction product **3** if desired (SI). Full conversion to the desired product, albeit with disappointing asymmetric induction, was observed with the squaramide catalysts **SQ-1** to **SQ-4** in the presence of cesium carbonate (entries 2–5). This did not improve significantly when we examined urea and thiourea catalysts **U-1** and **TU-1** (entries 6 and 7). Early during our method optimization efforts, we noticed that the use of soluble bases results in low *ee*’s due to considerable background reaction and we therefore decided to employ the well-known cinchonidine-derived phase-transfer catalysts **PTC-1** to **PTC-3** and the binaphthyl ammonium salt **PTC-4** under heterogeneous conditions. **PTC-1** proved to afford **3** in high diastereomeric ratios and *ee*’s up to 62% which was very promising (entries 8–12). We then found that **PTC-2** is capable of generating **3** in even higher enantiomeric excess, but to some extent at the cost of diastereoselectivity when ethereal solvents are used (entries 13 and 14). We finally tested the four phase-transfer catalysts in toluene using potassium carbonate as base and discovered that **3** is formed in 94% *ee* and >20:1 *dr* at −40 °C after 48 h in the presence of 10 mol % of the cinchona alkaloid ammonium salt **PTC-2** (entries 15–18).

**Table 1:**
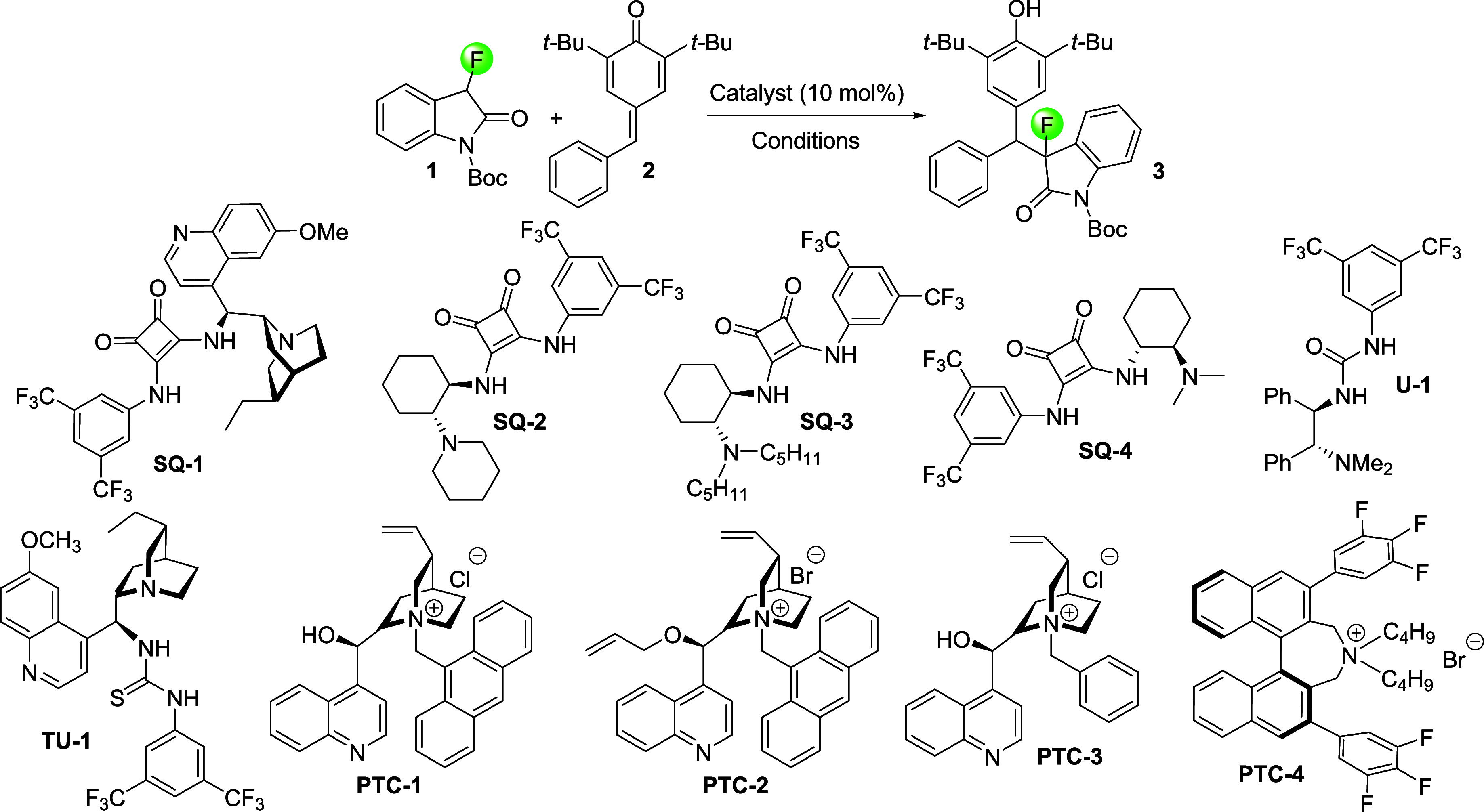
Optimization of the Organocatalytic Asymmetric Conjugate Addition of *N*-Boc-3-fluoro-2-oxindole, **1**, to Quinone Methide **2**^a^

entry	catalyst	base	solvent	temp. (°C)	time (h)	*dr*^b^	% *ee*^c^	conversion^d^
1	**SQ-1**		DCM	–40	24	n.d.	n.d.	0
2	**SQ-1**	Cs_2_CO_3_	Dioxane	0	72	1.5:1	0	99
3	**SQ-2**	Cs_2_CO_3_	DBE	–40	24	2:1	9	99
4	**SQ-3**	Cs_2_CO_3_	DBE	–40	24	1:1	31	99
5	**SQ-4**	Cs_2_CO_3_	DBE	–40	24	1.5:1	5	99
6	**U-1**	Cs_2_CO_3_	DCM	–40	24	2:1	60	99
7	**TU-1**	Cs_2_CO_3_	DBE	–40	24	1:1	27	99
8	**PTC-1**	Cs_2_CO_3_	DME	–40	24	>20:1	13	30
9	**PTC-1**	Cs_2_CO_3_	DBE	–78	24	>20:1	70	95
10	**PTC-1**	K_2_CO_3_	ACN	0	18	>20:1	0	99
11	**PTC-1**	K_2_CO_3_	EtOAc	0	18	>20:1	47	99
12	**PTC-1**	K_2_CO_3_	Dioxane	0	24	9:1	62	99
13	**PTC-2**	Cs_2_CO_3_	Dioxane	–40	24	>20:1	71	99
14	**PTC-2**	Cs_2_CO_3_	DBE	–40	24	1.5:1	95	99
15	**PTC-3**	K_2_CO_3_	Toluene	–40	24	3:1	81	99
16	**PTC-4**	K_2_CO_3_	Toluene	–40	24	>20:1	43	99
17	**PTC-1**	K_2_CO_3_	Toluene	–40	24	>20:1	96	80
18	**PTC-2**	K_2_CO_3_	Toluene	–40	48	>20:1	94	99

a Reaction conditions: *N*-Boc-3-fluoro-2-oxindole (15.0 mg, 0.06 mmol), *para*-quinone methide (17.5 mg, 0.06 mmol), and 10 mol % of the catalyst were dissolved in the indicated solvent (0.2 mL), followed by the addition of the base. Entries 2–6, 8:1.5 equiv of base was used. Entries 7, 9–19:1 equiv of base was used.

b Determined by ^19^F NMR spectroscopy.

c Determined by chiral HPLC.

d Determined by ^1^H NMR spectroscopy. Boc = *t*-butoxy carbonyl. DCM = dichloromethane. DBE = dibutylether. ACN = acetonitrile. DME = dimethoxyethane. n.d. = not determined.

**Scheme 2:**
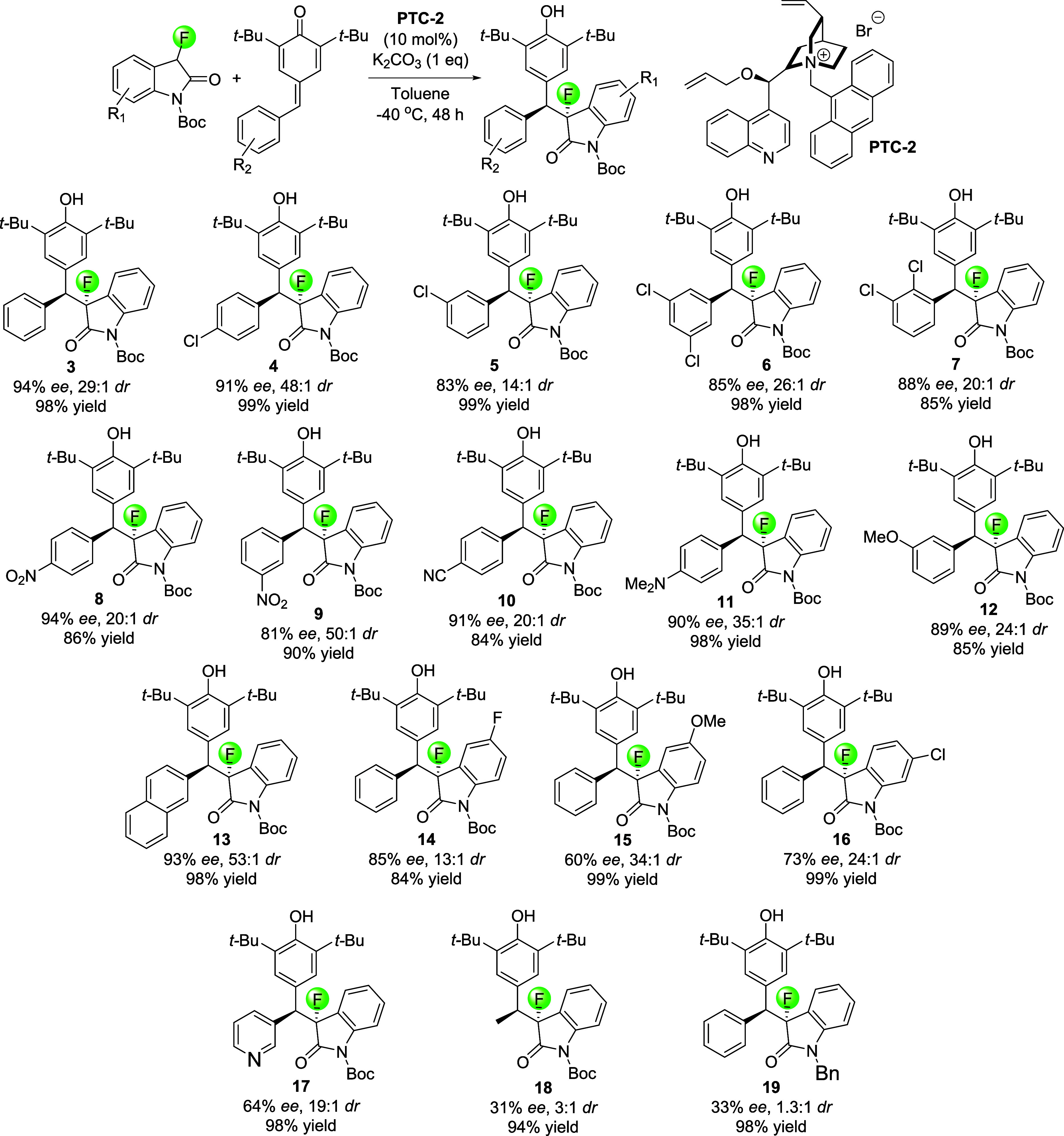
Scope of the Asymmetric Conjugate Addition with the Chiral Phase-Transfer Catalyst PTC-2 General conditions: the *N*-Boc-3-fluoro-2-oxindole (0.06 mmol), *para*-quinone methide (0.06 mmol), potassium carbonate (0.06 mmol), and **PTC-2** (10 mol %) were added to an oven-dried vial under nitrogen. Anhydrous toluene was added and the resulting mixture was stirred at −40 °C for 48 h. The absolute configuration of **11** was determined as described below. The stereochemistry of all other products was assigned by analogy. See SI for details.

With an optimized reaction protocol that gives nearly quantitative yields and allows excellent control of both stereocenters in hand, we continued with investigating the scope of the organocatalytic asymmetric Michael addition (Scheme 2). Compound **3** was isolated in 98% yield, 94% *ee,* and 27:1 *dr*. We then applied our protocol to a broad selection of chlorinated quinone methides and obtained **4**–**7** in high yields, *ee*’s and *dr*’s. The presence of ortho-substituents apparently slows the reaction but is well tolerated otherwise. Very good yields and stereoselectivities were also achieved with quinone methides carrying either electron-withdrawing nitro and cyano groups or electron-donating amino and methoxy groups. The corresponding products **8**–**12** were produced with 84–98% yields, *ee*’s ranging from 81 to 94%, and at least 20:1 *dr*. Exchanging the phenyl moiety with a naphthyl ring gave excellent results, and **13** was produced in 98% yield, 93% *ee,* and 53:1 diastereomeric ratio. Changes in the oxindole structure proved more challenging and were less tolerated. For example, **14** was formed from *N*-Boc-5-fluorooxindole in yields and *ee*’s that are comparable to the results when we varied the quinone methide scaffold but the *dr* was reduced to 13:1. By contrast, we isolated **15** and **16** carrying a 5-methoxy and 6-chloro substituent, respectively, in excellent yields and *dr*’s but with diminished enantiomeric excess. Quinone methides with a heteroaryl or aliphatic substituent can also be used, but we determined decreasing stereoselectivities. In fact, **17** and **18** were obtained in high yields but with low *ee*’s and 19:1 or 3:1 *dr*’s. As mentioned above, the use of Boc-protected fluorooxindoles in this reaction proved to be essential. This becomes apparent from a comparison of the results obtained with *N*-Boc fluorooxindole versus the *N*-benzyl analog. While the asymmetric conjugate addition with the former gives **3** in excellent yield and with high enantio- and diastereoselectivity, the corresponding *N*-benzyl product **19** was produced with substantially lower *ee* and we observed the formation of almost equal amounts of the diastereomers.

**Scheme 3:**
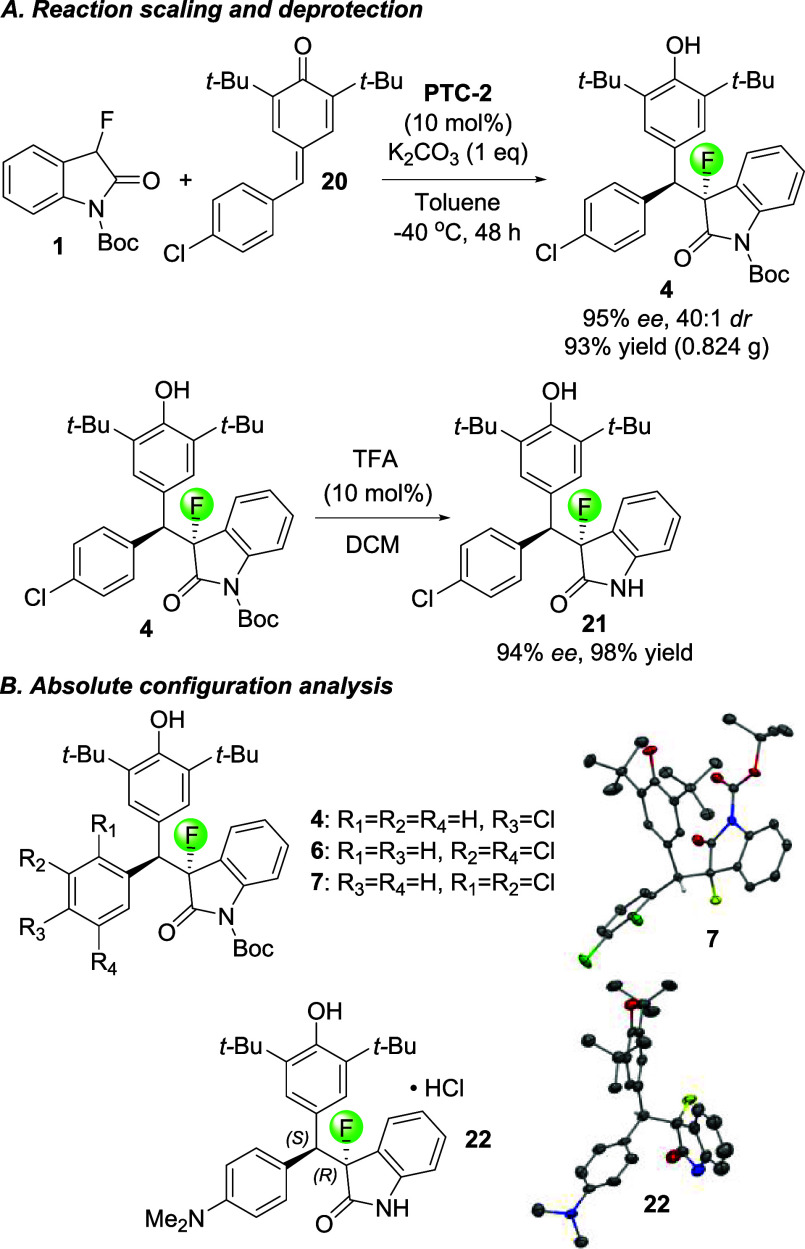
Reaction Scaling, Deprotection, and Determination of the Absolute Configuration See the SI for details.

We then decided to determine whether the reaction could be scaled up without compromising the yield and stereocontrol (Scheme 3). For this purpose, we chose to use oxindole **1** and chloro-substituted quinone methide **20**, of which we applied 500 mg in our optimized protocol. Fortunately, this gave product **4** in 93% yield, 95% *ee,* and 40:1 *dr* which compares well with the results shown in Scheme 2. As expected, the removal of the Boc protecting group can be achieved with very high yields using trifluoroacetic acid, and we isolated **21** in 98% yield. Finally, we set out to determine the absolute configuration of the asymmetric reaction products. This turned out to be a rather daunting task. We realized that compounds **4** (91% *ee*, *dr* = 48:1), **6** (85% *ee*, *dr* = 26:1), and **7** (88% *ee*, *dr* = 20:1) did not form single crystals despite numerous attempts, including slow evaporation of hexanes/isopropanol mixtures, pentane and dichloromethane solvent layering, vapor diffusion, and saturation in ether at 0 °C. By contrast, we easily obtained a single crystal with racemic materials of **7** (3:1 *dr*) suitable for crystallographic analysis which further confirmed that these types of compounds are rather unlikely to crystallize in enantiopure form.

To overcome this problem, we subjected **11** (90% *ee*, *dr* = 35:1) to 1 M HCl in acetone followed by the slow evaporation of the deprotected product **22** from 2-propanol in the hope that the corresponding HCl salt would yield a single crystal. Fortunately, this proved successful, and we were able to assign the absolute configuration at the fluorinated stereocenter and at the tertiary carbon as *R* and *S*, respectively. A polarimetric comparison of the bulk asymmetric reaction material and the single crystal showed that both display dextrorotatory rotation, confirming that the crystallographically determined structure is that of the major enantiomer.

In conclusion, we have developed a method that allows asymmetric Michael additions with *N*-Boc-protected fluorooxindoles and *para*-quinone methides. Comprehensive screening of reaction conditions revealed that this can be achieved with high yields, *ee*’s and *dr*’s by using a commercially available cinchona alkaloid ammonium phase-transfer catalyst and potassium carbonate as base in toluene at −40 °C. The asymmetric carbon–carbon bond construction combines two important, frequently encountered scaffolds that have received considerable interest in recent years and affords sterically crowded, multifunctional fluorinated organic compounds displaying two adjacent chirality centers. It is expected that this study will attract increasing attention to asymmetric catalysis with fluorinated nucleophiles and quinone methides.

## Experimental Section

### Organocatalytic Michael Addition Procedure

*N*-Boc-3-fluoro-2-oxindole (382.0 mg, 1.5 mmol), 2,6-di-*tert*-butyl-4-(4-chlorobenzylidene)cyclohexa-2,5-dien-1-one (500.0 mg, 1.5 mmol), potassium carbonate (210.0 mg, 1.5 mmol), and **PTC-2** (10 mol %) were added to an oven-dried vial under nitrogen. Anhydrous toluene (5.0 mL) was added, and the resulting mixture was stirred at −40 °C for 48 h. Upon completion, the reaction mixture was quenched with an aqueous solution of saturated ammonium chloride and extracted with dichloromethane. The combined organic layers were dried over sodium sulfate, concentrated, and purified via column chromatography using hexanes:ethyl acetate (95,5) as the mobile phase to afford compound **4** as a yellow crystalline solid in 93% yield (824 mg, 1.4 mmol). The *dr* was determined as 40:1 using ^1^H NMR spectroscopy. The *ee* was determined by chiral HPLC (Chiralpak IA, 98:2 hexanes/IPA, flow rate 1 mL/min, λ = 254 nm) as 95%, *t*_R_ (major) = 5.1 min, *t*_R_ (minor) = 4.5 min. ^1^H NMR (400 MHz, CDCl_3_) δ 7.74 (d, 1H, *J* = 8.2 Hz), 7.41 (dd, 1H, *J* = 7.8 Hz, *J* = 8.0 Hz), 7.16 (d, 2H, *J* = 8.5 Hz), 7.07 (dd, 1H, *J* = 7.6 Hz, *J* = 7.6 Hz), 7.02 (s, 2H), 6.95 (d, 2H, *J* = 8.4 Hz), 6.69 (d, 1H, *J* = 7.6 Hz), 5.19 (s, 1H), 4.83 (d, 1H, *J* = 14.0 Hz), 1.48 (s, 9H), 1.35 (s, 18H). ^13^C{^1^H} NMR (100 MHz, CDCl_3_)· δ 170.90 (d, *J* = 21.5 Hz), 153.30, 148.17, 140.88 (d, *J* = 5.5 Hz), 135.39, 134.73 (d, *J* = 7.6 Hz), 133.42, 131.61 (d, *J* = 2.9 Hz), 131.11, 128.42, 126.77 (d, *J* = 2.0 Hz), 126.27, 125.71, 124.21 (d, *J* = 2.8 Hz), 126.27, 125.71, 124.21 (d, *J* = 2.8 Hz), 123.75 (d, *J* = 19.6 Hz), 115.16, 93.79 (d, *J* = 195.0 Hz), 84.61, 60.36, 56.46 (d, *J* = 27.2 Hz), 34.37, 30.19, 27.85. ^19^F NMR (376 MHz, CDCl_3_) δ −145.96 (d, *J* = 14.1 Hz). HRMS (ESI-TOF) *m*/*z*: [M + Na]+ calcd for C_34_H_39_ClFNO_4_ 602.2444, found 602.2443. Melting point range = 63–72 °C.

## Data Availability

The data underlying this study are available in the published article and its Supporting Information.

## Supporting Information Available

Synthetic procedures, compound characterization, NMR spectra, HPLC chromatograms, and crystallographic information (PDF)

## References

[ref1] Müller K.; Faeh C.; Diederich F. Fluorine in Pharmaceuticals: Looking Beyond Intuition. Science 2007, 317, 1881–1886. 10.1126/science.1131943. 17901324

[ref2] Zhou Y.; Wang J.; Gu Z.; Wang S.; Zhu W.; Acena J. L.; Soloshonok V. A.; Izawa K.; Liu H. Next Generation of Fluorine-Containing Pharmaceuticals, Compounds Currently in Phase II–III Clinical Trials of Major Pharmaceutical Companies: New Structural Trends and Therapeutic Areas. Chem. Rev. 2016, 116, 422–518. 10.1021/acs.chemrev.5b00392. 26756377

[ref3] Smith B. R.; Eastman C. M.; Njardarson J. T. Beyond C, H, O, and N Analysis of the Elemental Composition of U.S. FDA Approved Drug Architectures. J. Med. Chem. 2014, 57, 9764–9773. 10.1021/jm501105n. 25255063

[ref4] Liang T.; Neumann C. N.; Ritter T. Introduction of Fluorine-containing Functional Groups. Angew. Chem., Int. Ed. 2013, 52, 8214–8264. 10.1002/anie.201206566. 23873766

[ref5] Cahard D.; Xu X.; Couve-Bonnaire S.; Pannecoucke X. Fluorine & Chirality: How to Create a Nonracemic Stereogenic Carbon–fluori-ne Centre?. Chem. Soc. Rev. 2010, 39, 558–568. 10.1039/B909566G. 20111780

[ref6] Yang X.; Wu T.; Phipps R. J.; Toste F. D. Advances in Catalytic Enantioselective Fluorination, Mono-, Di-, and Trifluoromethylation, and Trifluoromethylthiolation Reactions. Chem. Rev. 2015, 115, 826–870. 10.1021/cr500277b. 25337896 PMC4311656

[ref7] Champagne P. A.; Desroches J.; Hamel J.-D.; Vandamme M.; Paquin J.-F. Monofluorination of Organic Compounds: 10 Years of Innovation. Chem. Rev. 2015, 115, 9073–9174. 10.1021/cr500706a. 25854146

[ref8] Zhu Y.; Han J.; Wang J.; Shibata N.; Sodeoka M.; Soloshonok V. A.; Coelho J. A. S.; Toste F. D. Modern Approaches for Asymmetric Construction of Carbon–Fluorine Quaternary Stereogenic Centers: Synthetic Challenges and Pharmaceutical Needs. Chem. Rev. 2018, 118, 3887–3964. 10.1021/acs.chemrev.7b00778. 29608052 PMC6497456

[ref9] Liu J.; Yuan Q.; Toste F. D.; Sigman M. S. Enantioselective Construction of Remote Tertiary Carbon–Fluorine Bonds. Nat. Chem. 2019, 11, 710–715. 10.1038/s41557-019-0289-7. 31308495 PMC6679931

[ref10] Xu H.; Wolf C. Synthesis of Chiral Tertiary Trifluoromethyl Alcohols by Asymmetric Nitroaldol Reaction with a Cu(II)-Bisoxazolidine Catalyst. Chem. Commun. 2010, 46, 8026–8028. 10.1039/c0cc02378g. 20859576

[ref11] Wolf C.; Zhang P. Asymmetric Friedel-Crafts Reaction of Indoles with Ethyl Trifluoropyruvate Using a Copper(I)-Bisoxazolidine Catalyst. Adv. Synth. Catal. 2011, 353, 760–766. 10.1002/adsc.201000918.

[ref12] Zhang P.; Wolf C. Catalytic Enantioselective Difluoroalkylation of Aldehydes. Angew. Chem., Int. Ed. 2013, 52, 7869–7873. 10.1002/anie.201303551. 23780866

[ref13] Cook A. M.; Wolf C. Efficient Access to Multifunctional Trifluoromethyl Alcohols through Base-Free Catalytic Asymmetric C–C Bond Formation with Terminal Ynamides. Angew. Chem., Int. Ed. 2016, 55, 2929–2933. 10.1002/anie.201510910. PMC4806781 26806871

[ref14] Balaraman K.; Wolf C. Catalytic Enantioselective and Diastereoselective Allylic Alkylation with Fluoroenolates: Efficient Access to C3-Fluorinated and All-Carbon Quaternary Oxindoles. Angew. Chem., Int. Ed. 2017, 56, 1390–1395. 10.1002/anie.201608752. PMC5289271 28026079

[ref15] Balaraman K. Ding. R.; Wolf C. Stereoselective Synthesis of 3,3′-Bisindolines by Organocatalytic Michael Additions of Fluorooxindole Enolates to Isatylidene Malononitriles in Aqueous Solution. Adv. Synth. Catal. 2017, 359, 4165–4169. 10.1002/adsc.201701107. 29755308 PMC5939588

[ref16] Ding R.; Wolf C. Organocatalytic Asymmetric Synthesis of α-Oxetanyl and α-Azetidinyl Tertiary Alkyl Fluorides and Chlorides. Org. Lett. 2018, 20, 892–895. 10.1021/acs.orglett.8b00039. 29360370 PMC5937693

[ref17] Ding R.; De los Santos Z. A.; Wolf C. Catalytic Asymmetric Mannich Reaction of α-Fluoronitriles with Ketimines: Enantioselective and Diastereodivergent Construction of Vicinal Tetrasubstituted Stereocenters. ACS Catal. 2019, 9, 2169–2176. 10.1021/acscatal.8b05164. 30956891 PMC6449049

[ref18] Sripada A.; Wolf C. Catalytic Asymmetric Allylic Alkylation with Arylfluoroacetonitriles. J. Org. Chem. 2022, 87, 11880–11887. 10.1021/acs.joc.2c01414. 35975680 PMC9444989

[ref19] Gribkoff V. K.; Starrett J. E. Jr.; Dworetzky S. L.; Hewawasam P.; Boissard C. G.; Cook D. A.; Frantz S. W.; Heman K.; Hibbard J. R.; Huston K.; Johnson G.; Krishnan B. S.; Kinney G. G.; Lombardo L. A.; Meanwell N. A.; Molinoff P. B.; Myers R. A.; Moon S. L.; Ortiz A.; Pajor L.; Pieschl R. L.; Post-Munson D. J.; Signor L. J.; Srinivas N.; Taber M. T.; Thalody G.; Trojnacki J. T.; Wiener H.; Yeleswaram K.; Yeola S. W. Targeting Acute Ischemic Stroke with a Calcium-sensitive Opener of Maxi-K Potassium Channels. Nat. Med. 2001, 7, 471–477. 10.1038/86546. 11283675

[ref20] Hewawasam P.; Gribkoff V. K.; Pendri Y.; Dworetzky S. I.; Meanwell N. A.; Martinez E.; Boissard C. G.; Post-Munson D. J.; Trojnacki J. T.; Yeleswaram K.; Pajor L. M.; Knipe J.; Gao Q.; Perrone R.; Starrett J. E. Jr. The Synthesis and Characterization of BMS-204352 (MaxiPost) and Related 3-Fluorooxindoles as Openers of Maxi-K Potassium Channels. Bioorg. Med. Chem. Lett. 2002, 12, 1023–1026. 10.1016/S0960-894X(02)00101-4. 11909708

[ref21] Ranganath P. L. N.; Narsaiah A. V. Design, Synthesis and in Silico Studies of 3-Fluoro-3-substituted Oxindoles Against Cancer Targets. J. Fluorine Chem. 2023, 268, 110134 10.1016/j.jfluchem.2023.110134.

[ref22] Wang T.; Hoon D. L.; Lu Y. Enantioselective Synthesis of 3-Fluoro-3-allyl-oxindoles via Phosphine-catalyzed Asymmetric γ-Addition of 3-Fluoro-oxindoles to 2,3-Butadienoates. Chem. Commun. 2015, 51, 10186–10189. 10.1039/C5CC03289J. 26013076

[ref23] Dou X.; Lu Y. Enantioselective Conjugate Addition of 3-Fluoro-oxindoles to Vinyl Sulfone: An Organocatalytic Access to Chiral 3-Fluoro-3-substituted Oxindoles. Org. Biomol. Chem. 2013, 11, 5217–5221. 10.1039/c3ob41267a. 23842569

[ref24] Kim Y. S.; Kwon S. J.; Kim D. Y. Organocatalytic Enantioselective Conjugate Addition of 3-Fluorooxindoles to Vinyl Sulfone. Bull. Korean Chem. Soc. 2015, 36, 1512–1515. 10.1002/bkcs.10263.

[ref25] Chen X.; Li Y.; Zhao J.; Zheng B.; Lu Q.; Ren X. Stereoselective Mannich Reaction of *N*-(*tert*-Butylsulfinyl)imines with 3-Fluorooxindoles and Fluoroacetamides. Adv. Synth. Catal. 2017, 359, 3057–3062. 10.1002/adsc.201700353.

[ref26] Zhu Y.; Mei H.; Han J.; Soloshonok V. A.; Zhou J.; Pan Y. Chemoselective S_N_2′ Allylations of Detrifluoroacetylatively In Situ Generated 3-Fluoroindolin-2-one-Derived Tertiary Enolates with Morita–Baylis–Hillman Carbonates. J. Org. Chem. 2017, 82, 13663–13670. 10.1021/acs.joc.7b02409. 29178786

[ref27] Jin Y.; Chen M.; Ge S.; Hartwig J. F. Palladium-Catalyzed, Enantioselective α-Arylation of α-Fluorooxindoles. Org. Lett. 2017, 19, 1390–1393. 10.1021/acs.orglett.7b00294. 28263071 PMC11651418

[ref28] Paladhi S.; Park S. Y.; Yang J. W.; Song C. E. Asymmetric Synthesis of α-Fluoro-β-Amino-oxindoles with Tetrasubstituted C–F Stereogenic Centers via Cooperative Cation-Binding Catalysis. Org. Lett. 2017, 19, 5336–5339. 10.1021/acs.orglett.7b02628. 28953402

[ref29] Zhu Y.; Mao Y.; Mei H.; Pan Y.; Han J.; Soloshonok V. A.; Hayashi T. Palladium-Catalyzed Asymmetric Allylic Alkylations of Colby Pro-Enolates with MBH Carbonates: Enantioselective Access to Quaternary C–F Oxindoles. Chem.—Eur. J. 2018, 24, 8994–8998. 10.1002/chem.201801670. 29683211

[ref30] Hajra S.; Hazra A.; Mandal P. Stereocontrolled Nucleophilic Fluorination at the Tertiary sp^3^-Carbon Center for Enantiopure Synthesis of 3-Fluorooxindoles. Org. Lett. 2018, 20, 6471–6475. 10.1021/acs.orglett.8b02777. 30303391

[ref31] Zhao J.; Li Y.; Chen L.-Y.; Ren X. Enantioselective Mannich Reactions of 3-Fluorooxindoles with Cyclic N -Sulfamidate Aldimines. J. Org. Chem. 2019, 84, 5099–5108. 10.1021/acs.joc.9b00007. 30977656

[ref32] Li B.-Y.; Lin Y.; Du D.-M. Organocatalytic Asymmetric Mannich Addition of 3-Fluorooxindoles to Dibenzo[b,f][1,4]oxazepines: Highly Enantioselective Construction of Tetrasubstituted C–F Stereocenters. J. Org. Chem. 2019, 84, 11752–11762. 10.1021/acs.joc.9b01507. 31408331

[ref33] Qiu Z.-B.; Chen L.-Y.; Ji J.; Ren X.; Li Y. Highly Diastereoselective Aldol Reactions of 3-Fluorooxindoles Promoted by MgBr_2_•OEt_2_/*i*Pr_2_Net. J. Fluorine Chem. 2020, 236, 109594 10.1016/j.jfluchem.2020.109594.

[ref34] Butcher T. W.; Amberg W. M.; Hartwig J. F. Transition-Metal-Catalyzed Monofluoroalkylation: Strategies for the Synthesis of Alkyl Fluorides by C–C Bond Formation. Angew. Chem., Int. Ed. 2022, 61, e202112251 10.1002/anie.202112251. 34658121

[ref35] Liu Y.-L.; Wang X.-P.; Wei J.; Li Y. Synthesis of Oxindoles Bearing a Stereogenic 3-Fluorinated Carbon Center from 3-Fluorooxindoles. Org. Biomol. Chem. 2022, 20, 538–552. 10.1039/D1OB01964C. 34935824

[ref36] Sun C.; Nong Y.; Pang C.; Zhang S.; Li T. Carbene-Catalyzed Regioselective Addition of Oxindoles to Ynals for Quick Access to Allenes. Synlett 2023, 34, 1997–2000. 10.1055/s-0042-1752718.

[ref37] Parra A.; Tortosa M. *para*-Quinone Methide: a New Player in Asymmetric Catalysis. ChemCatChem. 2015, 7, 1524–1526. 10.1002/cctc.201500176.

[ref38] Xie K.-X.; Zhang Z.-P.; Li X. Bismuth Triflate-Catalyzed Vinylogous Nucleophilic 1,6-Conjugate Addition of *para*-Quinone Methides with 3-Propenyl-2-silyloxyindoles. Org. Lett. 2017, 19, 6708–6711. 10.1021/acs.orglett.7b03433. 29182289

[ref39] Rahman A.; Zhou Q.; Lin X. Asymmetric Organocatalytic Synthesis of Chiral 3,3-Disubstituted Oxindoles via a 1,6-Conjugate Addition Reaction *Org*. Biomol. Chem. 2018, 16, 5301–5309. 10.1039/C8OB01169A. 29993076

[ref40] Deng Y.; Chu W.; Shang Y.; Yu K.; Jia Z.; Fan C. P(NMe_2_)_3_-Mediated Umpolung Spirocyclopropanation Reaction of *p*-Quinone Methides: Diastereoselective Synthesis of Spirocyclopropane-Cyclohexadienones *Org*. Lett. 2020, 22, 8376–8381. 10.1021/acs.orglett.0c02998. 33044082

[ref41] Wang J.-Y.; Hao W.-J.; Tu S.-J.; Jiang B. Recent Developments in 1,6-Addition Reactions of *para*-Quinone Methides (p-QMs). Org. Chem. Front. 2020, 7, 1743–1778. 10.1039/D0QO00387E.

[ref42] Lima C. G. S.; Pauli F. P.; Costa D. C. S.; de Souza A. S.; Forezi L. S. M.; Ferreira V. F.; de Carvalho da Silva F. *para*-Quinone Methides as Acceptors in 1,6-Nucleophilic Conjugate Addition Reactions for the Synthesis of Structurally Diverse Molecules. Eur. J. Org. Chem. 2020, 2650–2692. 10.1002/ejoc.201901796.

[ref43] Tan Q.; Guo N.; Yang L.; Wang F.; Feng X.; Liu X. Asymmetric Organocatalytic 1,6-Conjugate Addition of *para*-Quinone Methides Using [1,2]-Phospha-Brook Rearrangement. J. Org. Chem. 2023, 88, 9332–9342. 10.1021/acs.joc.3c00910. 37347936

[ref44] Zhao K.; Zhi Y.; Wang A.; Enders D. Asymmetric Organocatalytic Synthesis of 3-Diarylmethine-Substituted Oxindoles Bearing a Quaternary Stereocenter via 1,6-Conjugate Addition to *para*-Quinone Methides. ACS Catal. 2016, 6, 657–660. 10.1021/acscatal.5b02519.

[ref45] Deng Y.; Zhang X.; Yu K.; Yan X.; Du J.; Huang H.; Fan C. Bifunctional Tertiary Amine-squaramide Catalyzed Asymmetric Catalytic 1,6-Conjugate Addition/aromatization of *para*-Quinone Methides with Oxindoles. Chem. Commun. 2016, 52, 4183–4186. 10.1039/C5CC10502A. 26908307

[ref46] Yuan A.; Steber S. E.; Xhili D.; Nelson E.; Wolf C. Enantioseparation and Racemization of 3-Fluorooxindoles. Chirality 2023, 35, 619–624. 10.1002/chir.23572. 37129272 PMC10516598

