# Supplementary material for: Organocatalytic Asymmetric Conjugate Addition of Fluorooxindoles to Quinone Methides
Source: J Org Chem. 2024 Apr 15;89(9):6100–5. doi: 10.1021/acs.joc.4c00062 (PMC11077483; doi:10.1021/acs.joc.4c00062)
Supplement: Supplementary file 1 — jo4c00062_si_001.pdf [file jo4c00062_si_001.pdf]

Organocatalytic Asymmetric Conjugate Addition of Fluorooxindoles to Quinone Methides

Maria Bouda, Jeffery A. Bertke and Christian Wolf*

Department of Chemistry, Georgetown University, Washington, DC 20057, USA

Table of Contents

1. **General information** **S2**

2. **Optimization studies** **S3**

3. **Synthesis procedures and compound characterization** **S8**

    3.1. **Synthesis of *para*-quinone methides** **S8**

    3.2. **General phase transfer catalysis procedure** **S10**

    3.3. **Boc deprotection of compound 4** **S27**

    3.4. **Upscaling of the general phase transfer catalysis procedure** **S28**

4. **Determination of absolute configuration** **S29**

5. **Crystallographic data** **S30**

6. **^{1}H, ^{13}C, ^{19}F NMR spectra of newly reported compounds** **S34**

7. **^{1}H NMR spectra of known compounds** **S90**

8. **HPLC chromatograms** **S107**

9. **References** **S143**

1. General information

All commercially available reagents and solvents were used without further purification unless noted otherwise. **PTC-1**, **2** and **3** are derived from the cinchona alkaloid cinchonidine. All catalysts used in this study were purchased from Sigma. 2.6-Di-*tert*-butylphenol, the aldehydes, phenols and oxindoles were obtained from Sigma with the following exceptions: 2-naphthaldehyde (TCI), 3-pyridinecarboxaldehyde (TCI), 2,6-di-*tert*-butyl-4-ethylphenol (TCI) and 5-methoxyoxindole (Ambeed). The *N*-Boc-3-fluorooxindoles and *N*-benzyl-3-fluorooxindole were prepared via literature procedures.[1,2,3] Solvents were stored over 4Å molecular sieves prior to use. Reaction products were purified by column chromatography on silica gel (particle size 32-63 µm) as described below. NMR spectra were obtained at 400 MHz (^{1}H NMR), 100 MHz (^{13}C NMR), and 376 MHz (^{19}F NMR) in $CDCl_3$. Chemical shifts are reported in ppm relative to the chloroform signal. All 1,6-addition reaction products were prepared in racemic form to develop a chiral HPLC method. The isolated asymmetric reaction products were then analyzed accordingly. HR-MS data were obtained using electron spray ionization time-of-flight (ESI-TOF) spectrometry. Commercially available compounds were used without further purification.

2. Optimization studies

Optimization studies were performed to identify optimal conditions with monofluorinated oxindoles. All optimization reactions were conducted at 0.06 mmol scale with 10 mol% of the catalyst, temperatures varying between -78 and 0 ºC, 1-5 equivalents of base and solvents typically used for phase transfer catalysis.

Catalysts used in this study

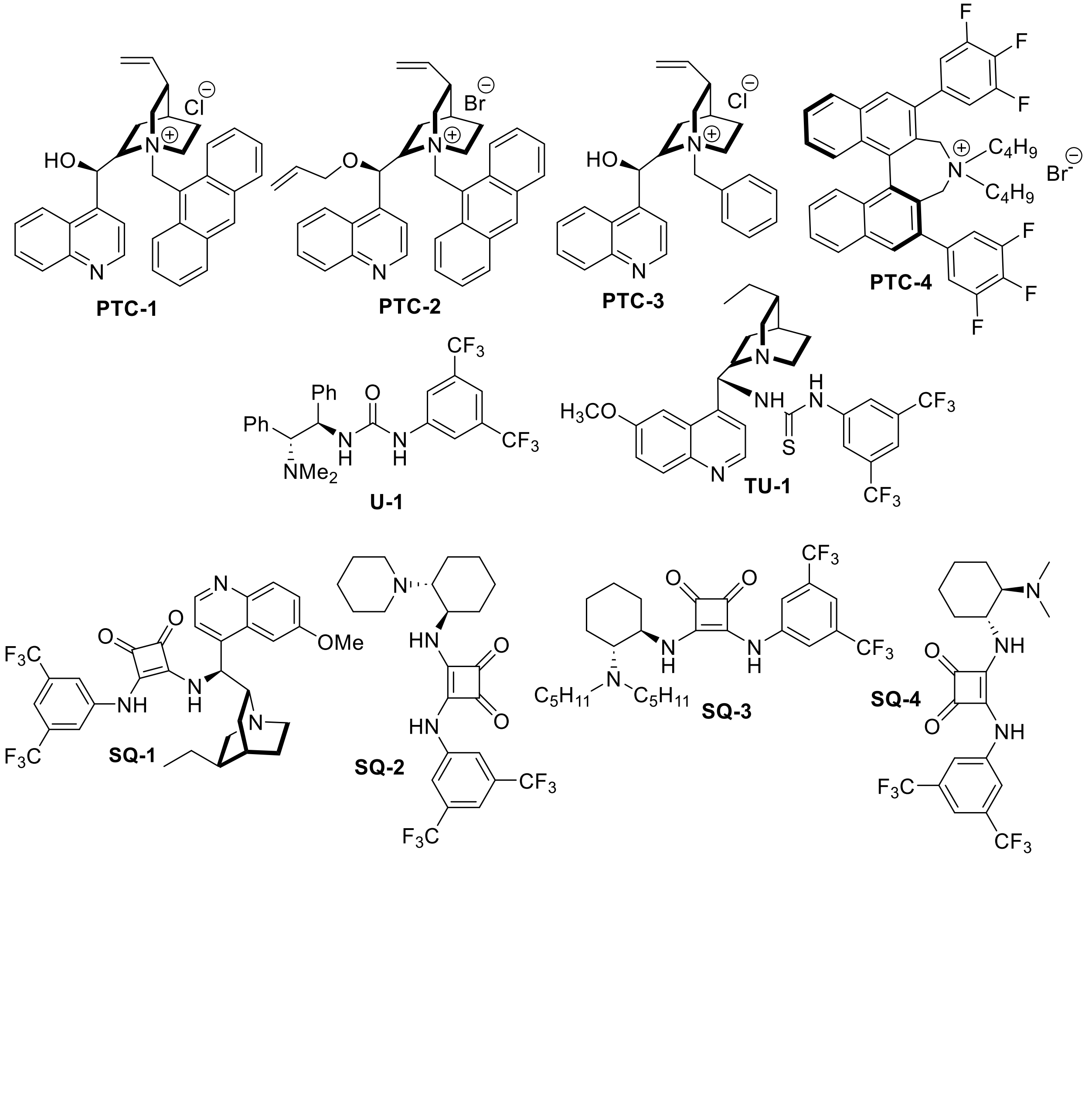

Table S1. Screening with *N*-methyl-3-fluorooxindole.

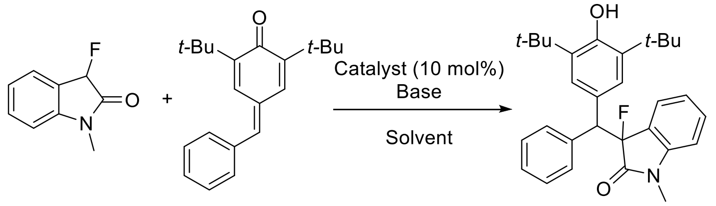

Catalyst	Base	Solvent	Time	Temp. (°C)	Conversion[b]	*dr*[c]	*ee*[d]
**PTC-1**	K_2CO_3 1eq	Toluene	20 h	0	99%	2 : 1	12
**PTC-1**	Cs_2CO_3 1eq	DCM	20 h	0	99%	2 : 1	3
**PTC-2**	K_2CO_3 1eq	1:1 DCM/H_2O	72 h	0	95%	2 : 1	5
**PTC-1**	K_2CO_3 1eq	DCM	24 h	-40	93%	2 : 1	12
**PTC-1**	K_2CO_3 1eq	Toluene	24 h	-40	85%	3 : 1	5
**PTC-1**	K_2CO_3 1eq	DCM	24 h	0	87%	2 : 1	9
**PTC-1**	K_2CO_3 1eq	1:1 DCM/H_2O	20 h	0	92%	2 : 1	0
**PTC-1**	K_2CO_3 1eq	1:1 DCM/H_2O	24 h	0	95%	2 : 1	0
**PTC-2**	K_2CO_3 1eq	Toluene	24 h	-40	0%	n.d.	n.d.
**PTC-1**	AcOK 1eq	Toluene	24 h	-40	0%	n.d.	n.d.
**PTC-1**	$PhCO_2Na$ 5eq	Toluene	24 h	-40	0%	n.d.	n.d.
**PTC-1**	AcONa 5eq	Toluene	24 h	-40	0%	n.d.	n.d.
**PTC-1**	Na_2HPO_4 1eq	Toluene	24 h	-40	0%	n.d.	n.d.
**PTC-1**	$KHCO_3$ 5eq	Toluene	24 h	-40	0%	n.d.	n.d.
**PTC-2**	Cs_2CO_3 1.5eq	DBE	20 h	-40	99%	2 : 1	33
**SQ-1**	n/a	ACN	24 h	25	30%	3 : 1	3
**TU-1**	n/a	ACN	45 h	25	50%	2 : 1	0
**TU-1**	n/a	DCM	65 h	25	50%	2 : 1	0
**SQ-3**	n/a	ACN	45 h	25	20%	n.d.	0%
**SQ-1**	n/a	DCM	24 h	25	5%	n.d.	n.d.
**SQ-3**	n/a	DCM	24 h	25	3%	n.d.	n.d.
**SQ-4**	n/a	ACN	72 h	25	0%	n.d.	n.d.
**SQ-2**	n/a	ACN	72 h	25	0%	n.d.	n.d.
**SQ-3**	K_2CO_3 1.5eq	DCM	24 h	0	80%	4 : 1	11
**SQ-1**	K_2CO_3 1.5eq	DCM	24 h	0	85%	3 : 1	3
**TU-1**	Cs_2CO_3 1.5eq	Dioxane	20 h	0	99%	1 : 1	0
**PTC-1**	K_2CO_3 1eq	Toluene	20 h	-40	0%	n.d.	n.d.[a]

Reaction conditions: *N*-methyl-3-fluoro-2-oxindole (10.0 mg, 0.06 mmol) and *para*-quinone methide (18.0 mg, 0.06 mmol) were dissolved in the indicated solvent (0.2 mL) and the indicated base was added. Upon completion, the reaction was extracted with a saturated solution of ammonium chloride and dichloromethane before being concentrated for spectroscopic and chromatographic analysis. [a]3-Fluorooxindole was used. [b]Determined by ^{1}H NMR. [c]Determined by ^{19}F NMR. [d]Determined by chiral HPLC. DCM = dichloromethane. DBE = dibutylether. ACN = acetonitrile.

Table S2. Screening with *N*-phenyl-3-fluorooxindole.

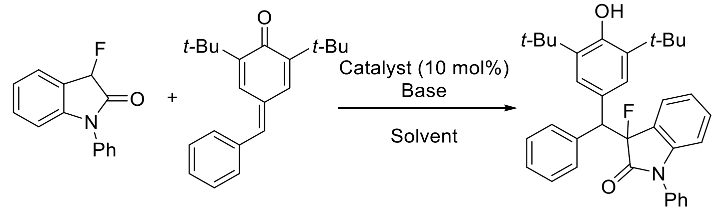

Catalyst	Base	Solvent	Time	Temp. (°C)	Conversion	*dr* [b]	*ee* [c]
**PTC-1**	K_2CO_3 1eq	Toluene	24 h	0	99%	8 : 1	7
**PTC-1**	K_2CO_3 1eq	Toluene	24 h	-40	99%	13 : 1	17
n/a	K_2CO_3 1eq	Toluene	48 h	0	0%	n.d.	n.d.
**TU-1**	Cs_2CO_3 1.5eq	Dioxane	24 h	0	99%	2 : 1	n.d.
**TU-1**	Cs_2CO_3 1.5eq	DBE	24 h	-40	36%	20 : 1	0
**TU-1**	Cs_2CO_3 1.5eq	DBE	24 h	-78	77%	1 : 1	n.d.
**PTC-2**	Cs_2CO_3 1.5eq	DBE	24 h	-40	99%	20 : 1	n.d.
n/a	Quinine 1eq	DCM	24 h	25	57%	1 : 1	10
n/a	(+)-sparteine 1eq	DBE	24 h	25	99%	2 : 1	0
**SQ-3**	K_2CO_3 1.5eq	DCM	72 h	-40	30%	n.d.	n.d.
n/a	Quinine 1eq	DCM	21 h	25	57%	1 : 1	10

Reaction conditions: *N*-phenyl-3-fluoro-2-oxindole (14.0 mg, 0.06 mmol) and *para*-quinone methide (18.2 mg, 0.06 mmol) were dissolved in the indicated solvent (0.2 mL) and the indicated base was added. Upon completion, the reaction was extracted with a saturated solution of ammonium chloride and dichloromethane before being concentrated for spectroscopic and chromatographic analysis. [a]Determined by ^{1}H NMR. [b]Determined by ^{19}F NMR. [c]Determined by chiral HPLC. DCM = dichloromethane. DBE = dibutylether.

Table S3. Screening of organocatalysts with *N*-Boc-3-fluorooxindole.

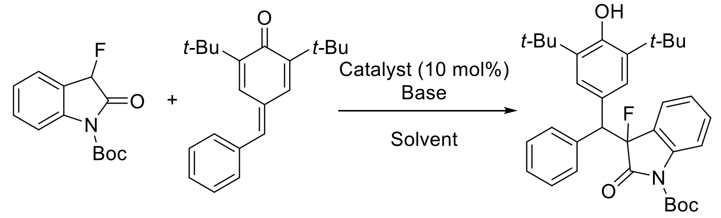

Catalyst	Base	Solvent	Time	Temp. (°C)	Conversion[a]	*dr*[b]	*ee*[c]
**U-1**	Cs_2CO_3 1.5eq	DCM	24 h	-40	99%	2 : 1	60
**SQ-1**	Cs_2CO_3 1.5eq	Dioxane	72 h	0	99%	1 : 1	0
**SQ-1**	n/a	DCM	24 h	-40	0%	n.d.	n.d.
**TU-1**	Cs_2CO_3 1.5eq	DBE	24 h	-40	99%	1 : 1	27
**TU-1**	Cs_2CO_3 1.5eq	Dioxane	10 h	-40	99%	2 : 1	0
**TU-1**	DBU	DBE	24 h	-40	99%	2 : 1	15
**TU-1**	Cs_2CO_3 1.5eq	DBE	24 h	-78	99%	2 : 1	39
**TU-1**	Cs_2CO_3 1.5eq	Dioxane	24 h	-78	99%	1 : 1	8
**TU-1**	KH_2PO_4 1.5eq	DBE	24 h	-40	0%	n.d.	n.d.
**TU-1**	$MgCO_3$ 1.5eq	DBE	24 h	-40	0%	n.d.	n.d.
**SQ-1**	Cs_2CO_3 1.5eq	DBE	24 h	-78	0%	n.d.	n.d.
**SQ-2**	Cs_2CO_3 1.5eq	DBE	24 h	-40	99%	1 : 1	9
**SQ-3**	Cs_2CO_3 1.5eq	DBE	24 h	-40	99%	1 : 1	31
**SQ-4**	Cs_2CO_3 1.5eq	DBE	24 h	-40	99%	1 : 1	5
**TU-1**	Cs_2CO_3 1.5eq	Dioxane	18 h	0C	99%	1 :1	0
**TU-1**	n/a	Dioxane	24 h	25	0%	n.d.	n.d.
**TU-1**	K_2CO_3 1eq	Dioxane	18 h	-40	0%	n.d.	n.d.
**TU-1**	Cs_2CO_3 1.5eq	DBE	10 h	-78	0%	n.d.	n.d.
**TU-1**	Cs_2CO_3 1.5eq	Toluene	24 h	-40	0%	n.d.	n.d.
**TU-1**	(+)-sparteine	DBE	24 h	-40	65%	20 : 1	n.d.

Reaction conditions: *N*-boc-3-fluoro-2-oxindole (15.0 mg, 0.06 mmol) and *para*-quinone methide (18.0 mg, 0.06 mmol) were dissolved in the indicated solvent (0.2 mL) and the indicated base was added. Upon completion, the reaction was extracted with a saturated solution of ammonium chloride and dichloromethane before being concentrated for spectroscopic and chromatographic analysis. [a]Determined by ^{1}H NMR. [b]Determined by ^{19}F NMR. [c]Determined by chiral HPLC. DCM = dichloromethane. DBE = dibutylether. Boc = *t*-butoxy carbonyl. DBU = 1,8-diazabicyclo[5.4.0]undec-7-ene.

Table S4. Screening of phase transfer catalysts with *N*-Boc-3-fluorooxindole.

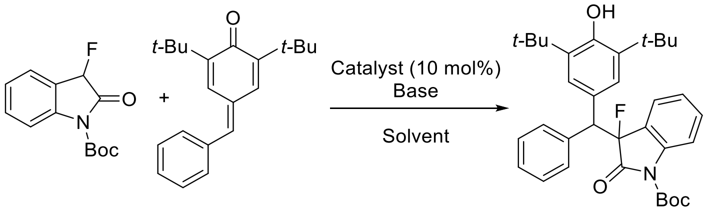

Catalyst	Base	Solvent	Time	Temp. (°C)	Conversion[a]	*dr*[b]	*ee*[c]
**PTC-3**	K_2CO_3 1eq	Toluene	24 h	-40	99%	3 : 1	81
**PTC-2**	Cs_2CO_3 1eq	Dioxane	18 h	0	99%	20 : 1	71
**PTC-2**	Cs_2CO_3 1.5eq	DBE	24 h	-40	99%	1 : 1	95
**PTC-1**	K_2CO_3 1.5eq	DBE	18 h	-40	n.d.	n.d.	n.d.
**PTC-1**	K_2CO_3 1eq	DCM	4 h	0	99%	20 : 1	20
**PTC-1**	Cs_2CO_3 1.5eq	MTBE	24 h	-40	99%	20 : 1	77
**PTC-1**	Cs_2CO_3 1.5eq	DBE	24 h	-40	99%	2 : 1	61
**PTC-1**	Cs_2CO_3 1.5eq	Glyme	24 h	-40	30%	20 : 1	13
**PTC-1**	Cs_2CO_3 1.5eq	1,2-Diethoxyethane	24 h	-40	n.d.	n.d.	n.d.
**PTC-1**	Cs_2CO_3 1.5eq	Ethyl diglyme	24 h	-40	99%	20 : 1	26
**PTC-1**	Cs_2CO_3 1.5eq	DBE	24 h	-78	99%	20 : 1	69
**PTC-1**	Cs_2CO_3 1eq	THF	4 h	0	99%	20 : 1	33
**PTC-1**	Cs_2CO_3 1eq	Ether	4 h	0	99%	20 : 1	47
**PTC-1**	K_2CO_3 1eq	Dioxane	24 h	0	99%	9 : 1	62
**PTC-1**	K_2CO_3 1eq	ACN	18 h	0	99%	20 : 1	0
**PTC-1**	K_2CO_3 1eq	EtOAc	18 h	0	99%	20 : 1	47
**PTC-1**	Cs_2CO_3 1eq	Dioxane	18 h	0	99%	3 : 1	59
**PTC-1**	K_2CO_3 1eq	Toluene	24 h	-40	80%	20 : 1	96
**PTC-4**	K_2CO_3 1eq	Toluene	24 h	-40	99%	20 : 1	43
**PTC-4**	K_2CO_3 1eq	1:1 Toluene/H_2O	18 h	-20	99%	20 : 1	55

Reaction conditions: *N*-methyl-3-fluoro-2-oxindole (15.0 mg, 0.06 mmol) and *para*-quinone methide (18.0 mg, 0.06 mmol) were dissolved in the indicated solvent (0.2 mL) and the indicated base was added. Upon completion, the reaction was extracted with a saturated solution of ammonium chloride and dichloromethane before being concentrated for spectroscopic and chromatographic analysis. [a]Determined by ^{1}H NMR. [b]Determined by ^{19}F NMR. [c]Determined by chiral HPLC. DCM = dichloromethane. DBE = dibutylether. MTBE = methyl *t*=butyl ether. THF = tetrahydrofuran. Boc = *t*-butoxy carbonyl. ACN = acetonitrile.

3. Synthesis procedures and compound characterization

3.1. Synthesis of *para*-quinone methides

A previously unreported *para*-quinone methide (2,6-di-*tert*-butyl-4-(2,3-dichlorobenzylidene)cyclohexa-2,5-dien-1-one) was prepared as described below. All other *para*-quinone methides were known and prepared following literature procedures.[3-12]

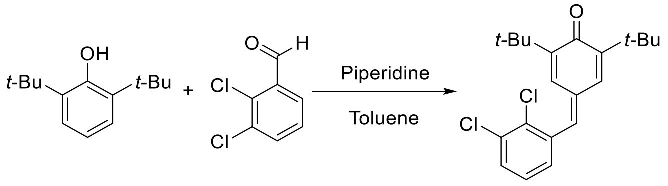

In an oven dried 3-neck flask, 2,6-di-*tert*-butylphenol (350.0 mg, 1.7 mmol) and 2,3-dichlorobenzaldehyde (300.0 mg, 1.7 mmol) were combined in toluene (5.0 mL) before adding piperidine (0.34 mL, 3.4 mmol) dropwise. The resulting mixture was refluxed under Dean Stark conditions for 12 hours. Upon completion, the mixture was brought to 100 ºC and acetic anhydride (0.32 mL, 3.4 mmol) was added. This mixture was stirred for an additional 45 minutes before extracting with dichloromethane and brine at room temperature. The combined organic layers were dried over sodium sulfate and purified via column chromatography as described below.

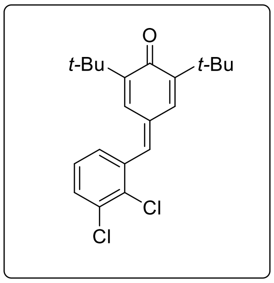

2,6-Di-*tert*-butyl-4-(2,3-dichlorobenzylidene)cyclohexa-2,5-dien-1-one was obtained as a yellow crystalline solid in 60% yield (380 mg, 1.1 mmol) after purification by flash chromatography using 100% hexanes as the mobile phase. ^{1}H NMR (400 MHz, $CDCl_3$) δ 7.50 (m, 1H), 7.28-7.29 (2H, m), 7.22-7.24 (m, 2H), 7.05 (s, 1H), 1.33 (s, 9H), 1.25 (s, 9H) $^{13}C\{^1H\}$ NMR (100MHz, $CDCl_3$) δ 186.5, 150.0, 148.6, 137.8, 136.3, 134.3, 133.3, 130.6, 130.2, 127.2, 127.0, 35.5, 35.1, 29.5. HRMS (ESI-TOF) m/z: $[M+H]^+$ calcd for $[C_{21}H_{24}Cl_2O]$ 363.1277, found 363.1276.

Melting point range = 79-81 ºC.

3.2. General phase transfer catalysis procedure

Figure S1. Asymmetric Michael addition of *N*-Boc-3-fluorooxindoles to *para*-quinone methides

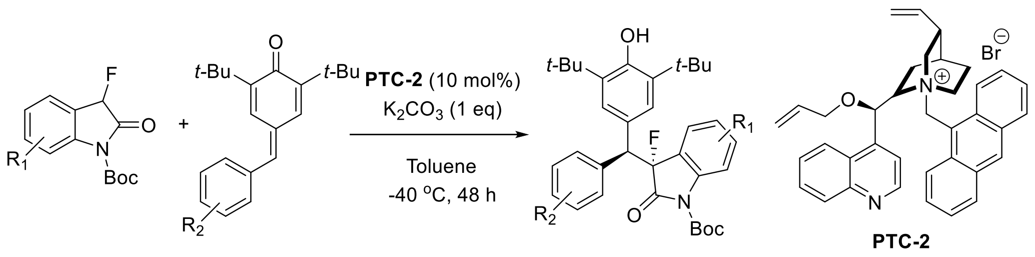

N-Boc-3-fluoro-2-oxindole (15.0 mg, 0.06 mmol), *para*-quinone methide (0.06 mmol), potassium carbonate (8.2 mg, 0.06 mmol), and **PTC-2** (10 mol%) were added to an oven-dried vial under nitrogen. Anhydrous toluene (200-600 μL) was added and the resulting mixture was stirred at -40 °C for 48 hours. Upon completion, the reaction mixture was quenched with an aqueous solution of saturated ammonium chloride and extracted with dichloromethane. The combined organic layers were dried over sodium sulfate, concentrated and purified via column chromatography as described below.

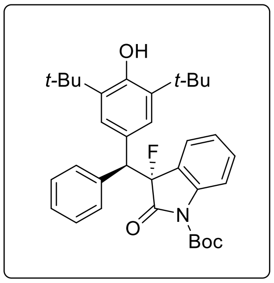

Compound **3** was obtained as a yellow crystalline solid in 98% isolated yield (32.1 mg, 0.06 mmol) from *N*-Boc-3-fluoro-2-oxindole (15.0 mg, 0.06 mmol) and 4-benzylidene-2,6-di-*tert*-butylcyclohexa-2,5-dien-1-one (17.5 mg, 0.06 mmol) following the general procedure described above after purification by flash chromatography using hexanes:ethyl acetate (98:2) as the mobile phase. The *dr* was determined as 29:1 using ^{1}H NMR spectroscopy. The *ee* was

determined by chiral HPLC ((*S,S*)-Whelk-O 1, hexanes/IPA, 99:1, flow rate 1 mL/min, λ=254 nm) as 94%, t_R (major)=9.8 min, t_R (minor)=11.3 min. ^{1}H NMR (400 MHz, $CDCl_3$) δ 7.76 (d, 1H, *J*=8.2 Hz), 7.41 (dd, 1H, *J*=7.8 Hz, *J*=7.9 Hz), 7.14-7.18 (m, 3H), 7.10 (s, 2H), 7.06 (dd, 1H, *J*=7.6 Hz, *J*=7.6 Hz), 6.94-6.98 (m, 2H), 6.63 (d, 1H, *J*=7.6 Hz), 5.17 (s, 1H), 4.86 (d, 1H, *J*=15.1 Hz), 1.45 (s, 9H), 1.36 (s, 18H). ^{13}C{^{1}H} NMR (100 MHz, $CDCl_3$) δ 171.0 (d, *J*=21.4 Hz), 154.2, 148.3, 141.1 (d, *J*=5.6 Hz), 136.1 (d, *J*=8.0 Hz), 135.2, 131.4 (d, *J*=3.3 Hz), 129.7, 128.3, 127.5, 127.0 (d, *J*=2.0 Hz), 126.4, 126.3, 124.1 (d, *J*=2.2 Hz), 124.0 (d, *J*=14.8), 115.1, 94.0 (d, *J*=194.1 Hz), 84.3, 57.0 (d, *J*=27.0 Hz), 34.4, 30.2, 27.8. ^{19}F NMR (376 MHz, $CDCl_3$) δ -145.4 (d, *J*=15.0 Hz). HRMS (ESI-TOF) m/z: $[M+Na]^+$ calcd for $C_{34}H_{40}FNO_4Na$ 568.2834, found 568.2827. Melting point range = 53-57 °C.

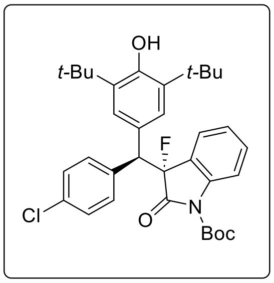

Compound **4** was obtained as a yellow crystalline solid in 99% isolated yield (34.0 mg, 0.06 mmol) from *N*-Boc-3-fluoro-2-oxindole (15.0 mg, 0.06 mmol) and 2,6-di-*tert*-butyl-4-(4-chlorobenzylidene)cyclohexa-2,5-dien-1-one (19.6 mg, 0.06 mmol) following the general procedure described above after purification by flash chromatography using hexanes:ethyl acetate (95:5) as the mobile phase. The *dr* was determined as 48:1 using ^{1}H NMR spectroscopy. The *ee* was determined by chiral HPLC (Chiralpak IA, 98:2 hexanes/ IPA, flow rate 1 mL/min, λ=254 nm) as 91%, t_R (major)= 5.1 min, t_R (minor)= 4.5 min. ^{1}H NMR (400 MHz, $CDCl_3$) δ 7.74 (d, 1H, *J*=8.2 Hz), 7.41 (dd, 1H, *J*=7.8 Hz, *J*=8.0 Hz), 7.16 (d, 2H, *J*=8.5 Hz), 7.07 (dd, 1H, *J*=7.6 Hz, J=7.6 Hz), 7.02 (s, 2H), 6.95 (d, 2H, *J*=8.4 Hz), 6.69 (d, 1H, *J*=7.6 Hz), 5.19 (s, 1H),

4.83 (d, 1H, *J*=14.0 Hz), 1.48 (s, 9H), 1.35 (s, 18H). $^{13}C\{^1H\}$ NMR (100 MHz, $CDCl_3$) δ 170.9 (d, *J*=21.5 Hz), 153.3, 148.2, 140.9 (d, *J*=5.5 Hz), 135.4, 134.7 (d, *J*=7.6 Hz), 133.4, 131.6 (d, *J*=2.9 Hz), 131.1, 128.4, 126.8 (d, *J*=2.0 Hz), 126.3, 125.7, 124.2 (d, *J*=2.8 Hz), 126.3, 125.7, 124.2 (d, *J*=2.8 Hz), 123.8 (d, *J*=19.6 Hz), 115.2, 93.8 (d, *J*=195.0 Hz), 84.6, 60.4, 56.5 (d, *J*=27.2 Hz), 34.4, 30.2, 27.9. ^{19}F NMR (376 MHz, $CDCl_3$) δ -146.0 (d, *J*=14.1 Hz). HRMS (ESI-TOF) m/z: $[M+Na]^+$ calcd for $C_{34}H_{39}ClFNO_4Na$ 602.2444, found 602.2443. Melting point range= 63-72 ºC.

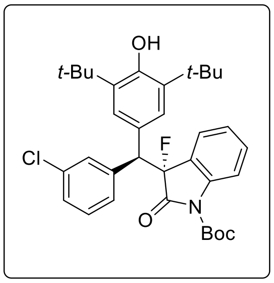

Compound **5** was obtained as a yellow amorphous solid in 99% isolated yield (36.0 mg, 0.06 mmol) from *N*-Boc-3-fluoro-2-oxindole (15.0 mg, 0.06 mmol) and 2,6-di-*tert*-butyl-4-(3-chlorobenzylidene)cyclohexa-2,5-dien-1-one (19.6 mg, 0.06 mmol) following the general procedure described above after purification by flash chromatography using hexanes:ethyl acetate (95:5) as the mobile phase. The *dr* was determined as 14:1 using ^1H NMR spectroscopy. The *ee* was determined by chiral HPLC ((*S,S)*-Whelk-O 1, 98:2 hexanes/IPA, flow rate 1 mL/min, λ=254 nm) as 83%, t_R (major)= 7.5 min, t_R (minor)=8.5 min. ^1H NMR (400 MHz, $CDCl_3$) δ 7.76 (d, 1H, *J*=8.3 Hz), 7.44 (dd, 1H, *J*= 7.7 Hz, *J*=7.8 Hz), 7.19-7.08 (m, 3H), 7.05 (s, 1H), 7.02 (s, 2H), 6.93 (d, 1H, *J*=7.4 Hz), 6.72 (d, 1H, *J*=7.6 Hz), 5.20 (s, 1H), 4.82 (d, 1H, *J*=13.9 Hz), 1.50 (s, 9H), 1.35 (s, 18H). $^{13}C\{^1H\}$ NMR (100 MHz, $CDCl_3$). δ 170.8 (d, *J*=21.6 Hz), 153.3, 148.1, 140.8 (d, *J*=5.4 Hz), 138.3 (d, *J*=7.5 Hz), 135.4, 134.1, 131.6 (d, *J*=2.9 Hz), 130.2, 129.4, 127.6 (d, *J*=2.3 Hz), 126.2, 125.5, 124.2 (d, *J*=2.7 Hz), 123.8 (d, *J*=19.6 Hz), 115.1,

93.7 (d, *J*=195.2 Hz), 84.6, 56.8 (d, *J*=27.3 Hz), 34.3, 30.2, 27.8. ^{19}F NMR (376 MHz, $CDCl_3$) δ -146.3 (d, *J*=13.8 Hz) HRMS (ESI-TOF) m/z: $[M+Na]^+$ calcd for $C_{34}H_{39}ClFNO_4Na$ 602.2444, found 602.2444.

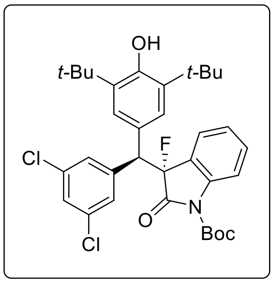

Compound **6** was obtained as a white crystalline solid in 98% isolated yield (36.0 mg, 0.06 mmol) from *N*-Boc-3-fluoro-2-oxindole (15.0 mg, 0.06 mmol) and 2,6-di-*tert*-butyl-4-(3,5-dichlorobenzylidene)cyclohexa-2,5-dien-1-one (21.7 mg, 0.06 mmol) following the general procedure described after purification by flash chromatography using hexanes:ethyl acetate (95:5) as the mobile phase. The *dr* was determined as 26:1 using ^{1}H NMR spectroscopy. The *ee* was determined by chiral HPLC ((*S,S*)-Whelk-O 1, hexanes/IPA, 98:2, flow rate 1 mL/min, λ=254 nm) as 85%, t_R (major)= 6.3 min, t_R (minor)= 7.2 min. ^{1}H NMR (400 MHz, $CDCl_3$) δ 7.75 (d, 1H, *J*=8.2 Hz), 7.44 (dd, 1H, *J*=7.6, *J*=7.8), 7.23 (s, 1H), 7.13 (dd, 1H, *J*= 7.5 Hz, *J*=7.5 Hz), 7.05 (s, 2H), 6.93 (s, 2H), 6.84 (d, 1H, *J*=7.6 Hz), 5.21 (s, 1H), 4.77 (d, 1H, *J*=12.3 Hz), 1.53 (s, 9H), 1.35 (s, 18H) $^{13}C\{^1H\}$ NMR (100 MHz, $CDCl_3$) 170.6 (d, *J*=21.7 Hz), 153.5, 148.0, 140.7 (d, *J*=5.3 Hz), 139.9 (d, *J*=6.1 Hz), 135.6, 134.7, 131.8 (d, *J*=3.0 Hz), 128.3 (d, *J*=1.3 Hz), 127.5, 126.6 (d, *J*=1.8 Hz), 126.0, 124.8, 124.4 (d, *J*=2.7 Hz), 123.7 (d, *J*=19.5 Hz), 115.2, 93.5 (d, *J*=196.5 Hz), 84.8, 56.8 (d, *J*= 27.7 Hz), 34.3, 30.1, 27.9. ^{19}F NMR (376 MHz, $CDCl_3$) δ -146.9 (d, *J*=12.1 Hz). HRMS (ESI-TOF) m/z: $[M+Na]^+$ calcd for $C_{34}H_{38}Cl_2FNO_4Na$ 636.2054, found 636.2054. Melting point range = 61-66 °C.

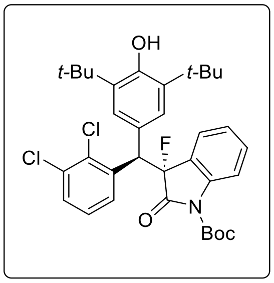

Compound **7** was obtained as a white crystalline solid in 85% isolated yield (31.0 mg, 0.05 mmol) from *N*-Boc-3-fluoro-2-oxindole (15.0 mg, 0.06 mmol) and 2,6-di-*tert*-butyl-4-(2,3-dichlorobenzylidene)cyclohexa-2,5-dien-1-one (21.7 mg, 0.06 mmol) following the general procedure described above for 4 days at -20 ºC after purification by flash chromatography using hexanes:ethyl acetate (95:5) as the mobile phase. The *dr* was determined as 20:1 using ^{19}F NMR spectroscopy. The *ee* was determined by chiral HPLC (Amylose-1, hexanes/IPA, 99:1, flow rate 1 mL/min, λ=254 nm) as 88%, t_R (major) =4.5 min, t_R (minor) =4.9 min. ^{1}H NMR (400 MHz, $CDCl_3$) δ 7.74 (d, 1H, *J*=8.2 Hz), 7.41-7.48 (m, 2H), 7.34 (d, 1H, *J*=8.0 Hz), 7.10-7.15 (m, 2H), 6.91 (d, 1H, *J*=7.7 Hz), 6.85 (s, 2H), 5.51 (d, 1H, *J*=12.1 Hz), 5.15 (s, 1H), 1.51 (s, 9H), 1.30 (s, 18H) . ^{13}C{^{1}H} NMR (100 MHz, $CDCl_3$) δ 170.6 (d, *J*=21.8 Hz), 153.3, 148.2, 140.7 (d, *J*=5.5 Hz), 137.5 (d, *J*=5.4 Hz), 135.3, 133.8, 133.4, 131.7 (d, *J*=2.9 Hz), 129.2, 127.7 (d, *J*=1.9 Hz), 127.0 (d, *J*=1.5 Hz), 126.5, 126.0, 124.7 (d, *J*=19.5 Hz), 124.6 (d, *J*=2.8 Hz), 124.2 (d, *J*=1.5 Hz), 115.1, 93.9 (d, *J*= 195.8 Hz), 84.7, 53.9 (d, *J*=28.6 Hz), 34.3, 30.1, 27.9. ^{19}F NMR (376 MHz, $CDCl_3$) δ -144.6 (d, *J*=12.2 Hz) HRMS (ESI-TOF) m/z: $[M+Na]^+$ calcd for $C_{34}H_{38}FCl_2NO_4Na$ 636.2060, found 636.2056. Melting point range = 70-75 ºC.

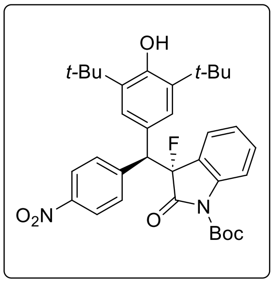

Compound **8** was obtained as a yellow amorphous solid in 86% isolated yield (35.0 mg, 0.06 mmol) from *N*-Boc-3-fluoro-2-oxindole (15.0 mg, 0.06 mmol) and 2,6-di-*tert*-butyl-4-(4-nitrobenzylidene)cyclohexa-2,5-dien-1-one (20.3 mg, 0.06 mmol) following the general procedure described above after purification by flash chromatography using hexanes:ethyl acetate (95:5) as the mobile phase. The *dr* was determined as 20:1 using ^{1}H NMR spectroscopy. The *ee* was determined by chiral HPLC (Chiralpak AD-H, 97:3 hexanes:IPA, flow rate 1 mL/min, λ=254 nm) as 94%, t_R (major)= 5.4 min, t_R (minor)= 24.8 min. ^{1}H NMR (400 MHz, $CDCl_3$) δ 8.10 (d, 2H, *J*=8.9 Hz), 7.72 (d, 1H, *J*=8.2 Hz), 7.44-7.42 (m, 3H), 7.15 (dd, 1H, *J*=7.4 Hz, *J*=7.6 Hz), 6.96 (d, 1H, *J*=7.4 Hz), 6.87 (s, 2H), 5.19 (s, 1H), 4.95 (d, 1H, *J*=11.3 Hz), 1.49 (s, 9H), 1.31 (s, 18H) ^{13}C{^{1}H} NMR (100 MHz, $CDCl_3$). δ 170.5 (d, *J*=21.8 Hz), 153.5, 148.1, 147.0, 144.5 (d, *J*=5.3 Hz), 140.7 (d, *J*=5.3 Hz), 135.7, 131.9 (d, *J*=3.0 Hz), 130.8 (d, *J*=1.5 Hz), 126.6 (d, *J*=1.7 Hz), 125.8, 124.8 (d, *J*=2.0 Hz), 124.6 (d, *J*=2.7 Hz), 124.0 (d, *J*=19.4 Hz), 123.3, 115.3, 93.6 (d, *J*=196.4 Hz), 85.0, 60.4, 57.3 (d, *J*=27.4 Hz), 34.3, 30.1, 27.9. ^{19}F NMR (376 MHz, $CDCl_3$) δ -145.7 (d, *J*=11.1 Hz) HRMS (ESI-TOF) m/z: $[M+Na]^+$ calcd for $C_{34}H_{39}FN_2O_6Na$ 613.2684, found 613.2685.

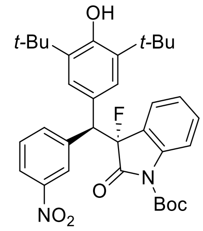

Compound **9** was obtained as a white crystalline solid in 90% isolated yield (31.6 mg, 0.05 mmol) from *N*-Boc-3-fluoro-2-oxindole (15.0 mg, 0.06 mmol) and 2,6-di-*tert*-butyl-4-(3-nitrobenzylidene)cyclohexa-2,5-dien-1-one (20.3 mg, 0.06 mmol) following the general procedure described after purification by flash chromatography using hexanes:ethyl acetate (95:5) as the mobile phase. The *dr* was determined as 50:1 using ^{1}H NMR spectroscopy. The *ee* was determined by chiral HPLC (Amylose-1, hexanes/IPA, 98:2, flow rate 1 mL/min, λ=254 nm) as 81%, t_R (major)= 6.7 min, t_R (minor)= 13.9 min. ^{1}H NMR (400 MHz, $CDCl_3$) δ 8.10 (m, 1H), 8.01 (dd, 1H, *J*=2.1 Hz, *J*=2.1 Hz), 7.70 (d, 1H, *J*=7.9 Hz), 7.41-7.46 (m, 2H), 7.16 (dd, 1H, *J*=7.6 Hz, *J*=7.6 Hz), 6.96 (d, 1H, *J*=7.5 Hz), 6.90 (s, 2H), 5.18 (s, 1H), 4.94 (d, 1H, *J*=11.3 Hz), 1.48 (s, 9H), 1.31 (s, 18H). $^{13}C\{^{1}H\}$ NMR (100 MHz, $CDCl_3$) δ 170.6 (d, *J*=21.8 Hz), 153.5, 148.0, 140.6 (d, *J*= 5.4 Hz), 139.0 (d, *J*=5.6 Hz), 136.1 (d, *J*=1.7 Hz), 135.7, 131.9 (d, *J*= 2.9 Hz), 129.2, 126.6 (d, *J*=1.6 Hz), 125.8, 124.8, 124.8 (d, *J*=1.4 Hz), 124.6 (d, *J*=2.6 Hz), 123.9 (d, *J*=19.4 Hz), 122.3, 115.3, 93.5 (d, *J*=196.5 Hz), 94.5, 92.6, 84.9, 60.3, 57.1 (d, *J*=27.5 Hz), 34.3, 30.1, 27.9. ^{19}F NMR (376 MHz, $CDCl_3$) δ -146.3 (d, *J*=10.9). HRMS (ESI-TOF) m/z: $[M+Na]^+$ calcd for $C_{34}H_{39}FN_2O_6Na$ 613.2684, found 613.2684. Melting point range = 71-74 °C.

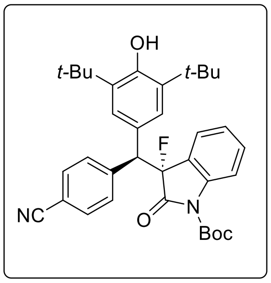

Compound **10** was obtained as a yellow amorphous solid in 84% isolated yield (27.0 mg, 0.05 mmol) from *N*-Boc-3-fluoro-2-oxindole (15.0 mg, 0.06 mmol) and 4-((3,5-di-tert-butyl-4-oxocyclohexa-2,5-dien-1-ylidene)methyl)benzonitrile (19.1 mg, 0.06 mmol) following the general procedure described after purification by flash chromatography using hexanes:ethyl acetate (95:5) as the mobile phase. The *dr* was determined as 20:1 using ^{1}H NMR spectroscopy. The *ee* was determined by chiral HPLC (Chiralpak-IA, hexanes/IPA, 99:1, flow rate 1 mL/min, λ=254 nm) as 91%, t_R (major)=8.0 min, t_R (minor)=14.1 min. ^{1}H NMR (400 MHz, $CDCl_3$) δ 7.72 (d, 1H, *J*=8.2 Hz), 7.53 (d, 2H, *J*=8.3 Hz), 7.42 (dd, 1H, *J*=6.7 Hz, *J*=9.0 Hz), 7.33 (d, 2H, *J*=7.9 Hz), 7.13 (dd, 1H, *J*=7.5 Hz, *J*=7.6 Hz), 6.87-6.91 (m, 3H), 5.18 (s, 1H), 4.89 (d, 1H, *J*=11.7 Hz), 1.50 (s, 9H), 1.30 (s, 18H) $^{13}C\{^{1}H\}$ NMR (100 MHz, $CDCl_3$) δ 170.5 (d, *J*=21.8 Hz), 153.4, 148.1, 142.4 (d, *J*=5.6 Hz), 140.7 (d, *J*=5.4 Hz), 135.7, 132.0, 131.8 (d, *J*=3.0 Hz), 130.6 (d, *J*=1.4 Hz), 126.6 (d, *J*=1.8 Hz), 125.9, 124.9 (d, *J*=1.6 Hz), 124.5 (d, *J*=2.7 Hz), 124.0 (d, *J*=19.4 Hz), 118.6, 115.2, 111.2, 93.6 (d, *J*=196.3 Hz), 84.9, 57.4 (d, *J*=27.4 Hz), 34.3, 30.1, 27.9. ^{19}F NMR (376 MHz, $CDCl_3$) δ -145.8 (d, *J*=11.5 Hz) HRMS (ESI-TOF) m/z: $[M+Na]^+$ calcd for $C_{35}H_{39}FN_2O_4Na$ 593.2786, found 593.2786.

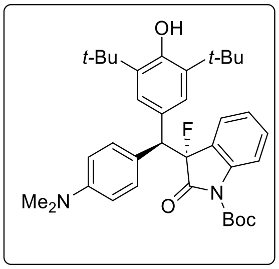

Compound **11** was obtained as a white crystalline solid in 98% isolated yield (33.0 mg, 0.06 mmol) from *N*-Boc-3-fluoro-2-oxindole (15.0 mg, 0.06 mmol) and 2,6-di-*tert*-butyl-4-(4-(dimethylamino)benzylidene)cyclohexa-2,5-dien-1-one (20.1 mg, 0.06 mmol) following the general procedure described after purification by flash chromatography using hexanes:ethyl acetate (95:5) as the mobile phase. The *dr* was determined as 35:1 using ^{19}F NMR spectroscopy. The *ee* was determined by chiral HPLC ((*S,S*)-Whelk-O 1, hexanes/IPA, 98:2, flow rate 1 mL/min, λ=254 nm) as 90%, t_R (major)= 13.6 min, t_R (minor)= 18.0 min. ^{1}H NMR (400 MHz, $CDCl_3$) δ 7.75 (d, 1H, *J*=8.2 Hz), 7.39 (dd, 1H, *J*=8.0 Hz, *J*=7.9), 7.15 (s, 2H), 7.03 (dd, 1H, *J*=7.6 Hz, *J*=7.6 Hz), 6.75 (d, 2H, *J*=8.4 Hz), 6.56 (d, 1H, *J*=7.5 Hz), 6.50 (d, 2H, *J*=8.6 Hz), 5.15 (s, 1H), 4.75 (d, 1H, *J*= 15.6 Hz), 2.84 (s, 6H), 1.44 (s, 9H), 1.37 (s, 18H) ^{13}C{^{1}H} NMR (100 MHz, $CDCl_3$) δ 171.5 (d, *J*=21.7 Hz), 153.0, 149.7, 148.4, 141.1 (d, *J*=5.5 Hz), 135.0, 131.2 (d, *J*=2.8 Hz), 130.4, 127.2, 127.0 (d, *J*=2.1 Hz), 126.5, 124.1 (d, *J*=19.8 Hz), 123.9 (d, *J*=2.6 Hz), 123.1 (d, *J*=8.8 Hz), 115.0, 112.3, 94.2 (d, *J*=195.1 Hz), 84.0, 56.0 (d, *J*=26.4 Hz), 40.4, 34.4, 30.3, 27.8. ^{19}F NMR (376 MHz, $CDCl_3$) δ -145.6 (d, *J*=15.5 Hz). HRMS (ESI-TOF) m/z: $[M+Na]^+$ calcd for $C_{36}H_{45}FN_2O_4Na$ 611.3256, found 611.3254. Melting point range= 72-74 ºC.

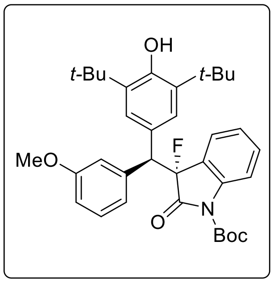

Compound **12** was obtained as a yellow amorphous solid in 85% isolated yield (29.0 mg, 0.05 mmol) from *N*-Boc-3-fluoro-2-oxindole (15.0 mg, 0.06 mmol) and 2,6-di-*tert*-butyl-4-(3-methoxybenzylidene)cyclohexa-2,5-dien-1-one (19.4 mg, 0.06 mmol) following the general procedure described above after purification by flash chromatography using hexanes:ethyl acetate (95:5) as the mobile phase. The *dr* was determined as 24:1 using ^{1}H NMR spectroscopy. The *ee* was determined by chiral HPLC (Chiralpak AD-H, hexanes/IPA 98:2, flow rate 1 mL/min, λ=254 nm) as 89%, t_R (major)= 4.5 min, t_R (minor)=6.5 min. ^{1}H NMR (400 MHz, $CDCl_3$) δ 7.77 (d, 1H, *J*=8.3 Hz), 7.42 (dd, 1H, *J*=7.7 Hz, *J*=7.6 Hz), 7.13 (s, 2H), 7.04-7.10 (m, 2H), 6.73 (d, 1H, *J*=8.3 Hz), 6.62 (d, 1H, *J*=7.6 Hz), 6.58 (d, 1H, *J*=7.7 Hz), 6.49 (s, 1H), 5.19 (s, 1H), 4.84 (d, 1H, *J*=15.3 Hz), 3.57 (s, 3H), 1.47 (s, 9H), 1.37 (s, 18H). $^{13}C\{^{1}H\}$ NMR (100 MHz, $CDCl_3$) δ 171.0 (d, *J*=21.6 Hz), 159.3, 153.3, 148.3, 141.1 (d, *J*=5.6 Hz), 137.4 (d, *J*=8.4 Hz), 135.2, 131.4 (d, *J*=2.8 Hz), 129.3, 127.0 (d, *J*=1.9 Hz), 126.5, 126.2, 124.1 (d, *J*=2.6 Hz), 124.0 (d, *J*=19.5 Hz), 122.2, 115.1, 114.3, 113.9, 93.9 (d, *J*=193.9 Hz), 84.4, 56.9 (d, *J*=27.1 Hz), 54.8, 34.4, 30.2, 27.8. ^{19}F NMR (376 MHz, $CDCl_3$) δ -145.4 (d, *J*= 15.1 Hz). HRMS (ESI-TOF) m/z: $[M+Na]^+$ calcd for $C_{35}H_{42}FNO_5Na$, 598.2939, found 598.2936.

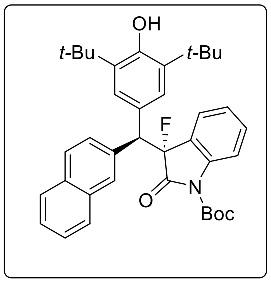

Compound **13** was obtained as a yellow amorphous solid in 98% isolated yield (35.0 mg, 0.06 mmol) from *N*-Boc-3-fluoro-2-oxindole (15.0 mg, 0.06 mmol) and 2,6-di-*tert*-butyl-4-(naphthalen-2-ylmethylene)cyclohexa-2,5-dien-1-one (20.6 mg, 0.06 mmol) following the general procedure described after purification by flash chromatography using hexanes:ethyl acetate (95:5) as the mobile phase. The *dr* was determined as 53:1 using ^{1}H NMR spectroscopy. The *ee* was determined by chiral HPLC ((*S,S*)-Whelk-O 1, hexanes/IPA, 98.5:5, flow rate 1 mL/min, λ=254 nm) as 93%, t_R (major)= 11.3 min, t_R (minor)= 21.9 min. ^{1}H NMR (400 MHz, $CDCl_3$) δ 7.73-7.79 (m, 2H), 7.65-7.66 (m, 2H), 7.44-7.50 (m, 2H), 7.40-7.42 (m, 2H), 7.26 (s, 1H), 7.11 (dd, 1H, *J*=7.6 Hz, *J*=7.6 Hz), 7.01 (d, 1H, *J*=8.6 Hz), 6.71 (d, 1H, *J*=7.5 Hz), 5.20 (s, 1H), 5.05 (d, 1H, *J*=14.9 Hz), 1.37 (s, 18H), 1.25 (s, 9H) ^{13}C{^{1}H} NMR (100 MHz, CDCl3) δ 171.1 (d, *J*=21.4 Hz), 153.2, 148.1, 141.2 (d, *J*=5.6 Hz), 135.3, 133.5 (d, *J*=8.2 Hz), 133.2, 132.6, 131.5 (d, *J*=2.8 Hz), 129.1, 128.0, 127.7, 127.5, 127.3, 127.0 (d, *J*=2.2 Hz), 126.5, 126.3, 125.9 (d, *J*=6.4 Hz), 124.1 (d, *J*=2.1 Hz), 124.1 (d, *J*=14.9 Hz), 115.1, 94.2 (d, *J*=194.9 Hz), 84.2, 57.1 (d, *J*=26.9 Hz), 34.4, 30.2, 27.5. ^{19}F NMR (376 MHz, $CDCl_3$) δ -145.9 (d, *J*=14.9 Hz). HRMS (ESI-TOF) m/z: $[M+Na]^+$ calcd for $C_{38}H_{42}FNO_4Na$ 618.2990, found 618.2989.

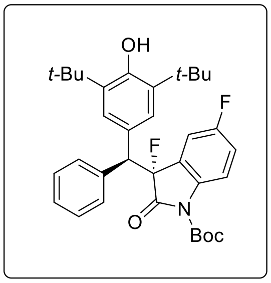

Compound **14** was obtained as a yellow amorphous solid in 84% isolated yield (30.5 mg, 0.05 mmol) from *N*-Boc-3-fluoro-5-fluoro-2-oxindole (16.1 mg, 0.06 mmol) and 4-benzylidene-2,6-di-*tert*-butylcyclohexa-2,5-dien-1-one (17.6 mg, 0.06 mmol)following the general procedure described after purification by flash chromatography using hexanes:ethyl acetate (98:2) as the mobile phase. The *dr* was determined as 13:1 using ^{19}F NMR spectroscopy. The *ee* was determined by chiral HPLC ((*S,S*)-Whelk-O 1, hexanes/IPA, 99.5:0.5, flow rate 1 mL/min, λ=254 nm) as 85%, t_R (major)= 10.5 min, t_R (minor)= 13.6 min. ^{1}H NMR (400 MHz, $CDCl_3$) 7.76 (m, 1H), 7.15-7.19 (m, 3H), 7.08-7.15 (m, 3H), 6.89-6.95 (m, 2H), 6.29 (m, 1H), 5.21 (s, 1H), 4.85 (d, 1H, *J*=15.9 Hz), 1.44 (s, 9H), 1.37 (s, 18H) ^{13}C{^{1}H} NMR (100 MHz, $CDCl_3$) δ 170.6 (d, *J*=21.6 Hz), 160.5 (dd, *J*= 3.2 Hz, *J*= 244.8 Hz), 158.0 (d, *J*=3.2 Hz), 153.4, 148.2, 137.0, 135.7 (d, *J*=8.3 Hz), 135.4, 129.9, 128.4, 127.6, 126.9 (d, *J*=2.0 Hz), 125.9, 125.6 (d, *J*=8.4 Hz), 125.4 (d, *J*=8.3 Hz), 118.1, 117.8, 116.6 (d, *J*=7.7 Hz), 114.1 (d, *J*=25.4 Hz), 93.5 (d, *J*=194.4 Hz), 84.6, 84.4, 56.8 (d, *J*=26.5 Hz), 34.4, 30.2, 27.8. ^{19}F NMR (376 MHz, $CDCl_3$) δ -117.2, -145.2 (d, *J*=15.8 Hz). HRMS (ESI-TOF) m/z: $[M+Na]^+$ calcd for $C_{34}H_{39}F_2NO_4Na$ 586.2739, found 586.2737.

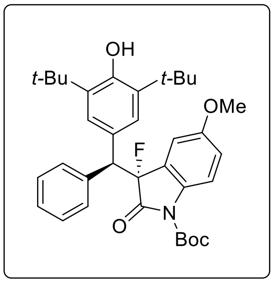

Compound **15** was obtained as a yellow amorphous solid in 99% isolated yield (30.0 mg, 0.05 mmol) from *N*-Boc-3-fluoro-5-methoxy-2-oxindole (16.9 mg, 0.06 mmol) and 4-benzylidene-2,6-di-*tert*-butylcyclohexa-2,5-dien-1-one (17.6 mg, 0.06 mmol) following the general procedure described above after purification by flash chromatography using hexanes:ethyl acetate (95:5) as the mobile phase. The *dr* was determined as 34:1 using ^{1}H NMR spectroscopy. The *ee* was determined by chiral HPLC (Chiralpak IA, hexanes/IPA, 98:2, flow rate 1 mL/min, λ=254 nm) as 60%, t_R (major)= 5.2 min, t_R (minor)= 6.3 min. ^{1}H NMR (400 MHz, $CDCl_3$) δ 7.66 (d, 1H, *J*=8.8 Hz), 7.15-7.18 (m, 3H), 7.13 (s, 2H), 6.93-6.95 (m, 3H), 6.11 (s, 1H), 5.18 (s, 1H), 4.88 (d, 1H, *J*=16.3 Hz), 3.61 (s, 3H), 1.44 (s, 9H), 1.36 (s, 18H) $^{13}C\{^{1}H\}$ NMR (100 MHz, $CDCl_3$) δ 171.0 (d, *J*=21.6 Hz), 156.4 (d, *J*=2.9 Hz), 153.2, 148.4, 135.7 (d, *J*=8.6 Hz), 135.3, 134.2 (d, *J*=5.6 Hz), 129.8, 128.3, 127.5, 126.9 (d, *J*=2.4 Hz), 126.5, 124.9 (d, *J*=19.3 Hz), 116.9 (d, *J*=2.8 Hz), 116.1, 111.8, 94.2 (d, *J*=193.9 Hz), 84.1, 56.6 (d, *J*=26.4 Hz), 55.4, 34.4, 30.2, 27.9. ^{19}F NMR (376 MHz, $CDCl_3$) δ -144.3 (d, *J*=16.3 Hz) HRMS (ESI-TOF) m/z: $[M+Na]^+$ calcd for $C_{35}H_{42}FNO_5Na$, 598.2939, found 598.2939.

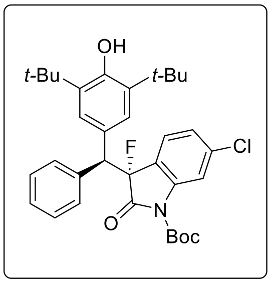

Compound **16** was obtained as a yellow amorphous solid in 99% isolated yield (34.0 mg, 0.06 mmol) from *N*-Boc-3-fluoro-6-chloro-2-oxindole (17.1 mg, 0.06 mmol) and 4-benzylidene-2,6-di-*tert*-butylcyclohexa-2,5-dien-1-one (17.7 mg, 0.06 mmol) following the general procedure described above after purification by flash chromatography using hexanes:ethyl acetate (95:5) as the mobile phase. The *dr* was determined as 24:1 using ^{1}H NMR spectroscopy. The *ee* was determined by chiral HPLC ((*S,S*)-Whelk-O 1, hexanes/IPA, 99:1, flow rate 0.65 mL/min, λ=254 nm) as 71%, t_R (major)= 12.2 min, t_R (minor)= 13.6 min. ^{1}H NMR (400 MHz, $CDCl_3$) δ 7.85 (m, 1H), 7.17-7.21 (m, 3H), 7.10 (s, 2H), 7.04 (dd, 1H, *J*=8.0 Hz, *J*=0.8 Hz), 6.94-6.97 (m, 2H), 6.52 (dd, 1H, *J*=8.1 Hz, *J*=1.8 Hz), 5.20 (s, 1H), 4.84 (d, 1H, *J*=15.6 Hz), 1.45 (s, 9H), 1.36 (s, 18H) $^{13}C\{^{1}H\}$ NMR (100 MHz, $CDCl_3$). δ 170.4 (d, *J*=21.4 Hz), 153.3, 148.1, 141.9 (d, *J*=5.5 Hz), 137.4 (d, *J*=3.4 Hz), 135.8 (d, *J*=4.9 Hz), 135.7, 135.4, 130.2 (d, *J*=2.8 Hz), 129.6, 128.1, 127.3, 126.9 (d, *J*=2.1 Hz), 126.6, 126.1, 124.2 (d, *J*=2.6 Hz), 122.4 (d, *J*=19.9 Hz), 116.0, 93.5 (d, *J*=194.3 Hz), 84.9, 56.8 (d, *J*=26.7 Hz), 34.4, 30.2, 27.8. ^{19}F NMR (376 MHz, $CDCl_3$) δ -144.5 (d, *J*=15.6 Hz). HRMS (ESI-TOF) m/z: $[M+Na]^+$ calcd for $C_{34}H_{39}FClNO_4Na$, 602.2444, found 602.2443.

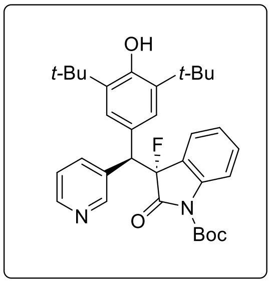

Compound **17** was obtained as a yellow amorphous solid in 98% isolated yield (31.9 mg, 0.06 mmol) from *N*-Boc-3-fluoro-2-oxindole (15.0 mg, 0.06 mmol) and 2,6-di-*tert*-butyl-4-(pyridin-3-ylmethylene)cyclohexa-2,5-dien-1-one (17.6 mg, 0.06 mmol) following the general procedure described above after purification by flash chromatography using hexanes:ethyl acetate (95:5) as

the mobile phase. The *dr* was determined as 19:1 using ^{1}H NMR spectroscopy. The *ee* was determined by chiral HPLC (Chiralpak-IB, hexanes/IPA, 98:2, flow rate 1.0 mL/min, λ=254 nm) as 64%, t_R (major)= 9.10 min, t_R (minor)= 10.5 min. ^{1}H NMR (400 MHz, $CDCl_3$) 8.46 (d, 1H, *J*=4.0 Hz), 8.34 (s, 1H), 7.75 (d, 1H, *J*=8.3 Hz), 7.54 (d, 1H, *J*=7.7 Hz), 7.42 (dd, 1H, *J*=7.8 Hz, *J*=8.0 Hz), 7.10-7.18 (m, 2H), 6.96 (s, 2H), 6.88 (d, 1H, *J*=12.1 Hz), 5.20 (s, 1H), 4.86 (d, 1H, *J*= 12.2 Hz), 1.49 (s, 9H), 1.33 (s, 18H). $^{13}C\{^{1}H\}$ NMR (100 MHz, $CDCl_3$) δ 170.7 (d, *J*=21.7 Hz), 153.4, 151.0, 148.7, 148.1, 140.7 (d, *J*=5.3 Hz), 137.0, 135.6, 132.5 (d, *J*=6.3 Hz), 131.8 (d, *J*=2.8 Hz), 126.7 (d, *J*=1.8 Hz), 126.0, 125.1, 124.4 (d, *J*=2.7 Hz), 123.8 (d, *J*=19.5 Hz), 123.0, 115.3, 93.5 (d, *J*=195.7 Hz), 84.8, 55.1 (d, *J*=27.6 Hz), 34.3, 30.1, 27.9. ^{19}F NMR (376 MHz, $CDCl_3$) δ -145.9 (d, *J*=12.0 Hz). HRMS (ESI-TOF) m/z: $[M+Na]^+$ calcd for $C_{33}H_{39}FN_2O_4Na$ 569.2786, found 569.2784.

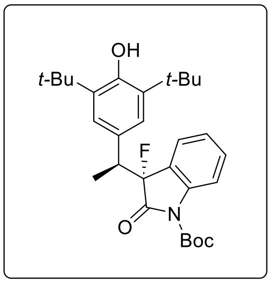

Compound **18** was obtained as a yellow amorphous solid in 94% isolated yield (27.2 mg, 0.06 mmol) from *N*-Boc-3-fluoro-2-oxindole (15.0 mg, 0.06 mmol) and 2,6-di-*tert*-butyl-4-ethylidenecyclohexa-2,5-dien-1-one (13.9 mg, 0.06 mmol) following the general procedure described above after purification by flash chromatography using hexanes:ethyl acetate (95:5) as the mobile phase. The *dr* was determined as 3:1 using ^{1}H NMR spectroscopy. The *ee* was determined by chiral HPLC (Chiralpak-IC, hexanes/IPA, 98:2, flow rate 0.75 mL/min, λ=254 nm) as 31%, t_R (major)= 6.6 min, t_R (minor)= 6.3 min. ^{1}H NMR (3:1 mixture of diastereomers) (400 MHz, $CDCl_3$) 7.66 (d, 3H, *J*=8.2 Hz), 7.59 (d, 1H, *J*=8.2 Hz), 7.52 (d, 1H, *J*=7.5 Hz), 7.32

(m, 1H), 7.28-7.30 (m, 3H), 7.22 (m, 1H), 7.08 (m, 3H), 6.94 (d, 3H, *J*=7.5 Hz), 6.80 (s, 6H), 6.53 (s, 2H), 5.08 (s, 3H), 5.02 (s, 1H), 3.56-3.64 (m, 4H), 1.59 (s, 27H), 1.48 (s, 9H), 1.41 (d, 3H, *J*=4.8 Hz), 1.39 (d, 9H, *J*=7.2 Hz), 1.32 (s, 54H), 1.22 (s, 18H). $^{13}C\{^1H\}$ NMR (3:1 mixture of diastereomers) (100 MHz, $CDCl_3$) δ 171.6 (d, *J*= 19.5 Hz), 171.4 (d, *J*= 22.0 Hz), 153.0, 152.9, 148.5, 140.4, 140.3 (d, *J*= 5.4 Hz), 135.3, 135.1, 130.9 (d, *J*= 3.0 Hz), 127.2 (d, *J*= 4.2 Hz), 126.9 (d, *J*= 3.0 Hz), 125.8, 125.5, 125.3, 125.2, 124.3 (d, *J*=19.5 Hz), 124.1 (d, *J*= 9.0 Hz), 124.0 (d, *J*=2.5 Hz), 123.8, 115.0, 114.8, 94.5 (d, *J*=194.9 Hz), 93.9 (d, *J*= 191.2 Hz), 84.6, 84.2, 45.7 (d, *J*= 15.7 Hz), 45.5 (d, *J*= 25.8 Hz), 34.2, 34.1, 30.2, 30.0, 29.7, 29.6, 28.1, 28.0, 13.8 (d, *J*= 4.3 Hz), 13.0 (d, *J*= 5.0 Hz). ^{19}F NMR (3:1 mixture of diastereomers) (376 MHz, $CDCl_3$) -153.2 (d, *J*= 9.6 Hz), -158.8 (d, *J*= 9.9 Hz). HRMS (ESI-TOF) m/z: $[M+Na]^+$ calcd for $C_{29}H_{38}FNO_4Na$ 506.2677, found 506.2674.

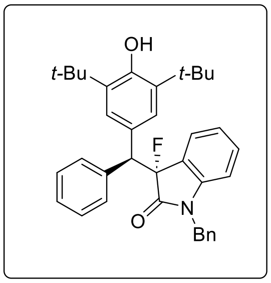

Compound **19** was obtained as a yellow amorphous solid in 98% isolated yield (31.5 mg, 0.06 mmol) from *N*-benzyl-3-fluoro-2-oxindole (14.5 mg, 0.06 mmol) and 4-benzylidene-2,6-di-*tert*-butylcyclohexa-2,5-dien-1-one (17.7 mg, 0.06 mmol) following the general procedure described above after purification by flash chromatography using hexanes:ethyl acetate (95:5) as the mobile phase. The *dr* was determined as 1.3:1 using ^1H NMR spectroscopy. The *ee* was determined by chiral HPLC ((*S,S*)-Whelk-O 1, hexanes/IPA, 98:2, flow rate 1.0 mL/min, λ=254 nm) as 33%, t_R (major)= 14.0 min, t_R (minor)= 12.3 min. ^1H NMR (1.3:1 mixture of diastereomers) (400 MHz, $CDCl_3$) 7.54 (m, 1H), 7.33-7.36 (m, 2H), 7.21-7.23 (m, 10H), 7.10-

7.16 (m, 7H), 6.96-6.98 (m, 2H), 6.94 (m, 2H), 6.82 (d, 1H, *J*= 7.5 Hz), 6.75 (s, 1H), 6.71 (d, 1H, *J*= 7.5 Hz), 6.59-6.61 (m, 2H), 6.50-6.54 (m, 3H), 5.18 (s, 1H,) 5.16 (s, 1H), 4.97-5.10 (m, 4H), 4.17-4.28 (m, 2H) 1.37 (s, 18H), 1.16 (s, 14H). $^{13}C\{^1H\}$ NMR (1.3:1 mixture of diastereomers) (100 MHz, $CDCl_3$) δ 173.0 (d, *J*= 21.0 Hz), 172.8 (d, *J*= 21.1 Hz), 153.4, 153.1, 144.5 (d, *J*= 24.3 Hz), 144.3 (d, *J*= 22.0 Hz), 137.5, 135.3, 135.1, 134.8, 134.7, 131.2 (d, *J*= 8.1 Hz), 131.1 (d, *J*= 9.1 Hz), 130.45, 130.42, 129.9, 128.9, 128.6, 128.5, 128.0, 127.4, 127.34, 127.32, 127.22, 127.20 (d, *J*= 1.9 Hz), 127.1, 126.8, 126.7, 126.4, 126.3, 126.2, 125.4, 125.3 (d, *J*= 2.5 Hz), 125.1 (d, *J*= 2.7 Hz), 122.8 (d, *J*= 3.0 Hz), 122.4 (d, *J*= 2.3 Hz), 109.4 (d, *J*= 22.2 Hz), 109.2 (d, *J*= 20.1 Hz), 95.1, 94.4 (d, *J*= 188.1 Hz), 94.3 (d, *J*= 180.0 Hz), 55.9, 55.6, 44.0 (d, *J*= 15.7 Hz), 43.9 (d, *J*= 15.0 Hz), 34.4, 34.1, 30.3, 30.2, 29.7. ^{19}F NMR (1:1 mixture of diastereomers) (376 MHz, $CDCl_3$) -146.8 (d, *J*=12.7 Hz), -147.0 (d, *J*= 12.5 Hz). HRMS (ESI-TOF) m/z: $[M+Na]^+$ calcd for $C_{36}H_{38}FNO_2Na$ 558.2779, found 558.2776.

3.3. Boc deprotection of compound 4

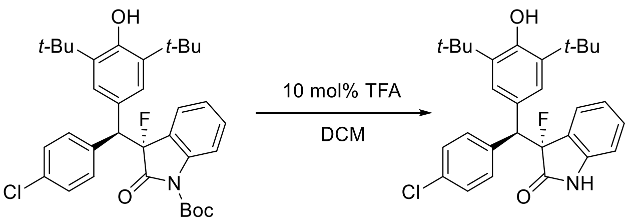

Compound **4** (15.0 mg, 0.026 mmol) and trifluoroacetic acid (10 mol%) were stirred in 200 µL dichloromethane at 25 ºC for 1 hour. Upon completion, the reaction mixture was quenched with water and extracted with dichloromethane. The combined organic layers were dried over sodium sulfate, concentrated and purified via column chromatography using hexanes:ethyl acetate (95:5) as the mobile phase to give compound **21** as a yellow crystalline solid in 98% isolated yield (12.2 mg, 0.025 mmol). The *dr* was determined as 48:1 using ^{1}H NMR spectroscopy. The *ee* was determined by chiral HPLC ((*S,S*)-Whelk-O 1, 95:5 hexanes/IPA, flow rate 1.0 mL/min, λ=254 nm) as 94%, t_R (major)= 6.7 min, t_R (minor)= 9.5 min. ^{1}H NMR (400 MHz, $CDCl_3$) δ 7.33 (dd, 1H, *J*=7.8 Hz, *J*=7.6 Hz), 7.13-7.17 (m, 2H), 7.10 (s, 1H), 6.96 (dd, 1H, *J*=7.5 Hz, *J*=7.7 Hz), 6.90 (2H, d, *J*= 8.1 Hz), 6.77 (d, 1H, *J*=7.8 Hz), 6.60 (1H, d, *J*=7.6 Hz), 5.20 (s, 1H), 4.85 (d, 1H, *J*=15.2 Hz), 1.37 (s, 18H). ^{13}C{^{1}H} NMR (100 MHz, $CDCl_3$) δ 173.6 (d, *J*=20.0 Hz), 153.3, 141.7 (d, *J*=6.0 Hz), 135.3, 135.1 (d, *J*=9.0 Hz), 133.3, 131.5 (d, *J*=3.0 Hz), 130.9, 128.5, 127.3, 126.9 (d, *J*=2.0 Hz), 126.2, 124.9 (d, *J*=19.5 Hz), 122.6 (d, *J*=2.7 Hz), 110.1, 93.8 (d, *J*=192.6 Hz), 55.1 (d, *J*=26.9 Hz), 34.3, 30.2. ^{19}F NMR δ -147.8 (d, *J*=15.5 Hz). (376 MHz, $CDCl_3$) HRMS (ESI-TOF) m/z: $[M+Na]^+$ calcd for $C_{29}H_{31}FClNO_2Na$, 502.1920, found 502.1919. Melting point range = 167-172 ºC.

3.4. Upscaling of the general phase transfer catalysis procedure

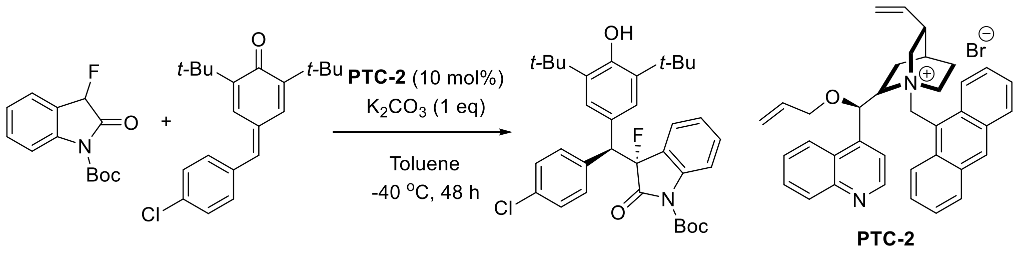

N-Boc-3-fluoro-2-oxindole (382.0 mg, 1.5 mmol), 2,6-di-*tert*-butyl-4-(4-chlorobenzylidene)cyclohexa-2,5-dien-1-one (500.0 mg, 1.5 mmol), potassium carbonate (210.0 mg, 1.5 mmol), and **PTC-2** (10 mol%) were added to an oven-dried vial under nitrogen. Anhydrous toluene (5.0 mL) was added and the resulting mixture was stirred at -40 °C for 48 hours. Upon completion, the reaction mixture was quenched with an aqueous solution of saturated ammonium chloride and extracted with dichloromethane. The combined organic layers were dried over sodium sulfate, concentrated and purified via column chromatography using hexanes:ethyl acetate (95:5) as the mobile phase to afford compound 4 in 93% yield (824 mg, 1.4 mmol). The *dr* was determined as 40:1 using ^{1}H NMR spectroscopy. The *ee* was determined by chiral HPLC (Chiralpak IA, 98:2 hexanes/ IPA, flow rate 1 mL/min, λ=254 nm) as 95%, t_R (major)= 5.1 min, t_R (minor)= 4.5 min.

4. Determination of absolute configuration

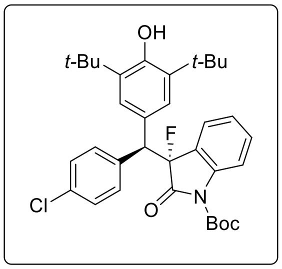

Compound **4**

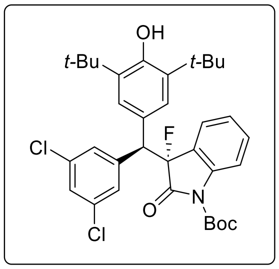

Compound **6**

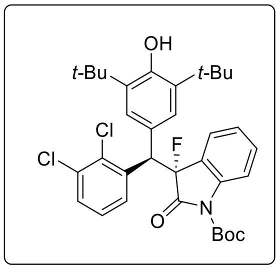

Compound **7**

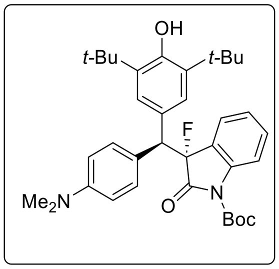

Compound **11**

Single crystal analysis was performed to determine the absolute configuration of the major product formed in the general phase-transfer catalysis procedure. It was found that compounds **4** (91% *ee, dr*=48:1), **6** (85% *ee, dr*=26:1), and **7** (88% *ee, dr*=20:1) did not form single crystals under numerous conditions, including slow evaporation of hexanes/IPA mixtures, pentane and dichloromethane solvent layering, vapor diffusion, and saturation in ether at 0 ºC. Despite this, racemic compound **7** (*dr*=3:1) easily yielded crystals through pentane and dichloromethane solvent layering as described above. To remedy this, compound **11** (90% *ee, dr*=35:1) was used and reacted with 1M HCl in 200 µL acetone followed by slow evaporation of the resulting mixture in IPA to yield the corresponding HCl salt as described above. Polarimetric analysis of the bulk material of the single crystal in acetone showed dextrorotatory rotation, confirming that the crystallographically determined structure is that of the major enantiomer.

5. **Crystallographic data**

Figure S2. X-ray structure (50% ellipsoid probability) of compound **7**, *tert*-butyl-3-(3,5-di-*tert*-butyl-4-hydroxyphenyl)(2,3-dichlorophenyl)methyl)-3-fluoro-2-oxoindoline-1-carboxylate.

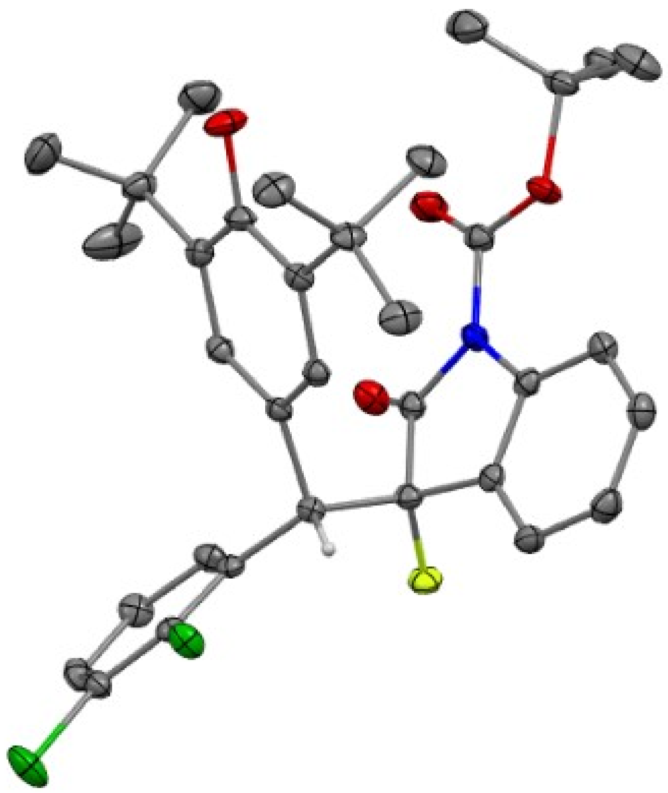

A single crystal was obtained by dissolving the racemic compound (20.0 mg, 0.03 mmol) in dichloromethane (1.0 mL) and layering with pentane (1.0 mL). Single crystal X-ray analysis was performed at 100 K using a Bruker DUO equipped with an APEXII CCD detector and Mo fine-focus sealed source (λ = 0.71073 Å). Data were integrated with the Bruker SAINT program. Structure solution and refinement were performed using the SHELXT/PC suite and ShelXle. Intensities were corrected for Lorentz and polarization effects and an empirical absorption correction was applied using Blessing's method as incorporated into the program SADABS. Non-hydrogen atoms were refined with anisotropic thermal parameters and hydrogen atoms were

included in idealized positions. Select hydrogens omitted for clarity. Crystal data: $C_{34}H_{38}Cl_2FNO_4$, M = 614.55, colorless plate, 0.262 x 0.224 x 0.050 mm^3, monoclinic, space group *P* 2_1/n, a = 9.8083(7), b = 17.5313(13), c = 18.5156(14) Å, V =3108.6(4) $Å^3$, Z = 4. Notable bond lengths: F1-C2: 1.3997 (16) Å. C2-C1: 1.5579 (19) Å. C15-C1: 1.5183 (19) Å. Notable bond angles: C1-C2-F1: 109.68 (11)°. C1-C15-C21: 113.74 (11)°. The CCDC number for this compound is 2312541.

Figure S3. X-ray structure (50% ellipsoid probability) of compound **22**, 4-((*S*)-((*R*)-1-(*tert*-butoxycarbonyl)-3-fluoro-2-oxoindolin-3-yl)(3,5-di-*tert*-butyl-4-hydroxyphenyl)methyl)-N,N-dimethylbenzenaminium chloride.

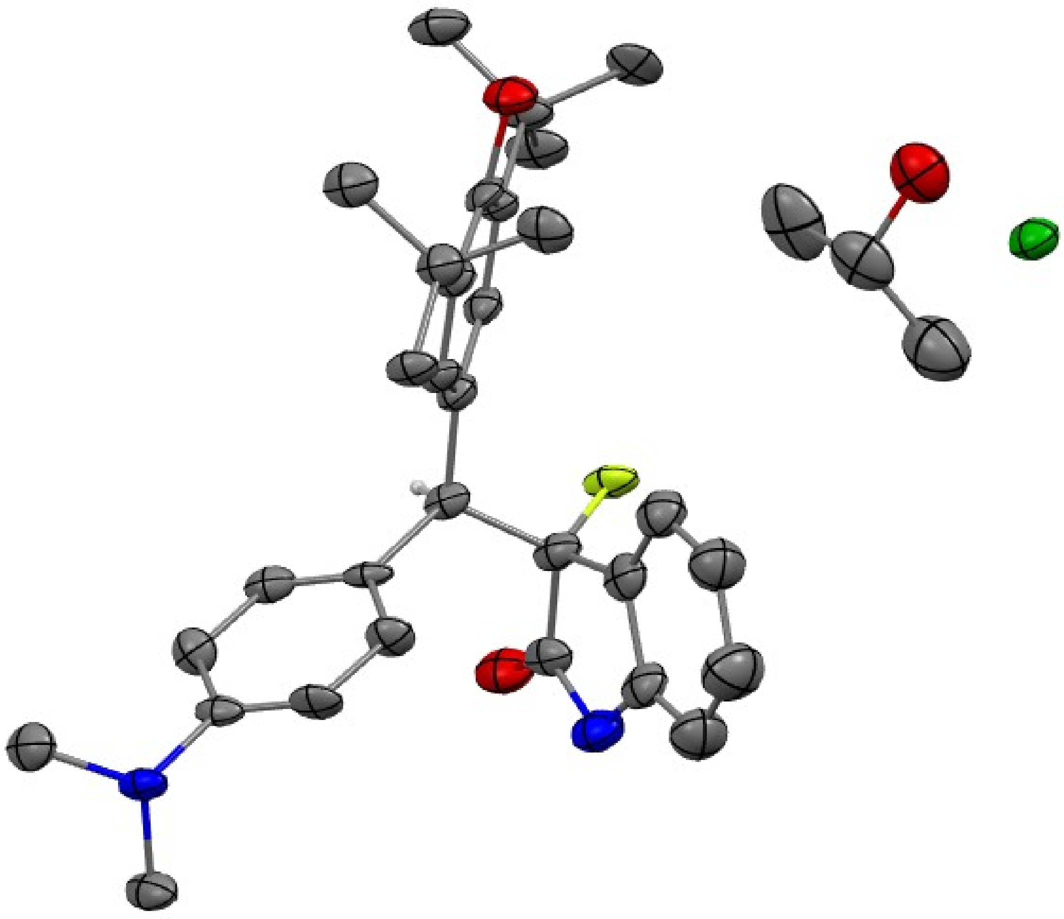

A single crystal was obtained by reacting compound **11** (15.0 mg, 0.03 mmol, 90% *ee, dr*=35:1) with one drop of 1M HCl in 200 μL acetone for 12 hours. After concentrating the reaction mixture, slow evaporation of a solution of the residue in 500 μL isopropanol yielded a single crystal. Single crystal X-ray analysis was performed at 100 K using a Bruker DUO equipped with an APEXII CCD detector and Mo fine-focus sealed source (λ = 0.71073 Å). Data were integrated with the Bruker SAINT program. Structure solution and refinement was performed

using the SHELXT/PC suite and ShelXle. Intensities were corrected for Lorentz and polarization effects and an empirical absorption correction was applied using Blessing's method as incorporated into the program SADABS. Non-hydrogen atoms were refined with anisotropic thermal parameters. The N-H hydrogen atom in the oxindole group was located in the difference map. The N-H distance was restrained to be 0.88 (esd 0.01Å). Remaining hydrogen atoms were included in idealized positions. Select hydrogens omitted for clarity. Crystal data: $C_{31}H_{37}FN_2O_2$, C_3H_8O, Cl. M = 584.17, colorless plate, 0.293 x 0.287 x 0.040 mm^3, orthorhombic, space group $P\, 2_1\, 2_1\, 2_1$, a = 8.683 (4), b = 13.026 (6), c = 30.400 (14) Å, V = 3439 (3) $Å^3$, Z = 4. Notable bond lengths: F1-C2: 1.421 (12) Å. C9-C2: 1.558 (15) Å. C9-C10: 1.520 (14) Å. Notable bond angles: F1-C2-C9: 105.7 (8)°. C3-C2-C1: 110.9 (10)°. Absolute structure parameter = 0.05 (9). The CCDC number for this compound is 2312540.

6. ^{1}H, ^{13}C, and ^{19}F NMR spectra of newly reported compounds

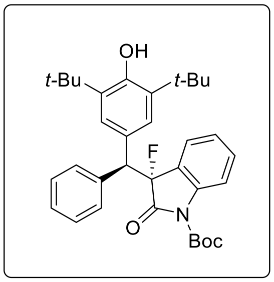

Figure S4. ^{1}H NMR (400 MHz) Spectrum of compound **3** (*dr* = 29:1) in $CDCl_3$.

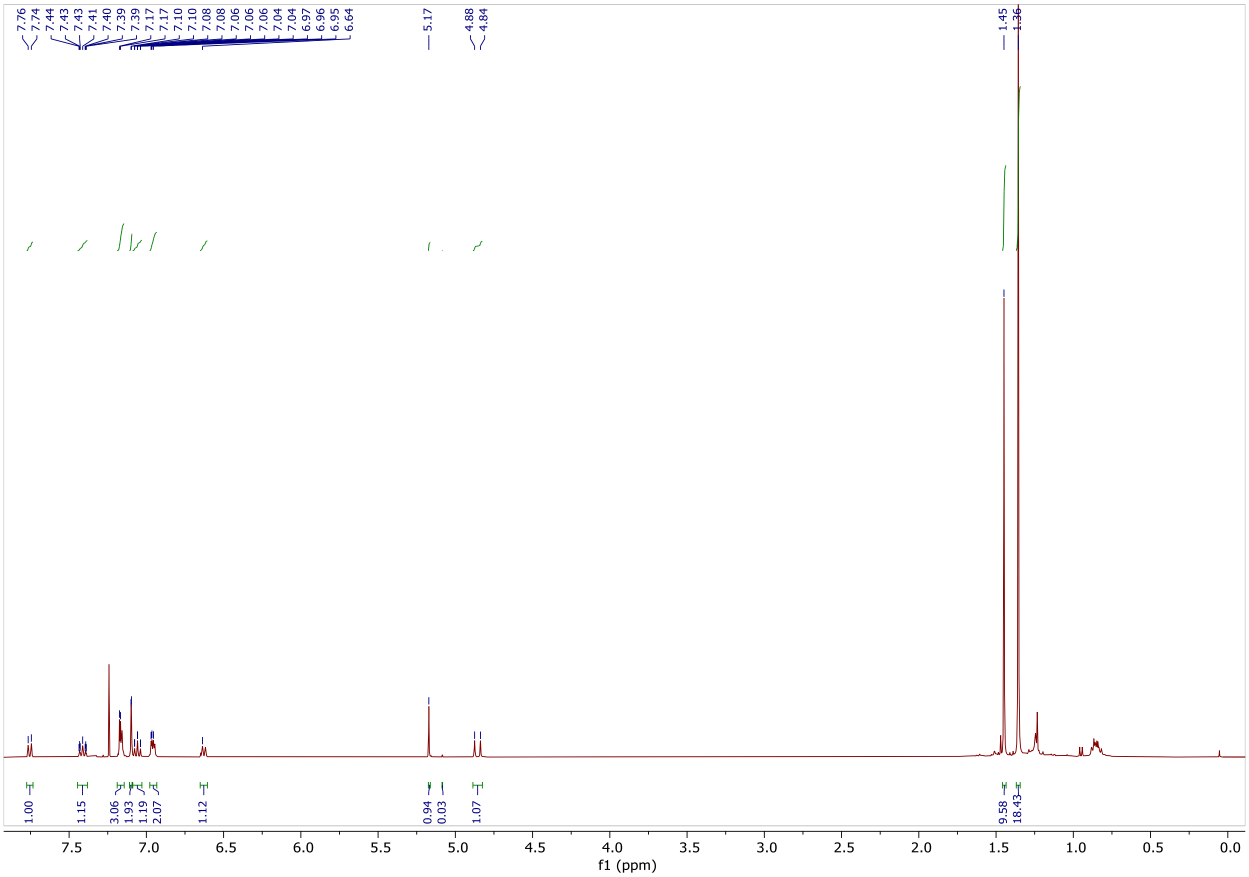

Figure S5. ^{19}F NMR (376 MHz) Spectrum of compound **3** (*dr* = 29:1) in $CDCl_3$.

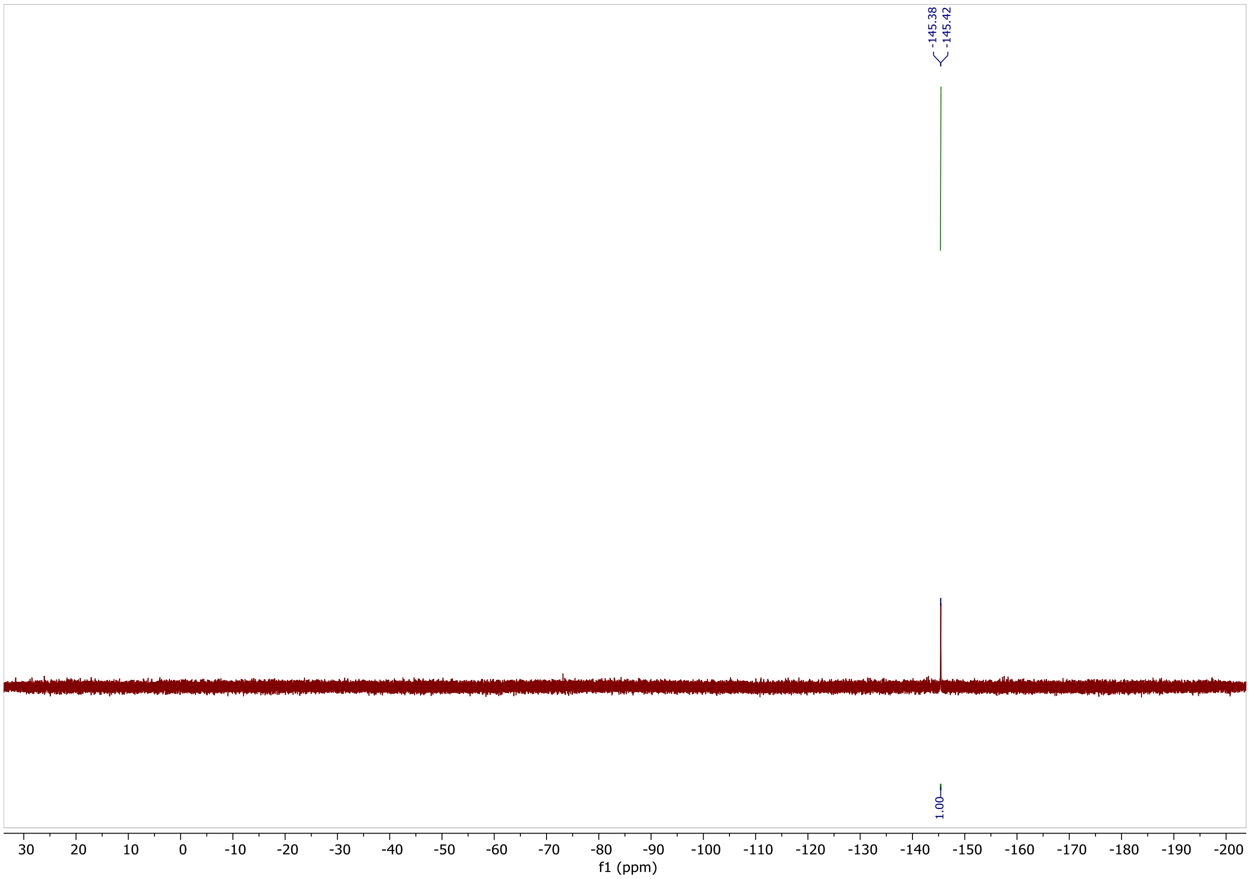

Figure S6. $^{13}C\{1H\}$ NMR (100 MHz) Spectrum of compound **3** (*dr* = 29:1) in $CDCl_3$.

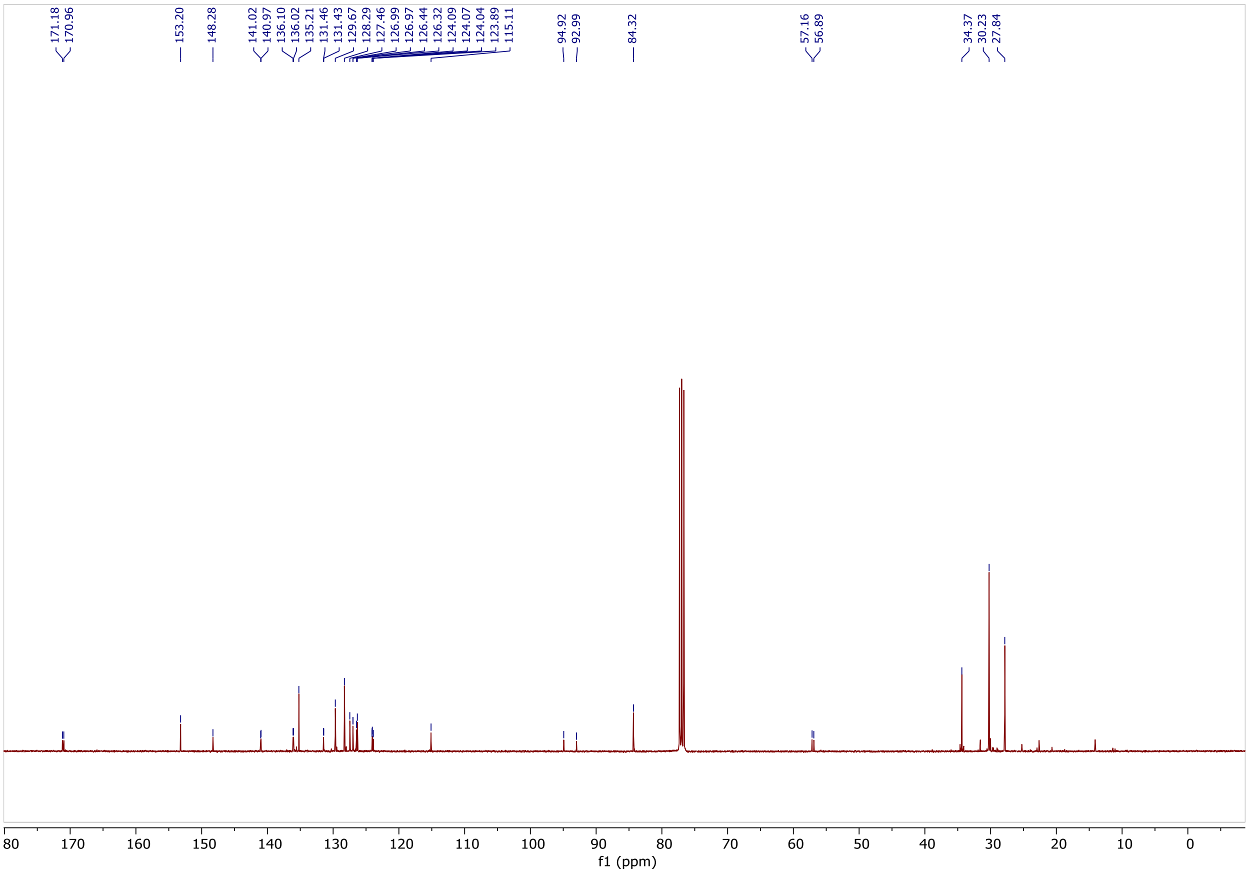

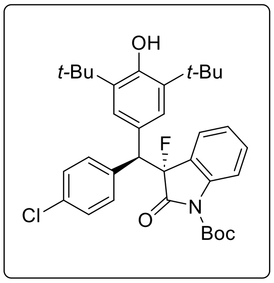

Figure S7. ^{1}H NMR (400 MHz) Spectrum of compound **4** (*dr* = 48:1) in $CDCl_3$.

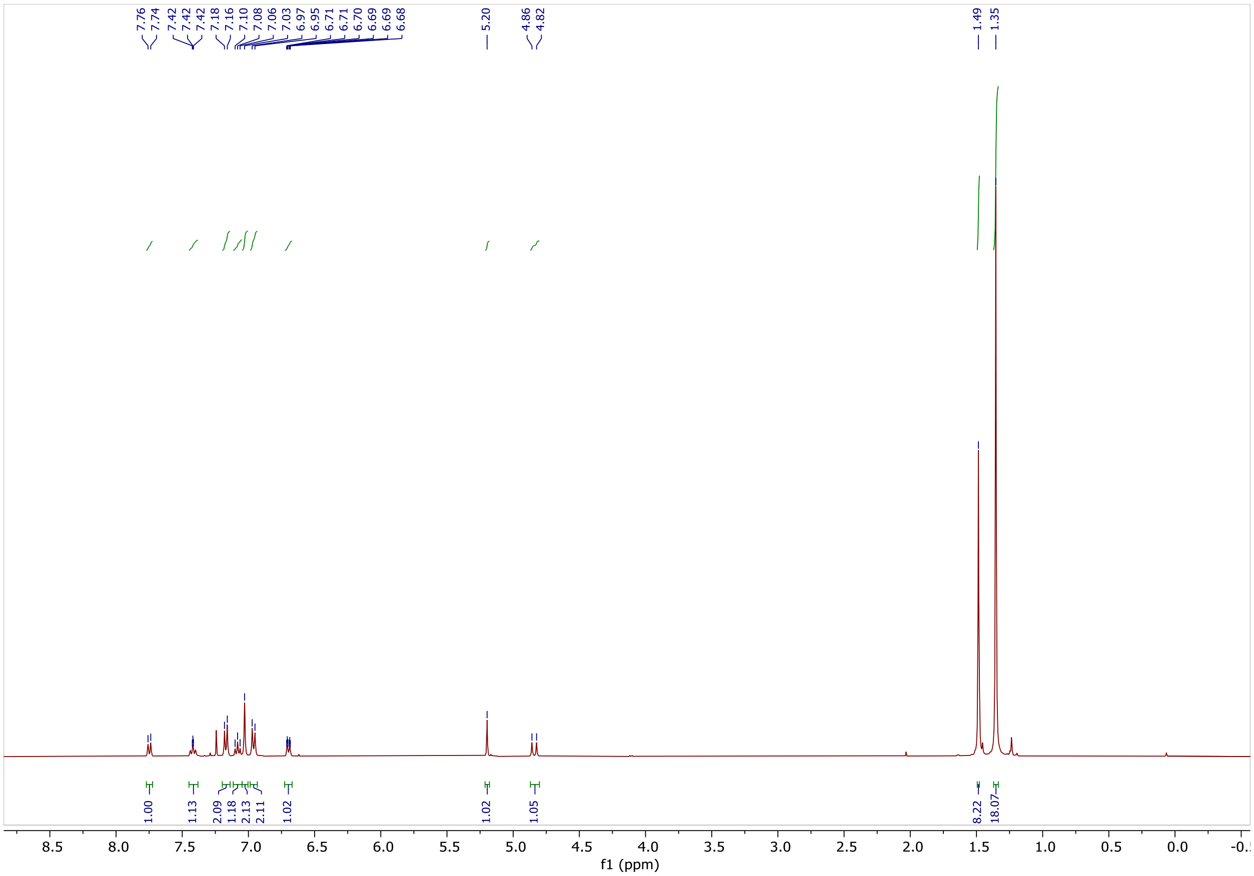

Figure S8. ^{19}F NMR (376 MHz) Spectrum of compound **4** (dr = 48:1) in $CDCl_3$.

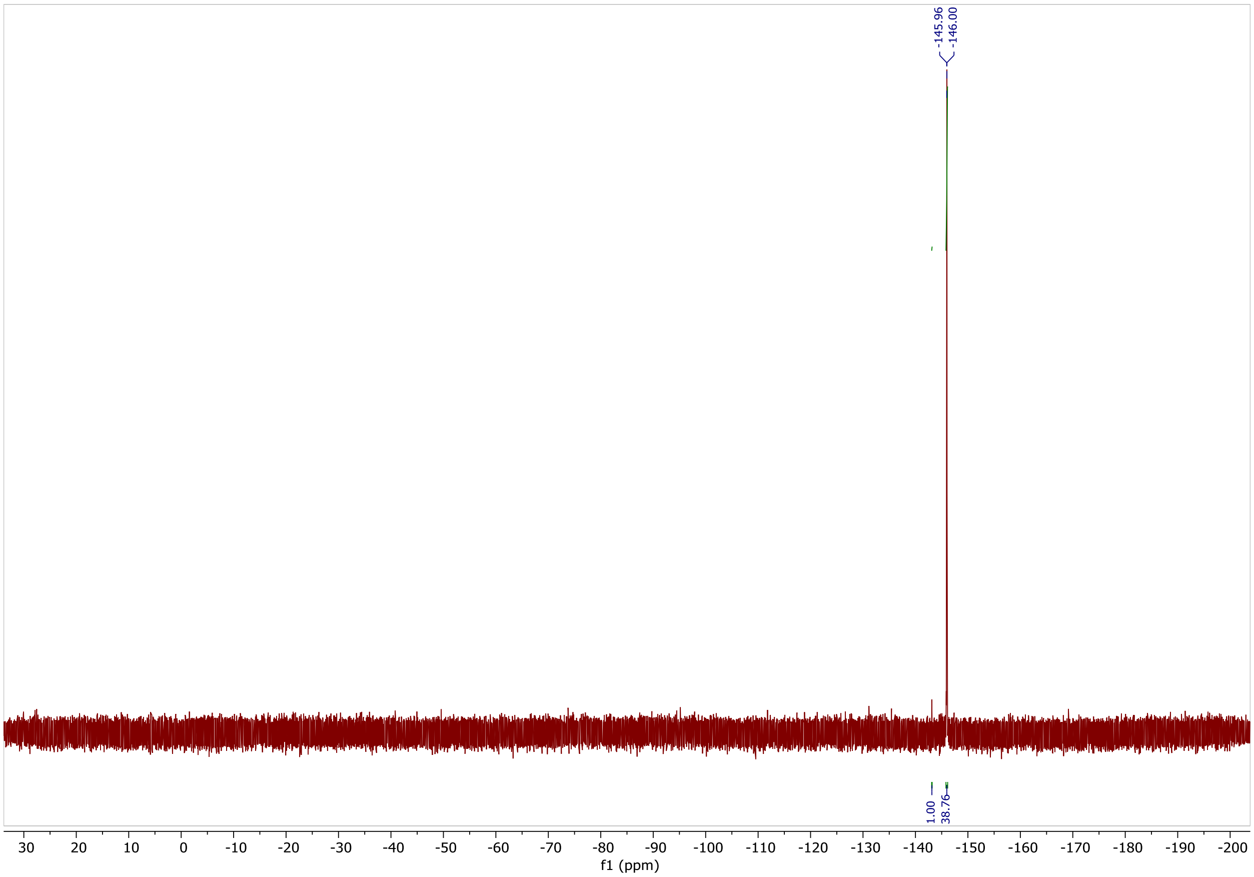

Figure S9. $^{13}C\{1H\}$ NMR (100 MHz) Spectrum of compound **4** (*dr* = 48:1) in $CDCl_3$.

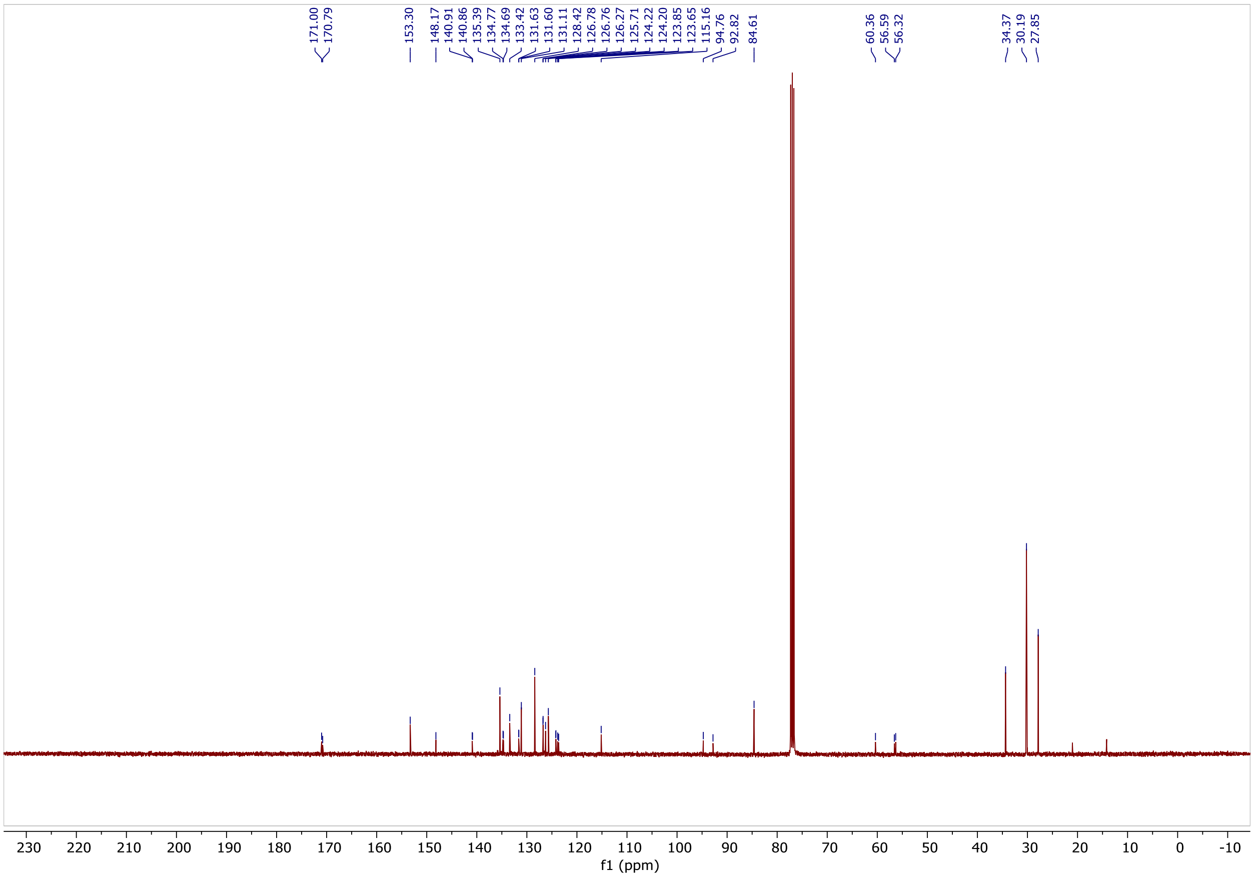

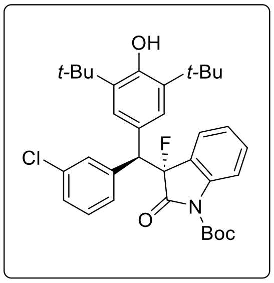

Figure S10. ^{1}H NMR (400 MHz) Spectrum of compound **5** (*dr* = 14:1) in $CDCl_3$.

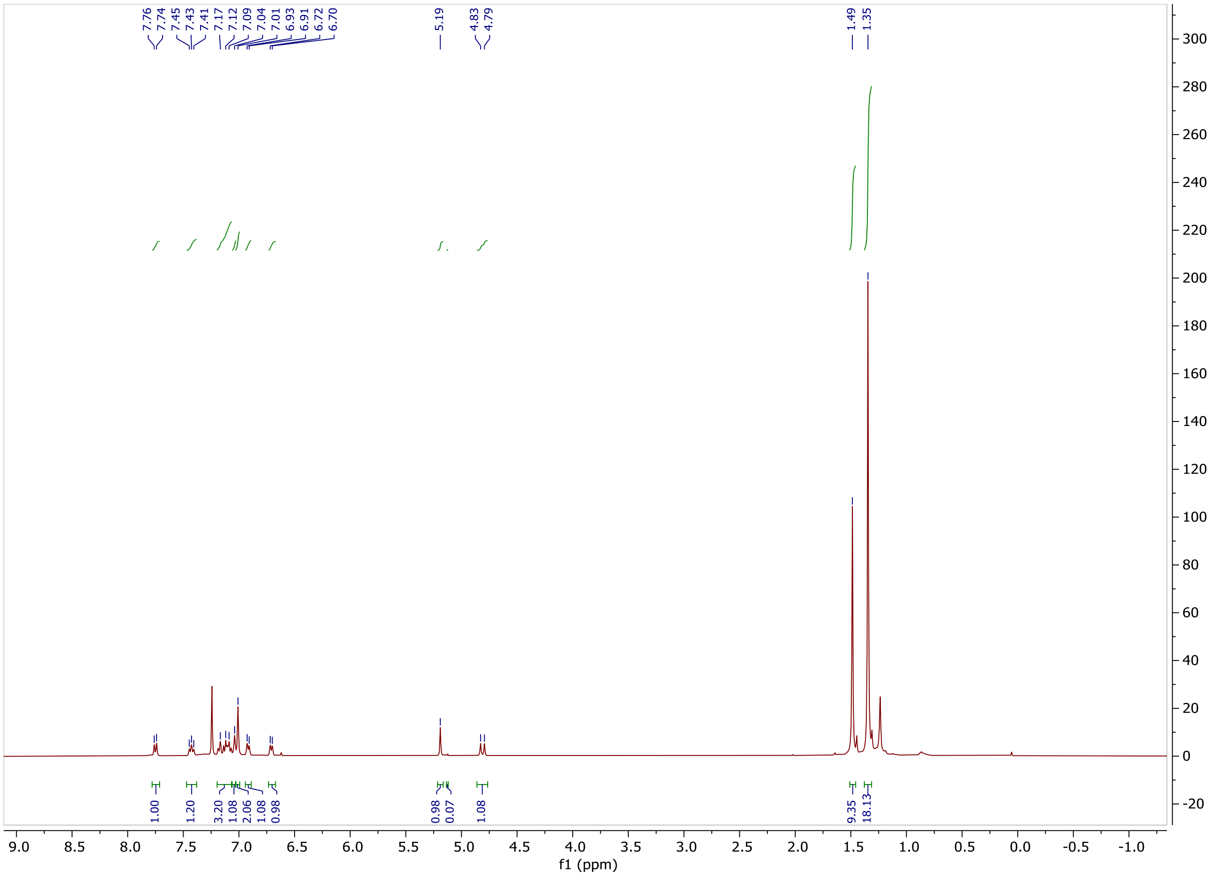

Figure S11. ^{19}F NMR (376 MHz) Spectrum of compound **5** (*dr* = 14:1) in $CDCl_3$.

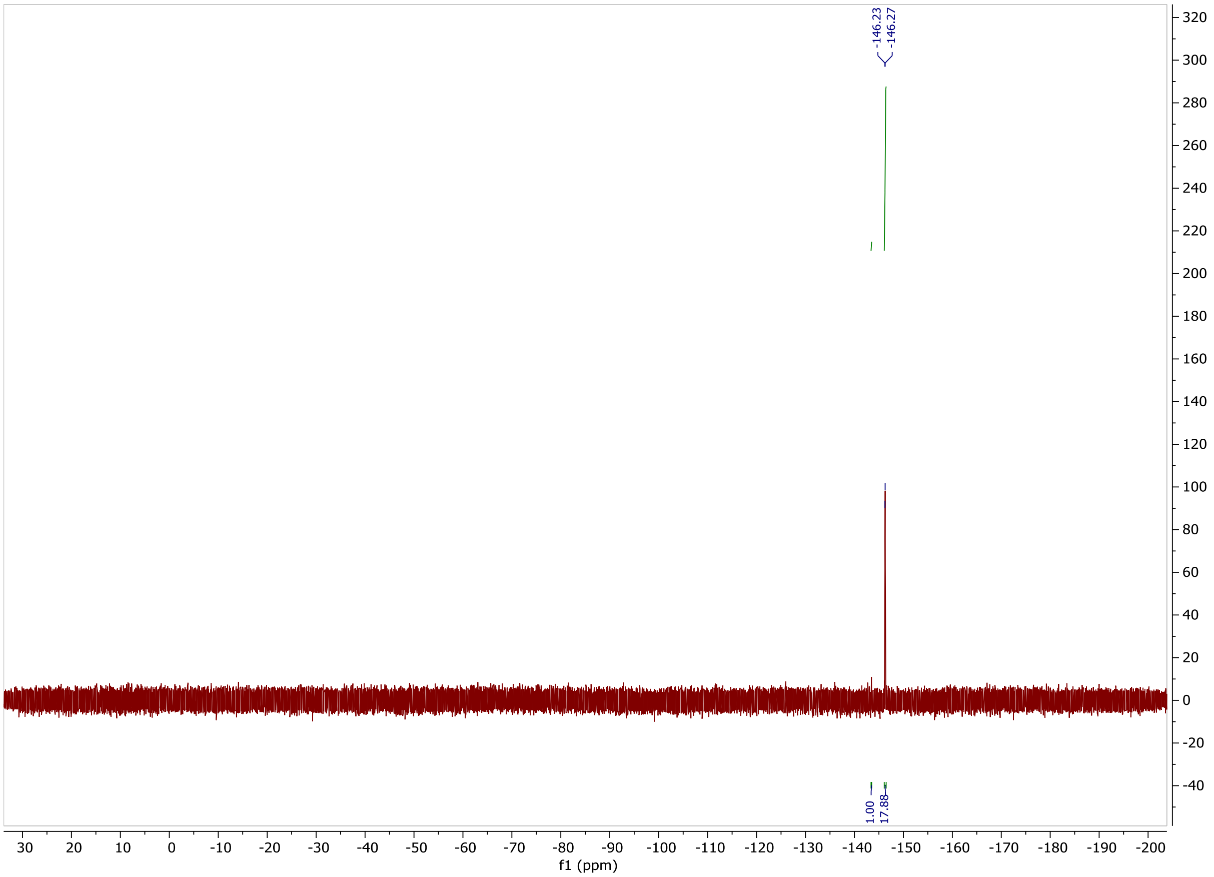

Figure S12. $^{13}C\{1H\}$ NMR (100 MHz) Spectrum of compound **5** (*dr* = 14:1) in $CDCl_3$.

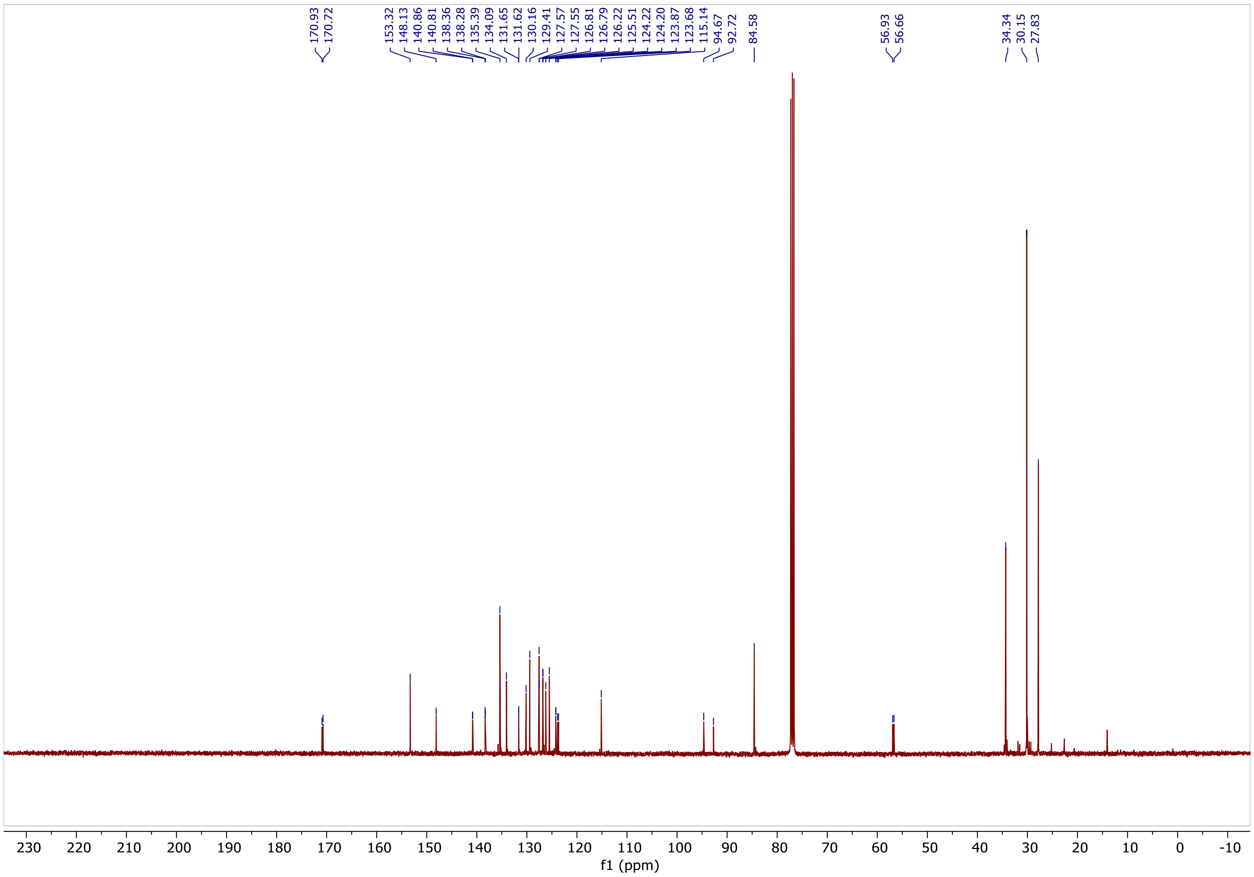

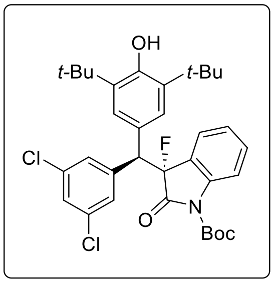

Figure S13. ^{1}H NMR (400 MHz) Spectrum of compound **6** (*dr* = 26:1) in $CDCl_3$.

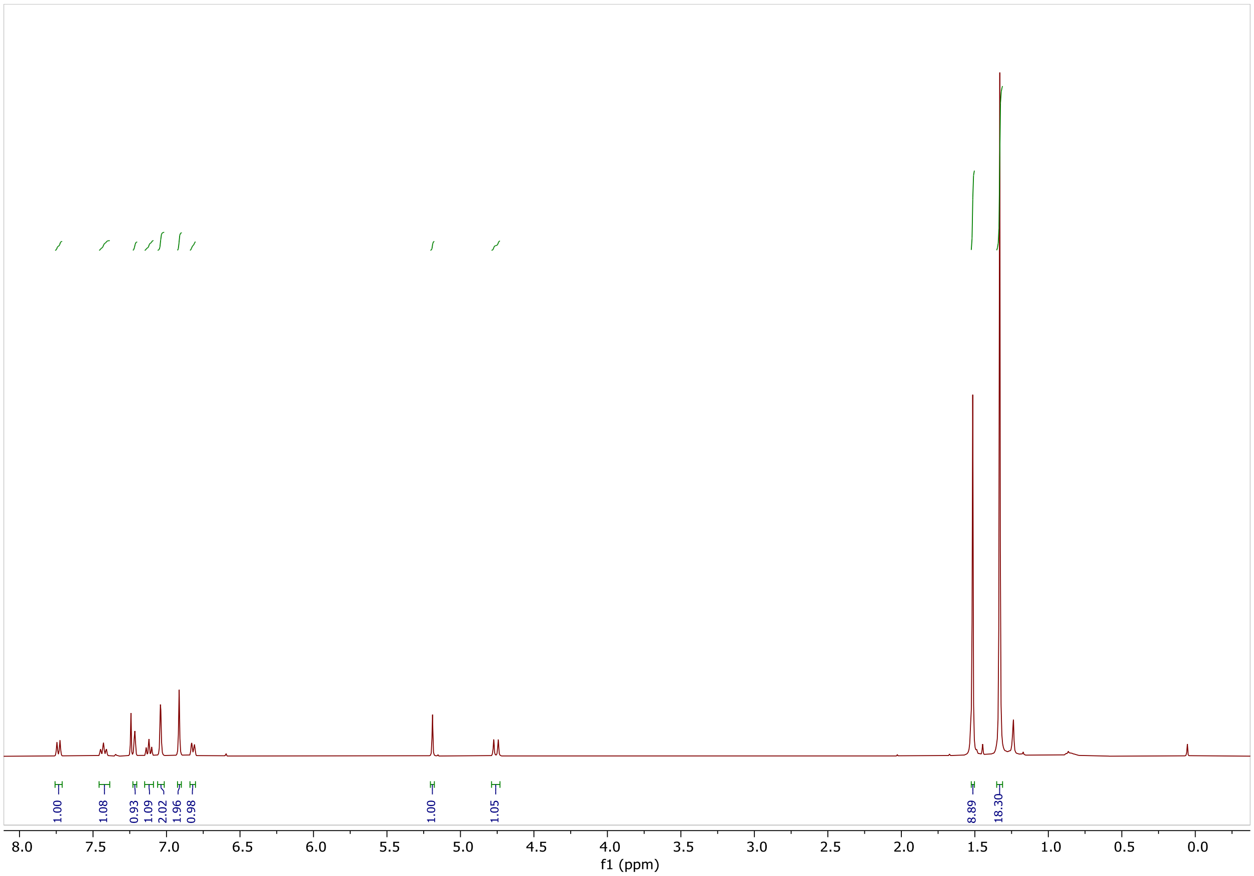

Figure S14. ^{19}F NMR (376 MHz) Spectrum of compound **6** (*dr* = 26:1) in $CDCl_3$.

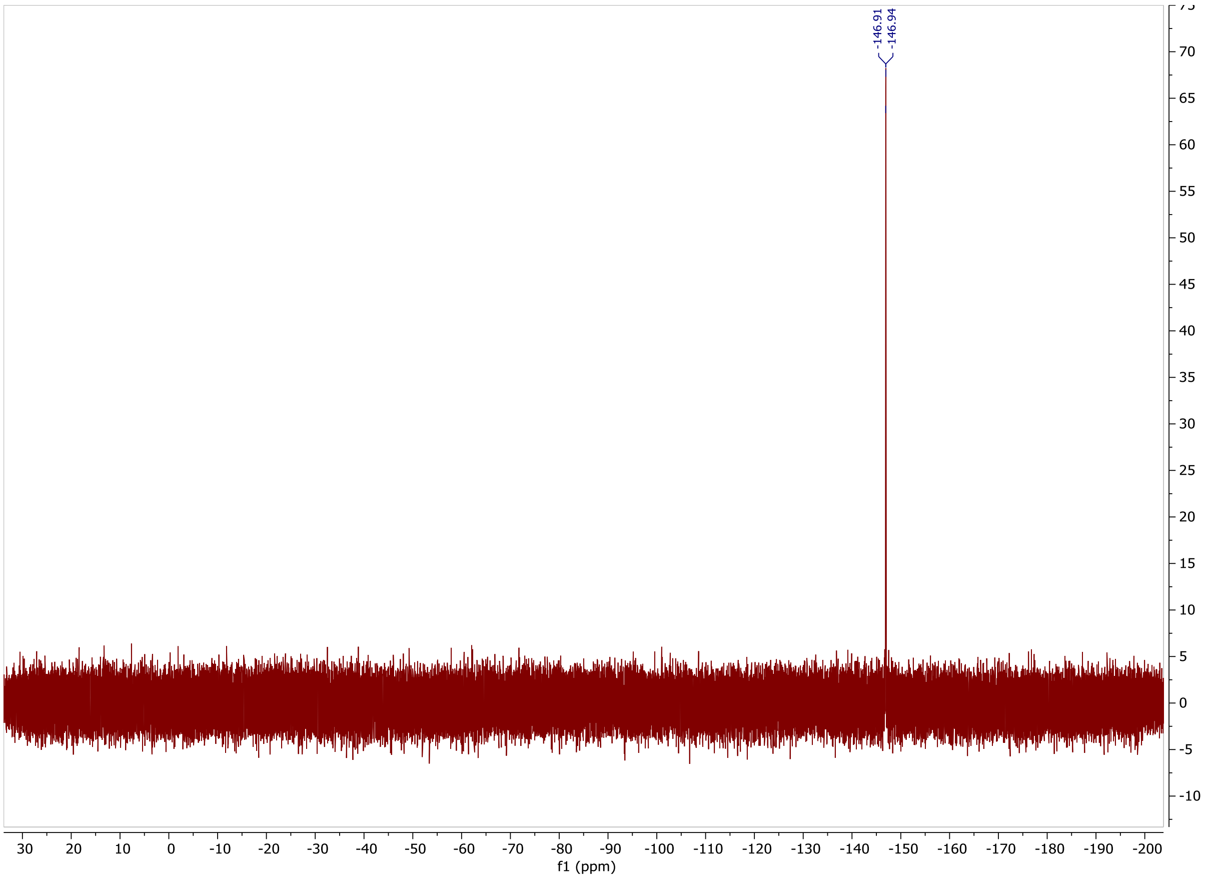

Figure S15. $^{13}C\{1H\}$ NMR (100 MHz) Spectrum of compound **6** (*dr* = 26:1) in $CDCl_3$.

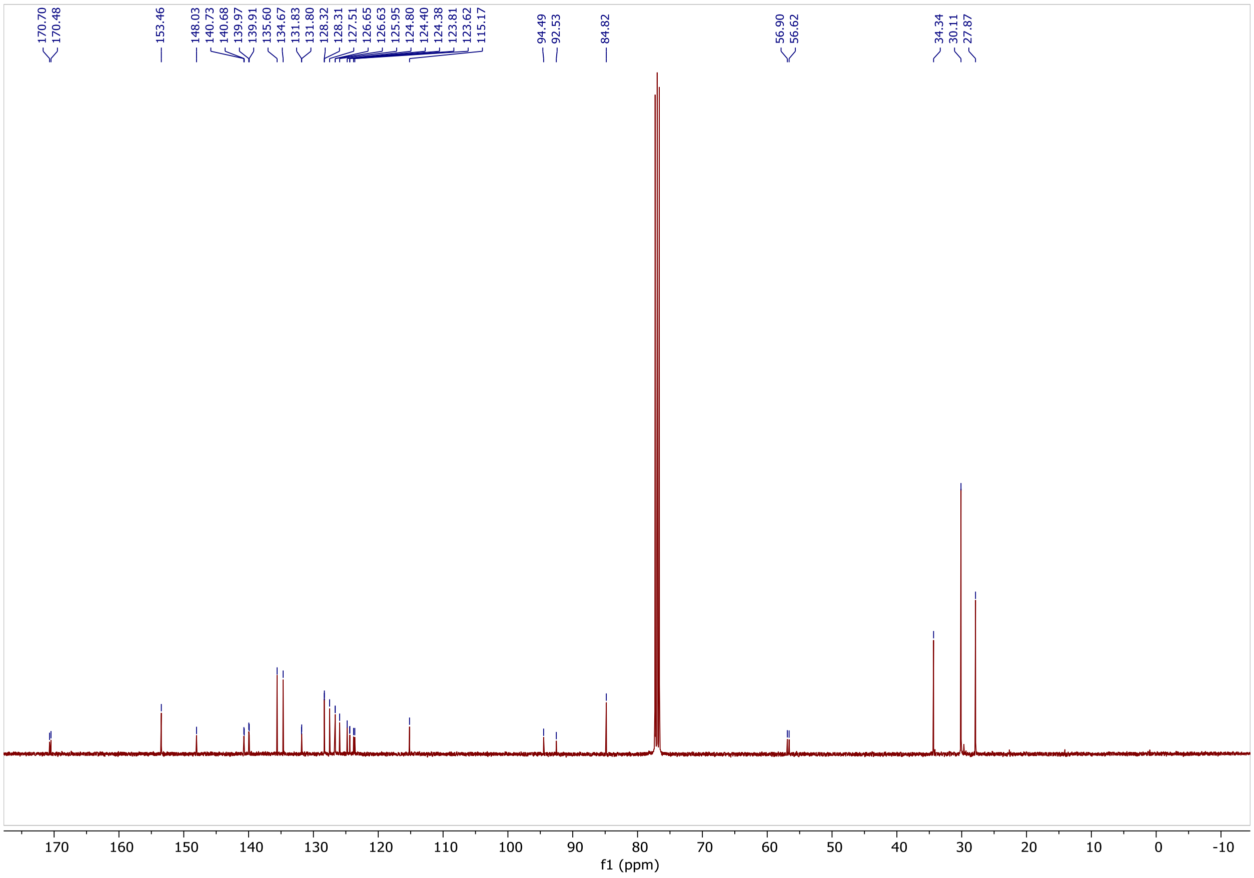

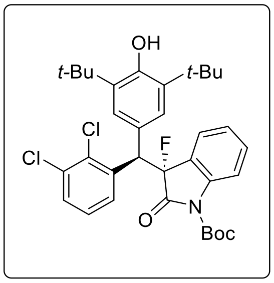

Figure S16. ^{1}H NMR (400 MHz) Spectrum of compound **7** (*dr* = 20:1) in $CDCl_3$.

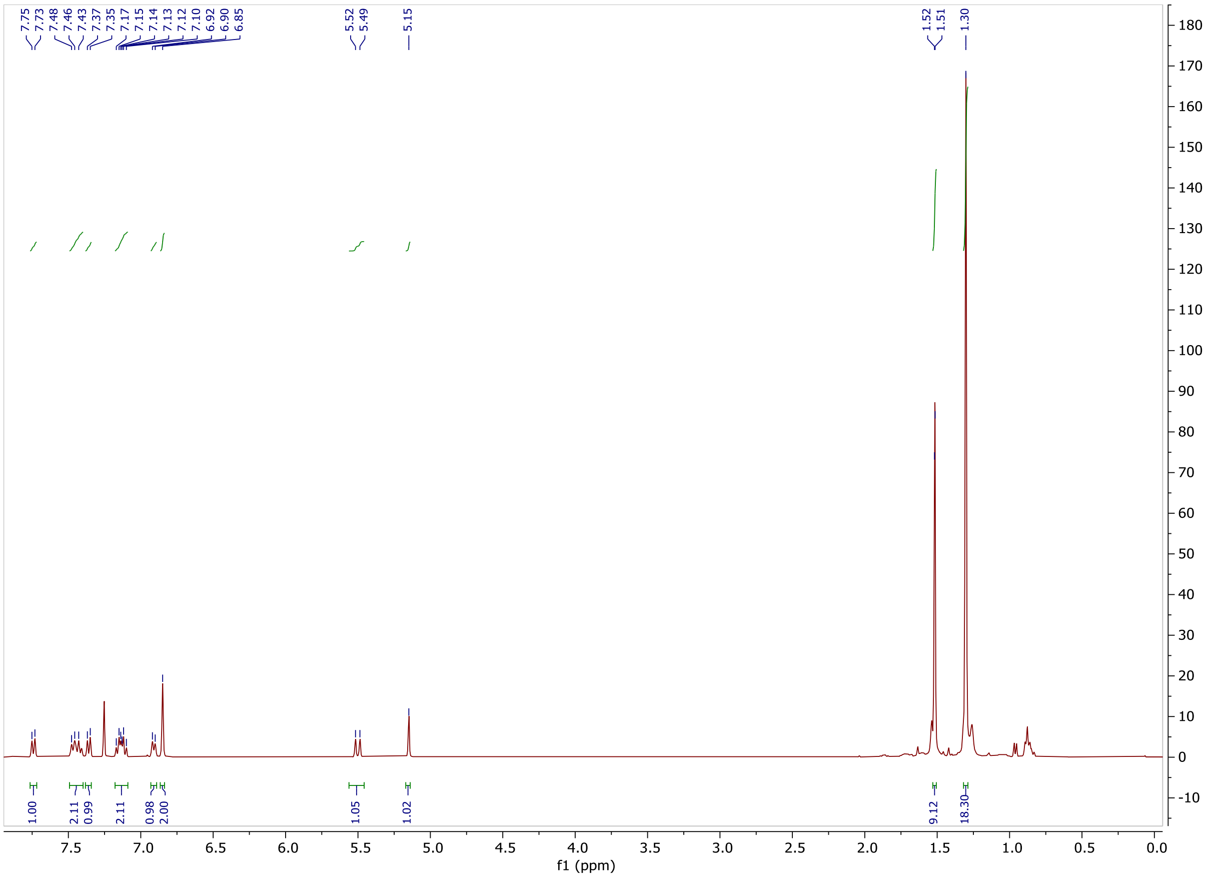

Figure S17. ^{19}F NMR (376 MHz) Spectrum of compound **7** (*dr* = 20:1) in $CDCl_3$.

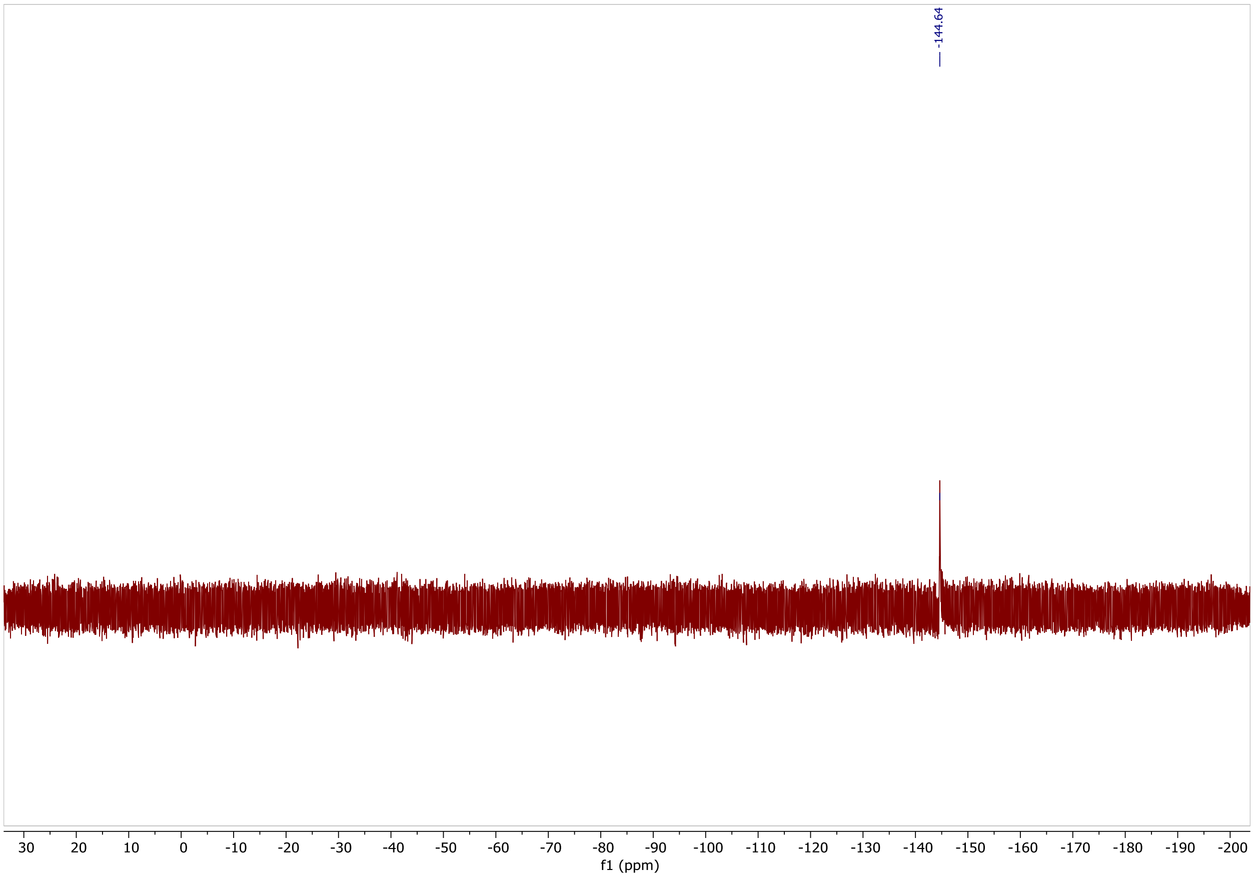

Figure S18. $^{13}C\{1H\}$ NMR (100 MHz) Spectrum of compound **7** (*dr* = 20:1) in $CDCl_3$.

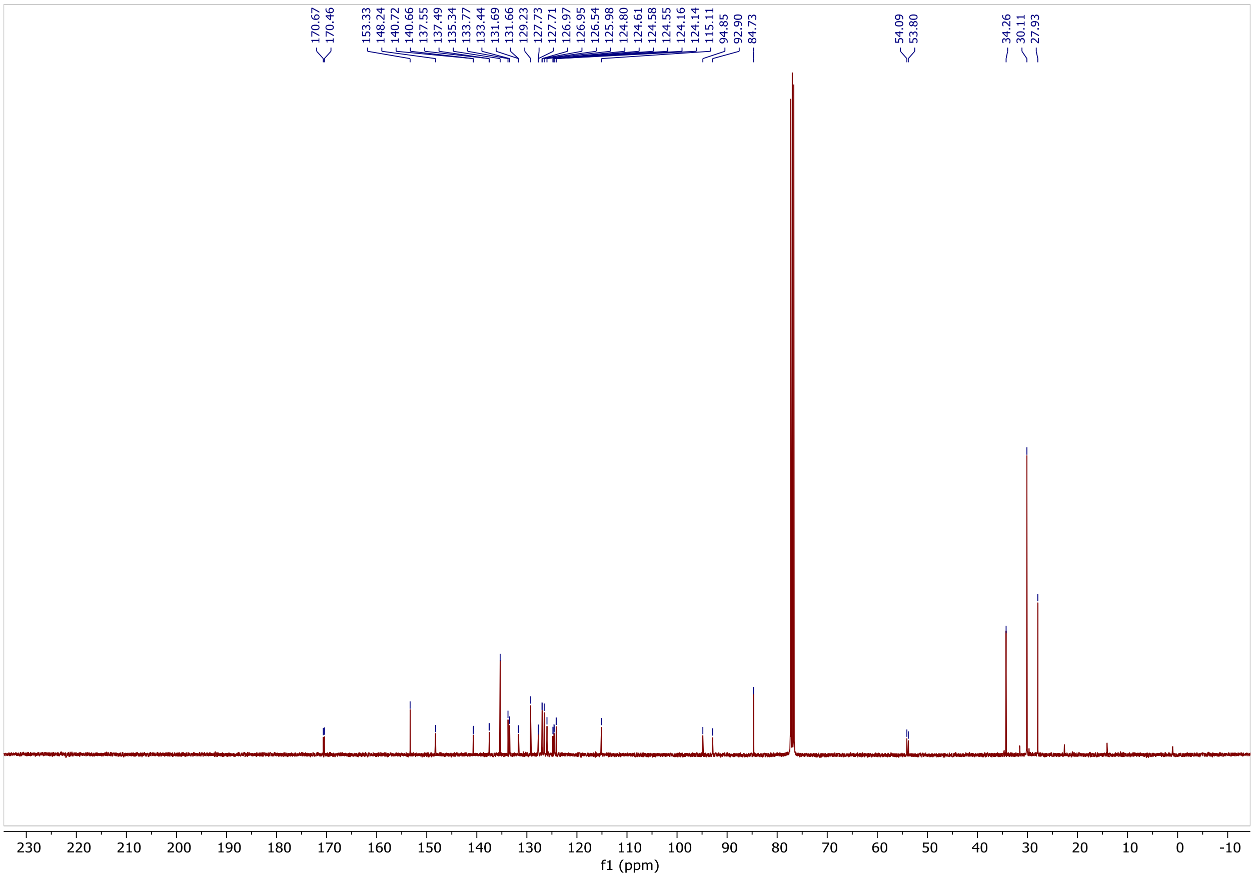

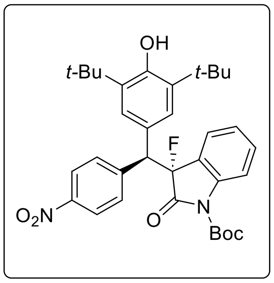

Figure S19. ^{1}H NMR (400 MHz) Spectrum of compound **8** (*dr* = 20:1) in $CDCl_3$.

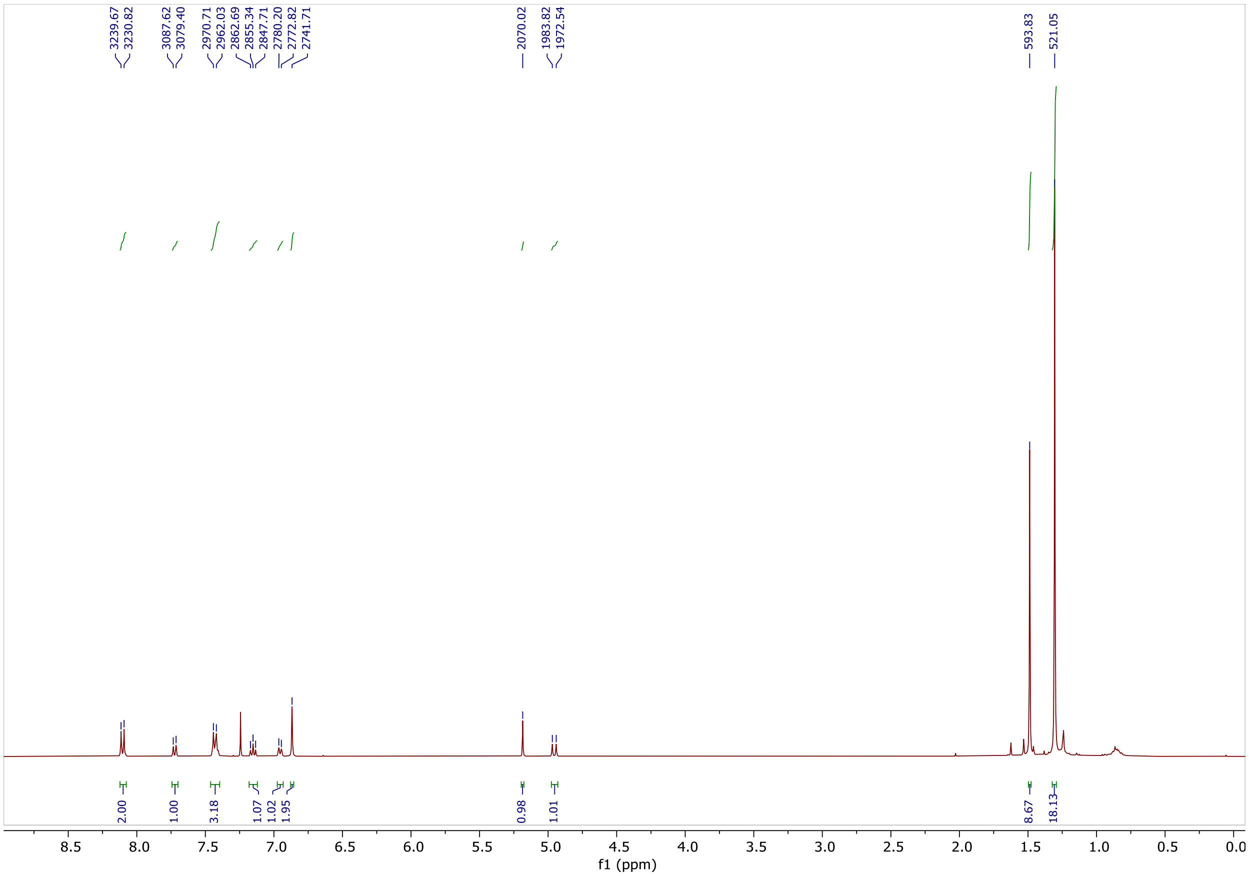

Figure S20. ^{19}F NMR (376 MHz) Spectrum of compound **8** (*dr* = 20:1) in $CDCl_3$.

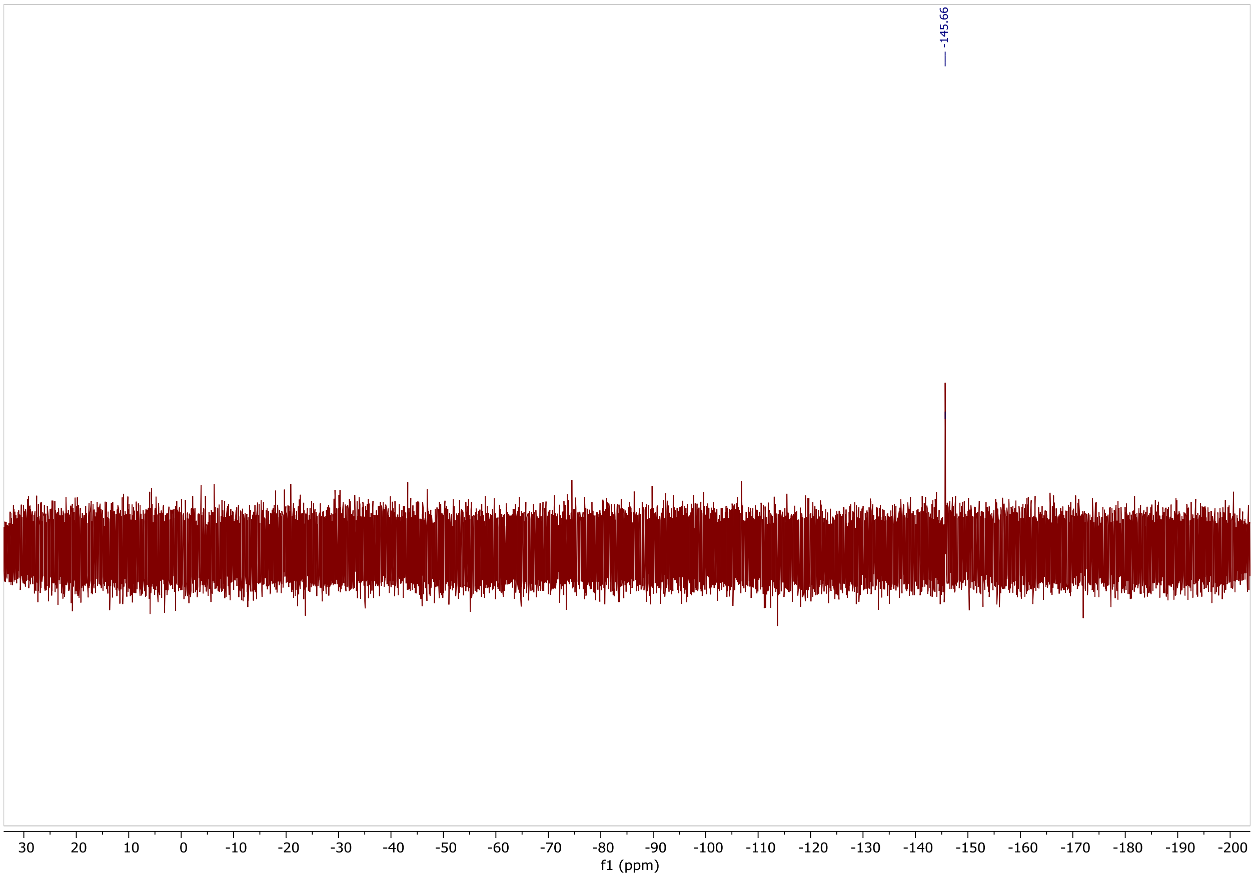

Figure S21. $^{13}C\{1H\}$ NMR (100 MHz) Spectrum of compound **8** (*dr* = 20:1) in $CDCl_3$.

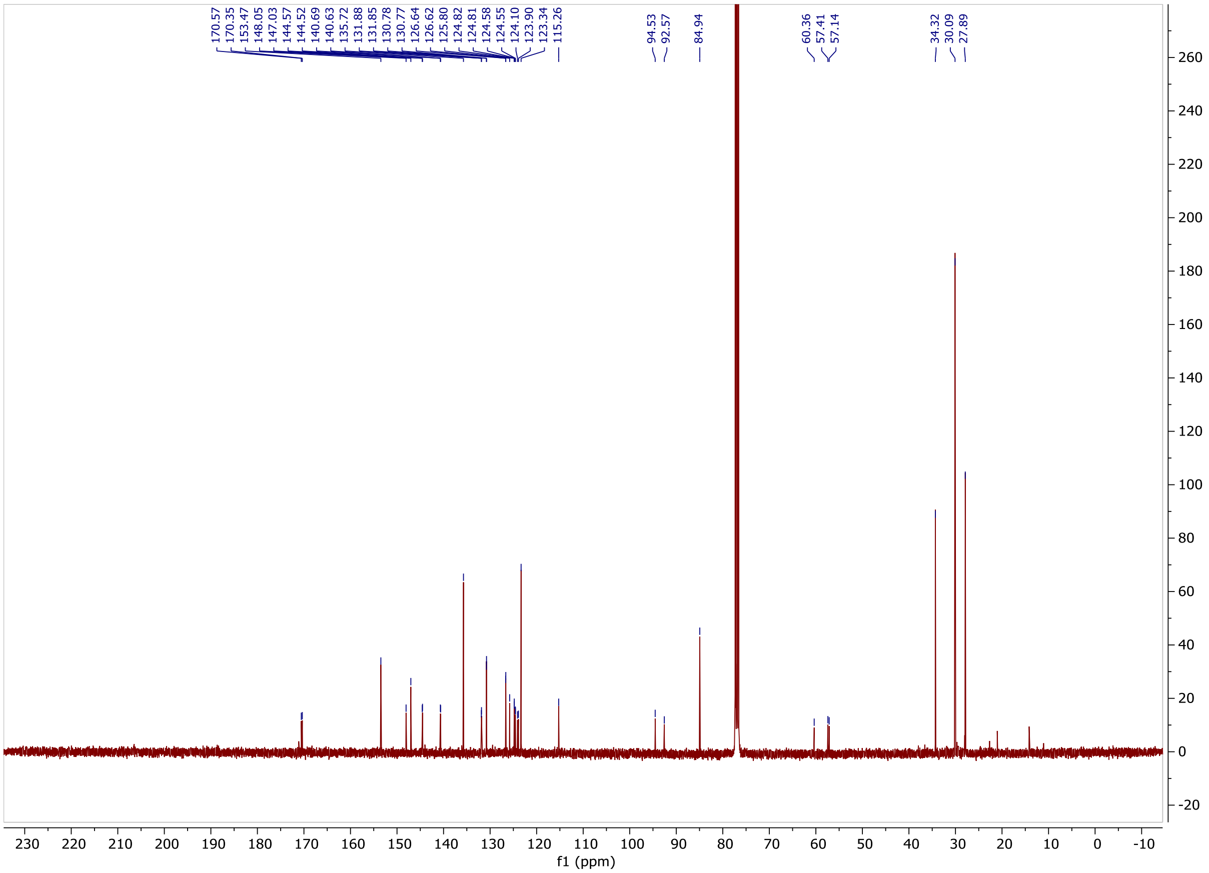

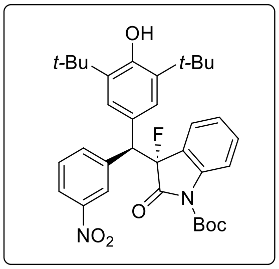

Figure S22. ^{1}H NMR (400 MHz) Spectrum of compound **9** (*dr* = 50:1) in $CDCl_3$.

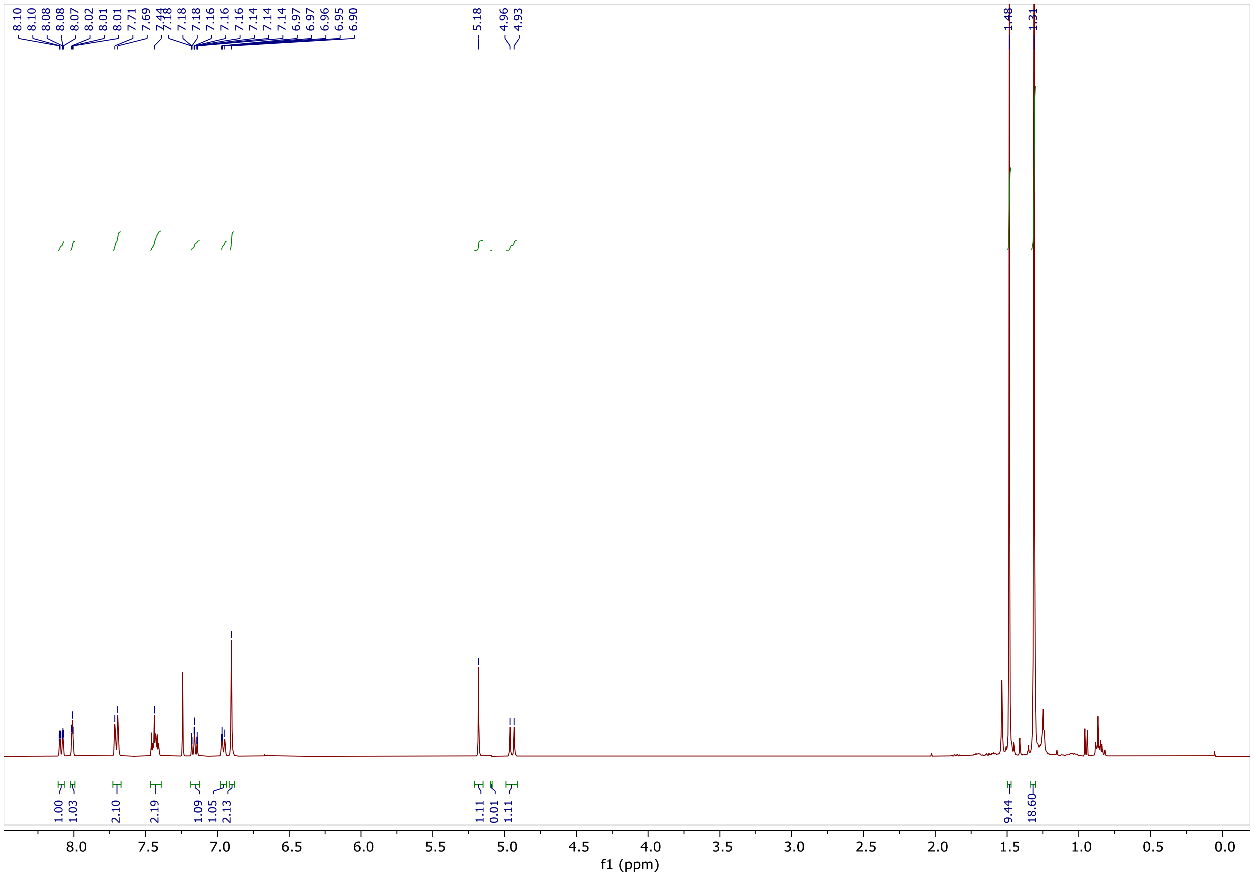

Figure S23. ^{19}F NMR (376 MHz) Spectrum of compound **9** (*dr* = 50:1) in $CDCl_3$.

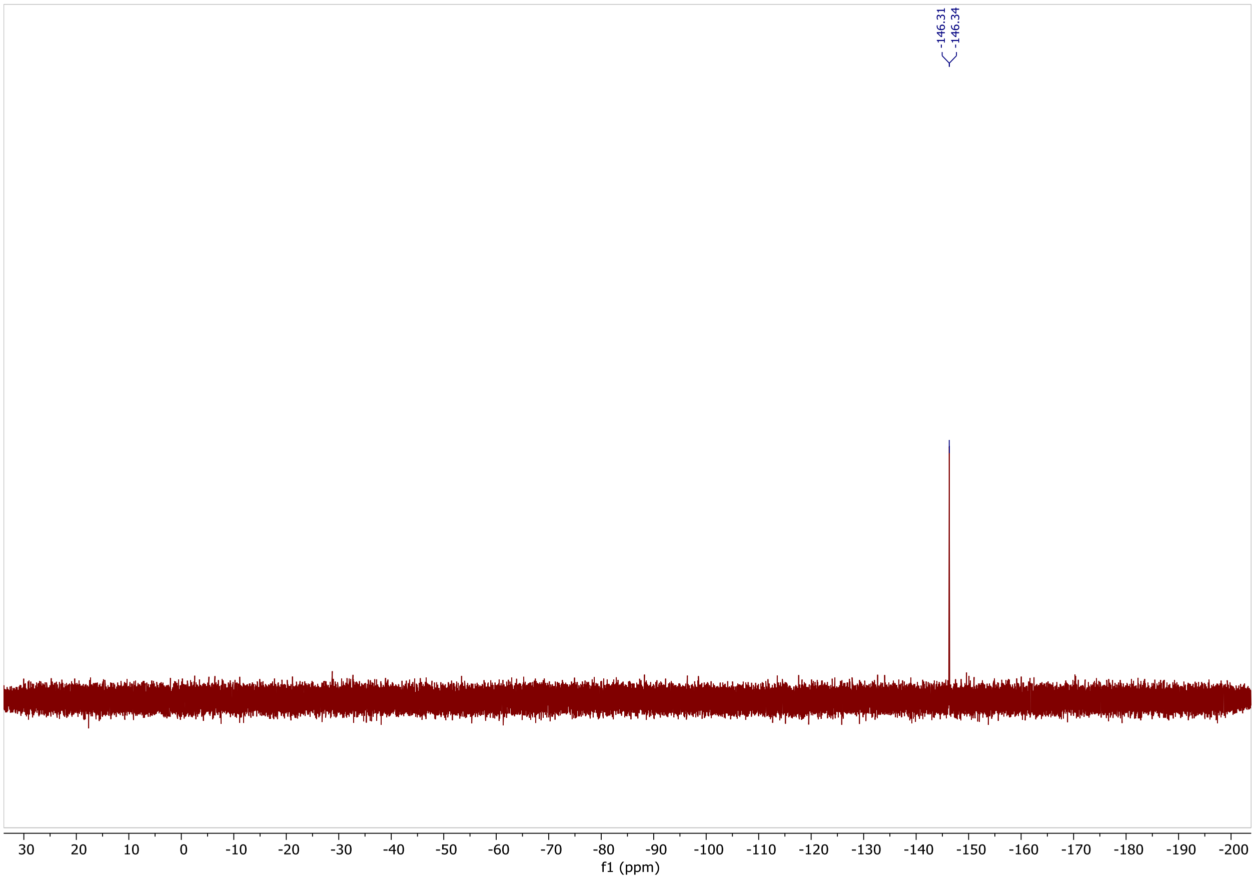

Figure S24. $^{13}C\{1H\}$ NMR (100 MHz) Spectrum of compound **9** (*dr* = 50:1) in $CDCl_3$.

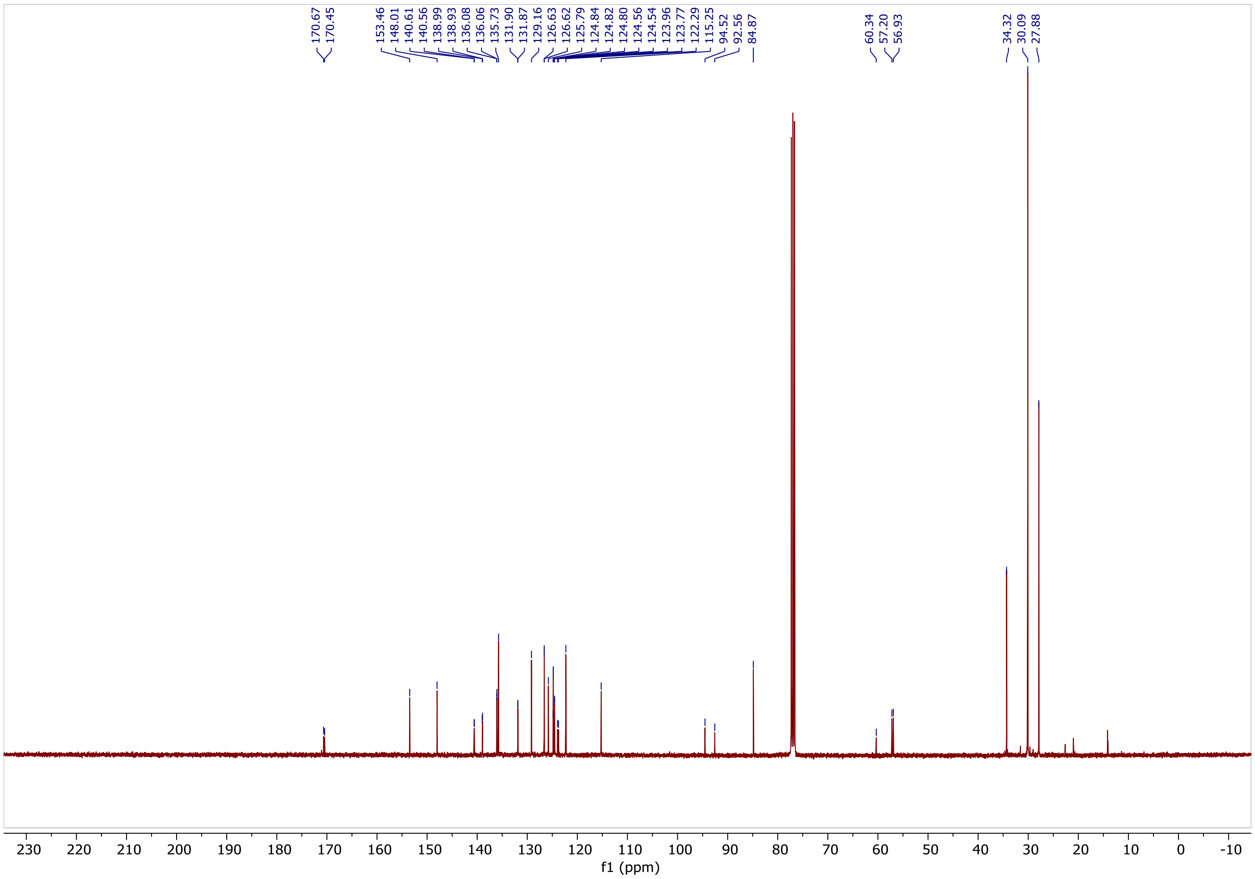

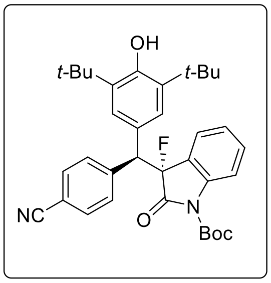

Figure S25. ^{1}H NMR (400 MHz) Spectrum of compound **10** (*dr* = 20:1) in $CDCl_3$.

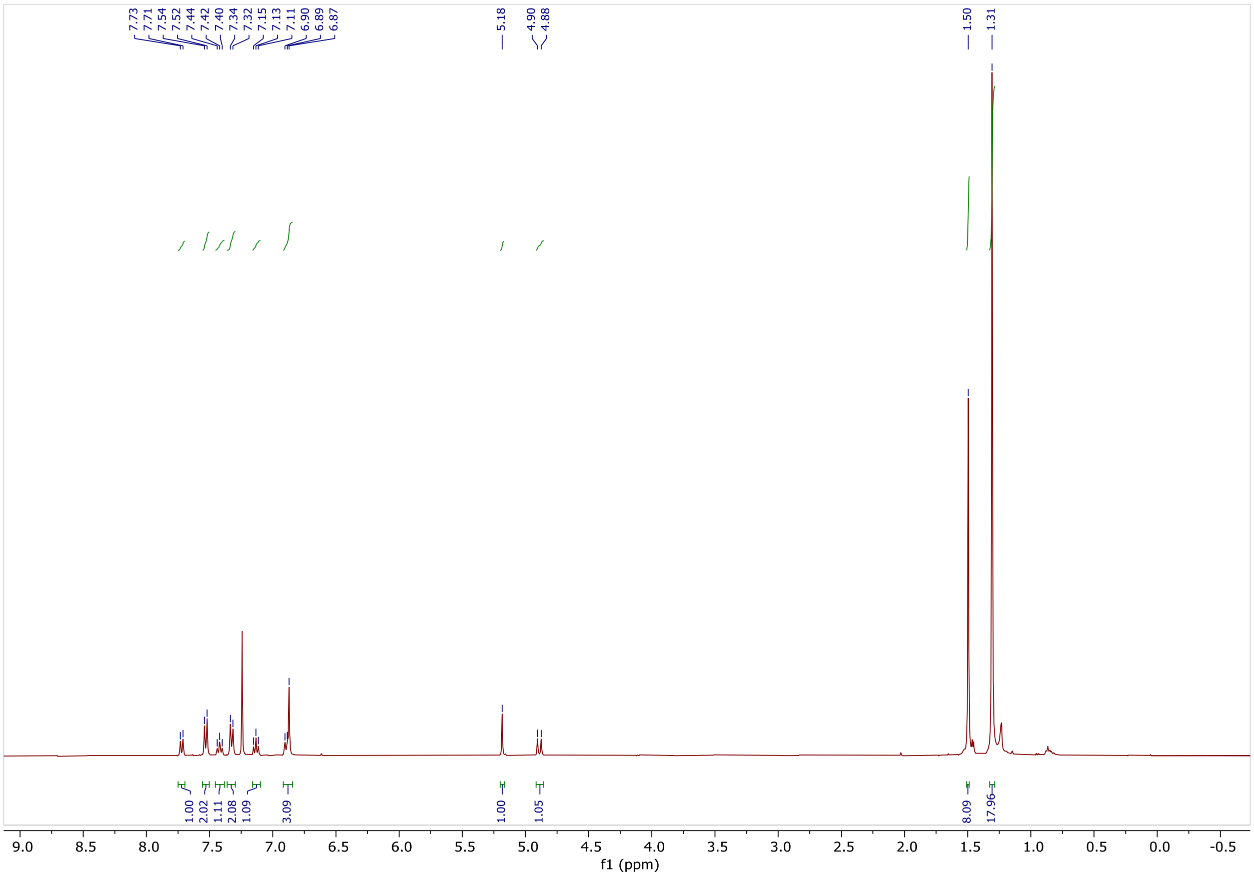

Figure S26. ^{19}F NMR (376 MHz) Spectrum of compound **10** (*dr* = 20:1) in $CDCl_3$.

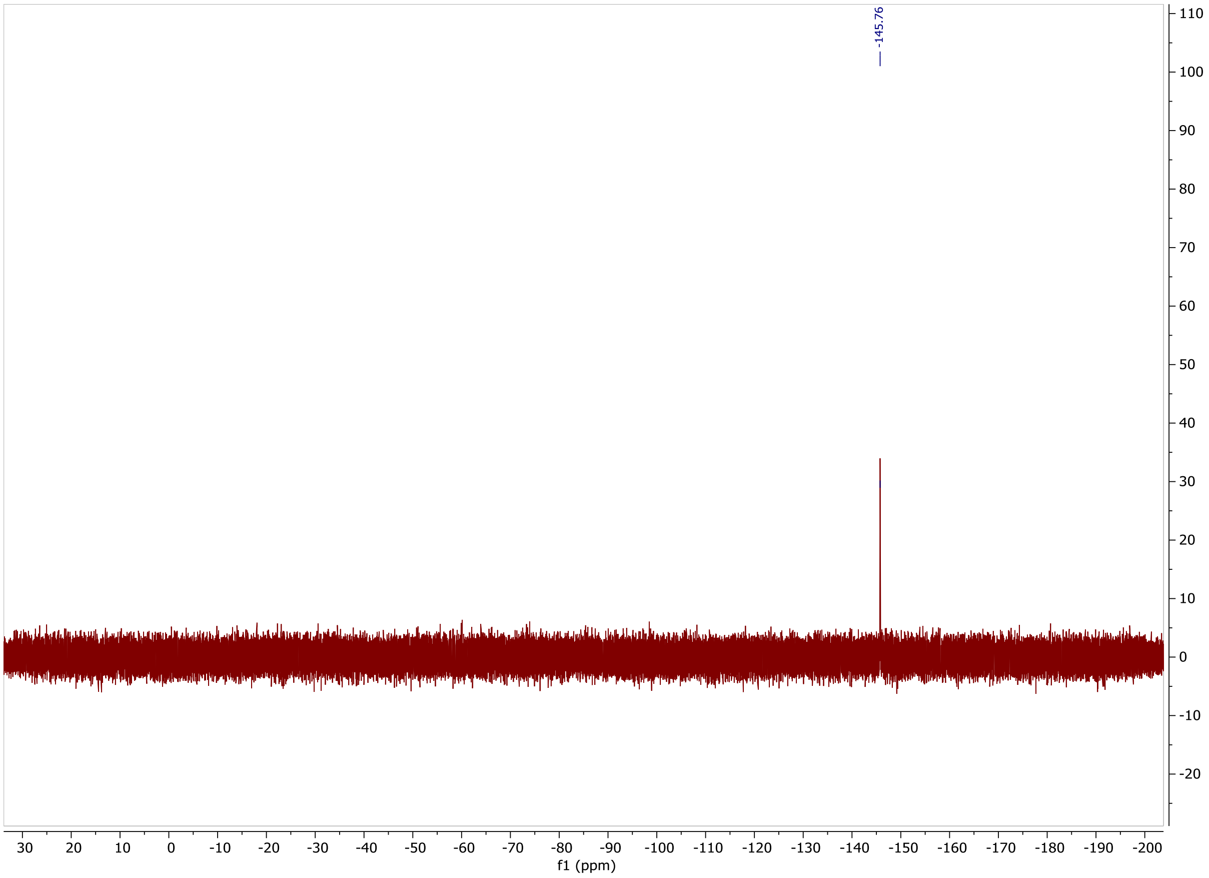

Figure S27. ^{13}C{1H} NMR (100 MHz) Spectrum of compound **10** (*dr* = 20:1) in $CDCl_3$.

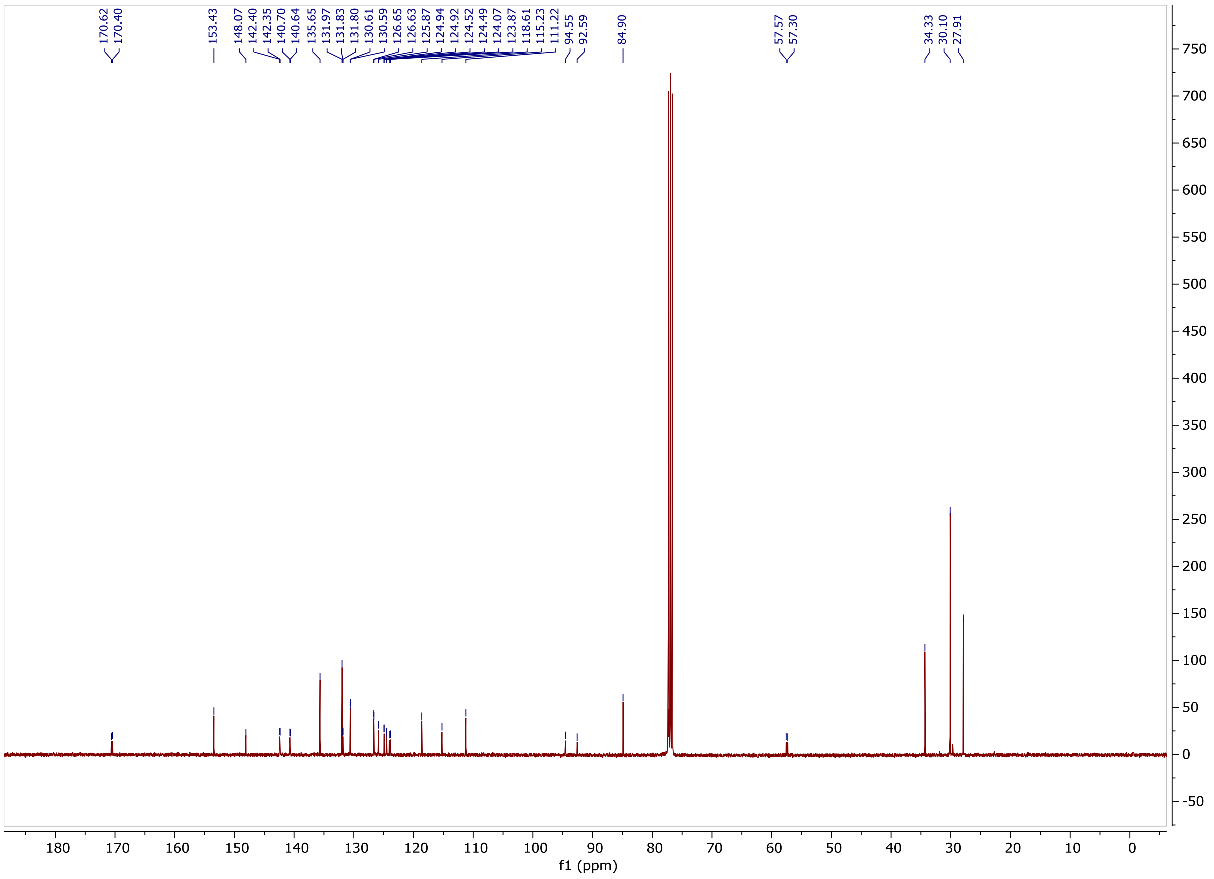

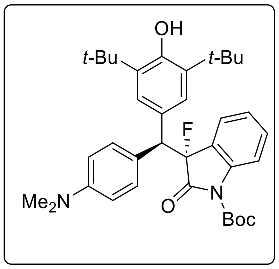

Figure S28. ^{1}H NMR (400 MHz) Spectrum of compound **11** (*dr* = 35:1) in $CDCl_3$.

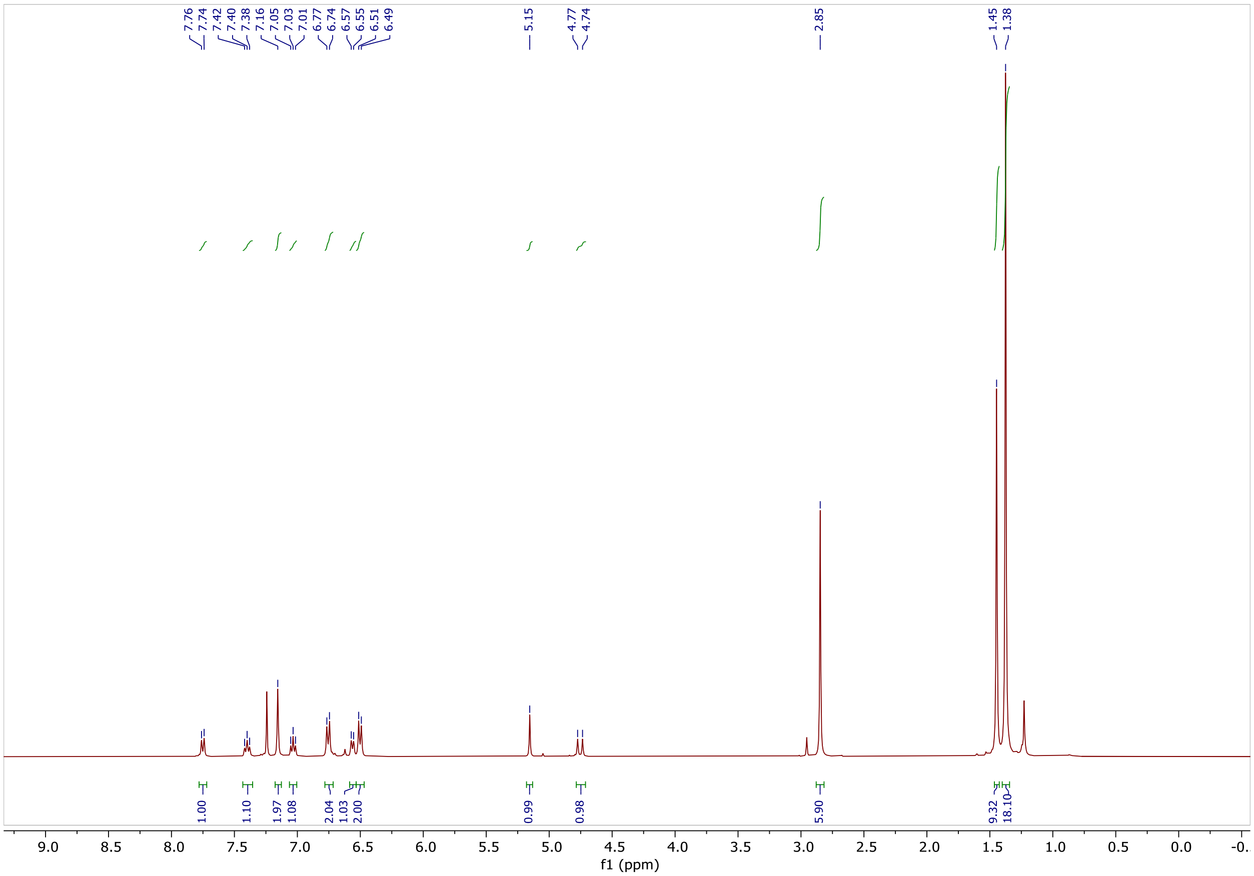

Figure S29. ^{19}F NMR (376 MHz) Spectrum of compound **11** (*dr* = 35:1) in $CDCl_3$.

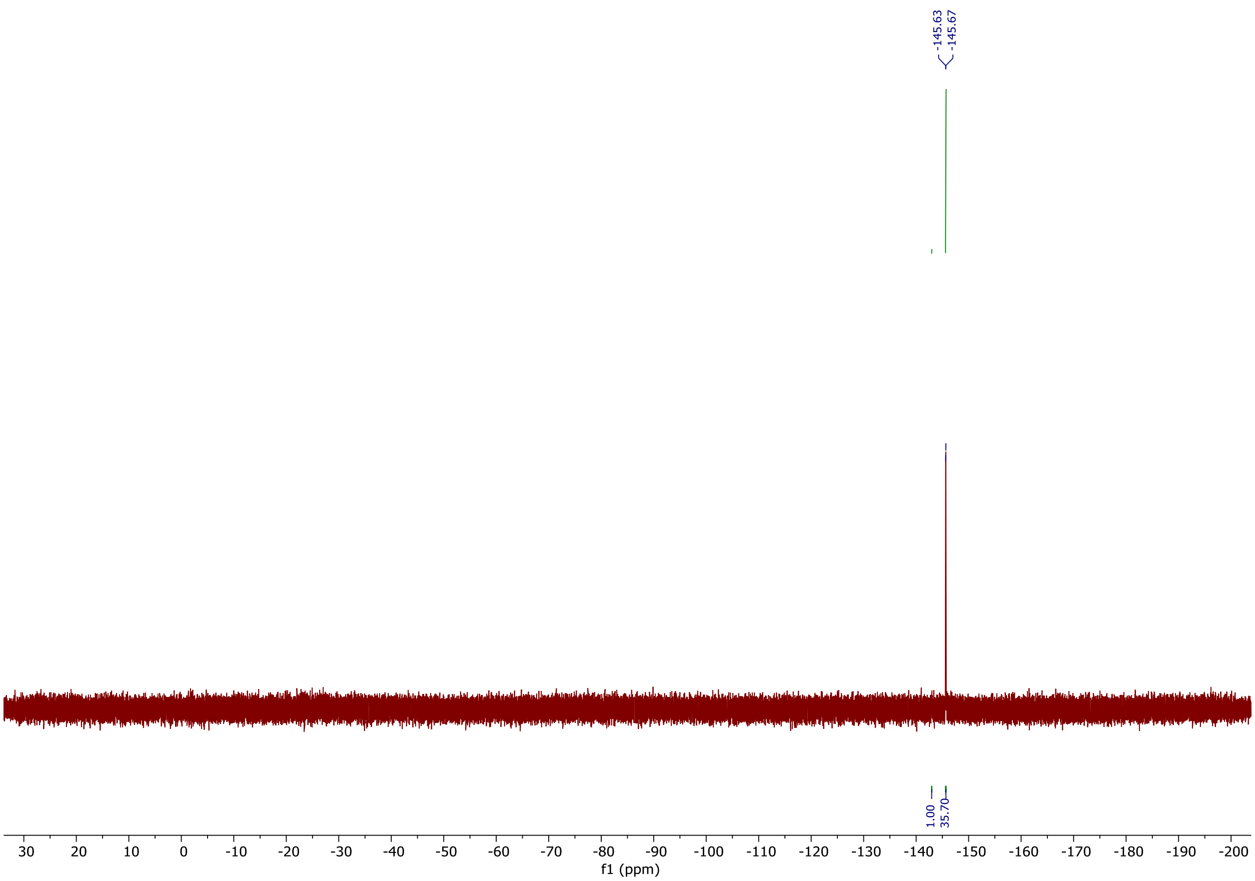

Figure S30. $^{13}C\{1H\}$ NMR (100 MHz) Spectrum of compound **11** (*dr* = 35:1) in $CDCl_3$.

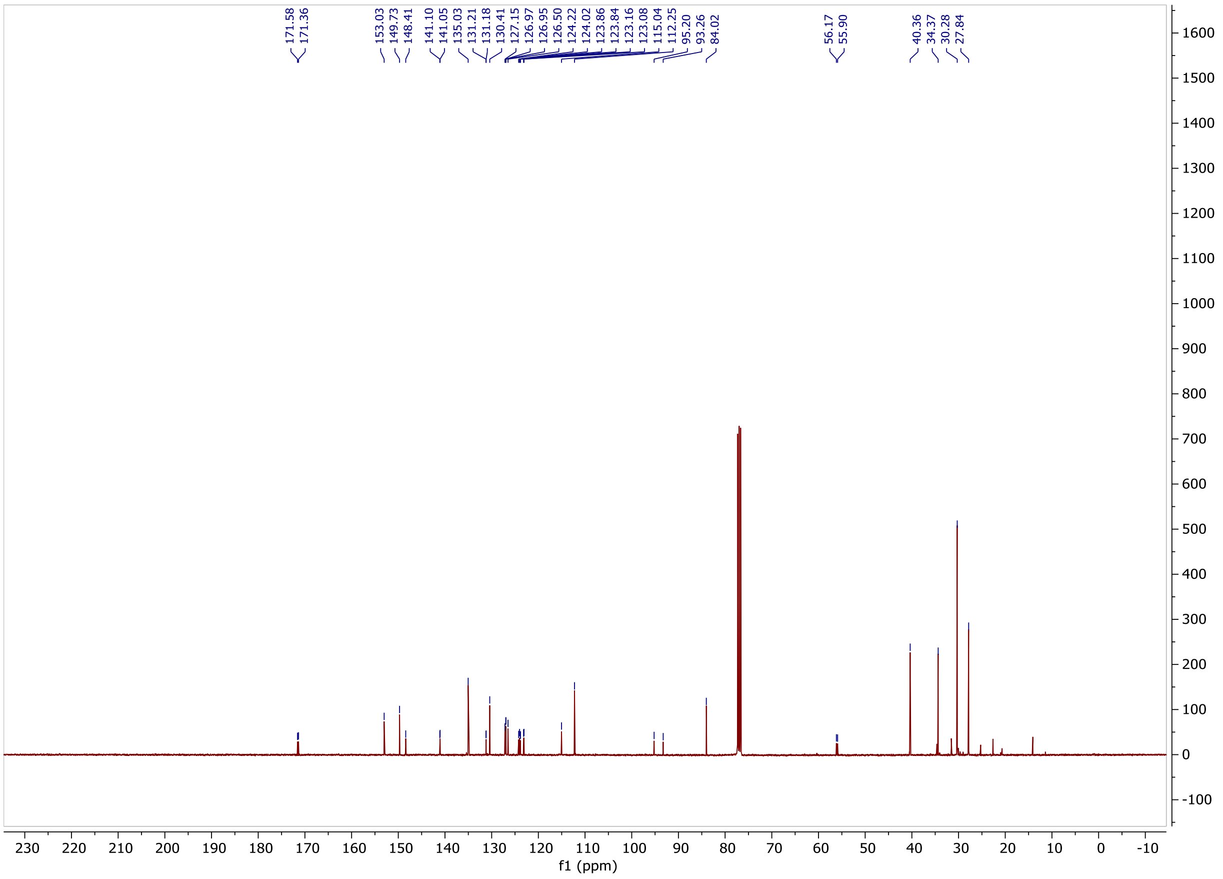

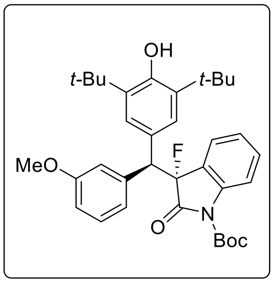

Figure S31. ^{1}H NMR (400 MHz) Spectrum of compound **12** (*dr* = 24:1) in $CDCl_3$.

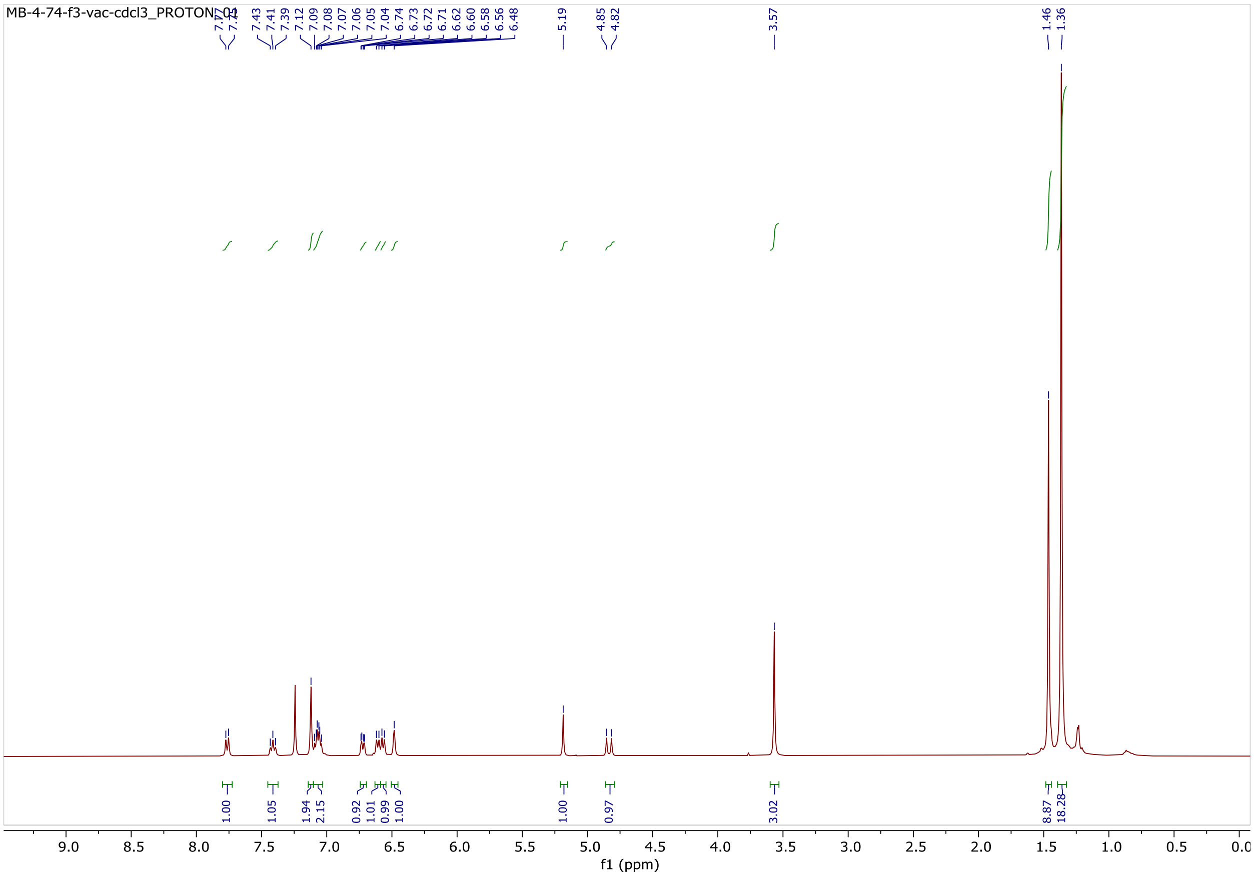

Figure S32. ^{19}F NMR (376 MHz) Spectrum of compound **12** (*dr* = 24:1) in $CDCl_3$.

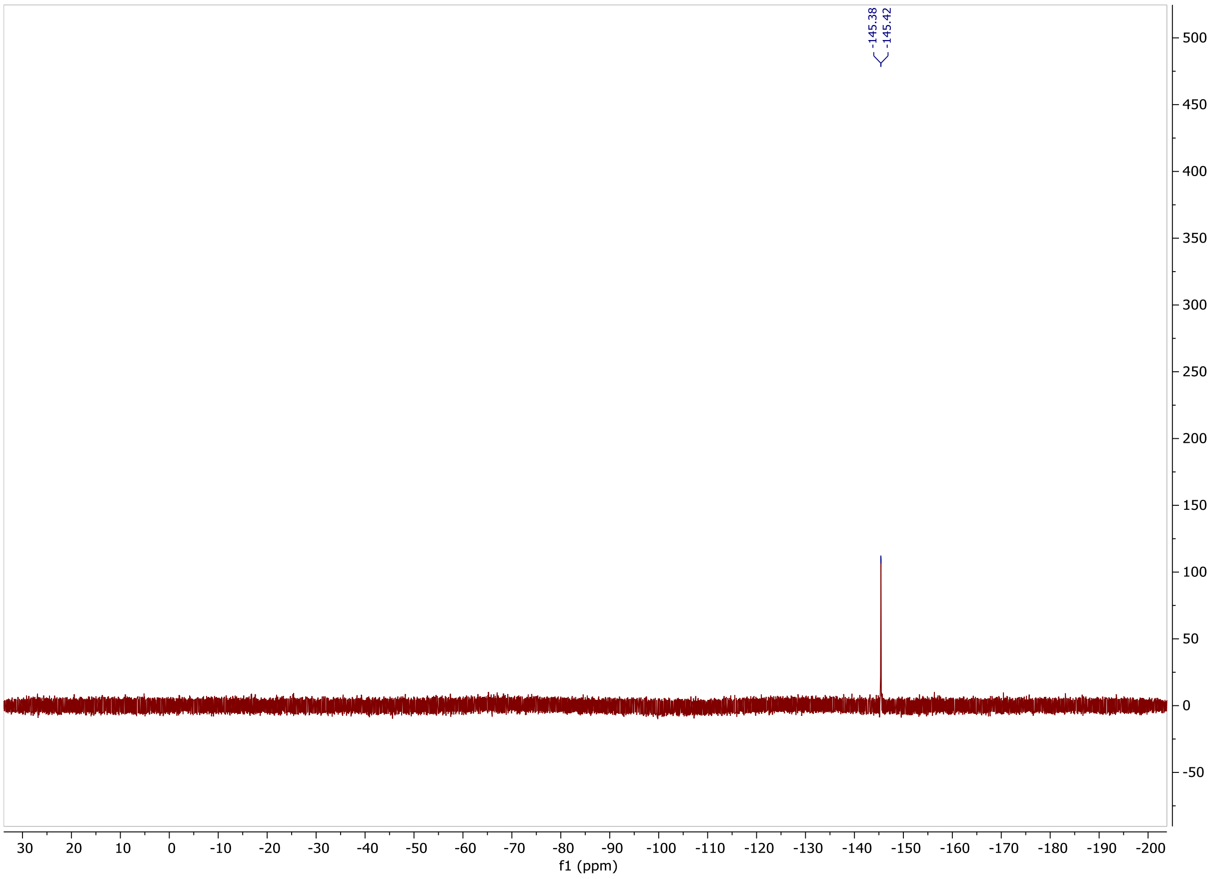

Figure S33. $^{13}C\{1H\}$ NMR (100 MHz) Spectrum of compound **12** (*dr* = 24:1) in $CDCl_3$.

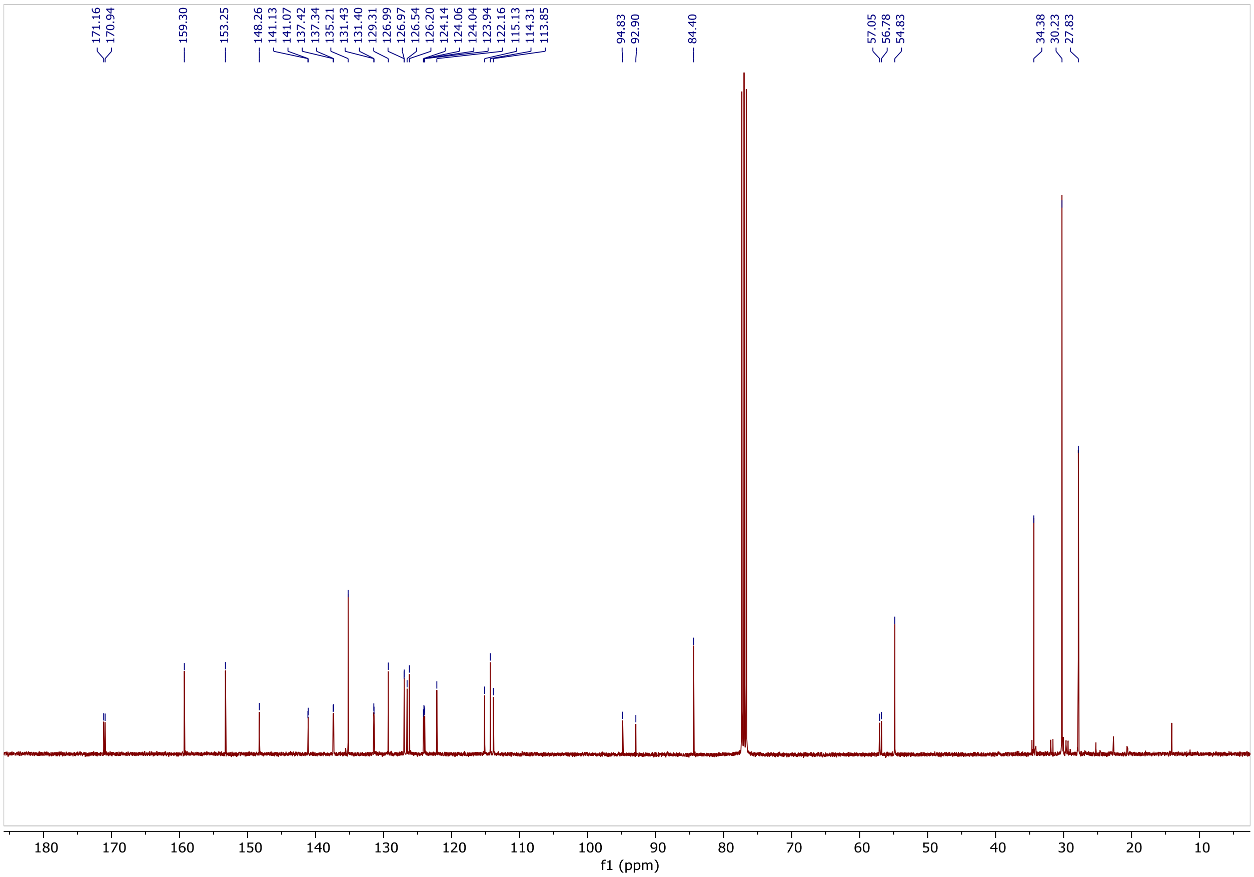

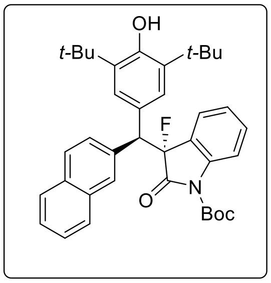

Figure S34. ^{1}H NMR (400 MHz) Spectrum of compound **13** (*dr* = 53:1) in $CDCl_3$.

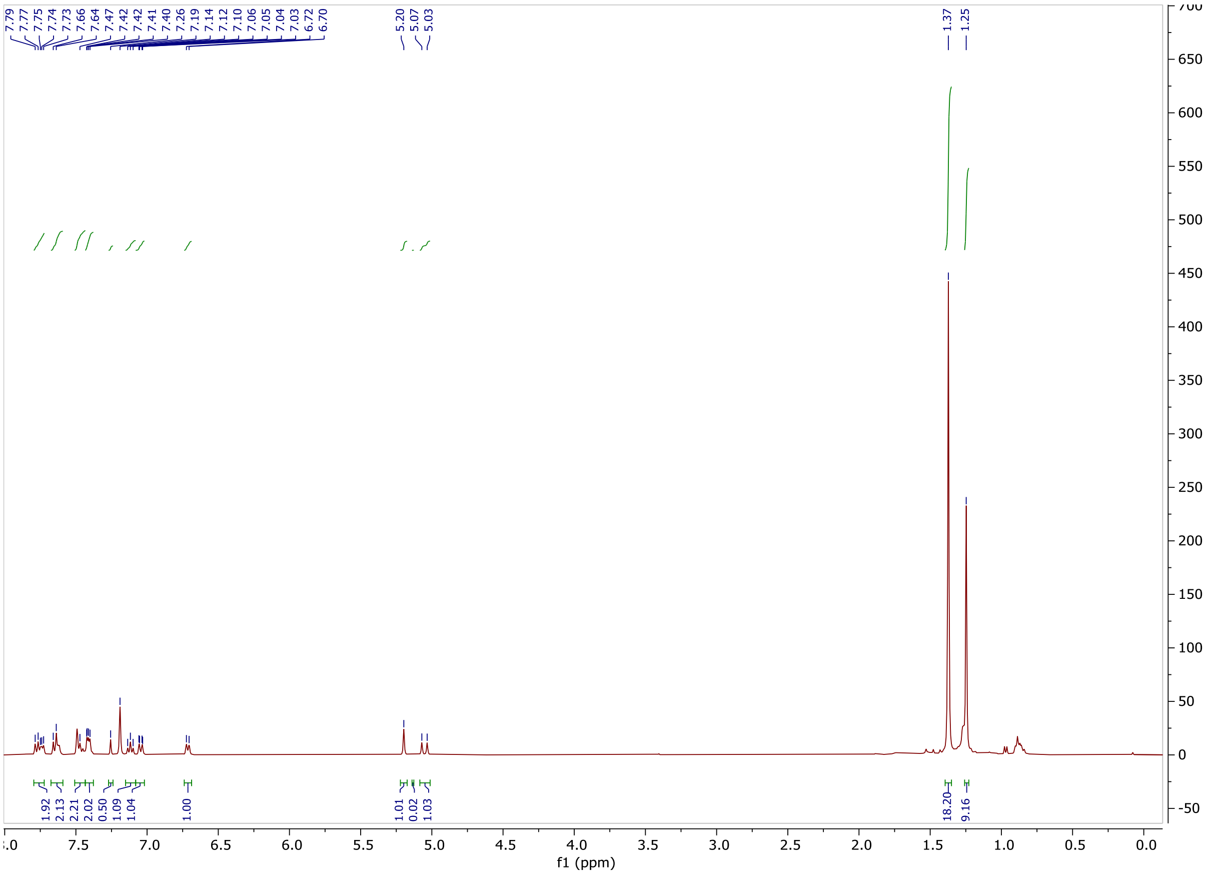

Figure S35. ^{19}F NMR (376 MHz) Spectrum of compound **13** (*dr* = 53:1) in $CDCl_3$.

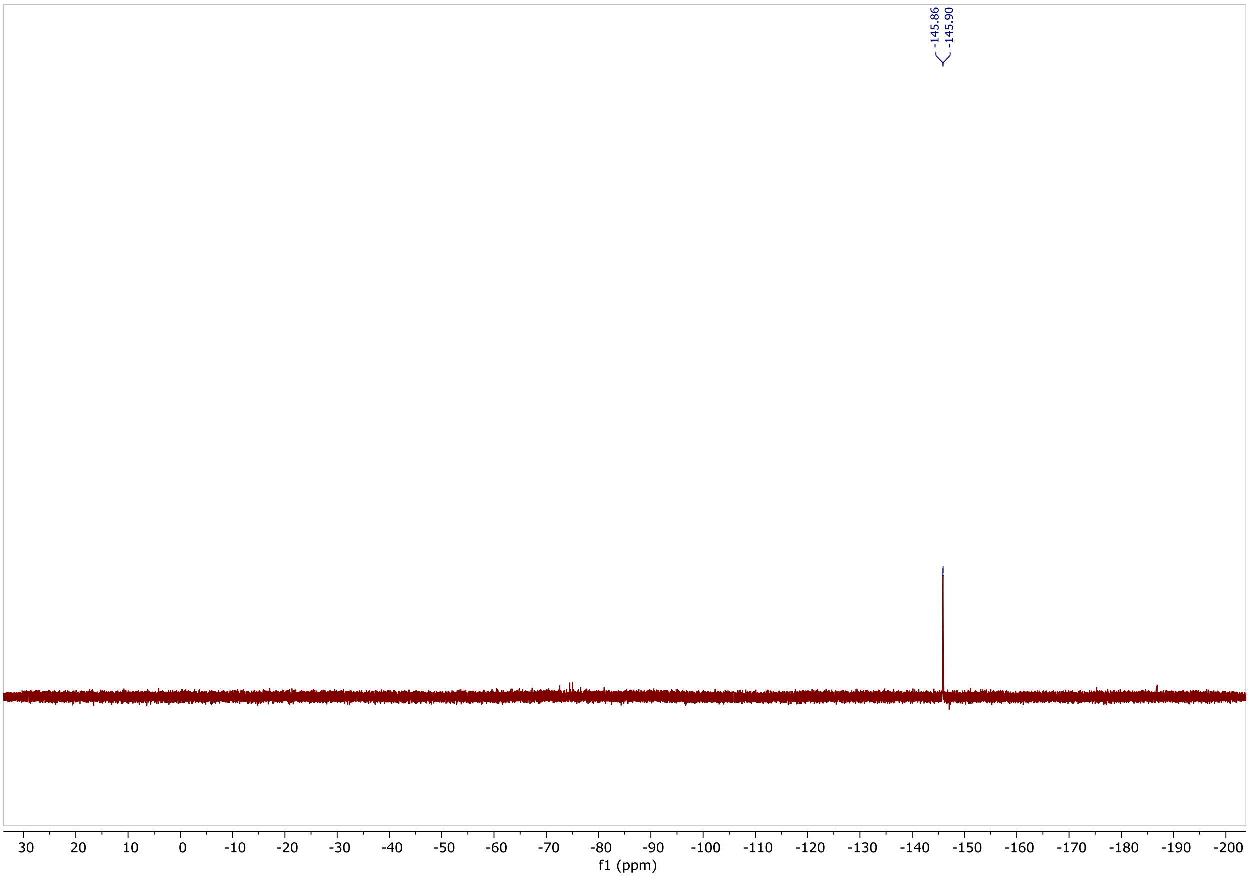

Figure S36. $^{13}C\{1H\}$ NMR (100 MHz) Spectrum of compound **13** (*dr* = 53:1) in $CDCl_3$.

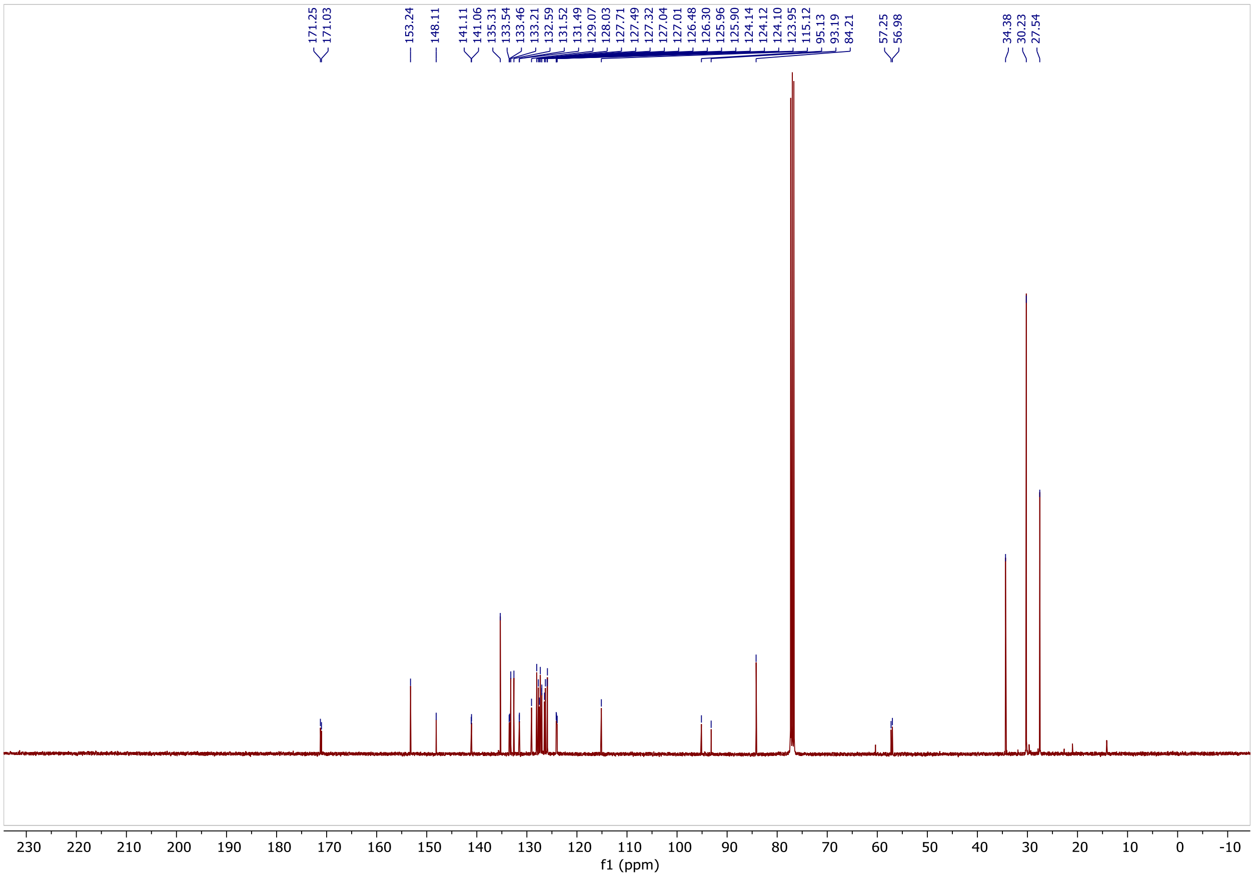

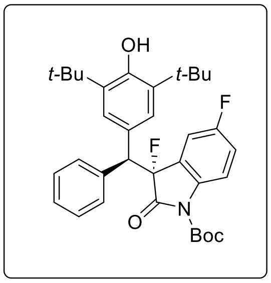

Figure S37. ^{1}H NMR (400 MHz) Spectrum of compound **14** (*dr* = 13:1) in $CDCl_3$.

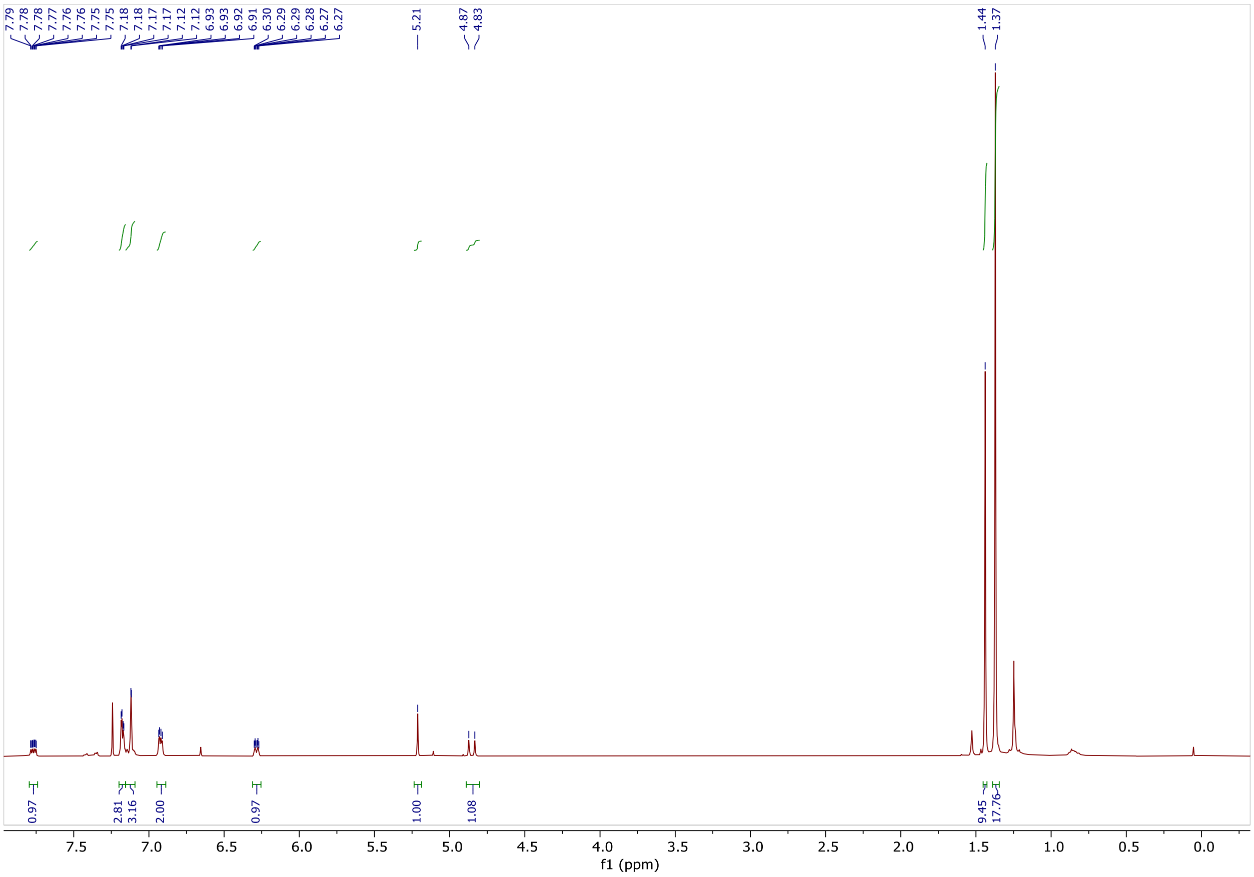

Figure S38. ^{19}F NMR (376 MHz) Spectrum of compound **14** (*dr* = 13:1) in $CDCl_3$.

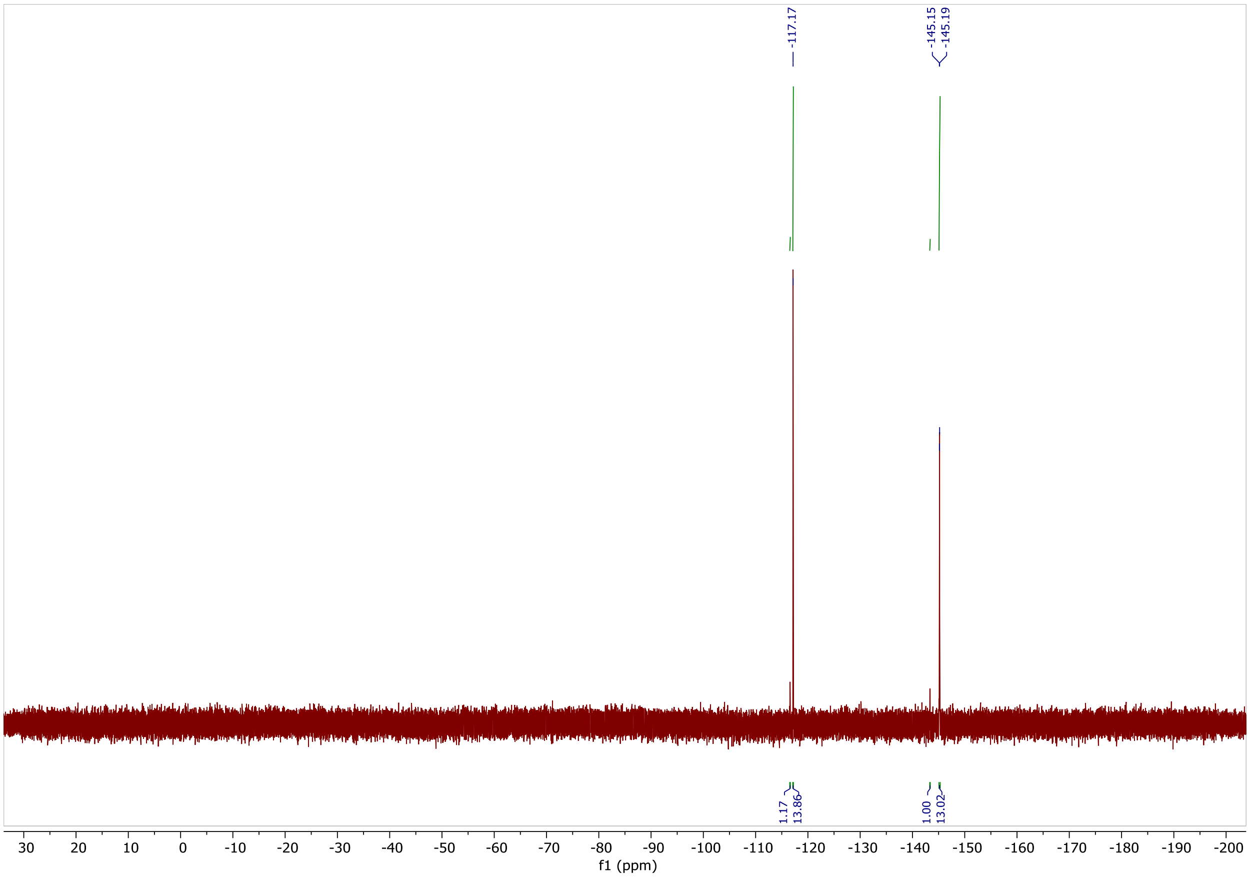

Figure S39. $^{13}C\{1H\}$ NMR (100 MHz) Spectrum of compound **14** (*dr* = 13:1) in $CDCl_3$.

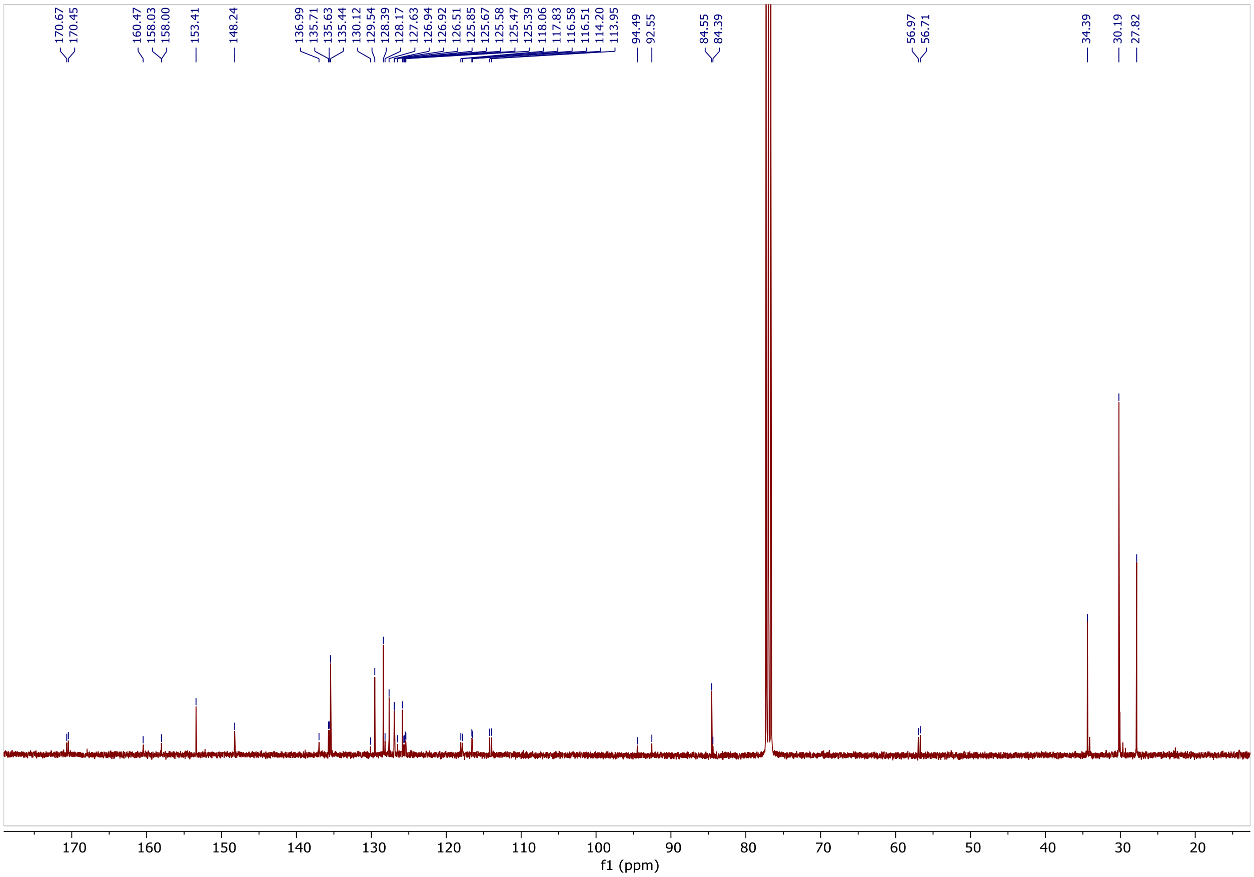

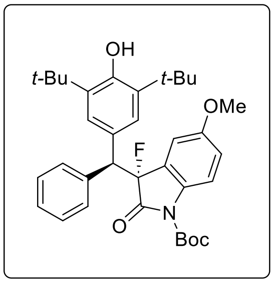

Figure S40. ^{1}H NMR (400 MHz) Spectrum of compound **15** (*dr* = 34:1) in $CDCl_3$.

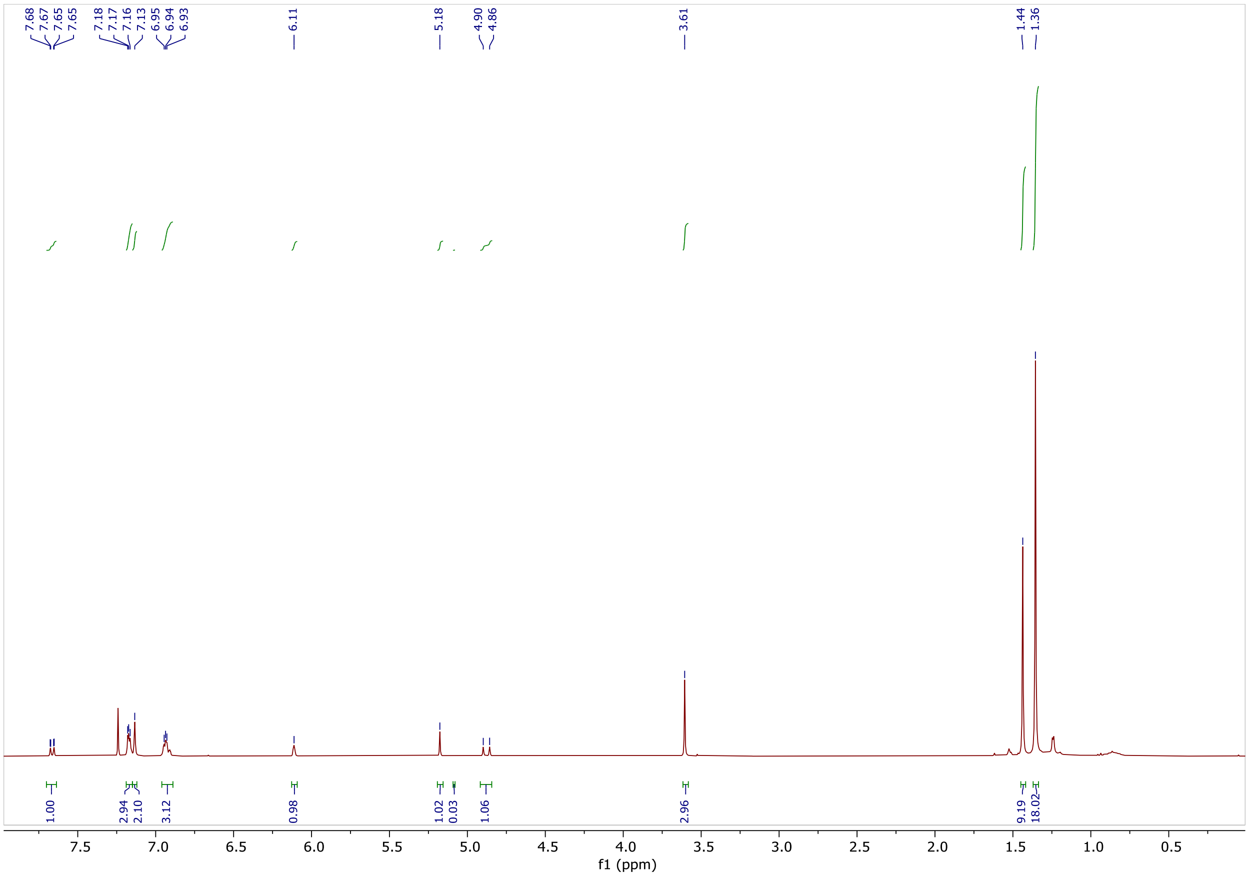

Figure S41. ^{19}F NMR (376 MHz) Spectrum of compound **15** (*dr* = 34:1) in $CDCl_3$.

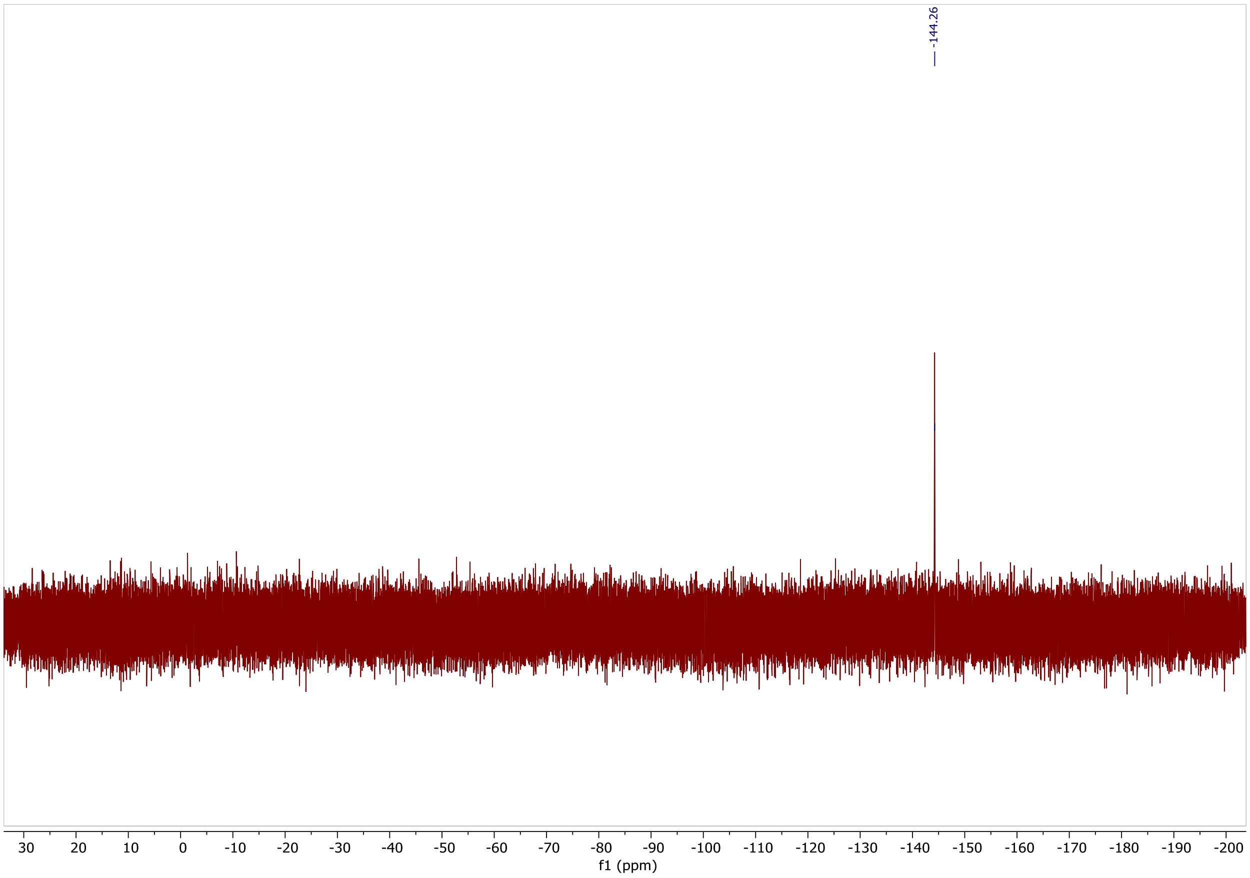

Figure S42. $^{13}C\{1H\}$ NMR (100 MHz) Spectrum of compound **15** (*dr* = 34:1) in $CDCl_3$.

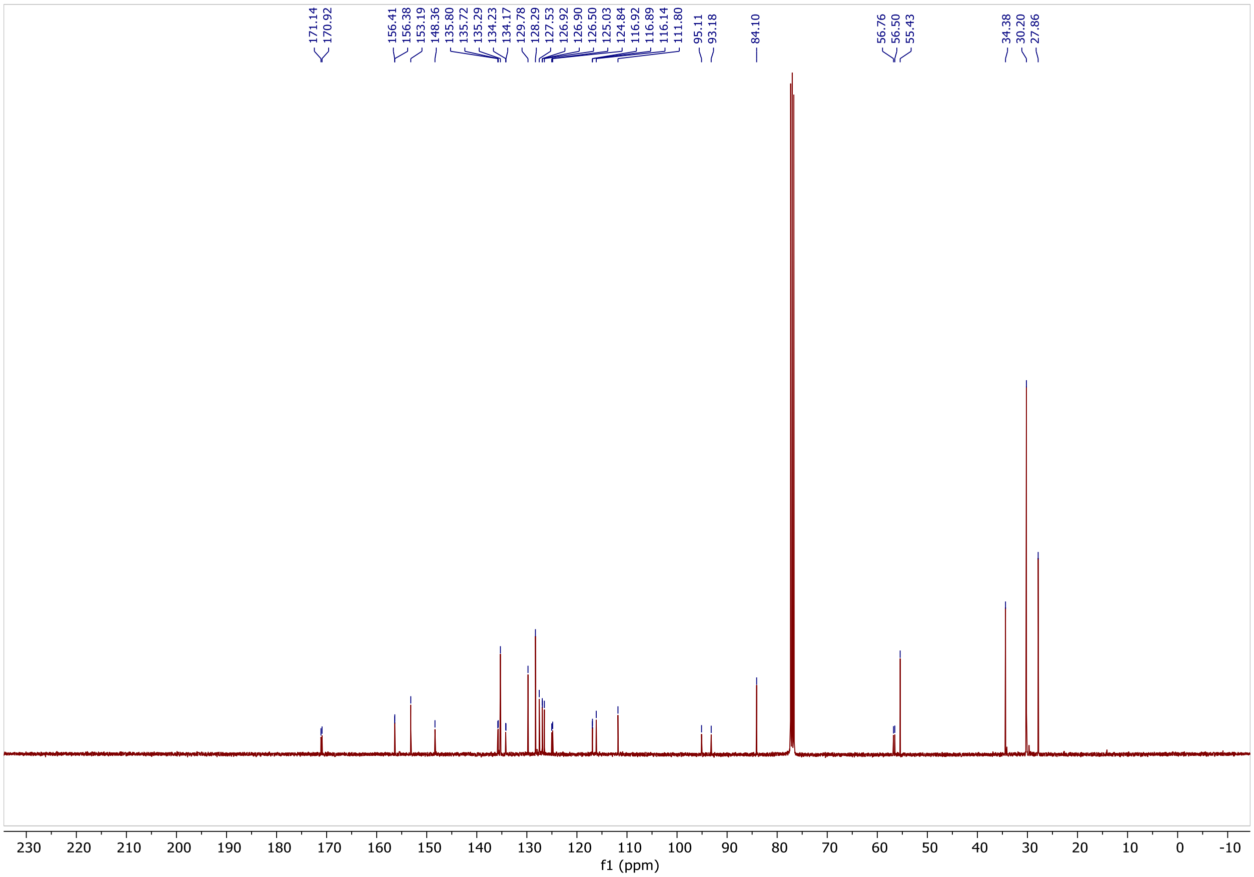

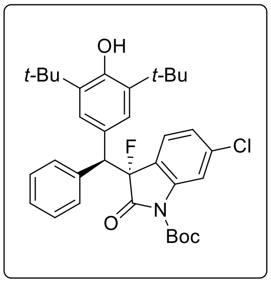

Figure S43. ^{1}H NMR (400 MHz) Spectrum of compound **16** (*dr* = 24:1) in $CDCl_3$.

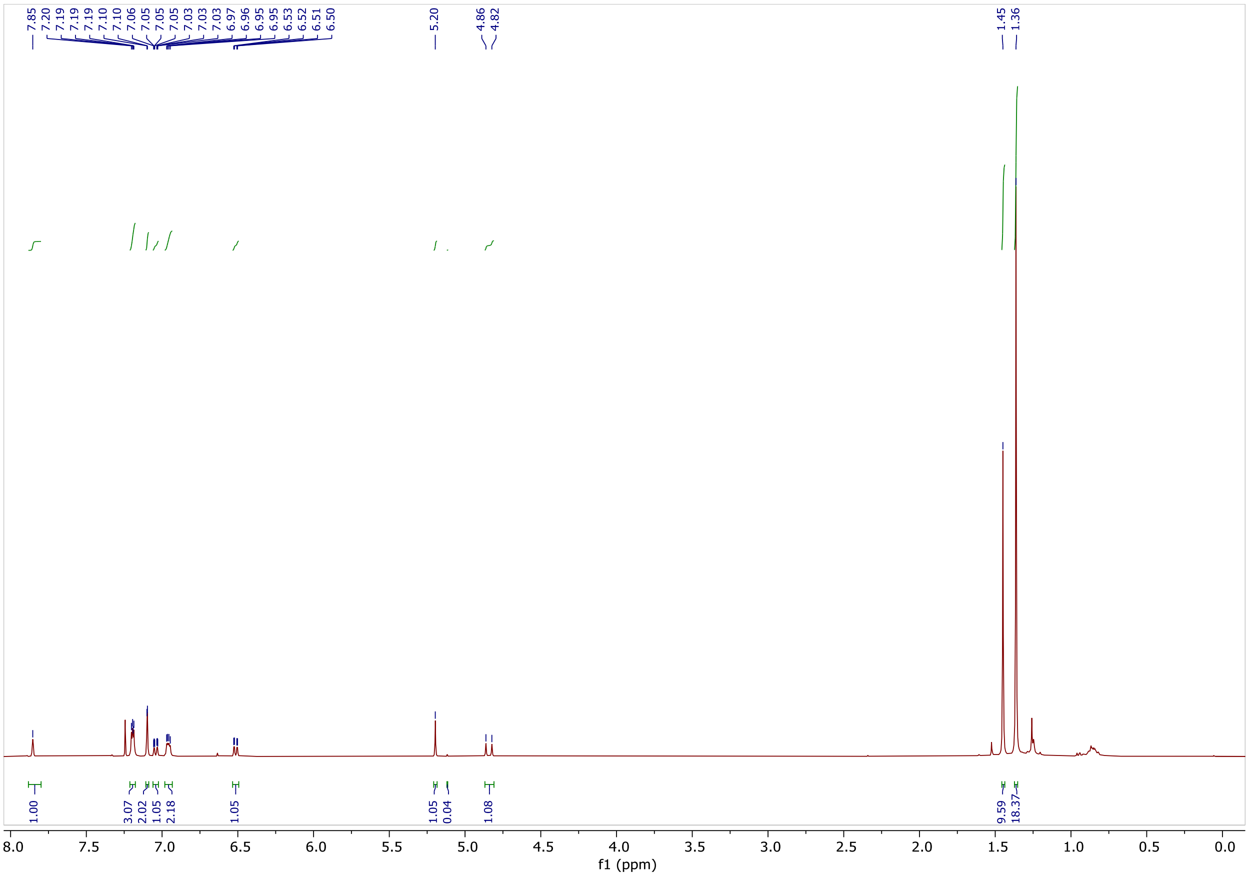

Figure S44. ^{19}F NMR (376 MHz) Spectrum of compound **16** (*dr* = 24:1) in $CDCl_3$.

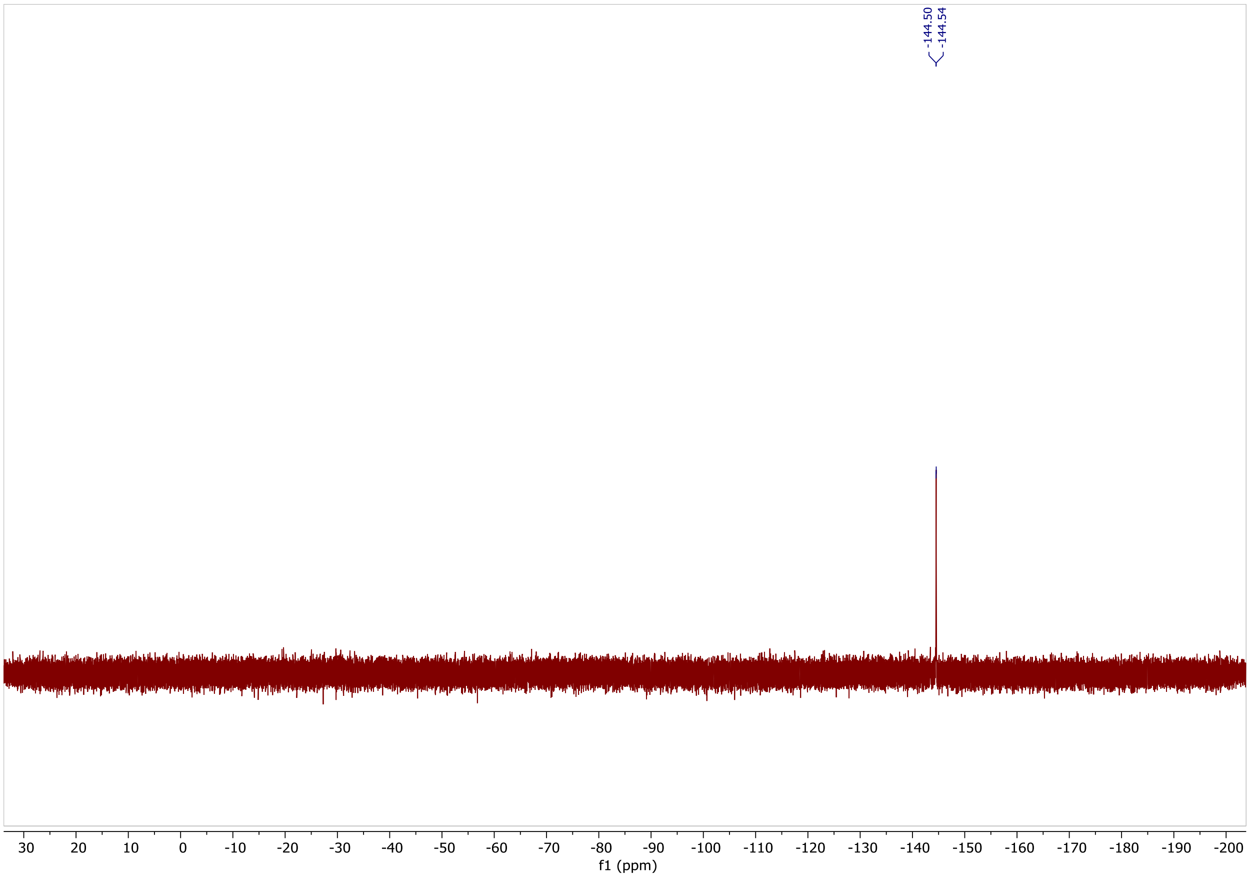

Figure S45. $^{13}C\{1H\}$ NMR (100 MHz) Spectrum of compound **16** (*dr* = 24:1) in $CDCl_3$.

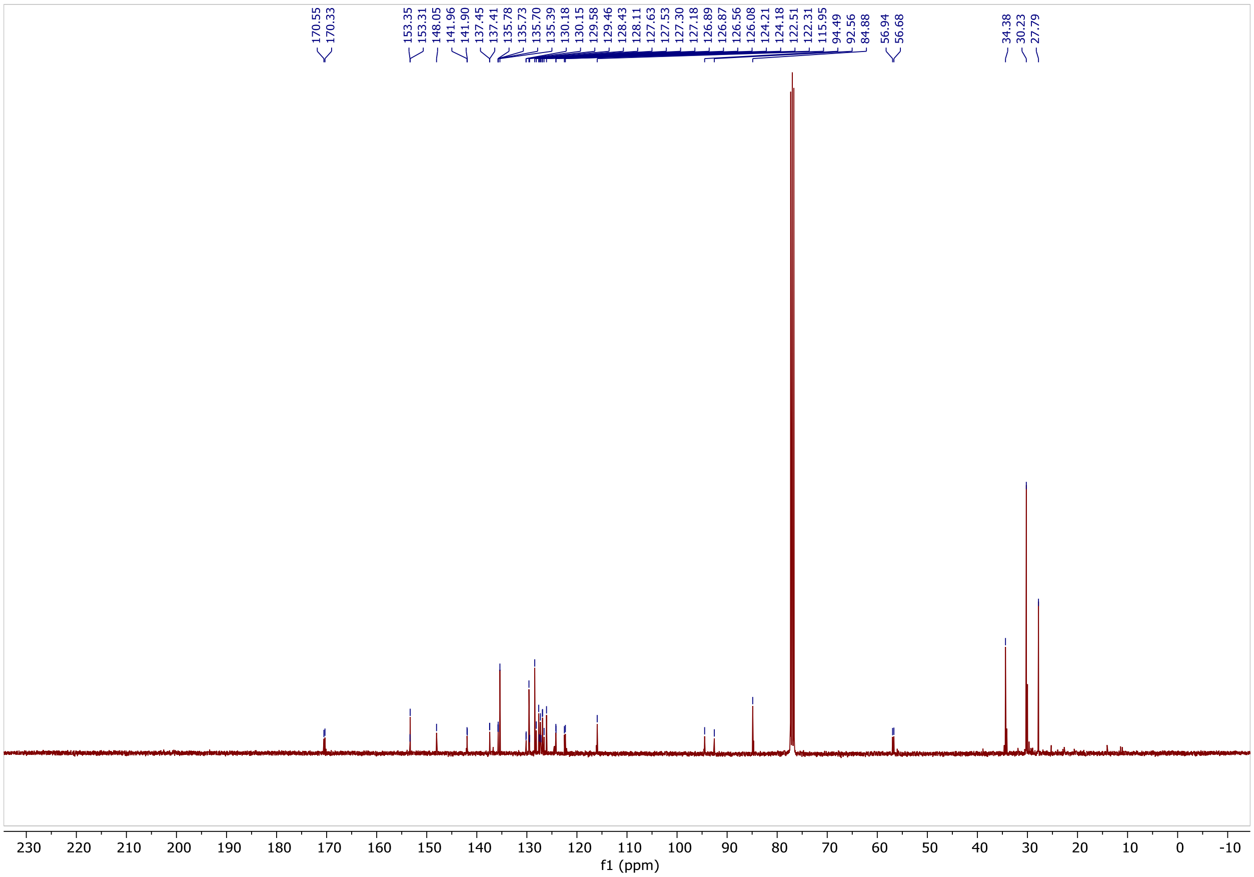

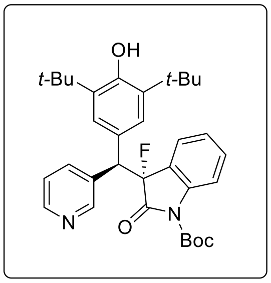

Figure S46. ^{1}H NMR (400 MHz) Spectrum of compound **17** (*dr* = 19:1) in $CDCl_3$.

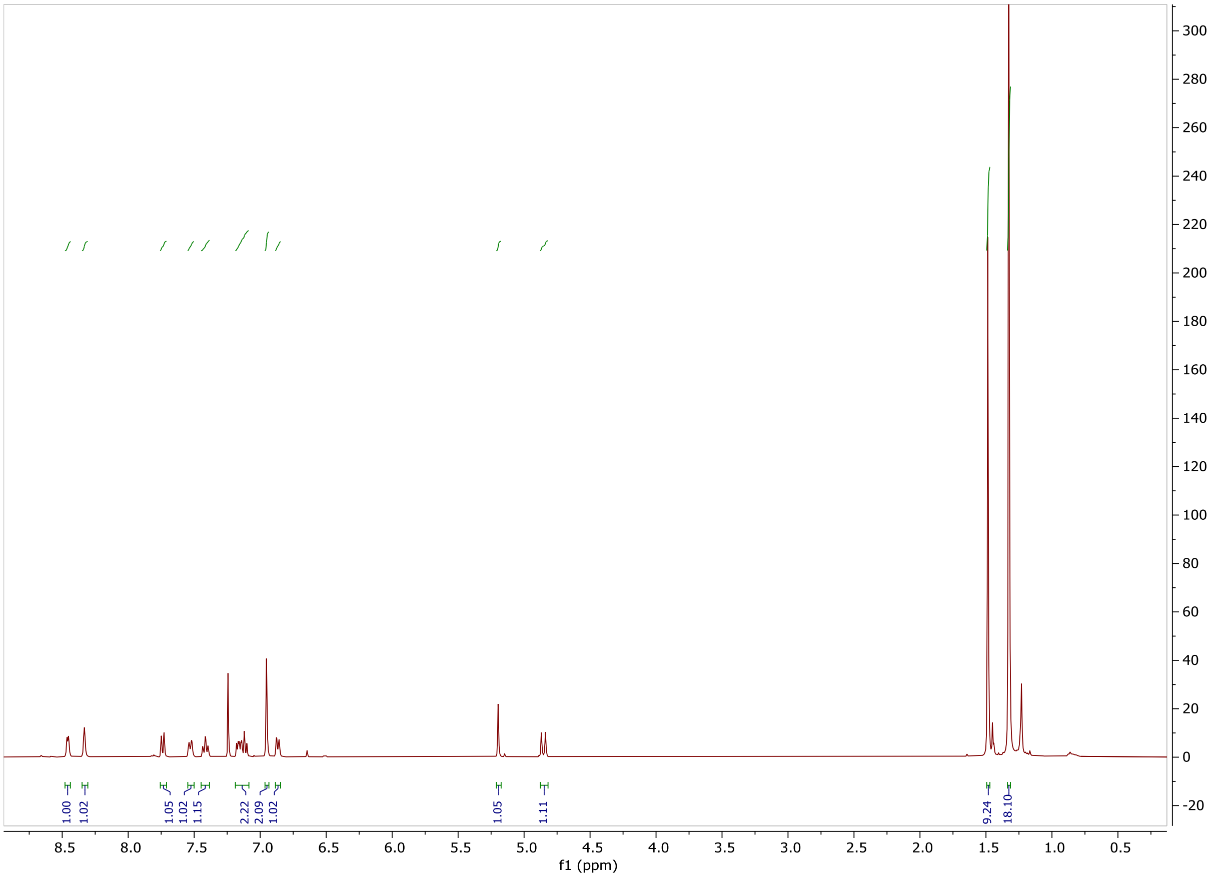

Figure S47. ^{19}F NMR (376 MHz) Spectrum of compound **17** (*dr* = 19:1) in $CDCl_3$.

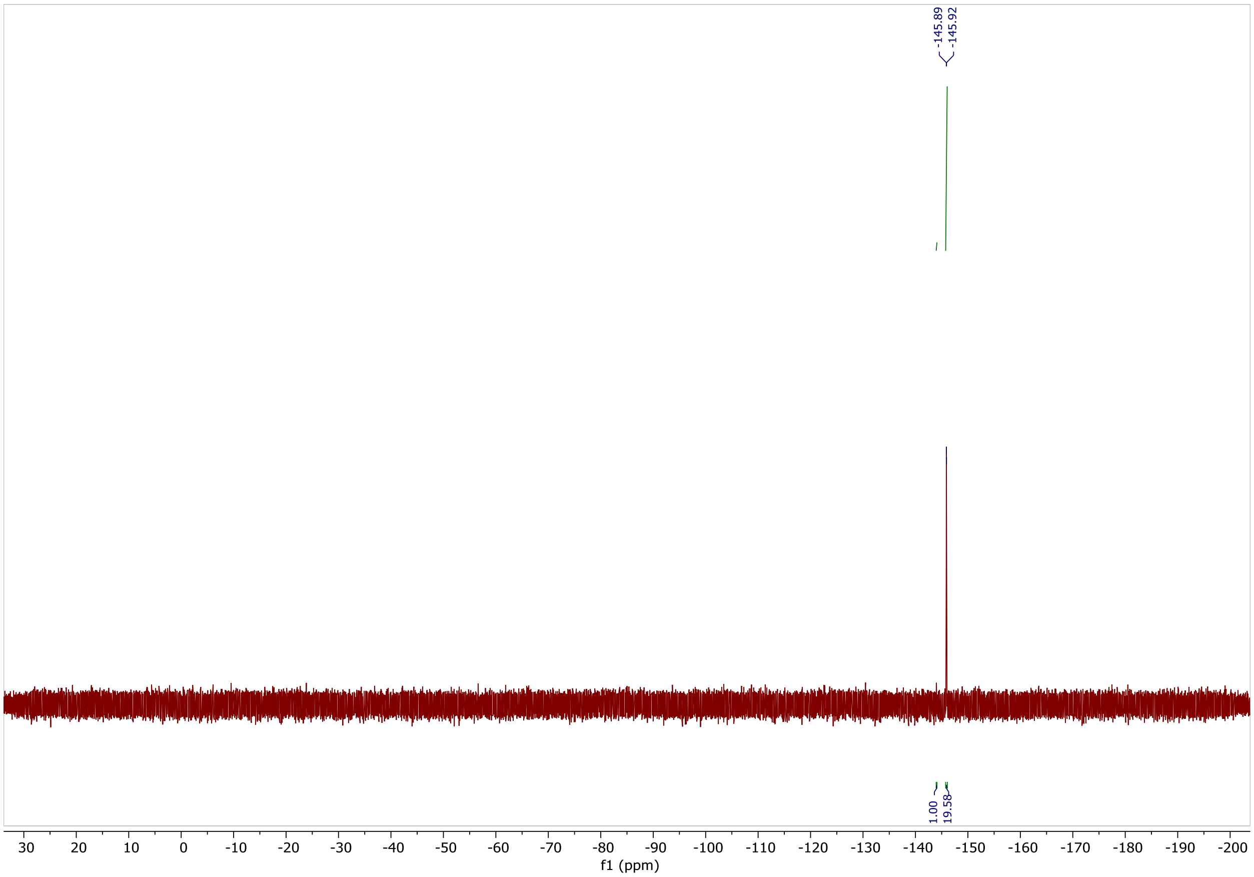

Figure S48. $^{13}C\{1H\}$ NMR (100 MHz) Spectrum of compound **17** (*dr* = 19:1) in $CDCl_3$.

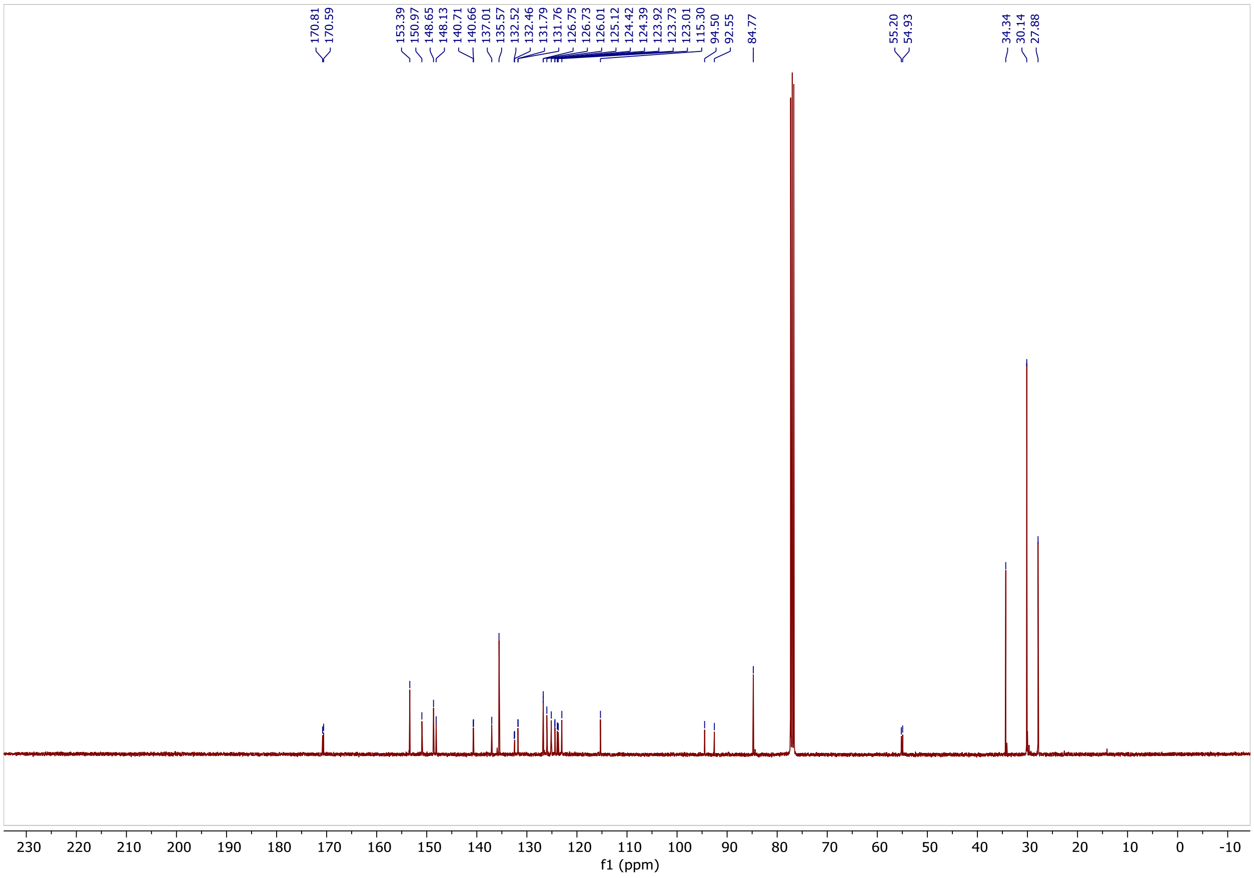

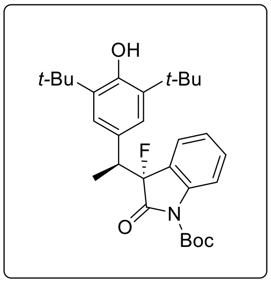

Figure S49. ^{1}H NMR (400 MHz) Spectrum of compound **18** (*dr* = 3:1) in $CDCl_3$.

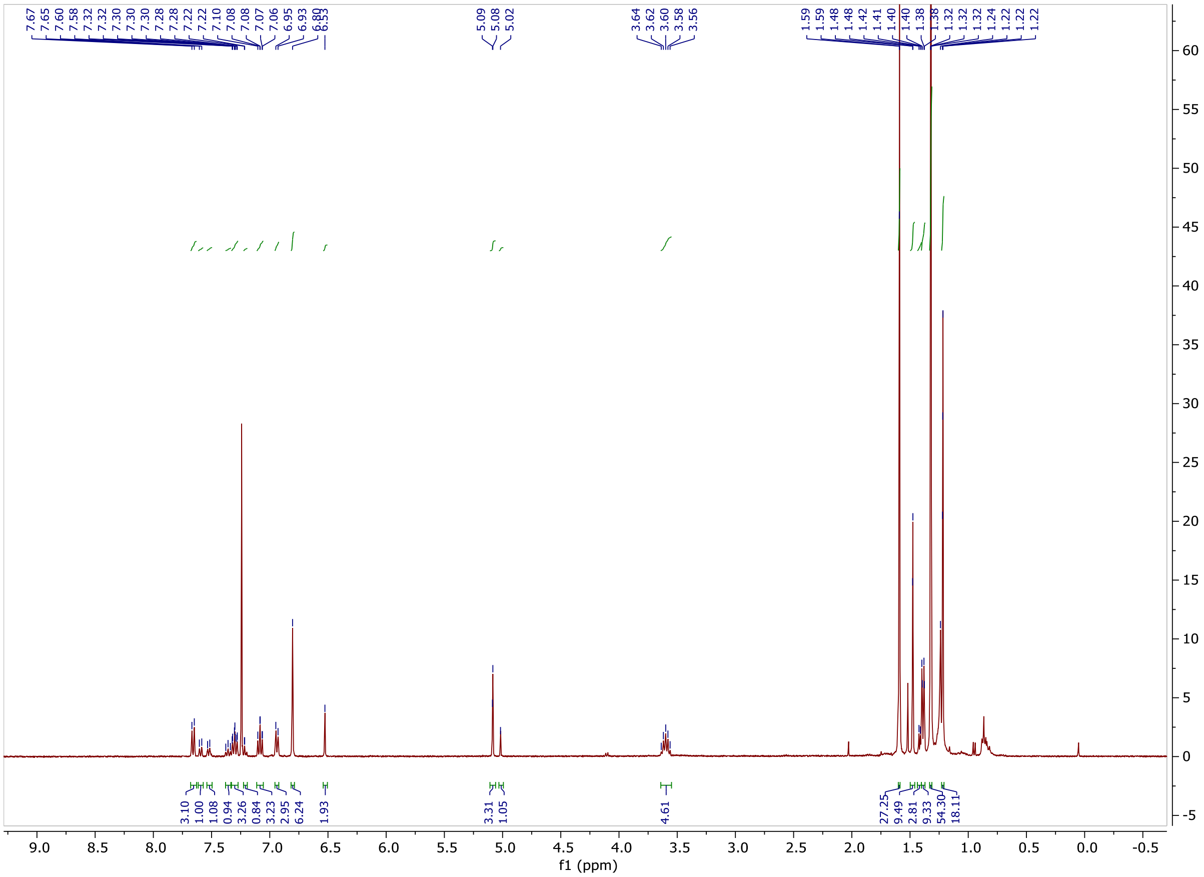

Figure S50. ^{19}F NMR (376 MHz) Spectrum of compound **18** (*dr* = 3:1) in $CDCl_3$.

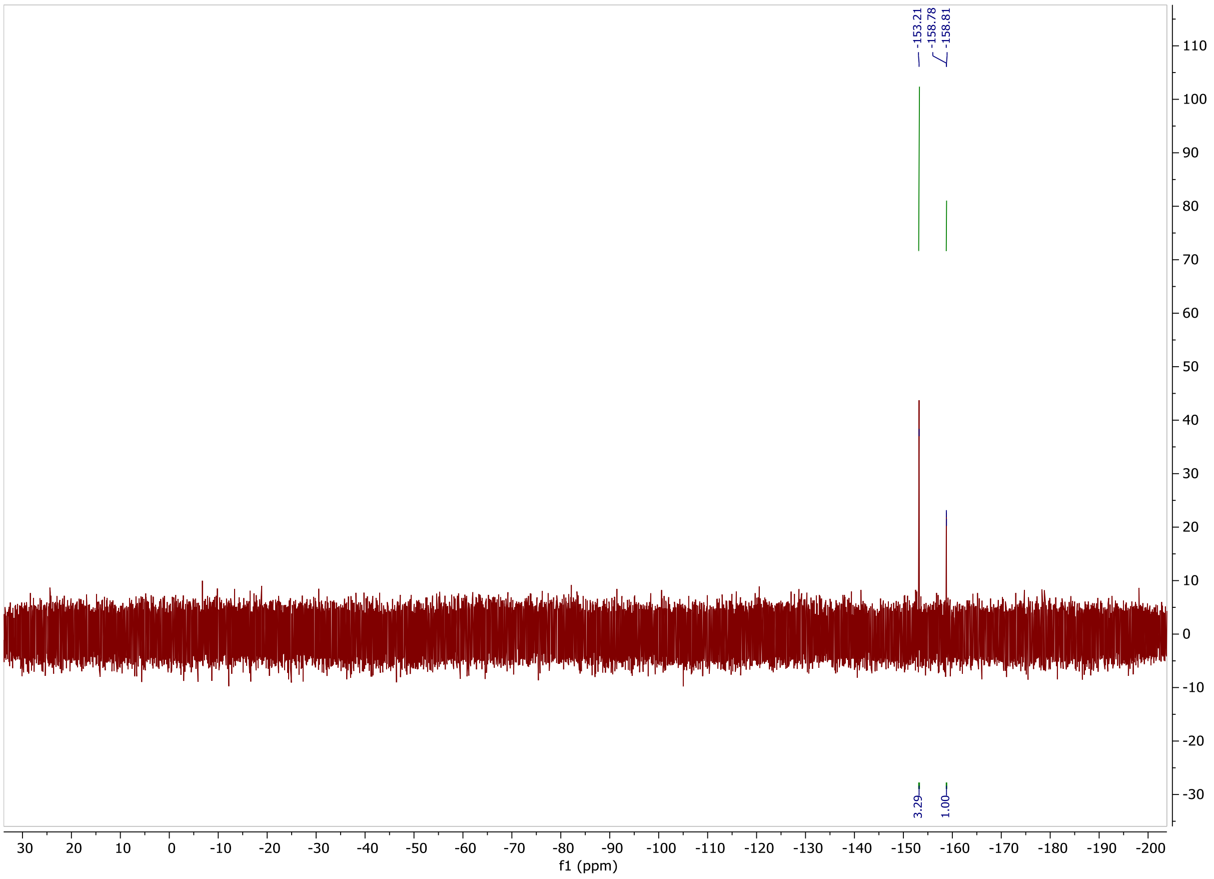

Figure S51. $^{13}C\{1H\}$ NMR (100 MHz) Spectrum of compound **18** (*dr* = 3:1) in $CDCl_3$.

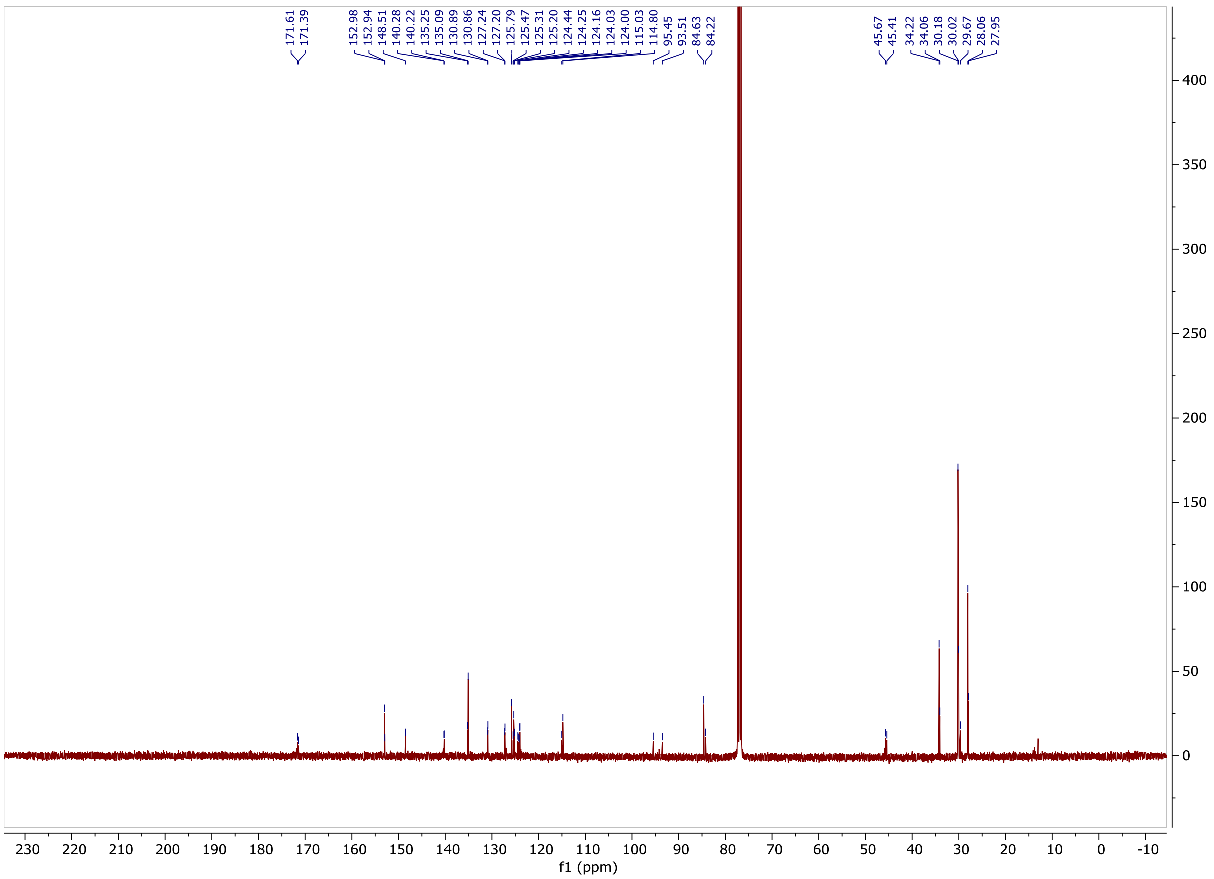

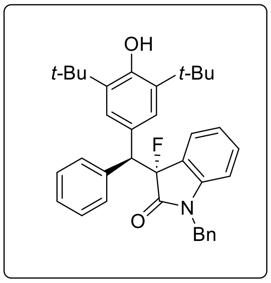

Figure S52. ^{1}H NMR (400 MHz) Spectrum of compound **19** (*dr* = 1.3:1) in $CDCl_3$.

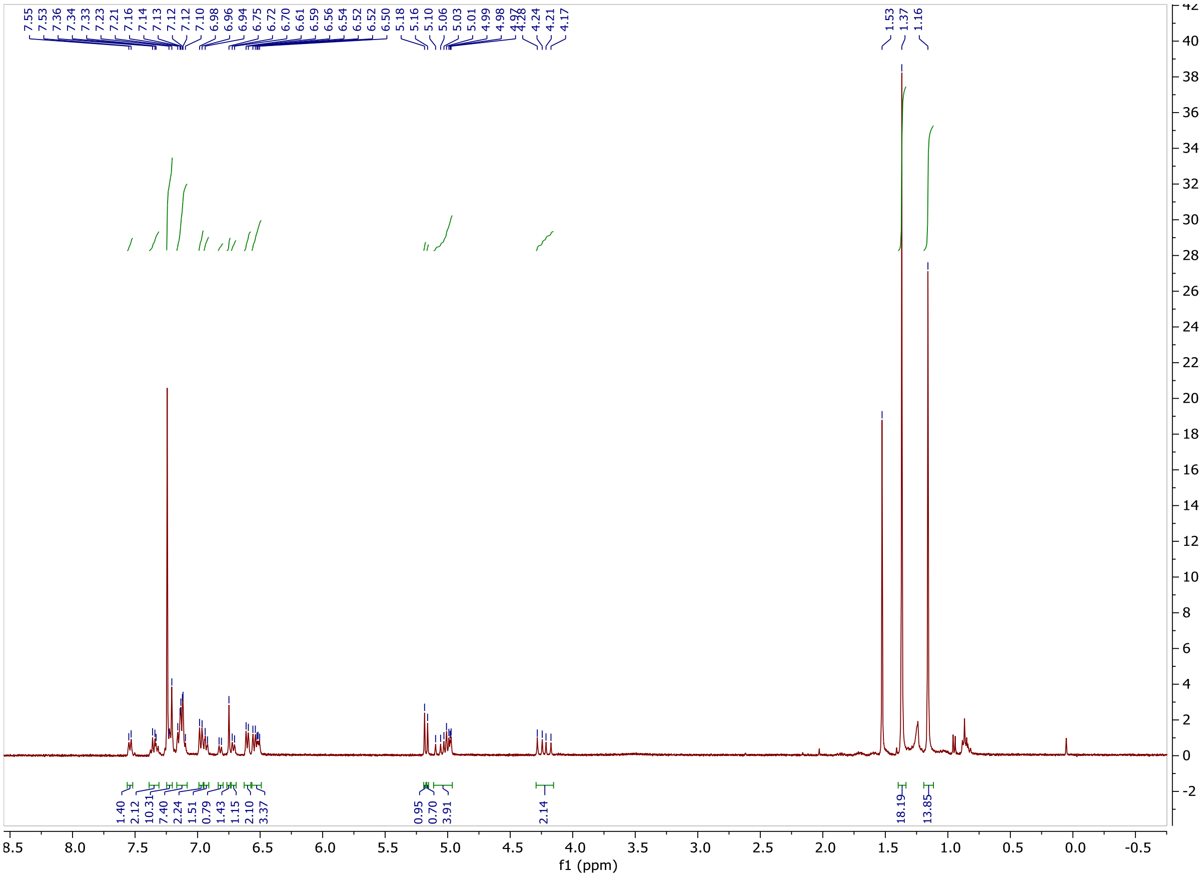

Figure S53. ^{19}F NMR (376 MHz) Spectrum of compound **19** (*dr* = 1.3:1) in $CDCl_3$.

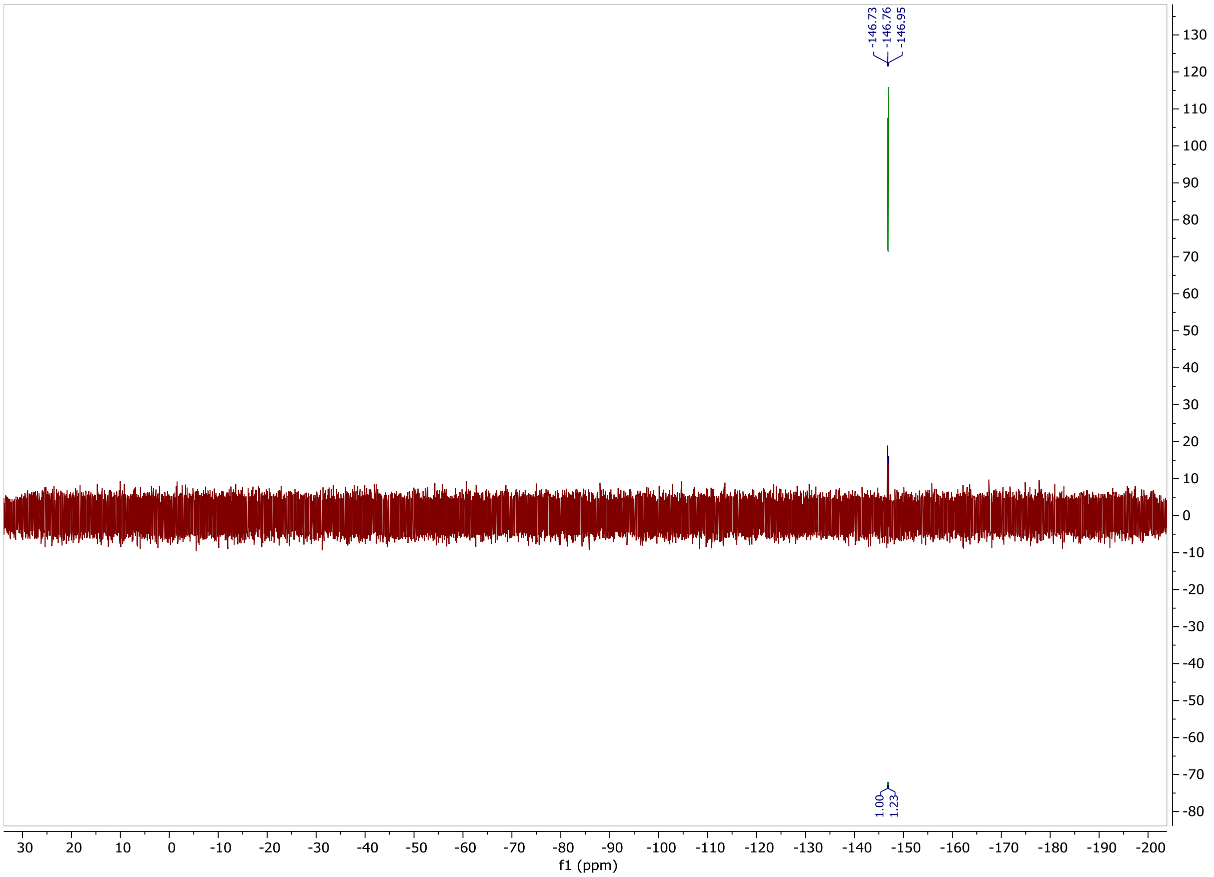

Figure S54. $^{13}C\{1H\}$ NMR (100 MHz) Spectrum of compound **19** (*dr* = 1.3:1) in $CDCl_3$.

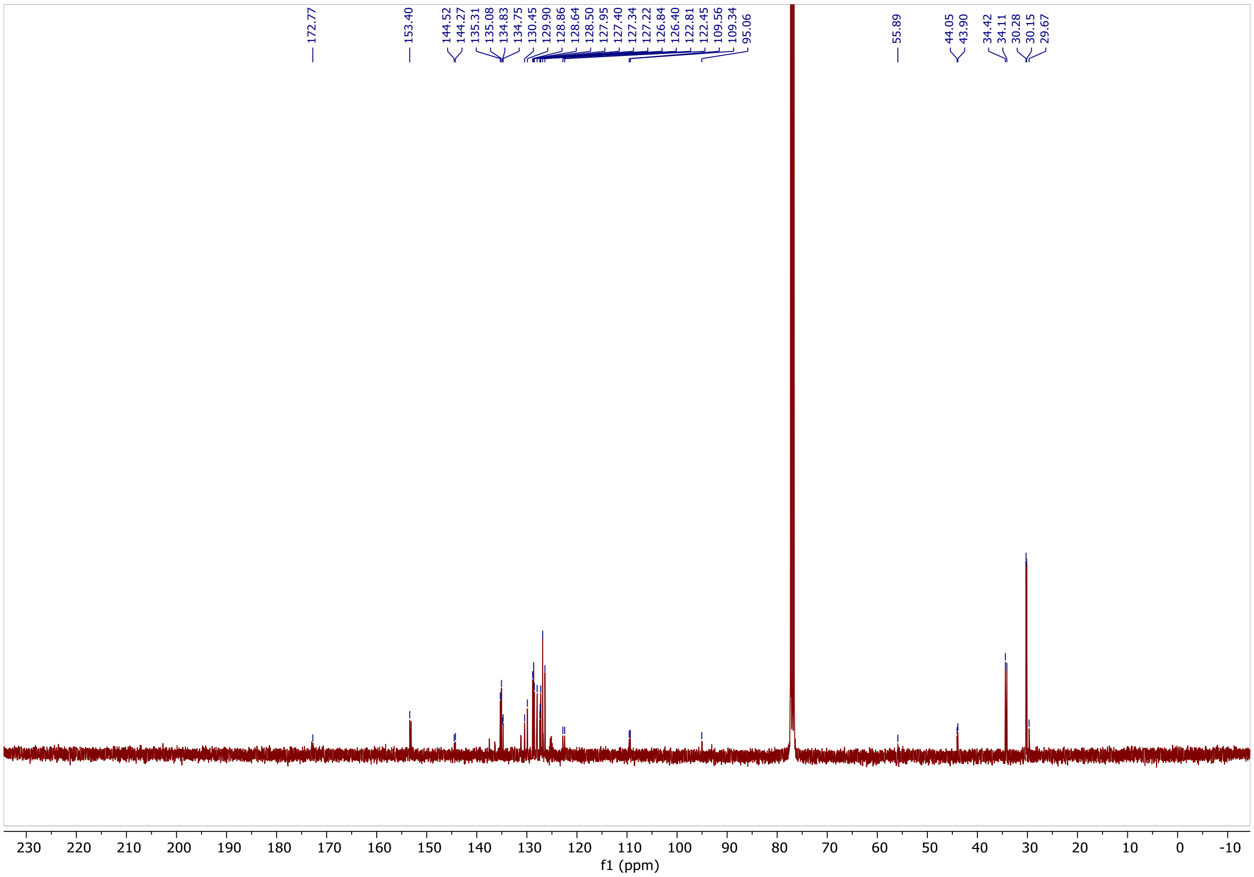

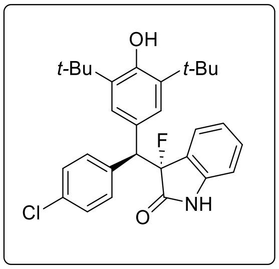

Figure S55. ^{1}H NMR (400 MHz) Spectrum of compound **21** (*dr* = 48:1) in $CDCl_3$.

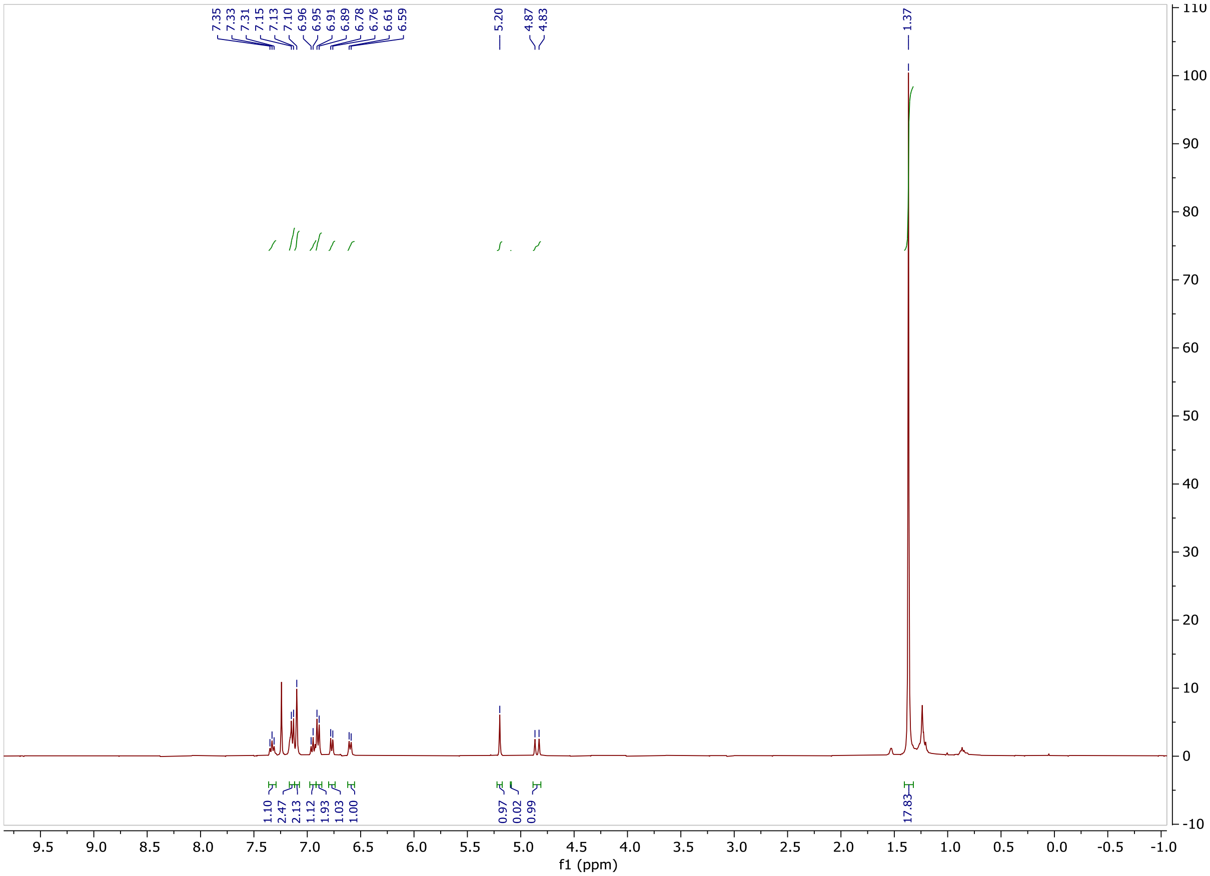

Figure S56. ^{19}F NMR (376 MHz) Spectrum of compound **21** (*dr* = 48:1) in $CDCl_3$.

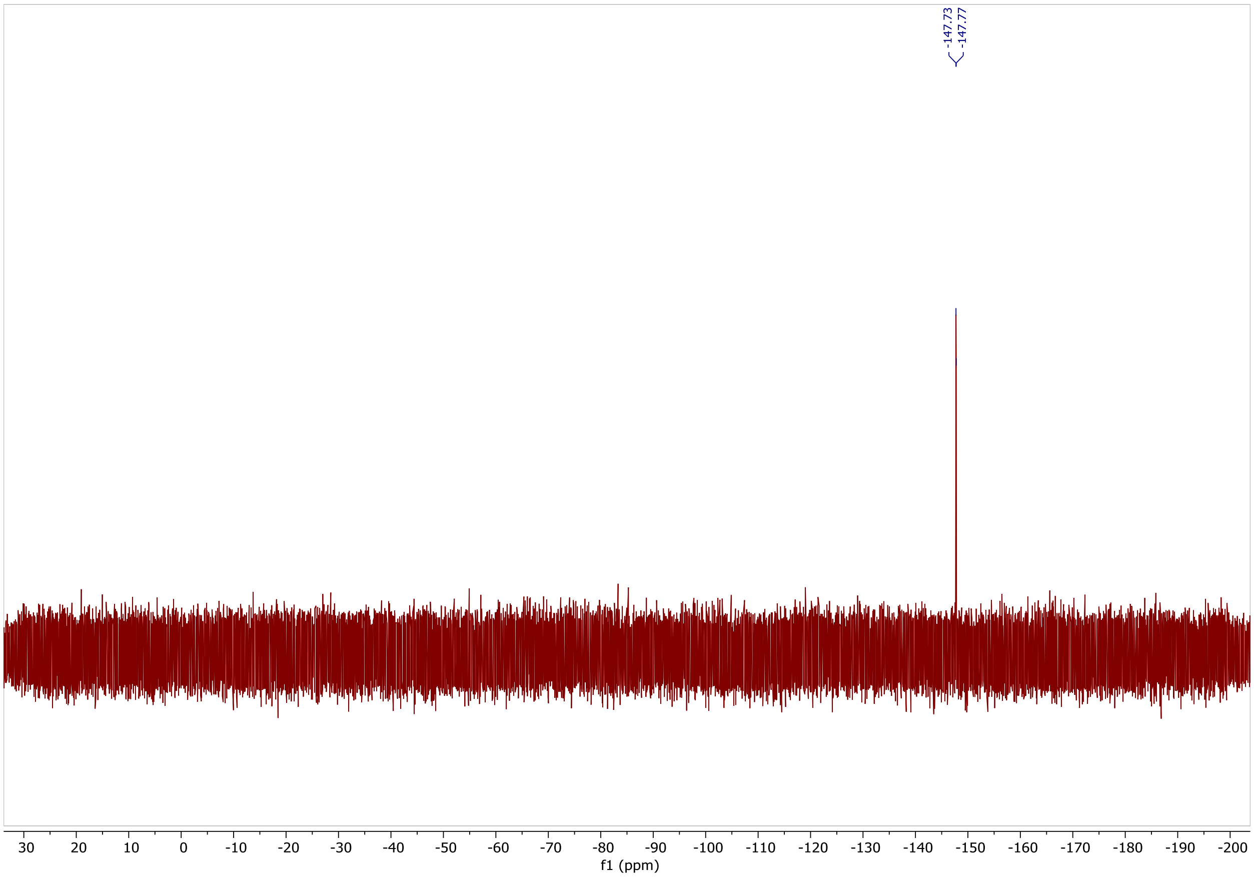

Figure S57. $^{13}C\{1H\}$ NMR (100 MHz) Spectrum of compound **21** (*dr* = 48:1) in $CDCl_3$.

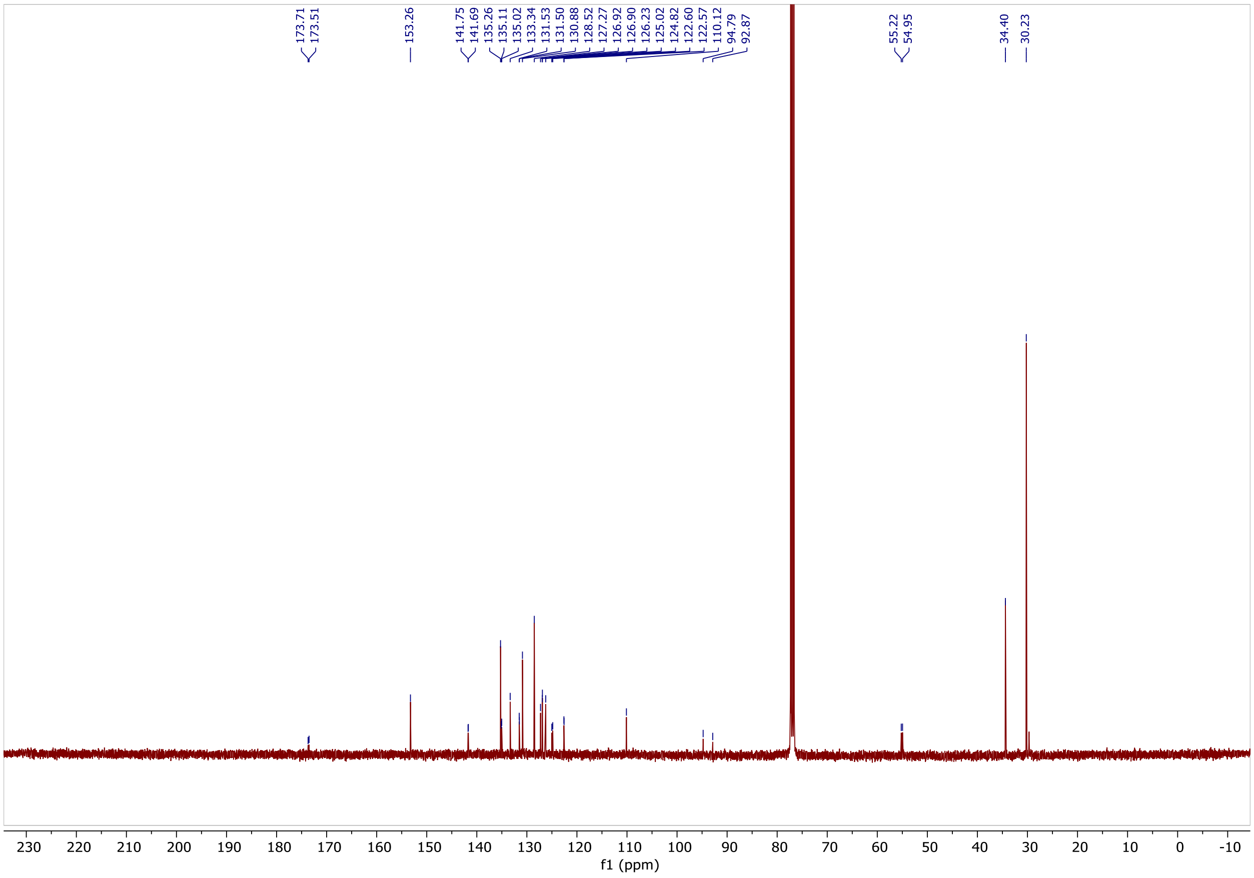

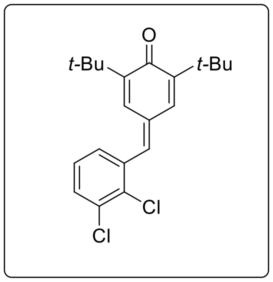

Figure S58. ^{1}H NMR (400 MHz) Spectrum of 2,6-di-*tert*-butyl-4-(2,3-dichlorobenzylidene)cyclohexa-2,5-dien-1-one in $CDCl_3$.

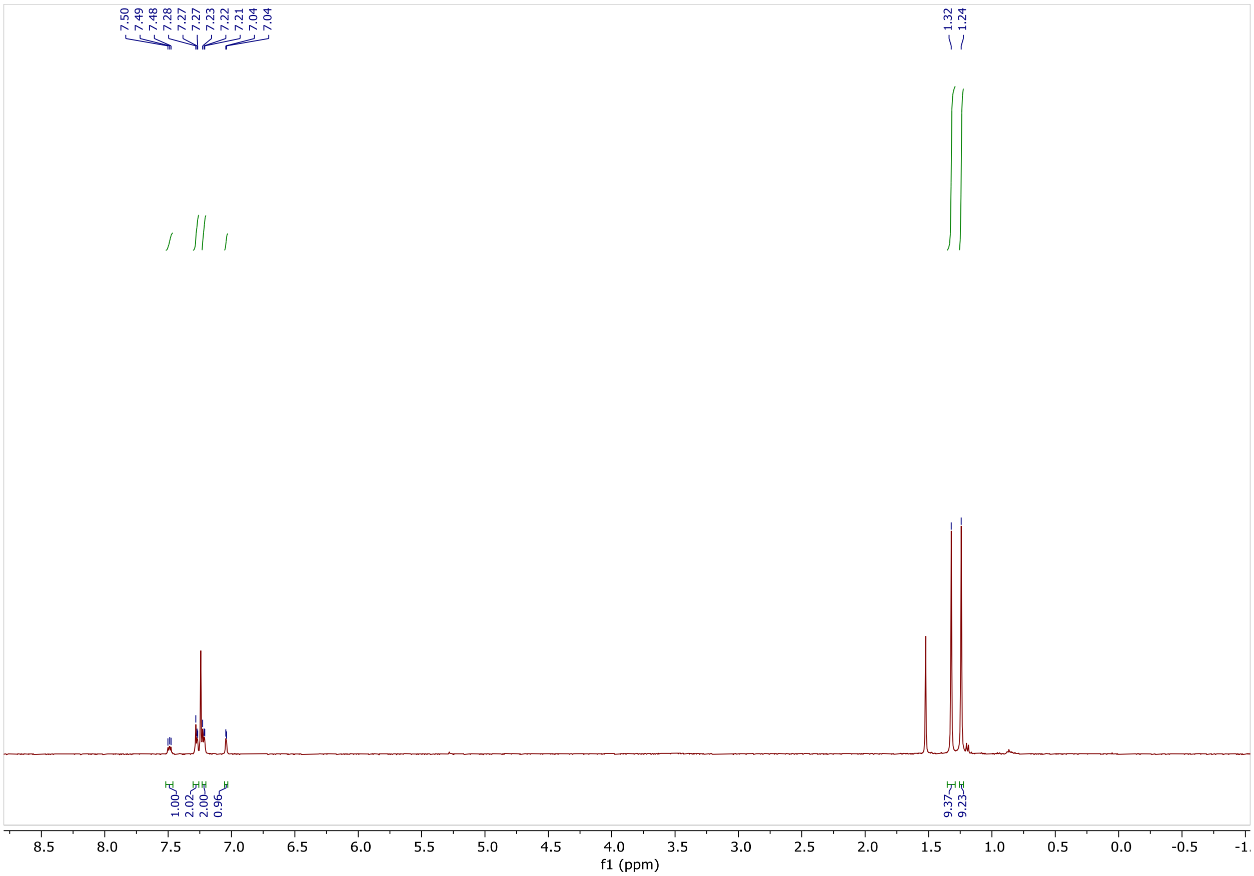

Figure S59. $^{13}C\{1H\}$ NMR (100 MHz) Spectrum of 2,6-di-*tert*-butyl-4-(2,3-dichlorobenzylidene)cyclohexa-2,5-dien-1-one in $CDCl_3$.

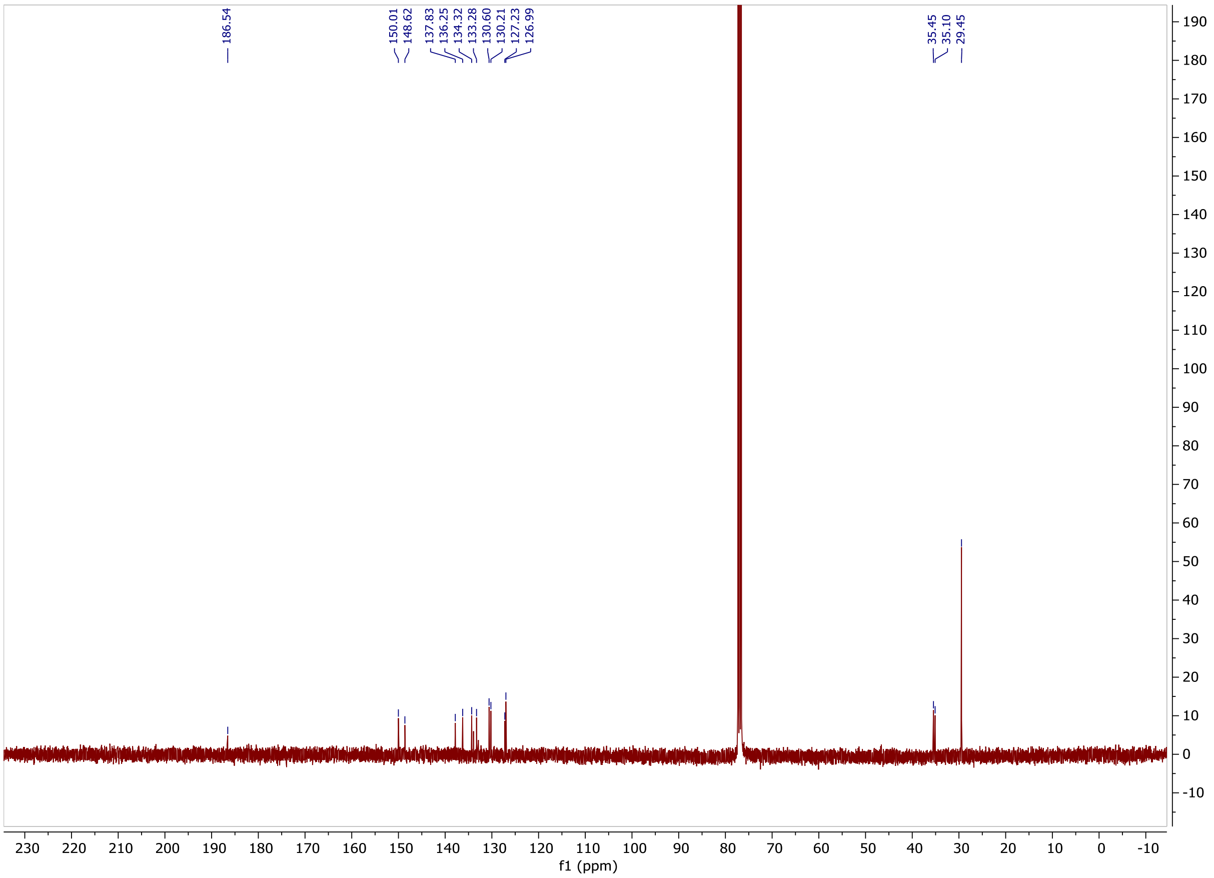

7. ^{1}H NMR spectra of known compounds

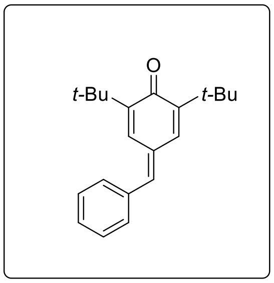

Figure S60. ^{1}H NMR (400 MHz) Spectrum of 4-benzylidene-2,6-di-*tert*-butylcyclohexa-2,5-dien-1-one.

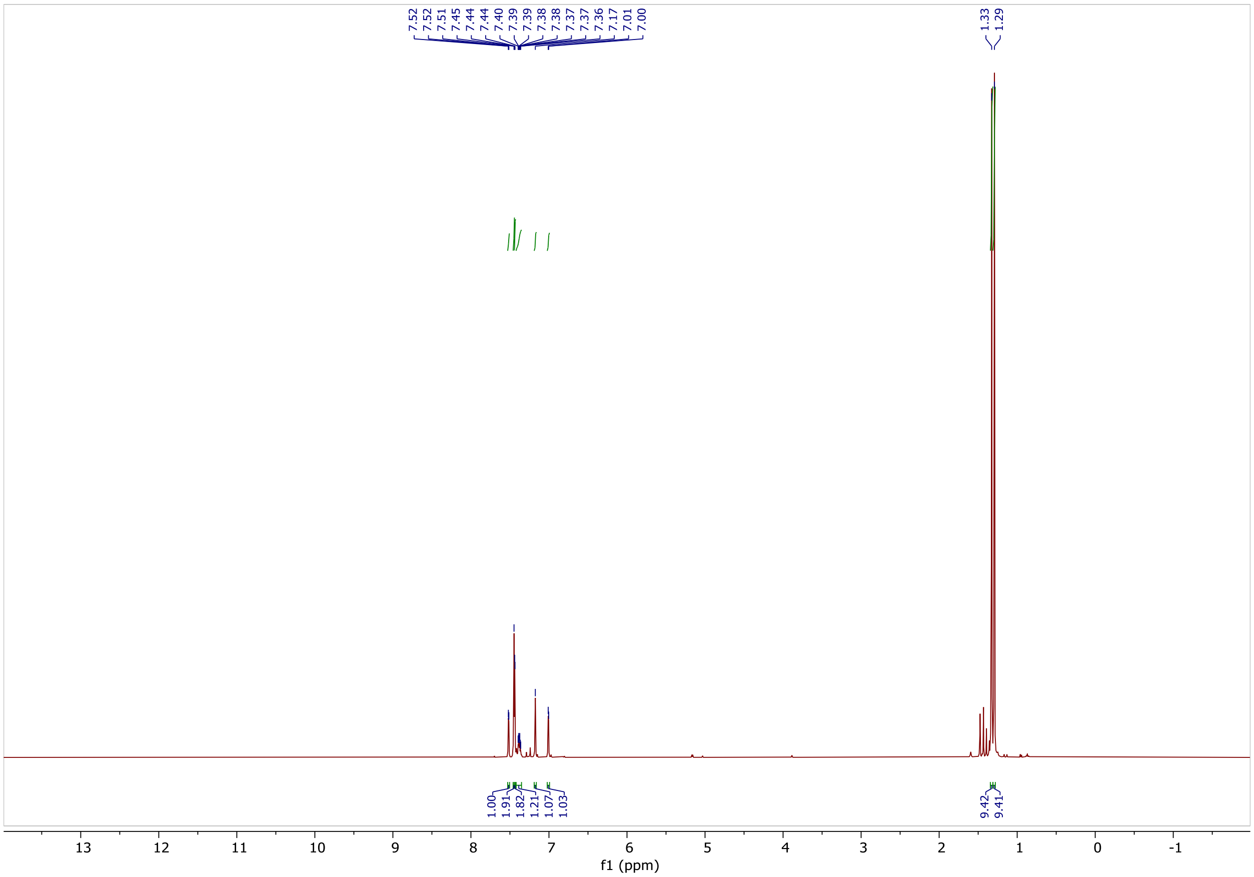

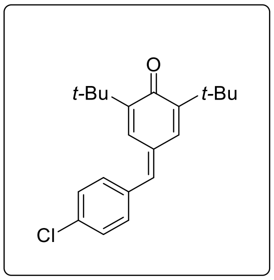

Figure S61. ^{1}H NMR (400 MHz) Spectrum of 2,6-di-*tert*-butyl-4-(4-chlorobenzylidene)cyclohexa-2,5-dien-1-one.

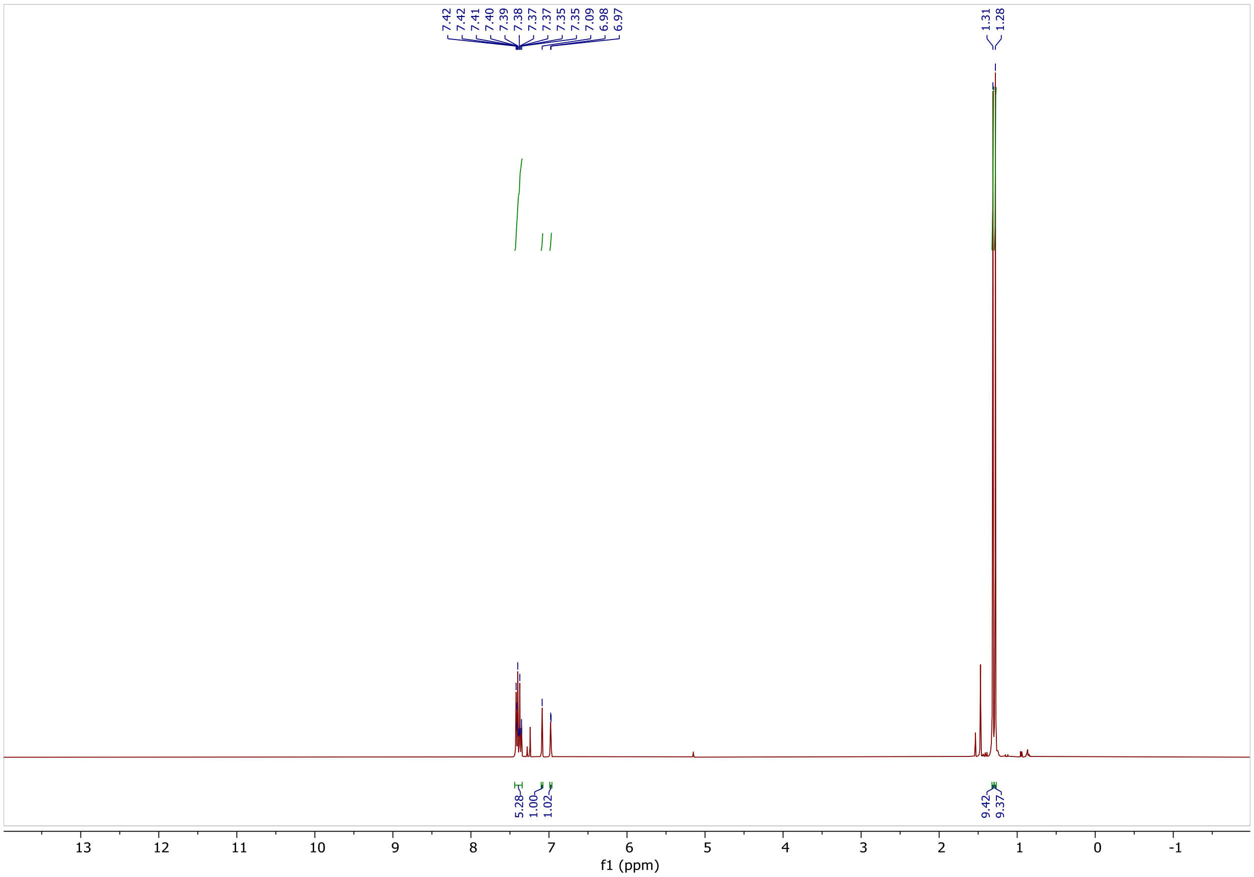

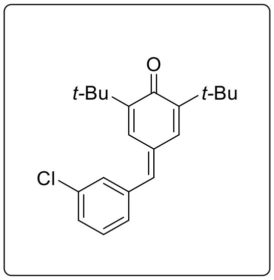

Figure S62. ^{1}H NMR (400 MHz) Spectrum of 2,6-di-*tert*-butyl-4-(3-chlorobenzylidene)cyclohexa-2,5-dien-1-one.

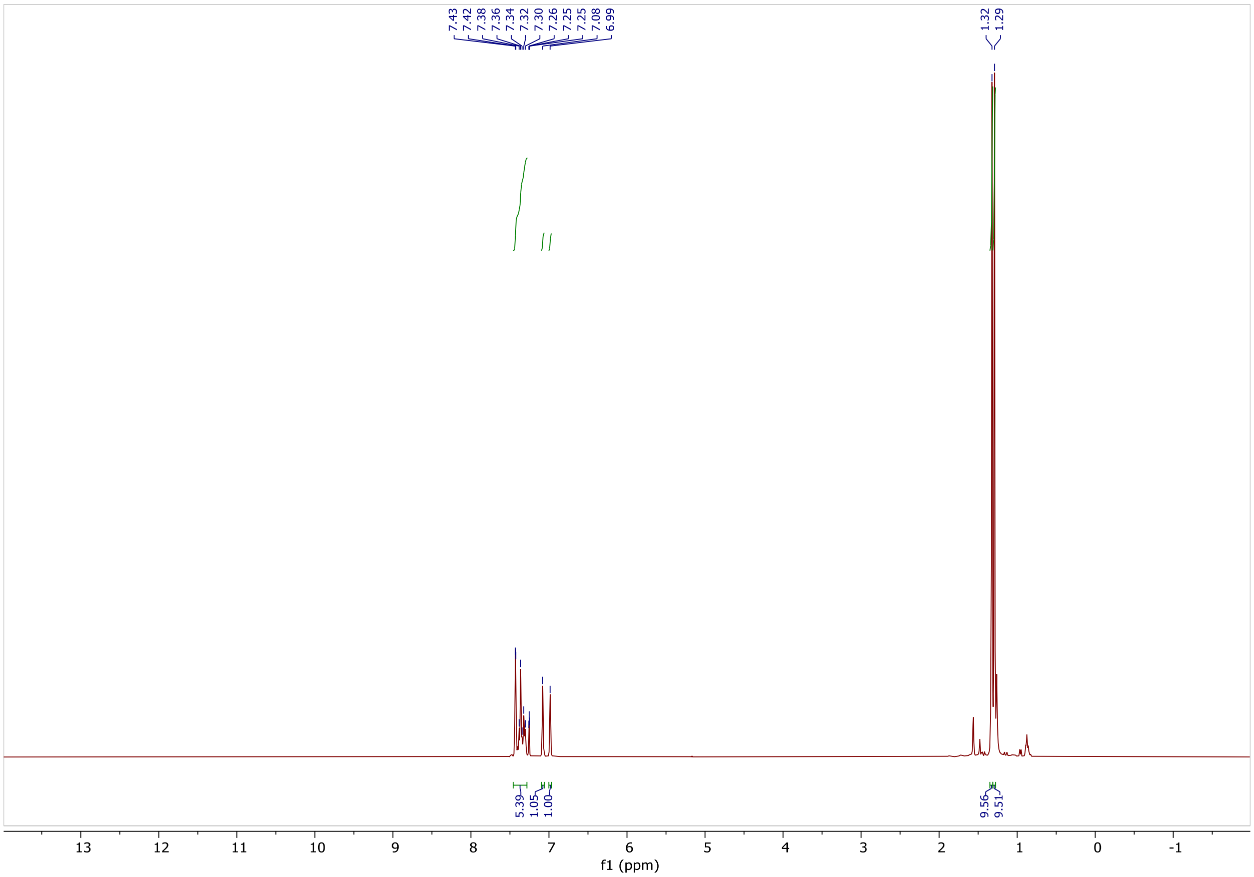

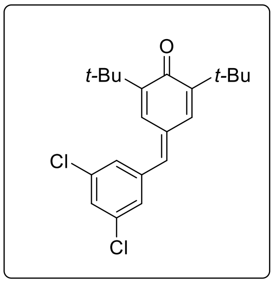

Figure S63. ^{1}H NMR (400 MHz) Spectrum of 2,6-di-*tert*-butyl-4-(3,5-dichlorobenzylidene)cyclohexa-2,5-dien-1-one.

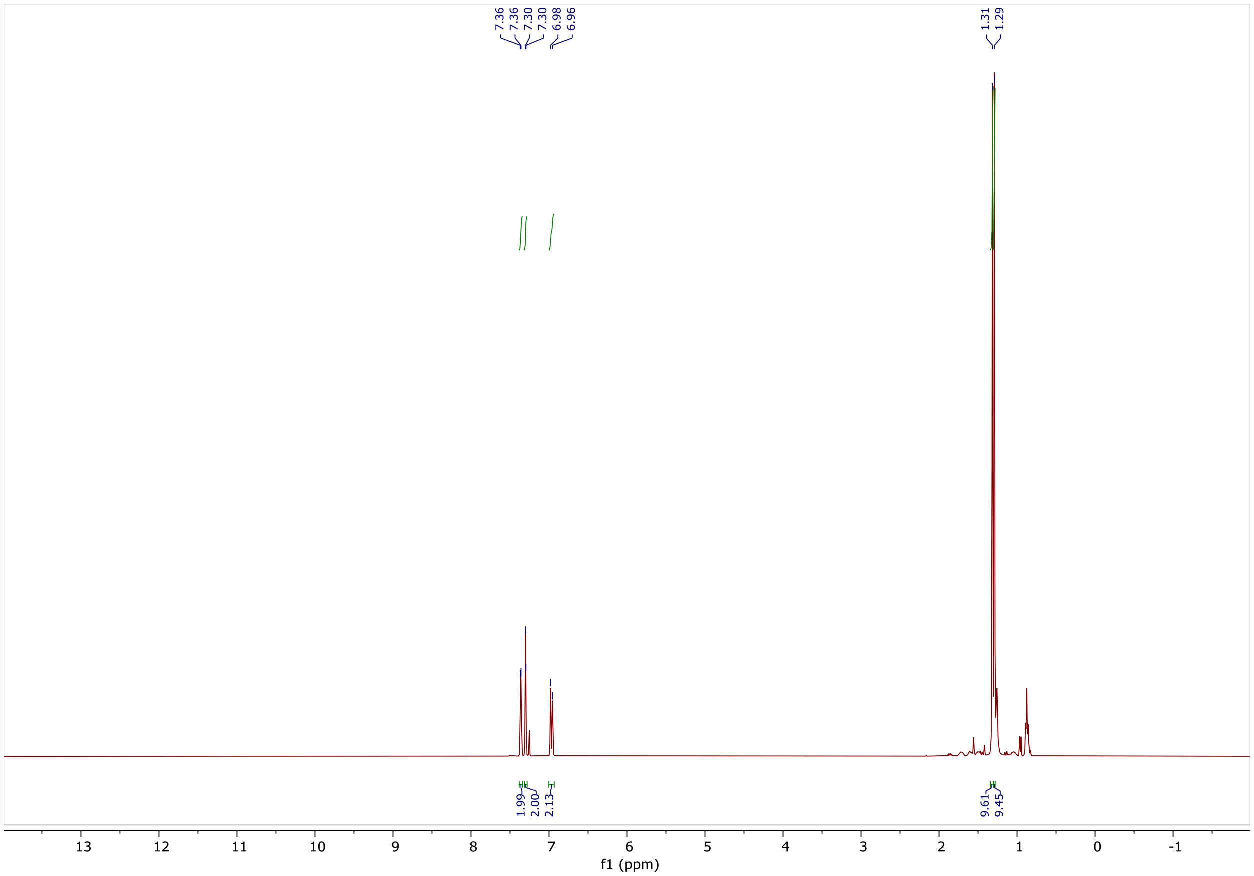

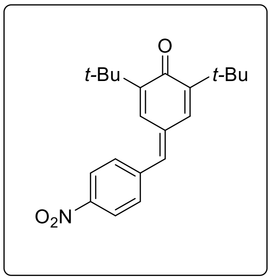

Figure S64. ^{1}H NMR (400 MHz) Spectrum of 2,6-di-*tert*-butyl-4-(4-nitrobenzylidene)cyclohexa-2,5-dien-1-one.

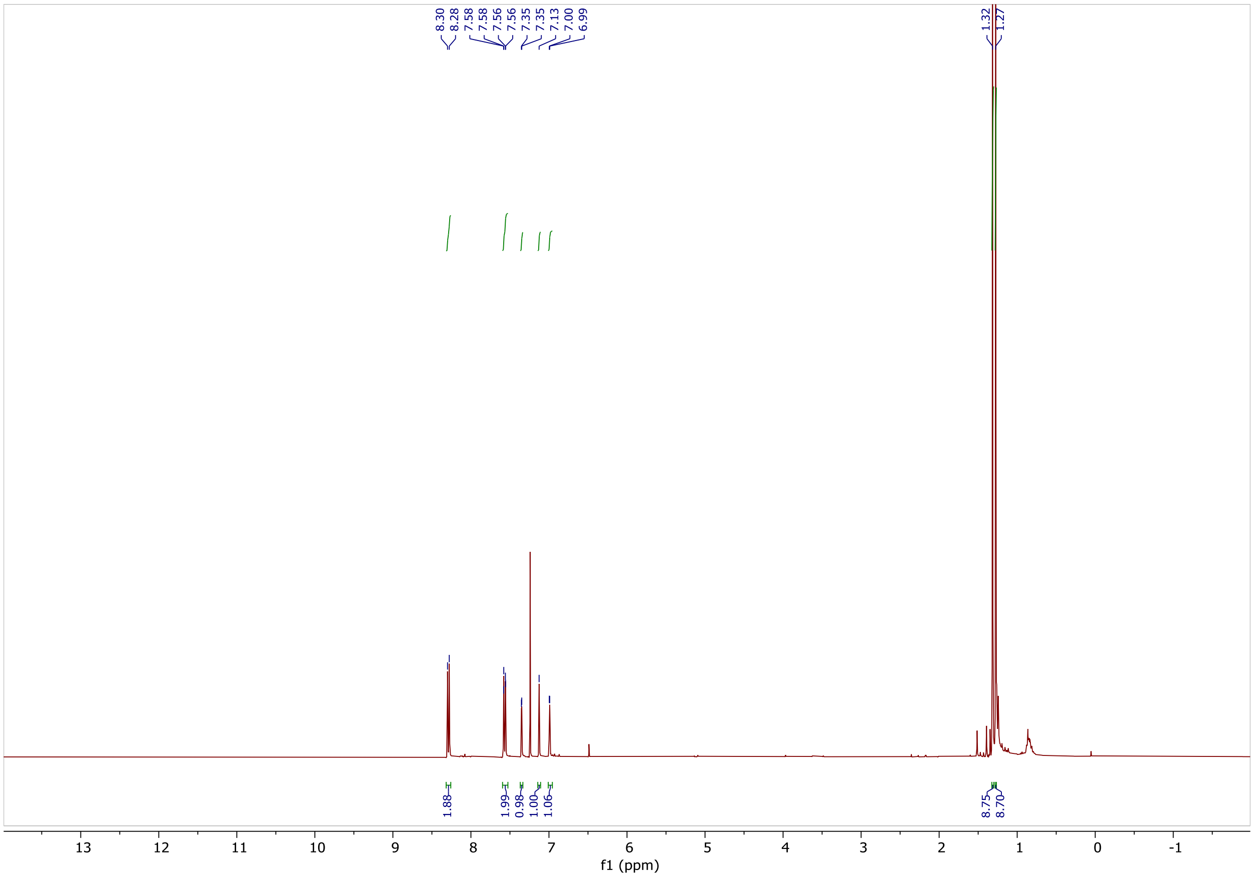

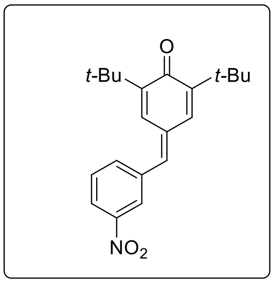

Figure S65. ^{1}H NMR (400 MHz) Spectrum of 2,6-di-*tert*-butyl-4-(3-nitrobenzylidene)cyclohexa-2,5-dien-1-one.

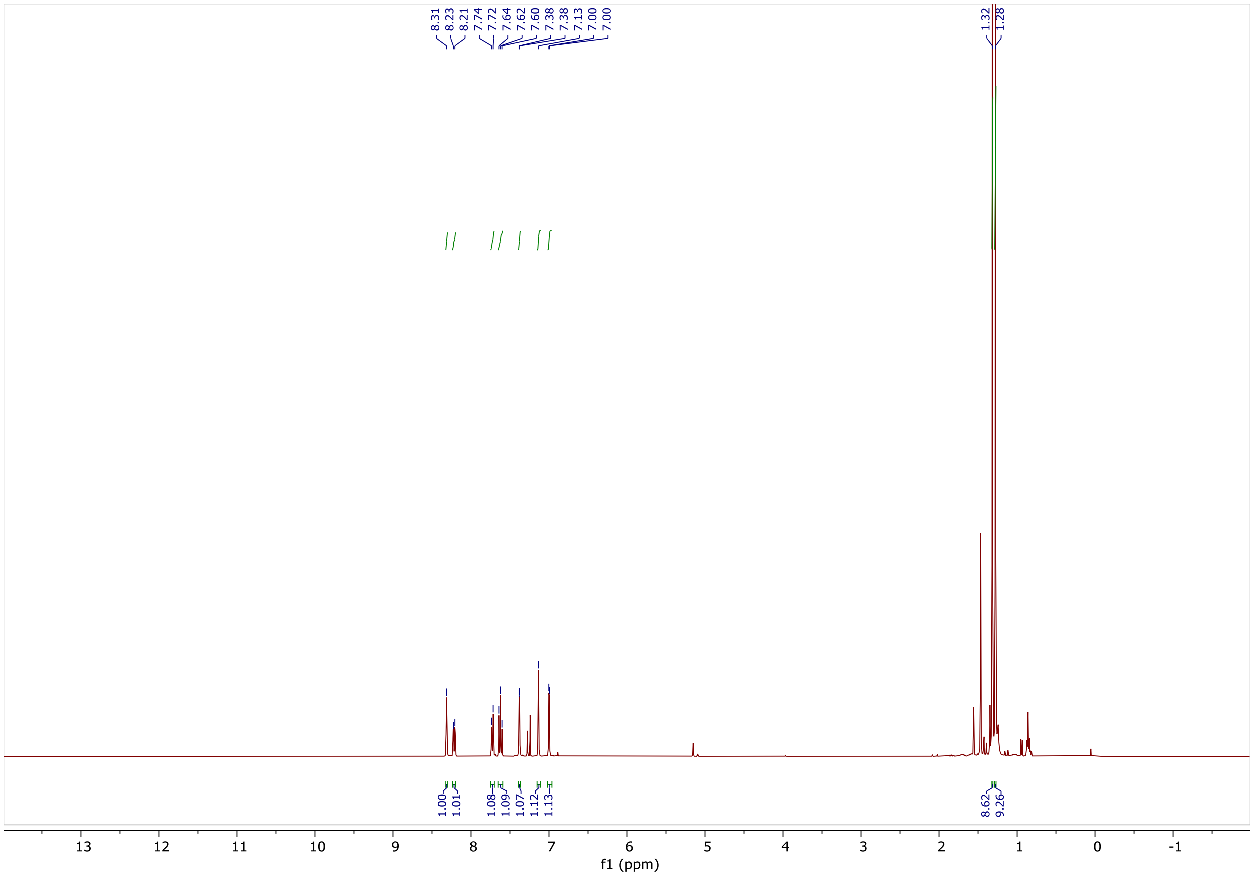

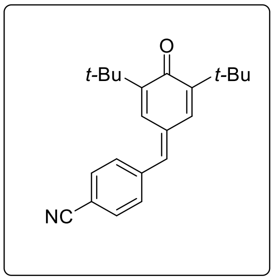

Figure S66. ^{1}H NMR (400 MHz) Spectrum of 4-((3,5-di-tert-butyl-4-oxocyclohexa-2,5-dien-1-ylidene)methyl)benzonitrile.

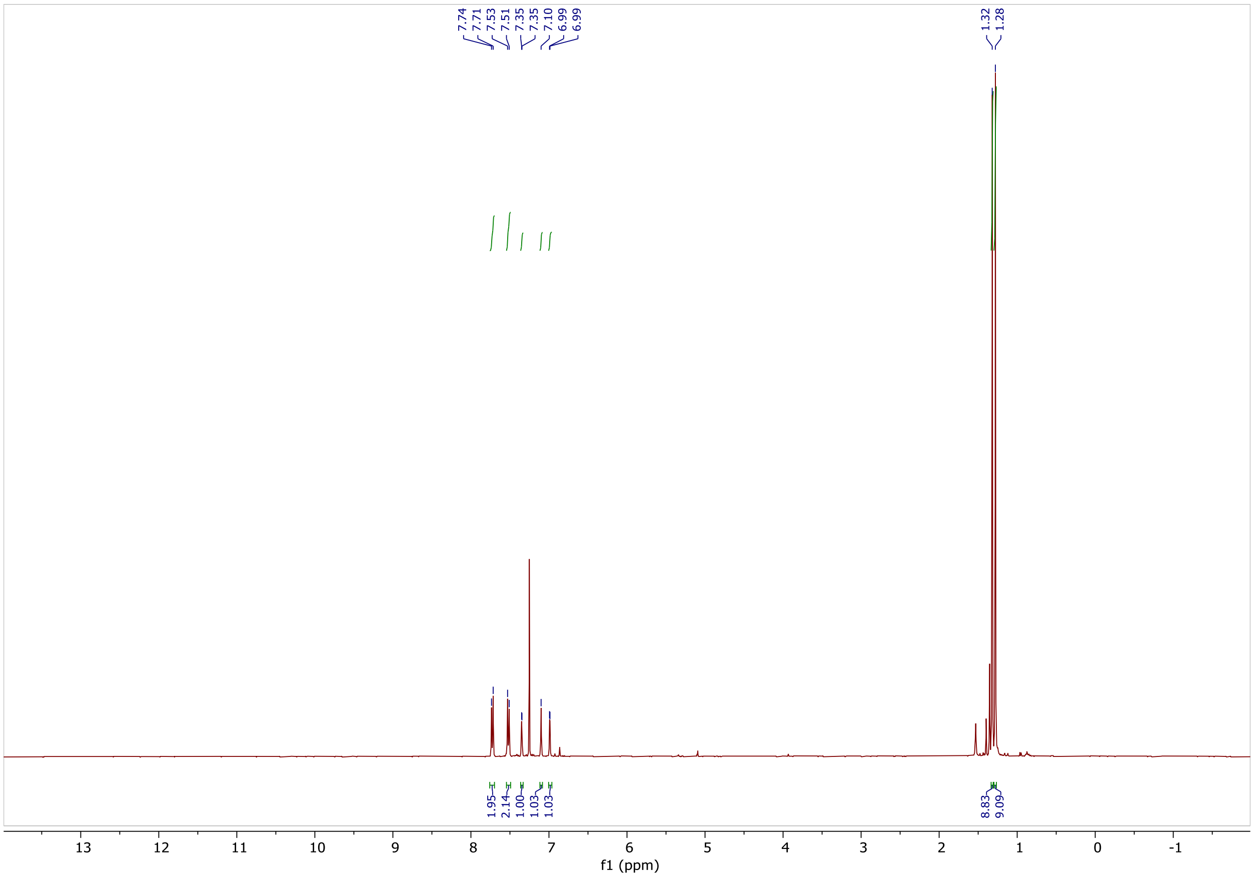

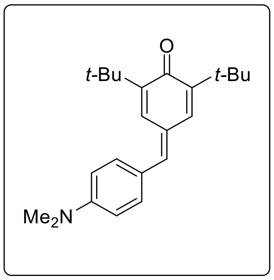

Figure S67. ^{1}H NMR (400 MHz) Spectrum of 2,6-di-*tert*-butyl-4-(4-(dimethylamino)benzylidene)cyclohexa-2,5-dien-1-one.

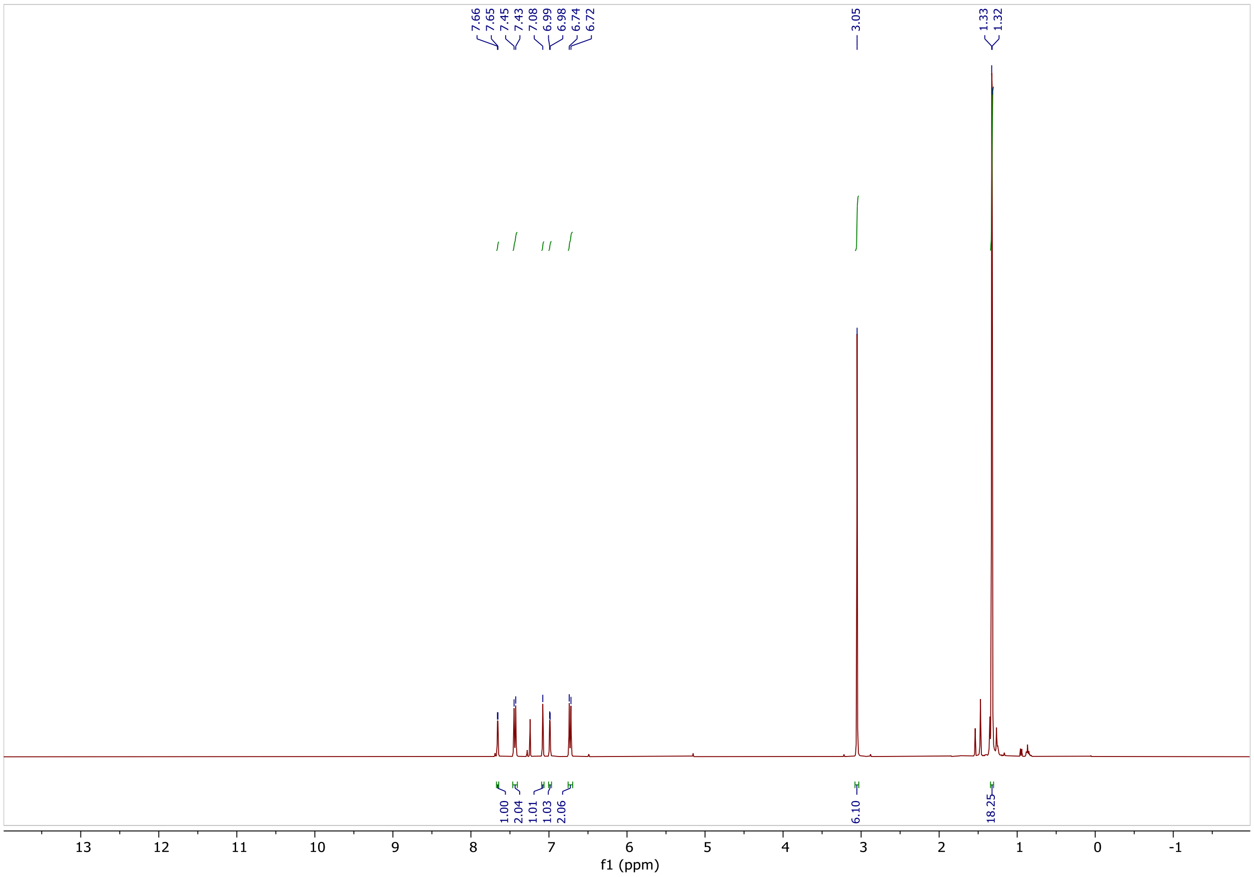

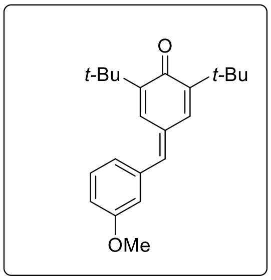

Figure S68. ^{1}H NMR (400 MHz) Spectrum of 2,6-di-*tert*-butyl-4-(3-methoxybenzylidene)cyclohexa-2,5-dien-1-one.

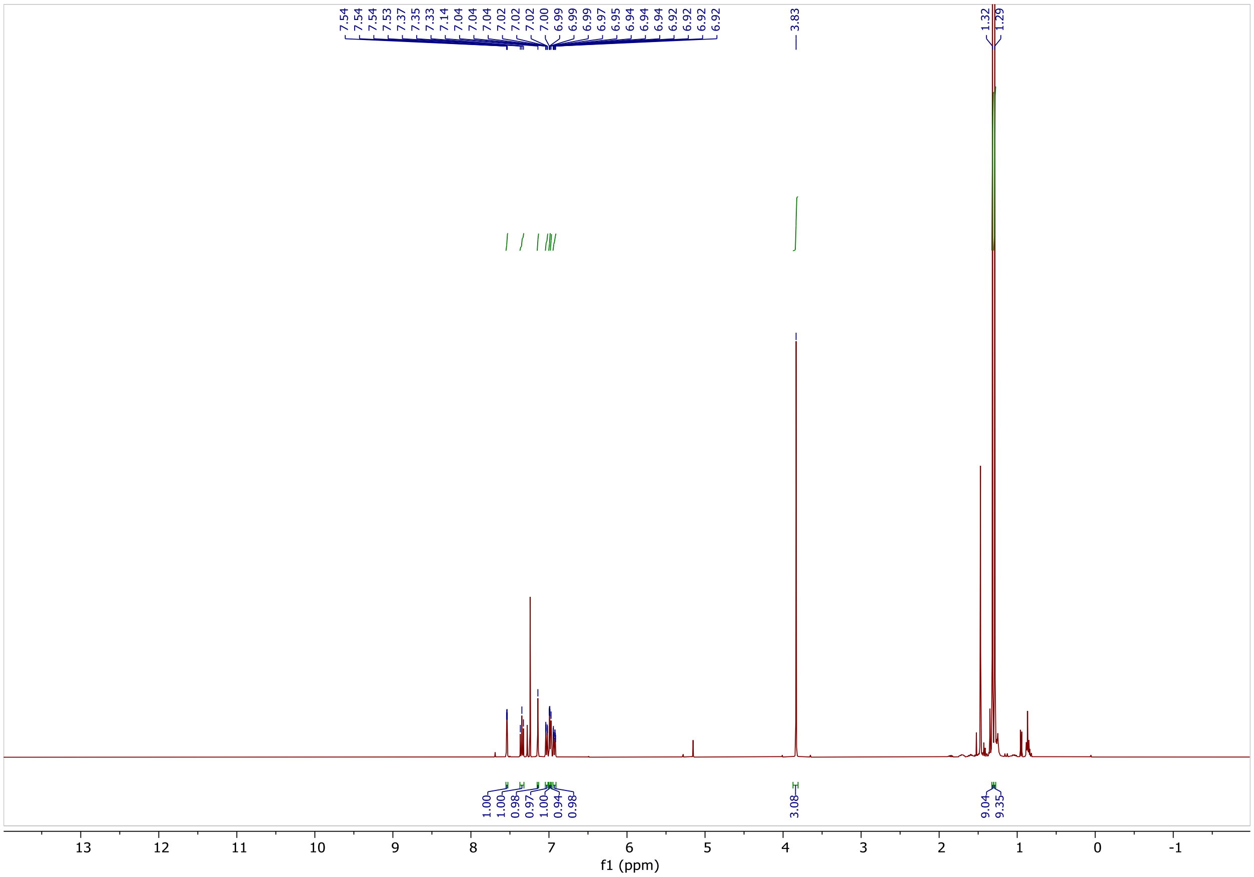

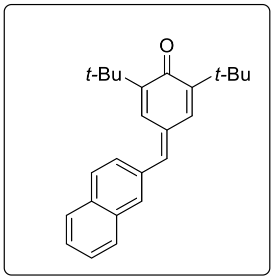

Figure S69. ^{1}H NMR (400 MHz) Spectrum of 2,6-di-*tert*-butyl-4-(naphthalen-2-ylmethylene)cyclohexa-2,5-dien-1-one.

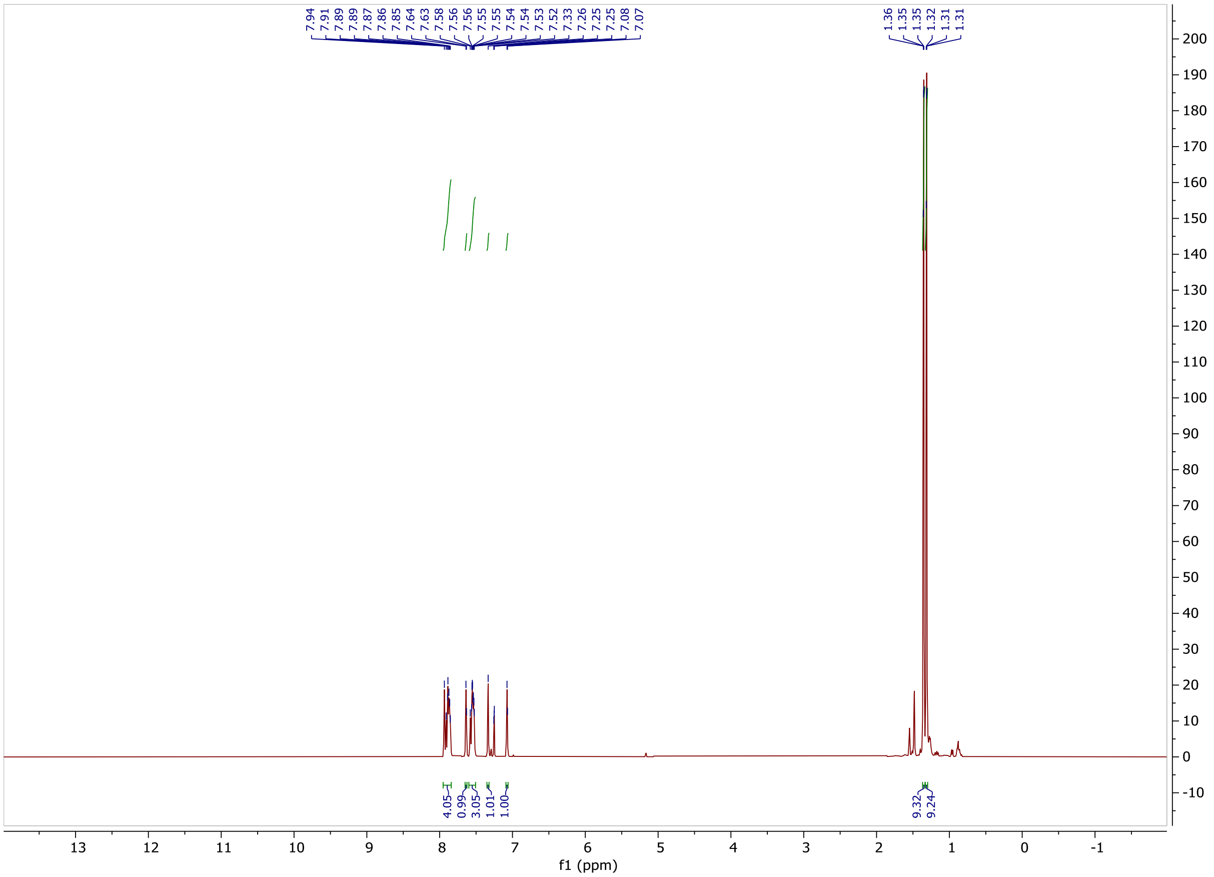

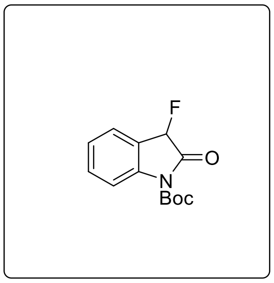

Figure S70. ^{1}H NMR (400 MHz) Spectrum of 3-fluoro-*N*-boc-2-oxindole.

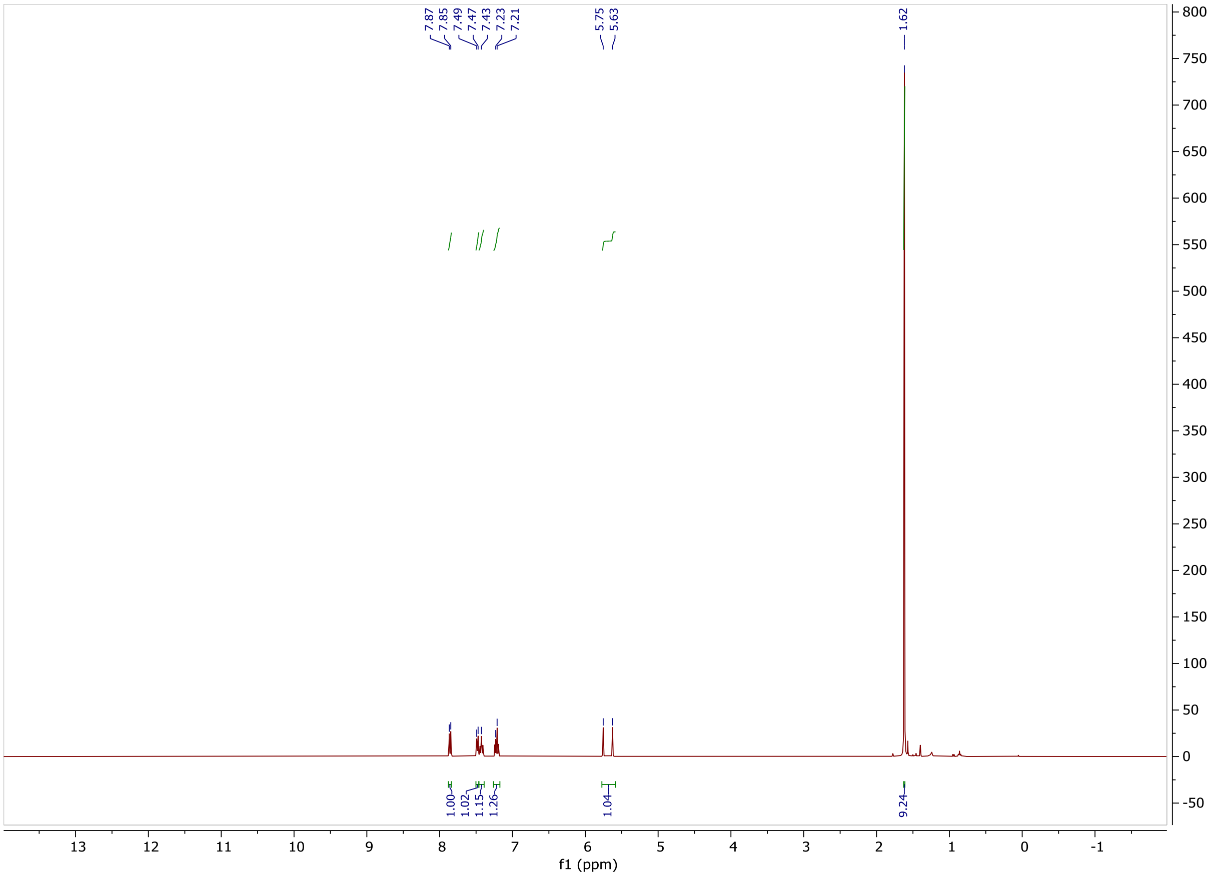

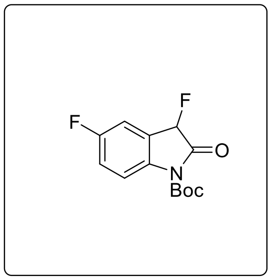

Figure S71. ^{1}H NMR (400 MHz) Spectrum of 3,5-difluoro-*N*-boc-2-oxindole.

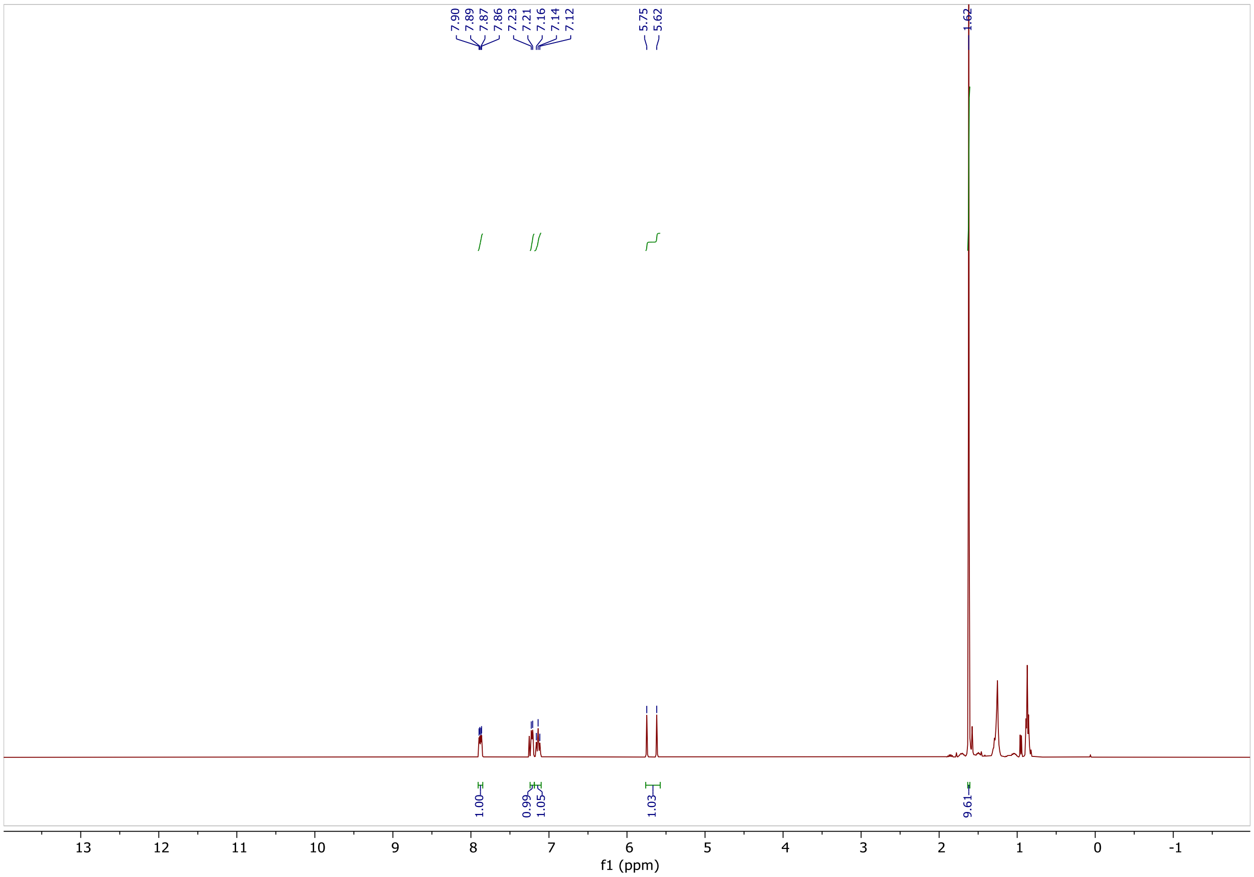

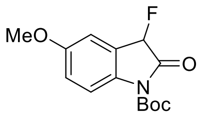

Figure S72. ^{1}H NMR (400 MHz) Spectrum of 3-fluoro-5-methoxy-*N*-boc-2-oxindole.

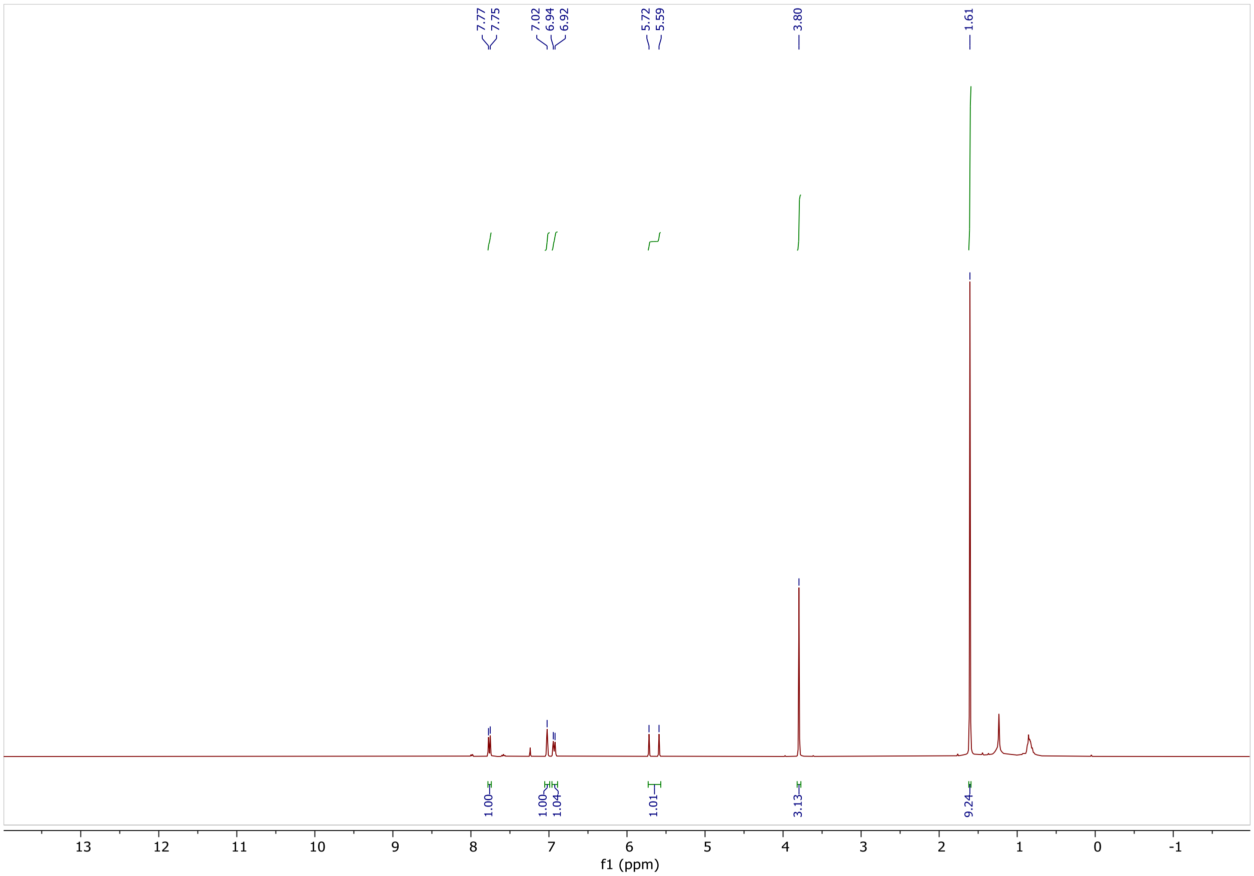

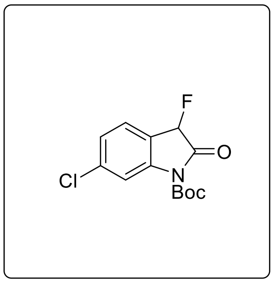

Figure S73. ^{1}H NMR (400 MHz) Spectrum of 3-fluoro-6-chloro-*N*-boc-2-oxindole.

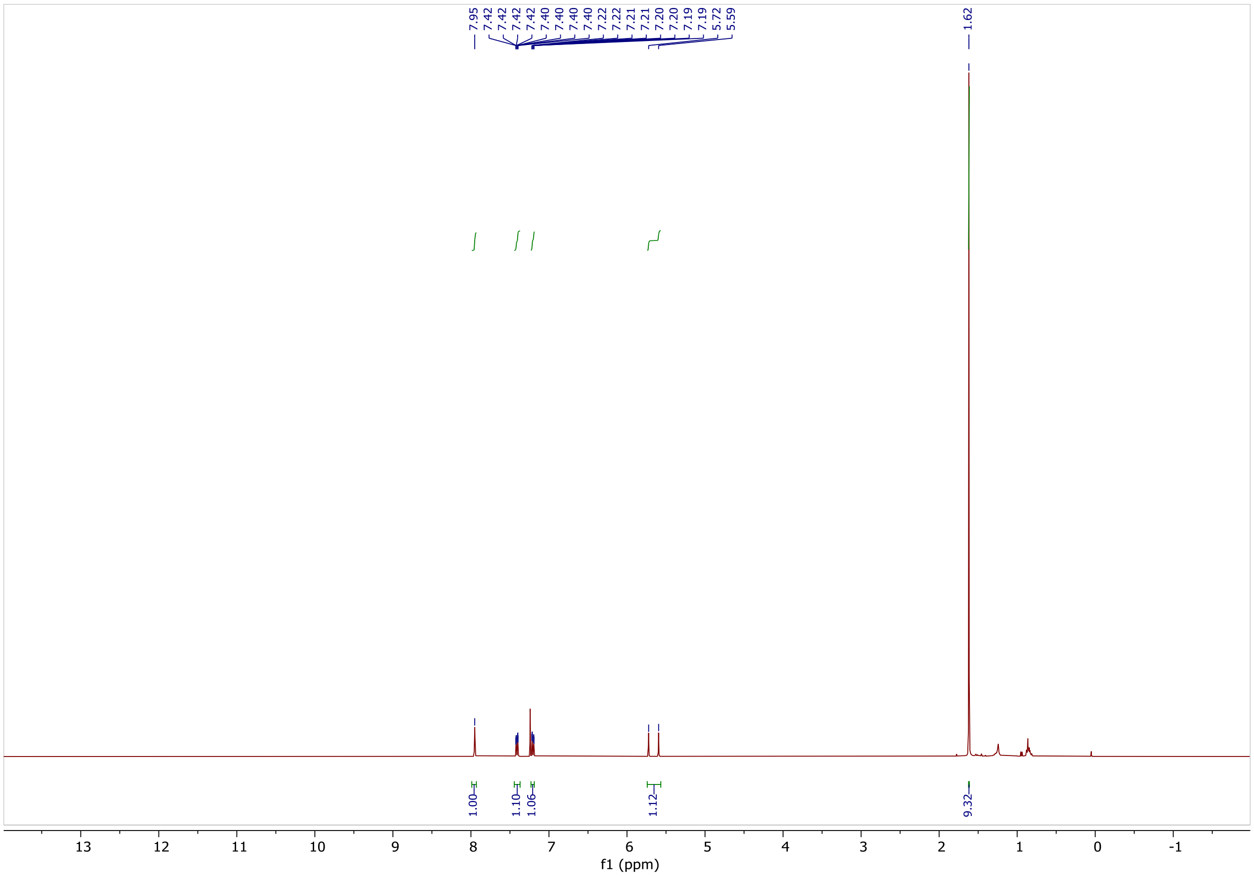

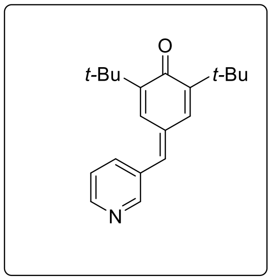

Figure S74. ^{1}H NMR (400 MHz) Spectrum of 2,6-di-*tert*-butyl-4-(pyridin-3-ylmethylene)cyclohexa-2,5-dien-1-one.

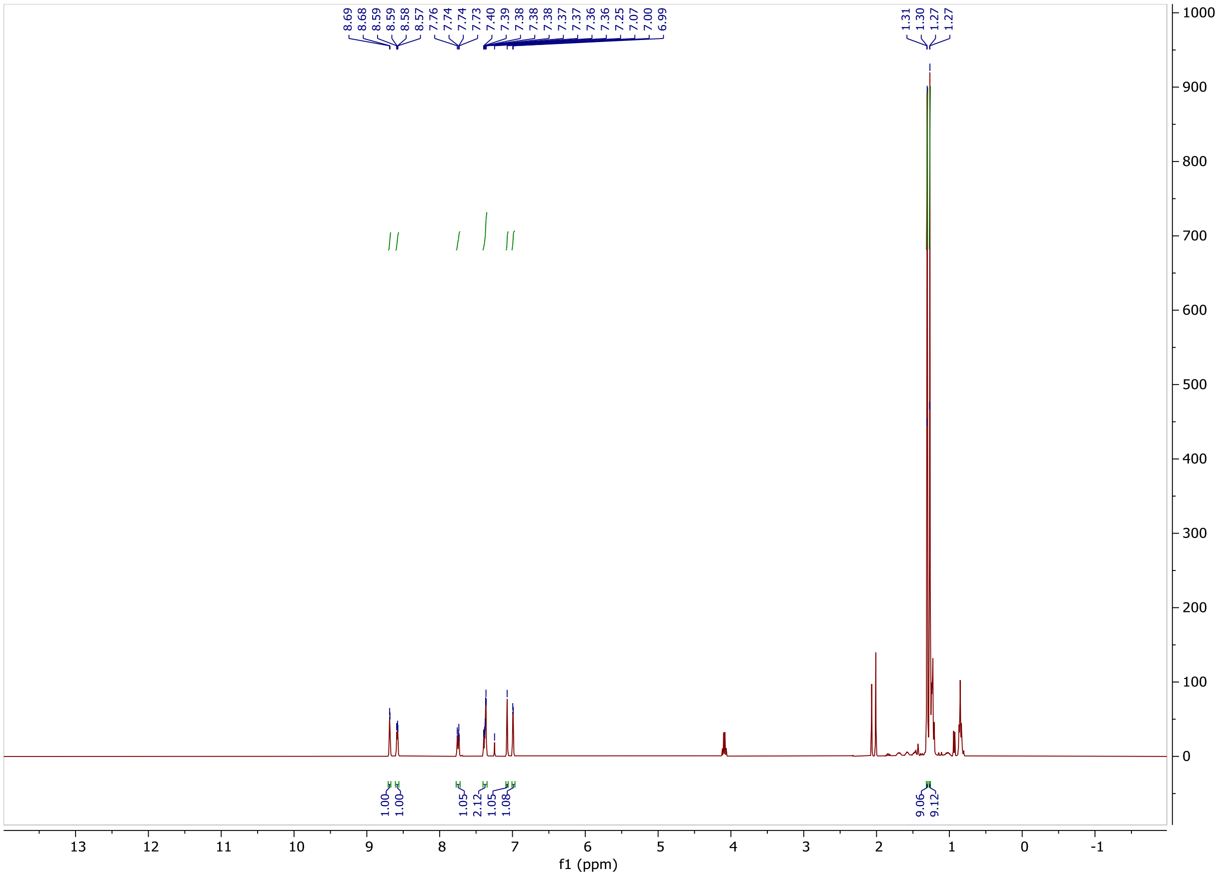

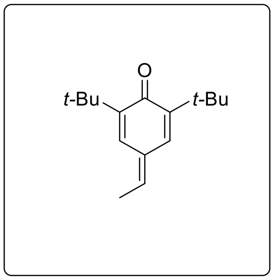

Figure S75. ^{1}H NMR (400 MHz) Spectrum of 2,6-di-*tert*-butyl-4-ethylidenecyclohexa-2,5-dien-1-one.

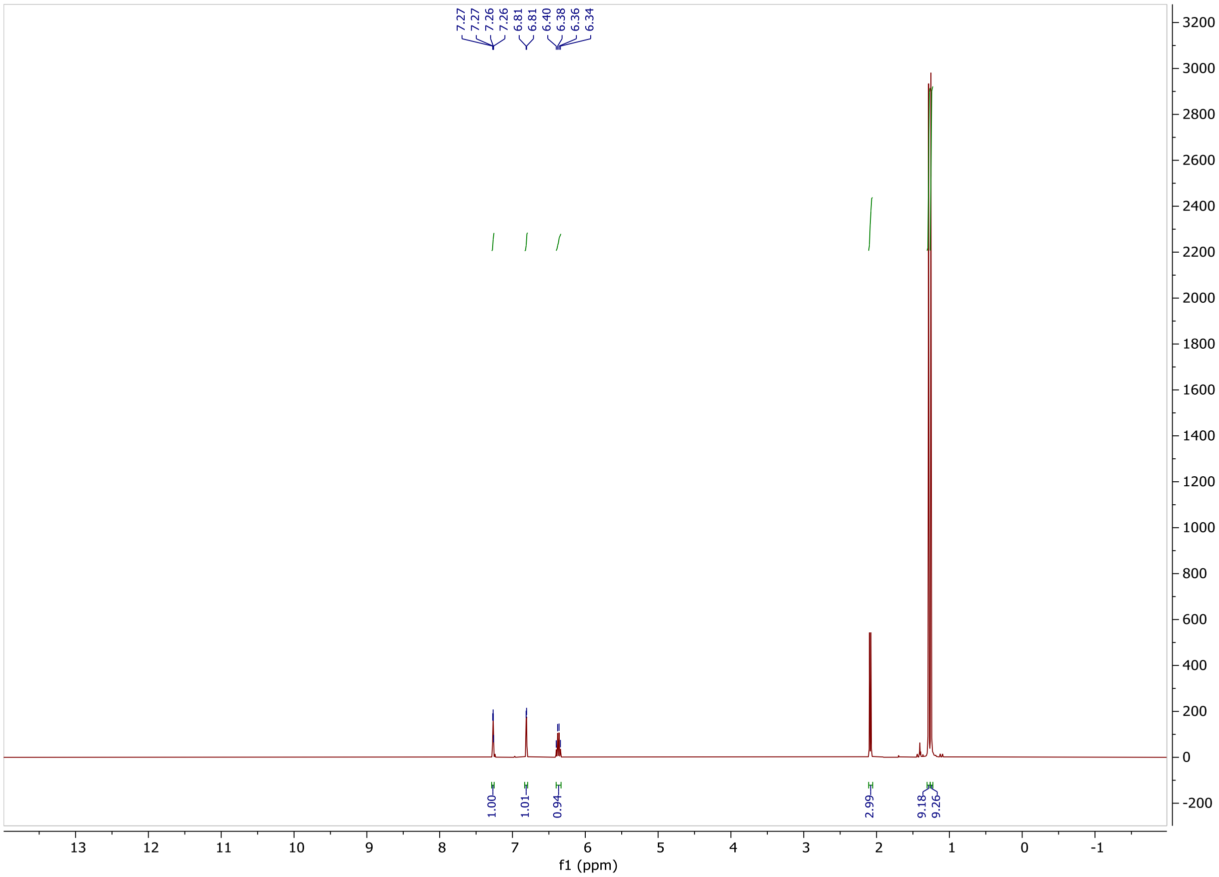

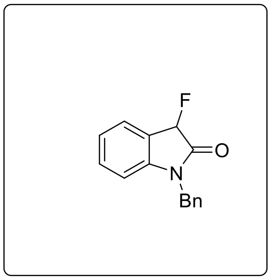

Figure S76. ^{1}H NMR (400 MHz) Spectrum of 3-fluoro-*N*-benzyl-2-oxindole.

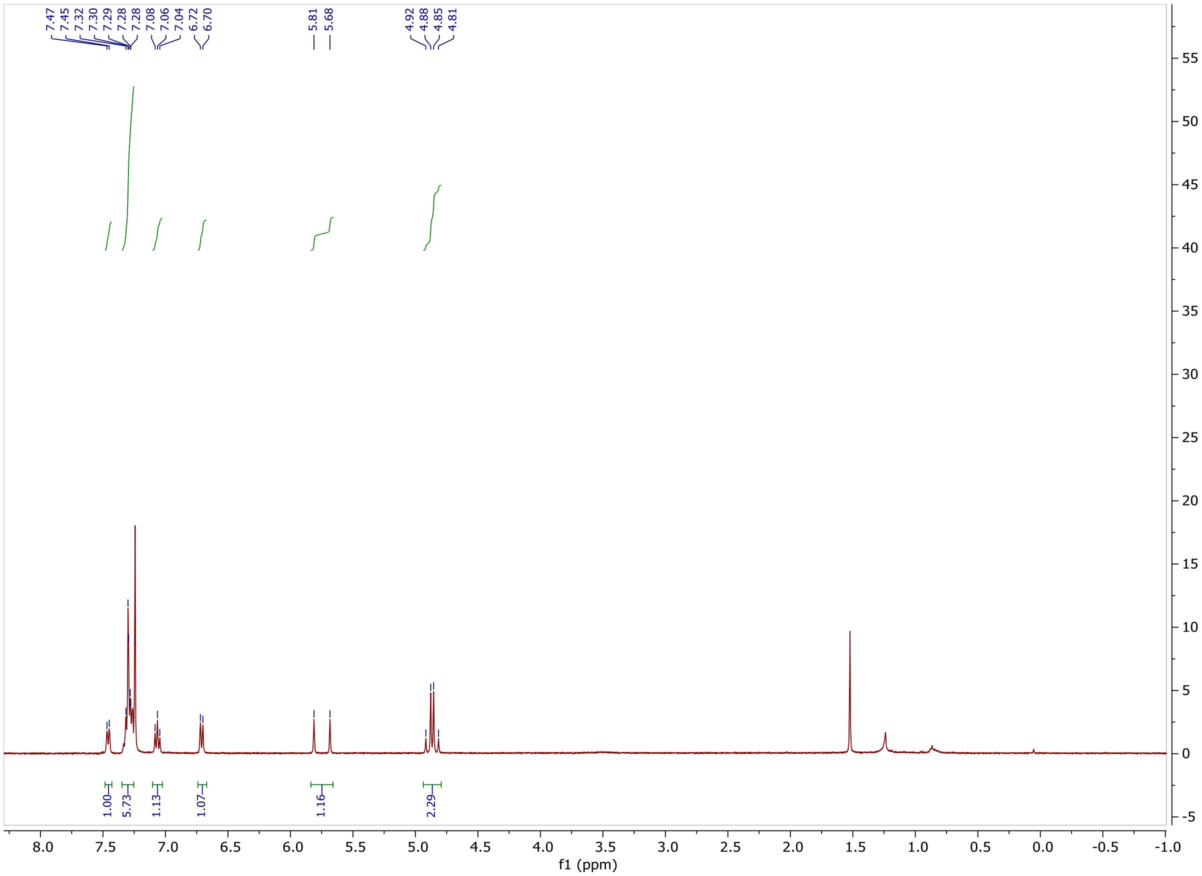

8. HPLC chromatograms

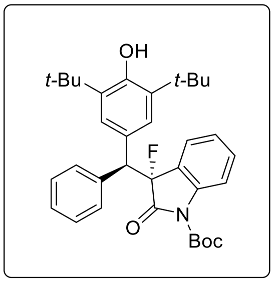

Figure S77. Chiral HPLC separation of a racemic mixture of compound **3** (*dr*=2:1).

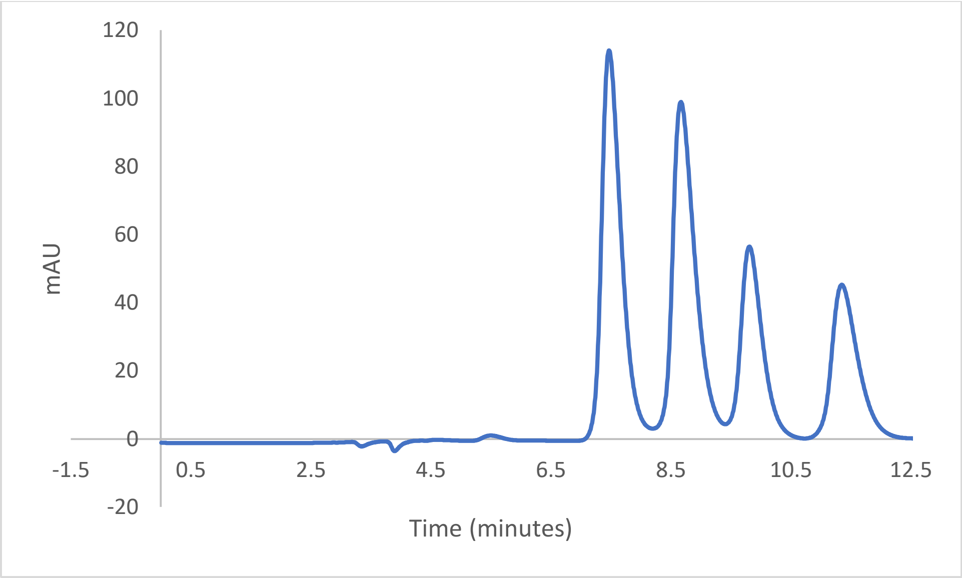

Conditions: (*S,S*)-Whelk-O 1, 99:1 hexanes/IPA, flow rate 1mL/min, λ=254 nm.

#	Time	Area	Height	Width	Area%	Symmetry
1	7.469	2659.9	114.5	0.3529	32.594	0.568
2	8.667	2662.2	99.2	0.403	32.622	0.562
3	9.808	1457	56.5	0.3886	17.854	0.657
4	11.348	1381.5	45.2	0.4609	16.929	0.593

Figure S78. Chiral HPLC separation of the asymmetric reaction product, compound **3** (*ee*=94%, *dr*=29:1).

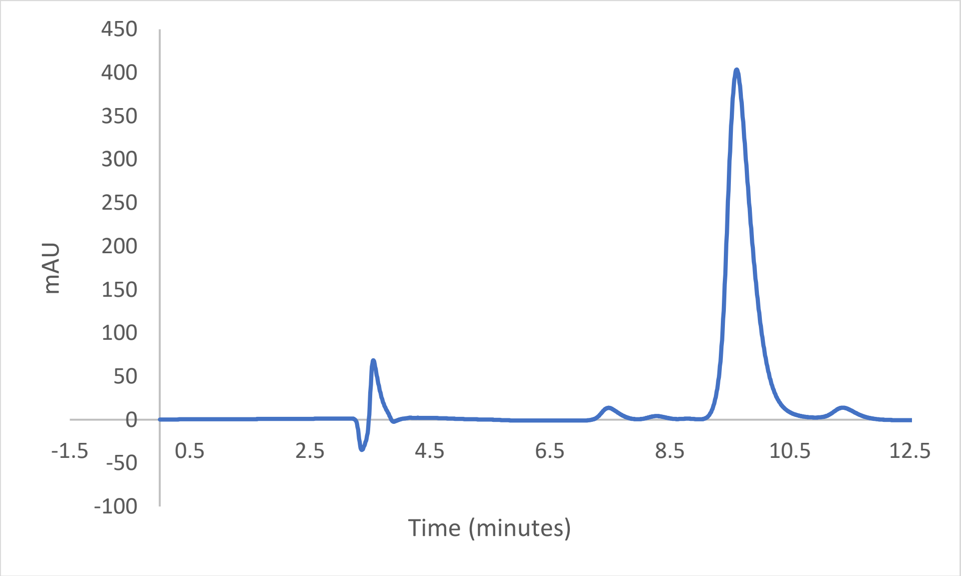

Conditions: (*S,S*)-Whelk-O 1, 99:1 hexanes/IPA, flow rate 1mL/min, λ=254 nm.

#	Time	Area	Height	Width	Area%	Symmetry
1	9.614	11809.1	402.5	0.4383	97.312	0.592
2	11.381	326.2	12.4	0.404	2.688	0.692

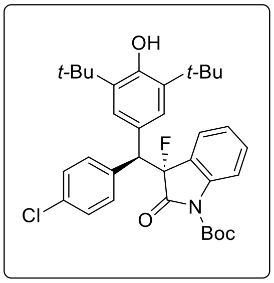

Figure S79. Chiral HPLC separation of a racemic mixture of compound **4** (*dr*=7:1).

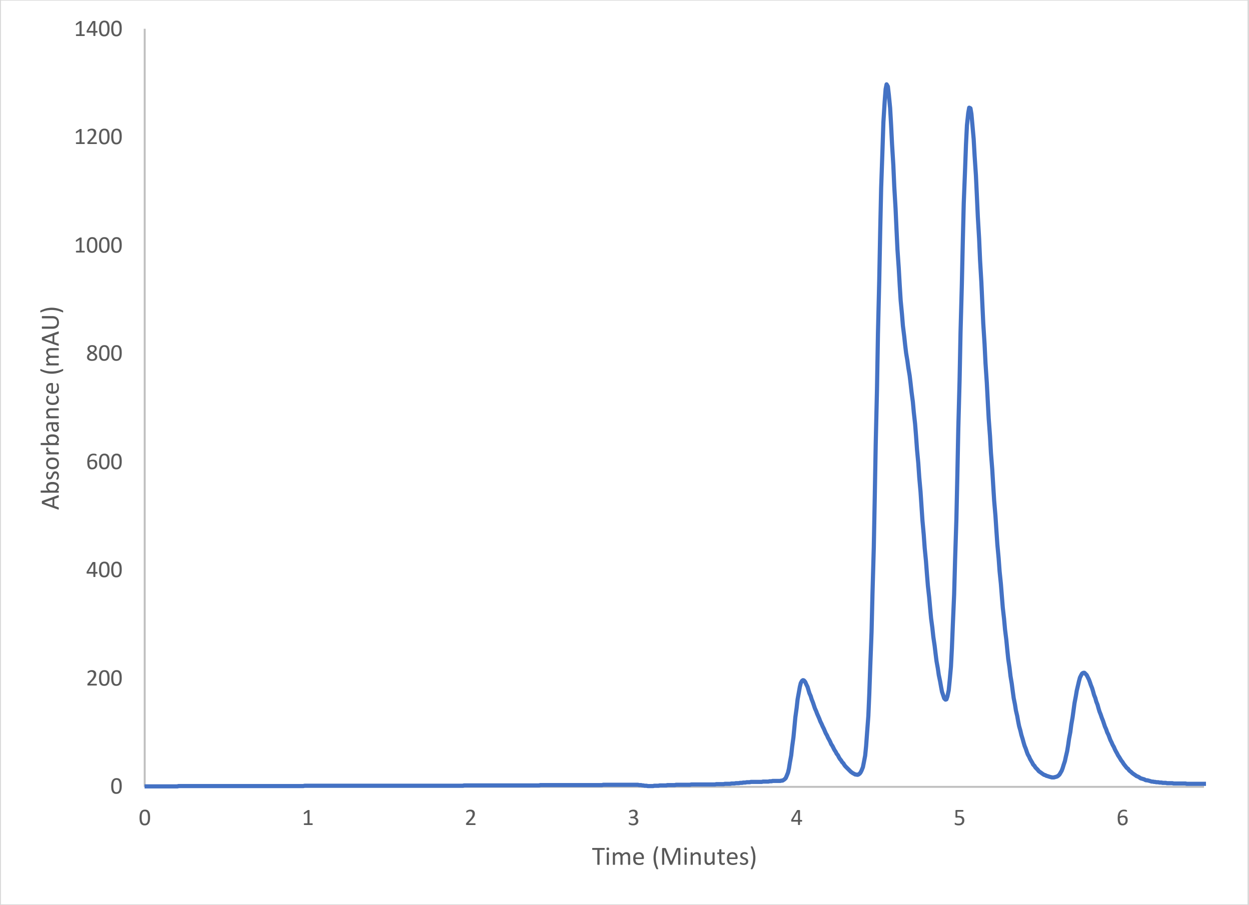

Conditions: Chiralpak IA, 98:2 hexanes/IPA, flow rate 1mL/min, λ=254 nm.

#	Time	Area	Height	Width	Area%	Symmetry
1	4.04	2420.6	189.7	0.1768	5.956	0.435
2	4.554	18962.3	1287	0.2039	46.662	0.376
3	5.062	16585.1	1241.6	0.1938	40.812	0.507
4	5.762	2669.6	196.3	0.2006	6.569	0.512

Figure S80. Chiral HPLC separation of the asymmetric reaction product, compound 4 (*ee*=91%, *dr*=48:1).

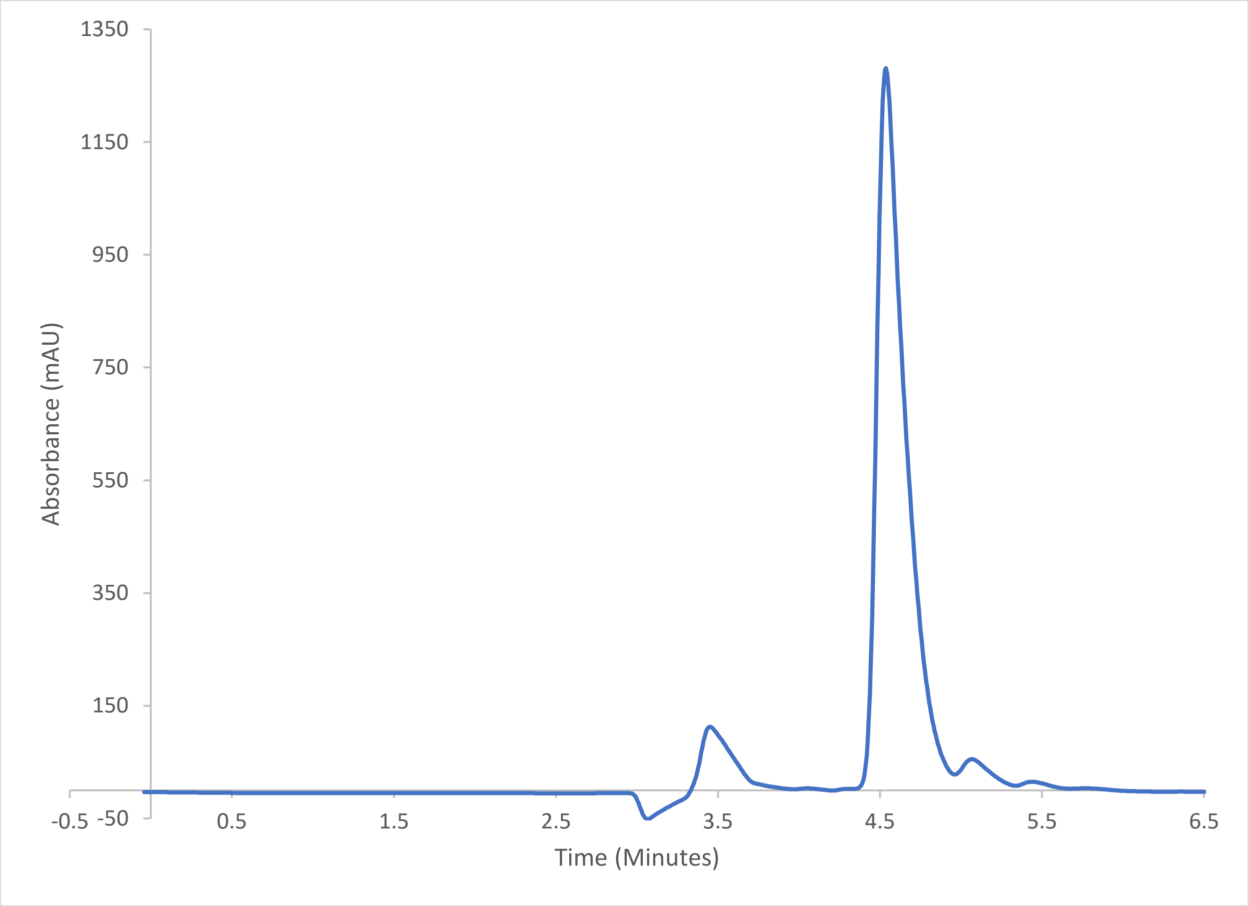

Conditions: Chiralpak IA, 98:2 hexanes/IPA, flow rate 1mL/min, λ=254 nm.

#	Time	Area	Height	Width	Area%	Symmetry
1	4.536	15984	1279.2	0.1778	95.940	0.425
2	5.067	676.4	52.6	0.1902	4.060	0.558

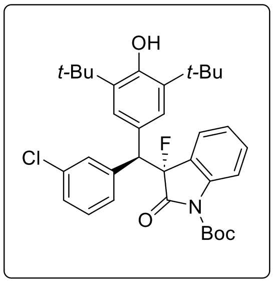

Figure S81. Chiral HPLC separation of a racemic mixture of compound **5** (*dr*=12:1).

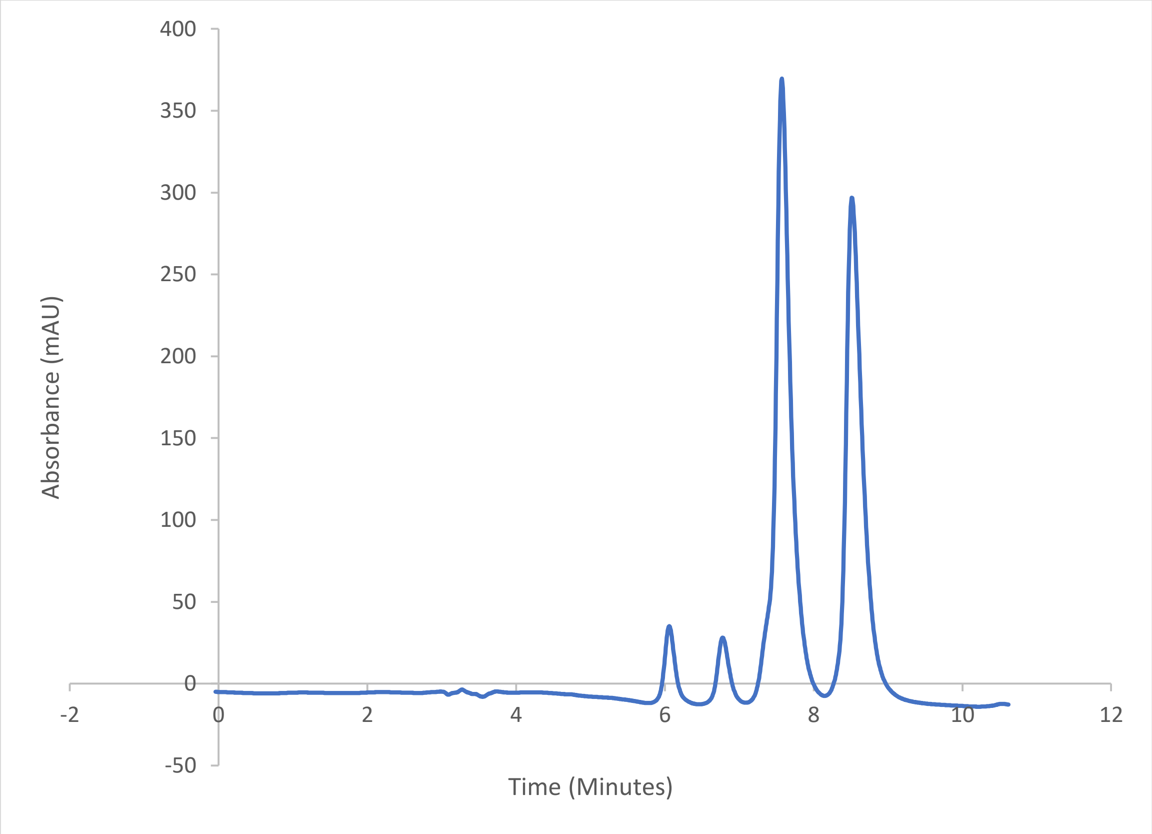

Conditions: (*S,S*)-Whelk-O 1, 98:2 hexanes/IPA, flow rate 1mL/min, λ=254 nm.

#	Time	Area	Height	Width	Area%	Symmetry
1	6.059	480.6	47.3	0.1526	4.230	0.79
2	6.777	446.9	40.3	0.1673	3.934	0.806
3	7.572	5281.9	377	0.2335	46.492	0.633
4	8.515	5151.5	309.7	0.242	45.344	0.533

Figure S82. Chiral HPLC separation of the asymmetric reaction product, compound **5** (*ee*=83%, *dr*=14:1).

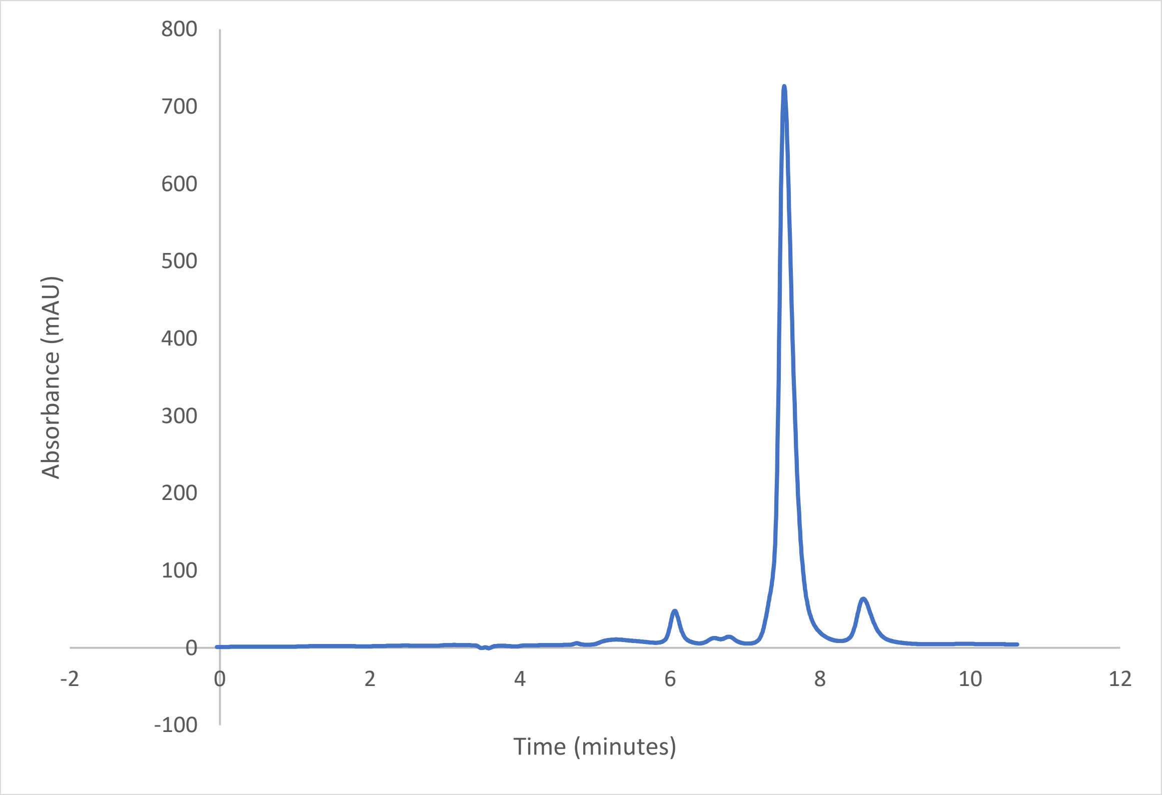

Conditions: (*S,S*)-Whelk-O 1, 98:2 hexanes/IPA, flow rate 1mL/min, λ=254 nm.

#	Time	Area	Height	Width	Area%	Symmetry
1	7.523	10225.5	721.6	0.2073	91.270	0.608
2	8.577	978.1	58.6	0.2447	8.730	0.674

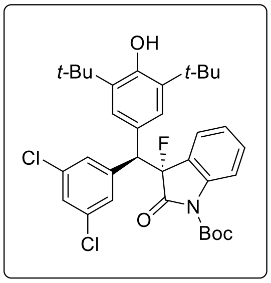

Figure S83. Chiral HPLC separation of a racemic mixture of compound **6** (*dr*=17:1).

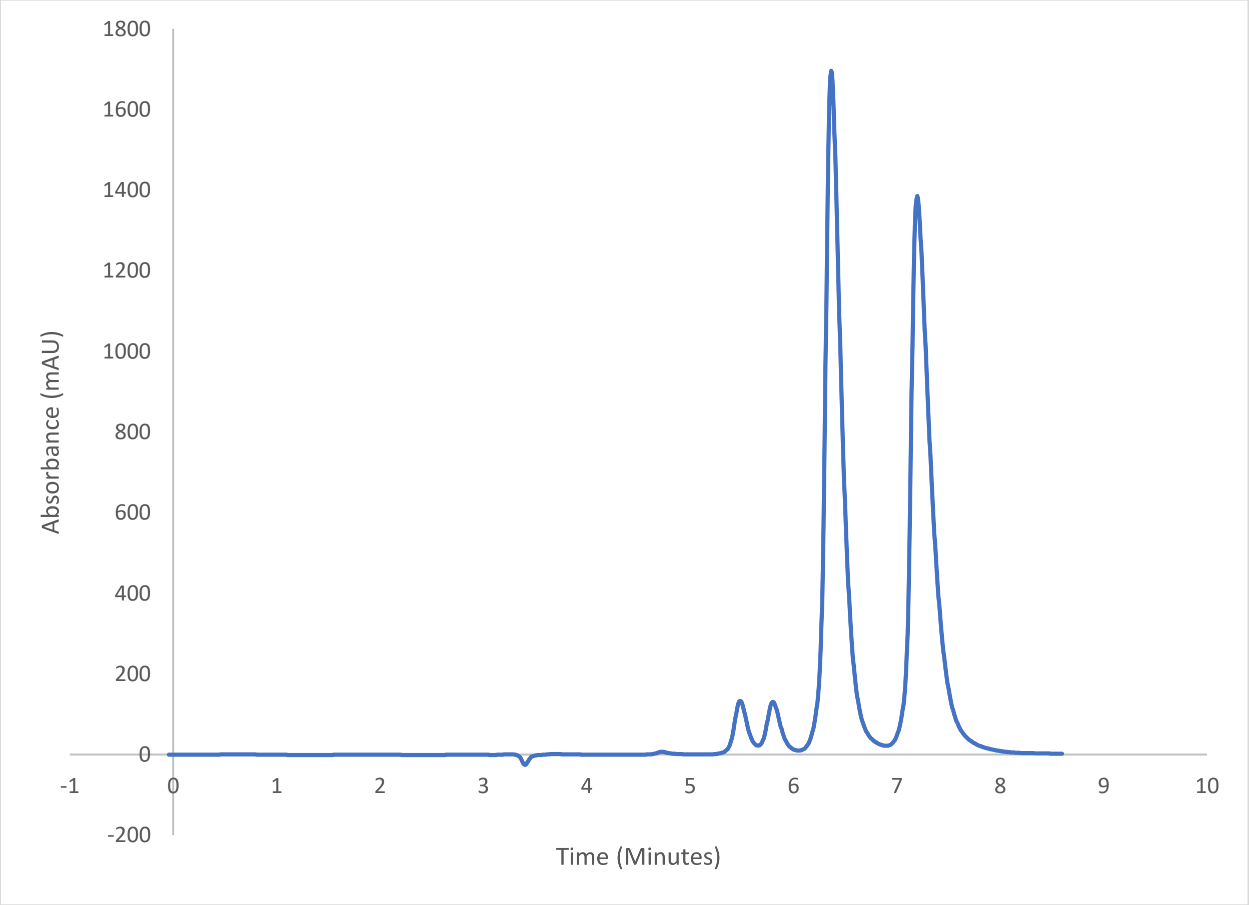

Conditions: (*S,S*)-Whelk-O 1, 98:2 hexanes/IPA, flow rate 1mL/min, λ=254 nm.

#	Time	Area	Height	Width	Area%	Symmetry
1	5.484	1166.3	129.8	0.1349	2.810	0.803
2	5.801	1155	123.5	0.1411	2.783	0.833
3	6.366	19430.8	1687.6	0.1683	46.815	0.539
4	7.196	19753.3	1378.9	0.2071	47.592	0.426

Figure S84. Chiral HPLC separation of the asymmetric reaction product, compound **6** (*ee*=85%, *dr*=26:1).

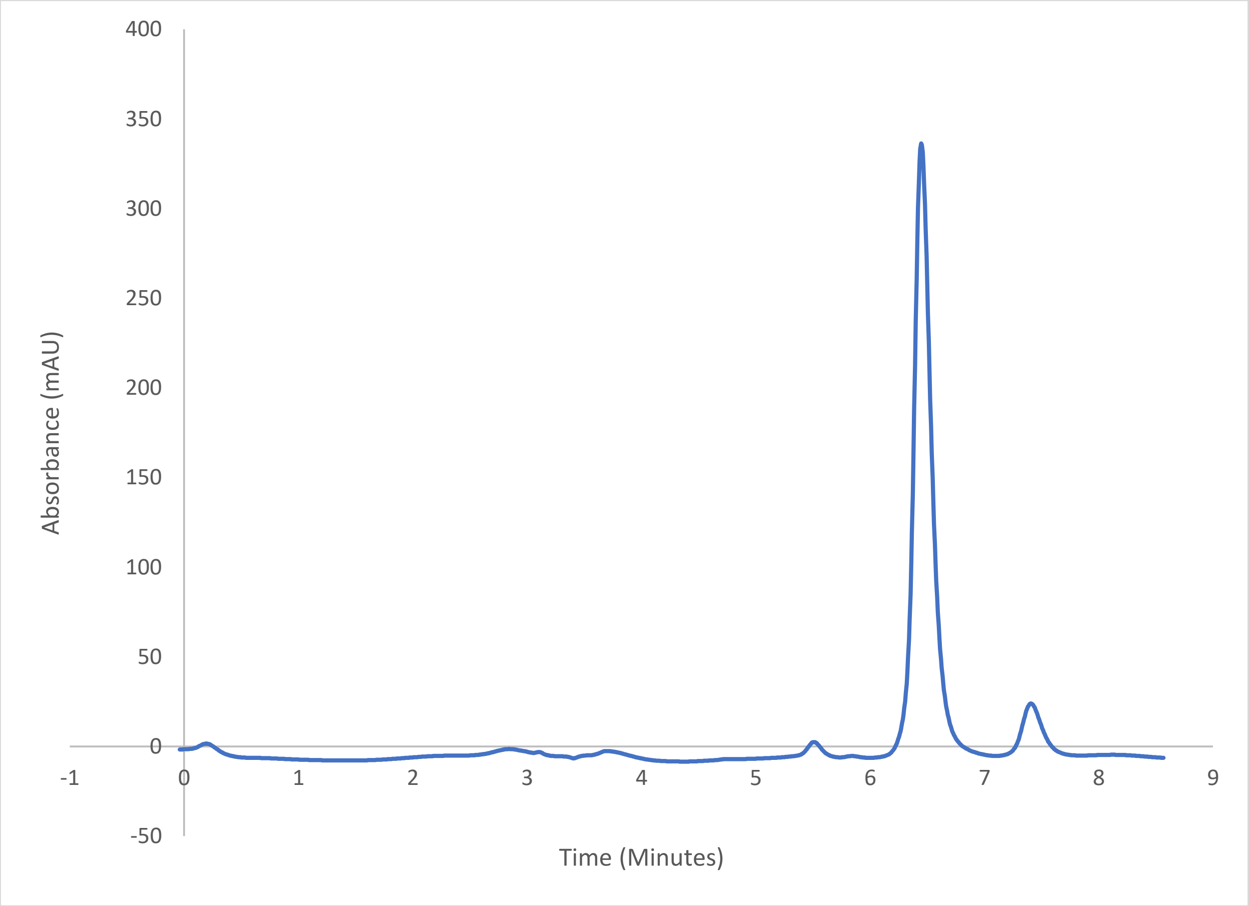

Conditions: (*S,S*)-Whelk-O 1, 98:2 hexanes/IPA, flow rate 1mL/min, λ=254 nm.

#	Time	Area	Height	Width	Area%	Symmetry
1	6.448	3845.4	342.6	0.1669	91.145	0.701
2	7.407	373.6	29.3	0.1926	8.855	0.776

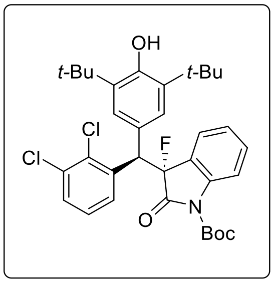

Figure S85. Chiral HPLC separation of a racemic mixture of compound **7** (*dr*=3:1).

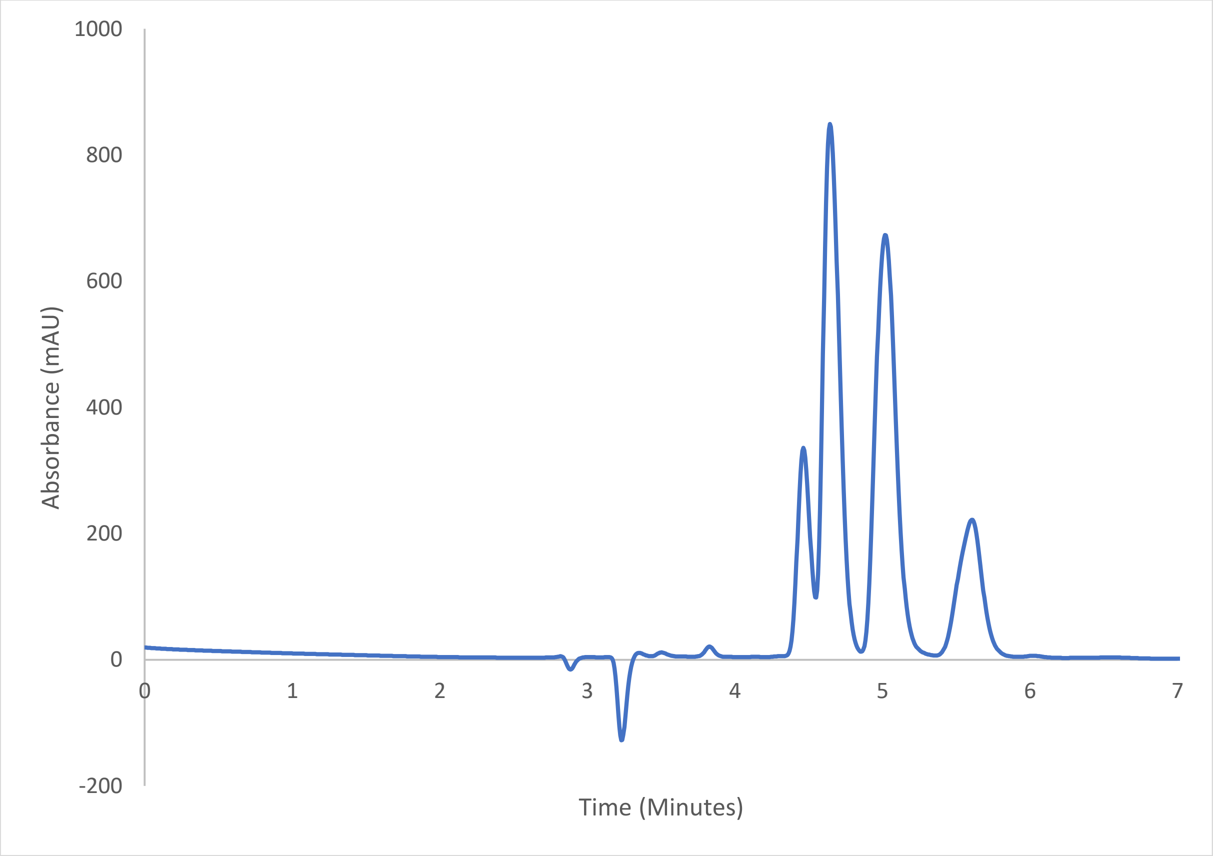

Conditions: Amylose-1, 99:1 Hexanes/IPA, flow rate 1mL/min, λ=254nm. Baseline adjusted by +110 mAU.

#	Time	Area	Height	Width	Area%	Symmetry
1	4.464	1982.2	331.2	0.0918	11.330	0.878
2	4.645	6578.7	844.2	0.1249	37.604	0.692
3	5.02	6486.1	667.8	0.1553	37.075	0.88
4	5.607	2447.5	216.4	0.184	13.990	1.242

Figure S86. Chiral HPLC separation of the asymmetric reaction product, compound **7** (*ee*=88%, *dr*=20:1)

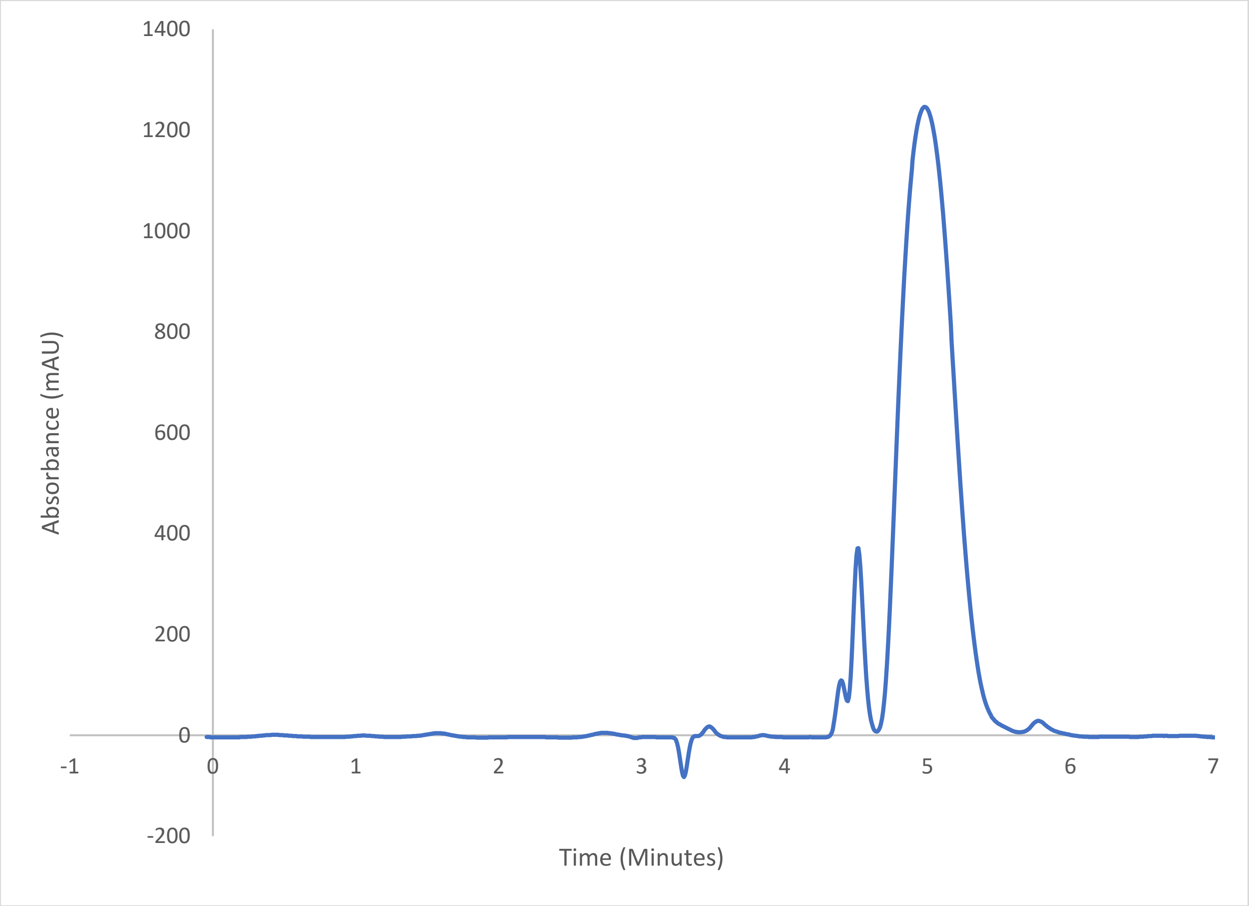

Conditions: Amylose-1, 99:1 Hexanes/IPA, flow rate 1mL/min, λ=254 nm.

#	Time	Area	Height	Width	Area%	Symmetry
1	4.515	2038.5	376.5	0.0832	6.075	0.832
2	4.984	31517.7	1250.1	0.4201	93.925	0.794

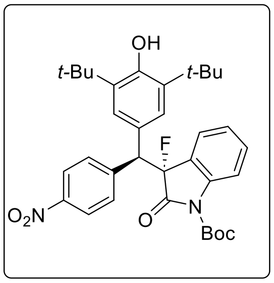

Figure S87. Chiral HPLC separation of a racemic mixture of compound **8** (*dr*=4:1).

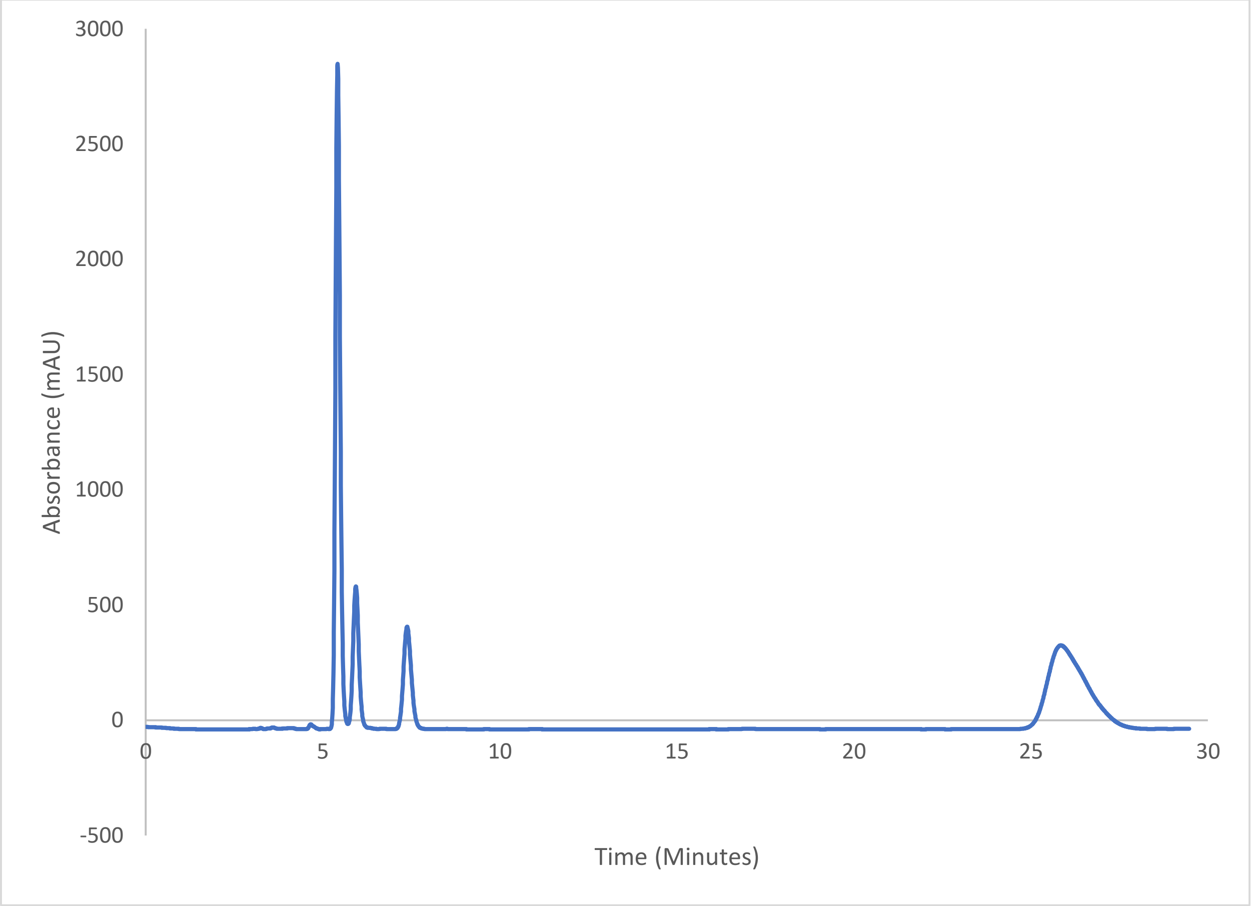

Conditions: Chiralpak AD-H, 97:3 hexanes/IPA, flow rate 1mL/min, λ=254 nm.

#	Time	Area	Height	Width	Area%	Symmetry
1	5.416	27049.5	2887.4	0.1473	39.079	0.789
2	5.932	7343.4	619	0.1824	10.609	0.911
3	7.379	6902	444.7	0.2411	9.971	0.833
4	25.845	27923.4	363.4	1.1105	40.341	0.518

Figure S88. Chiral HPLC separation of the asymmetric reaction product, compound **8** (*ee*=94%, *dr*=20:1).

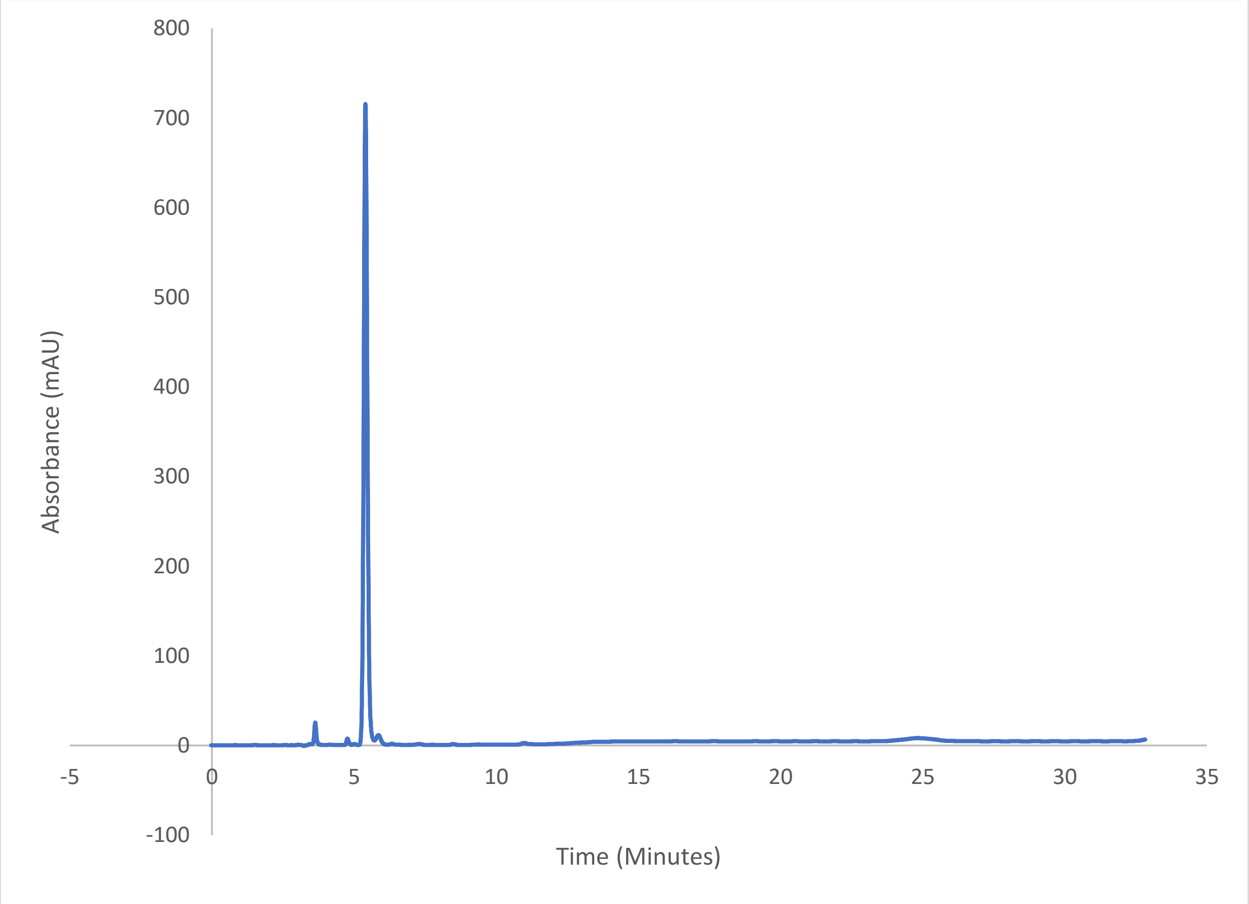

Conditions: Chiralpak AD-H, 97:3 hexanes/IPA, flow rate 1mL/min, λ=254 nm.

#	Time	Area	Height	Width	Area%	Symmetry
1	5.397	6388.7	715.1	0.1382	97.070	0.852
2	24.806	192.9	3	1.065	2.930	0.807

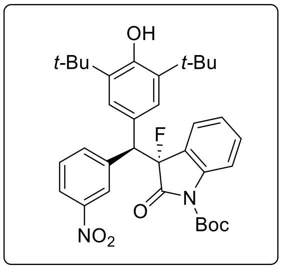

Figure S89. Chiral HPLC separation of a racemic mixture of compound **9** (*dr*=5:1).

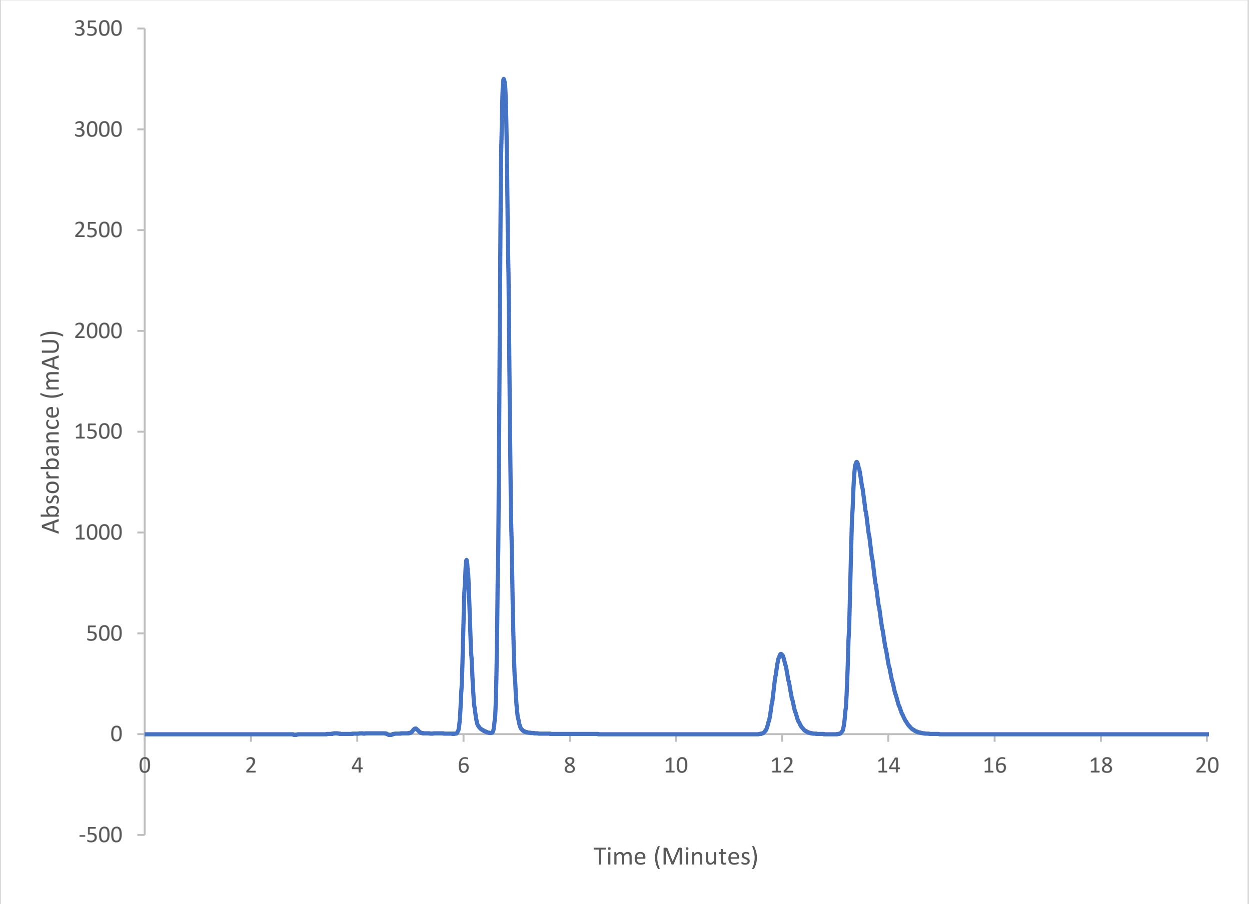

Conditions: Amylose-1, 98:2 hexanes/IPA, flow rate 1mL/min, λ=254 nm.

#	Time	Area	Height	Width	Area%	Symmetry
1	6.058	8815.4	862.8	0.1572	8.430	0.735
2	6.76	41237	3249.4	0.2001	39.433	0.718
3	11.98	8753.7	398.5	0.3403	8.371	0.728
4	13.403	45769.4	1351.1	0.5052	43.767	0.289

Figure S90. Chiral HPLC separation of the asymmetric reaction product, compound **9** (*ee*=81%, *dr*=50:1).

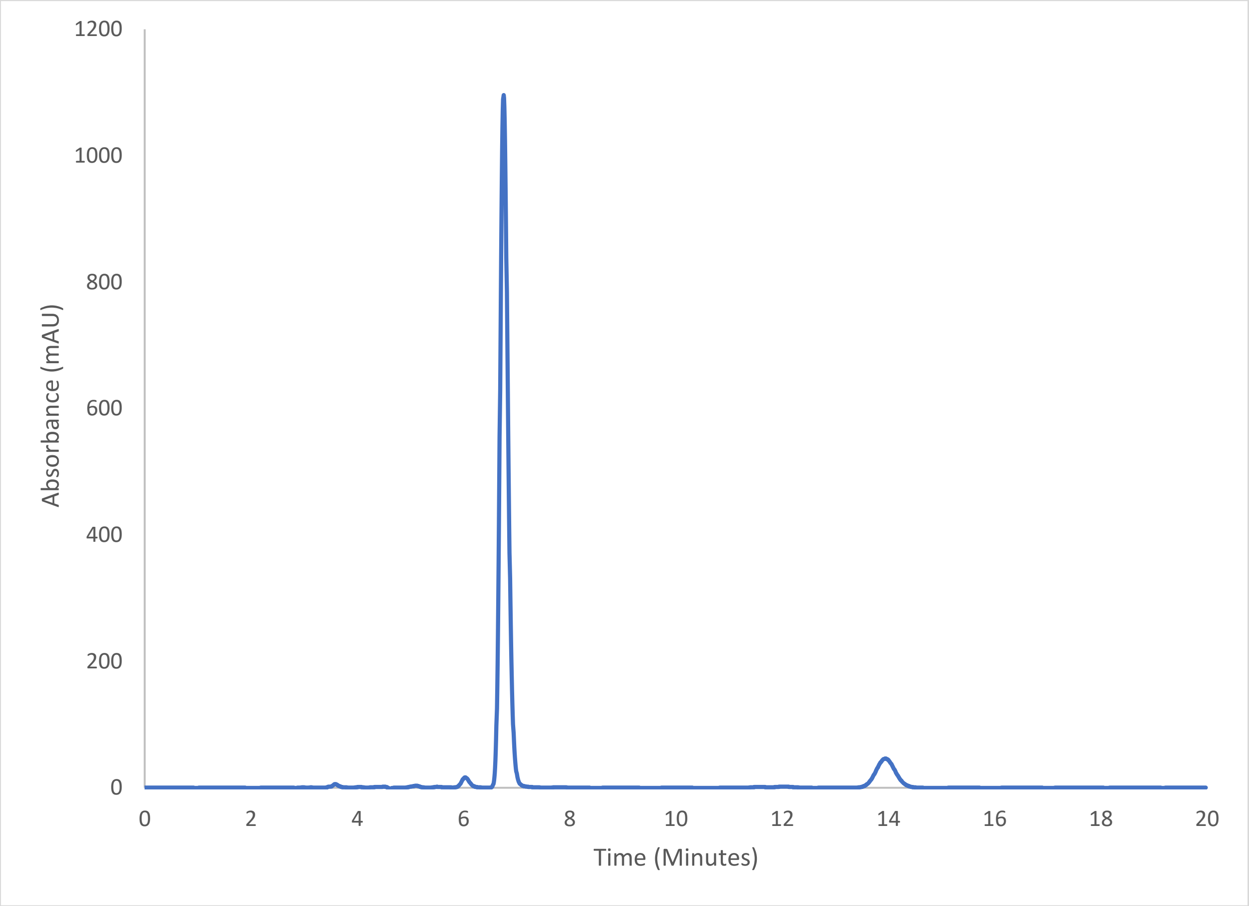

Conditions: Amylose-1, 98:2 hexanes/IPA, flow rate 1mL/min, λ=254 nm.

#	Time	Area	Height	Width	Area%	Symmetry
1	6.756	11809	1101.1	0.1787	90.570	0.808
2	13.945	1229.6	46.8	0.4381	9.430	0.954

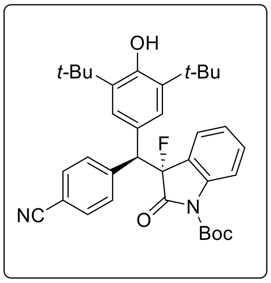

Figure S91. Chiral HPLC separation of a racemic mixture of compound **10** (*dr*=9:1).

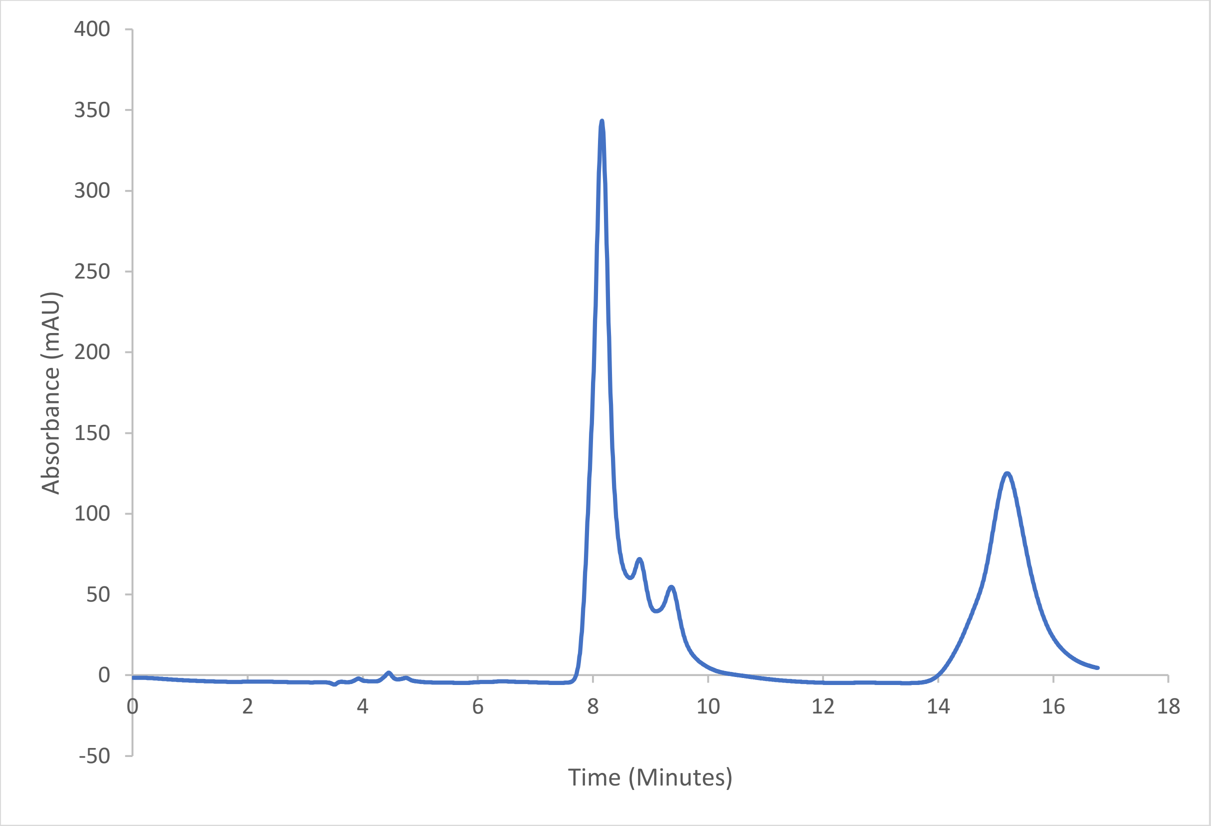

Conditions: Chiralpak-IA, 99:1 hexanes/IPA, flow rate 1mL/min, λ=254 nm.

#	Time	Area	Height	Width	Area%	Symmetry
1	8.156	7127.4	351.5	0.3379	45.050	1.271
2	8.852	582.9	37.2	0.2611	3.685	0.342
3	9.392	753	36.1	0.348	4.759	0.397
4	15.2	7357.9	125.3	0.8353	46.506	1.011

Figure S92. Chiral HPLC separation of the asymmetric reaction product, compound **10** (*ee*= 91%, *dr*= 20:1)

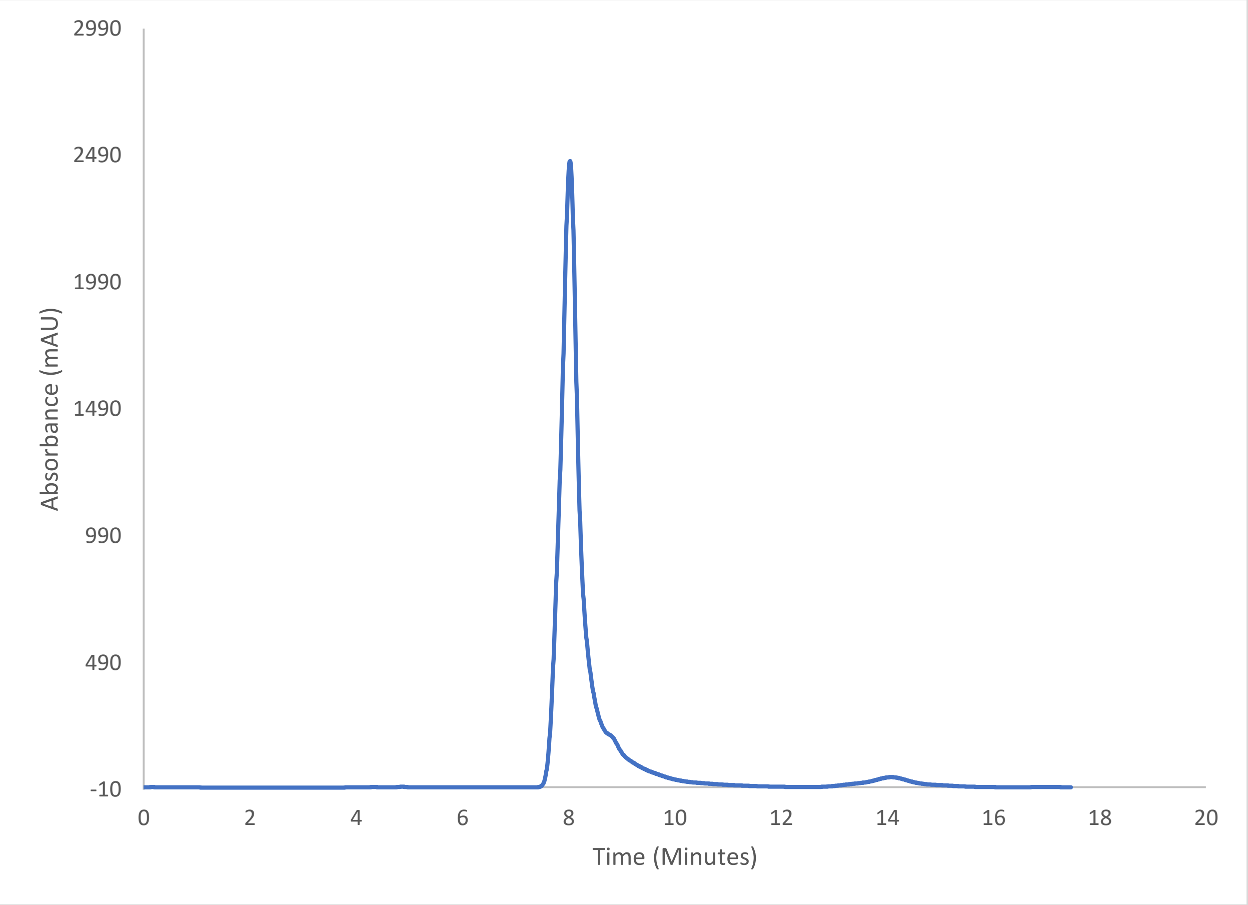

#	Time	Area	Height	Width	Area%	Symmetry
1	8.024	65435.9	2486.6	0.4386	96.098	0.893
2	14.075	2657.1	39.3	0.941	3.902	0.97

Conditions: Chiralpak-IA, 99:1 hexanes/IPA, flow rate 1mL/min, λ=254nm.

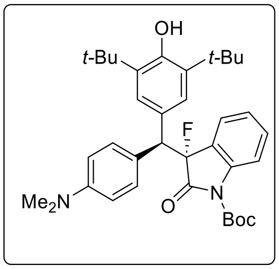

Figure S93. Chiral HPLC separation of a racemic mixture of compound **11** (*dr*=4:1).

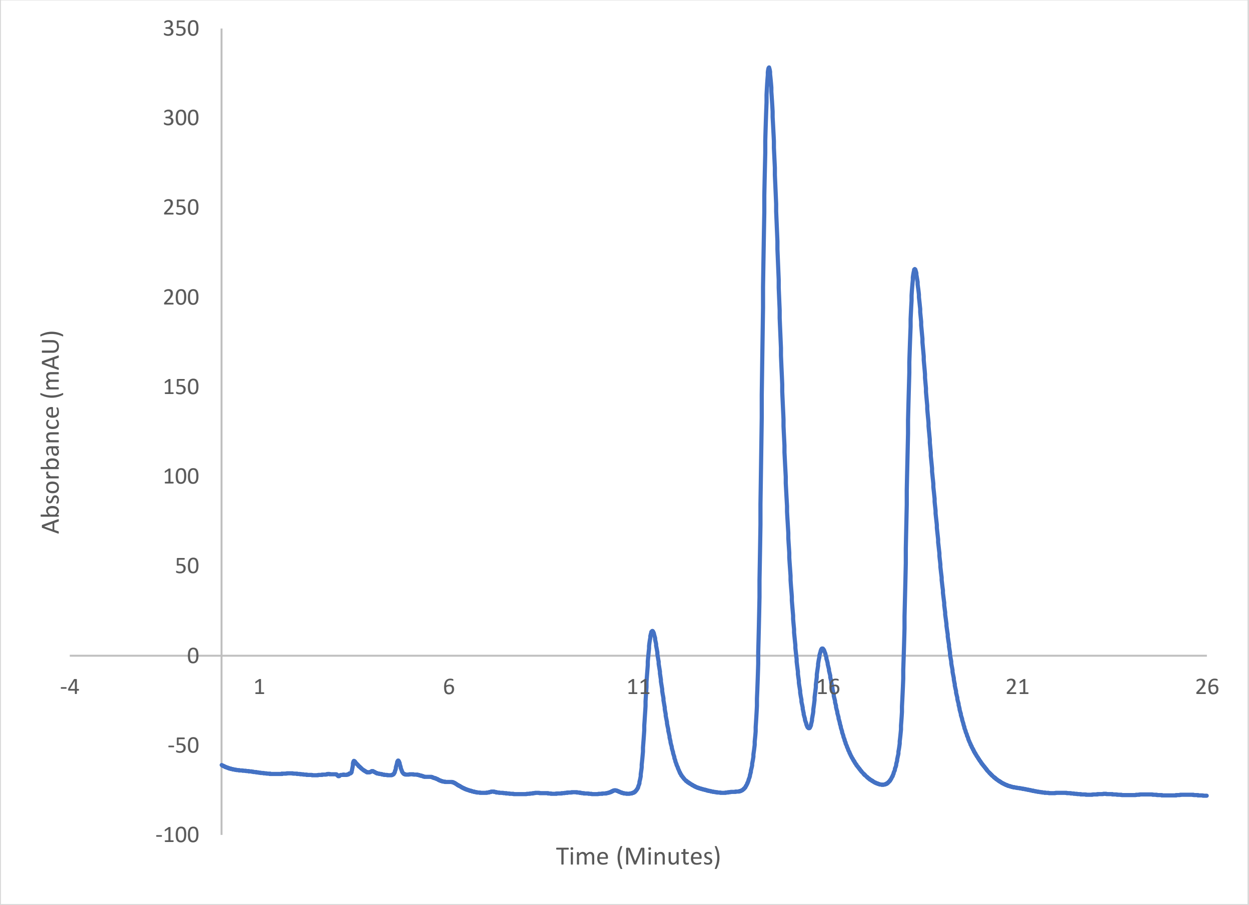

Conditions: (*S,S*)-Whelk-O 1, 98:2 hexanes/IPA, flow rate 1mL/min, λ=254 nm.

#	Time	Area	Height	Width	Area%	Symmetry
1	11.367	3341.4	90.7	0.5458	8.511	0.458
2	14.443	16006.1	403.5	0.5948	40.770	0.45
3	15.853	3555.2	77.7	0.6576	9.056	0.479
4	18.291	16357	288.6	0.8011	41.664	0.324

Figure S94. Chiral HPLC separation of the asymmetric reaction product, compound **11** (*ee*=90%, *dr*=35:1).

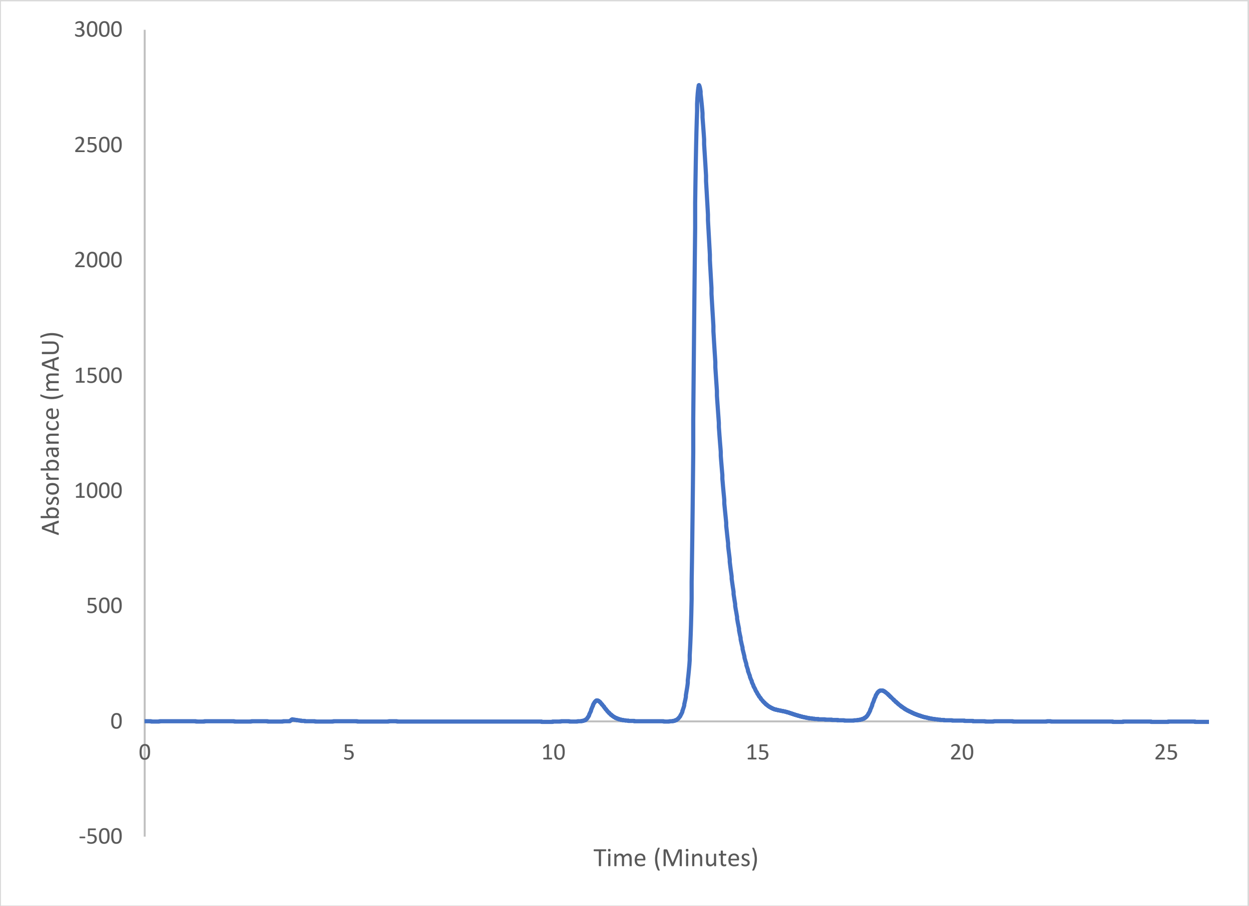

Conditions: (*S,S*)-Whelk-O 1, 98:2 hexanes/IPA, flow rate 1mL/min, λ=254 nm.

#	Time	Area	Height	Width	Area%	Symmetry
1	13.566	117747.7	2759.6	0.6058	94.785	0.247
2	18.028	6478.7	131.7	0.7101	5.215	0.401

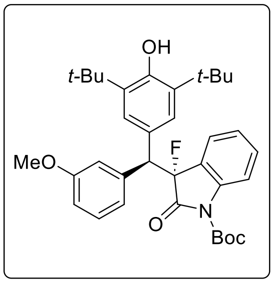

Figure S95. Chiral HPLC separation of a racemic mixture of compound **12** (*dr*=7:1).

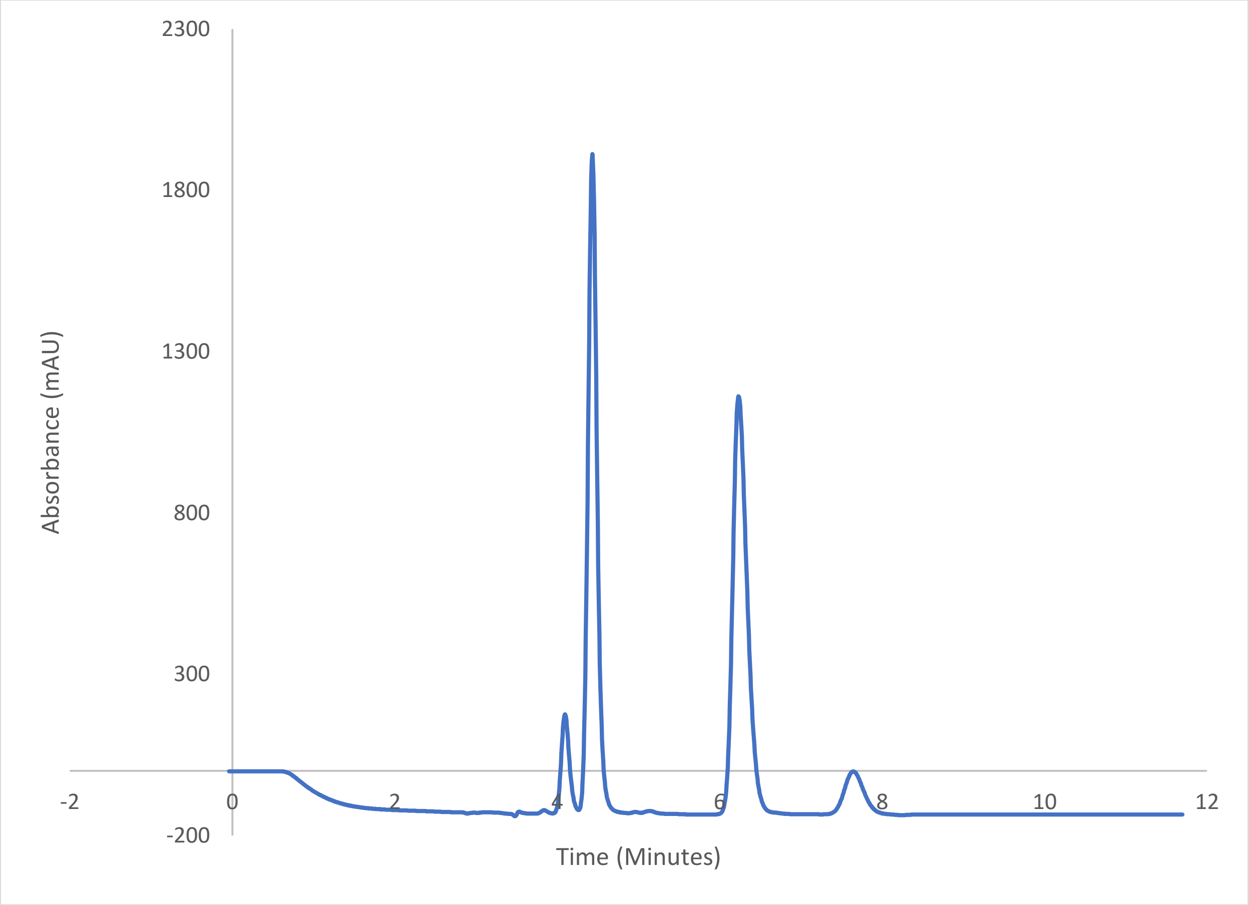

Conditions: Chiralpak AD-H, 98:2 hexanes/IPA, flow rate 1mL/min, λ=254 nm.

#	Time	Area	Height	Width	Area%	Symmetry
1	4.096	2262.2	312.8	0.1104	6.146	0.824
2	4.433	16113.8	2046.5	0.1219	43.781	0.883
3	6.234	16146.2	1297	0.1912	43.869	0.658
4	7.645	2283.2	134.1	0.2647	6.203	0.807

Figure S96. Chiral HPLC separation of the asymmetric reaction product, compound **12** (*ee*=89%, *dr*=24:1).

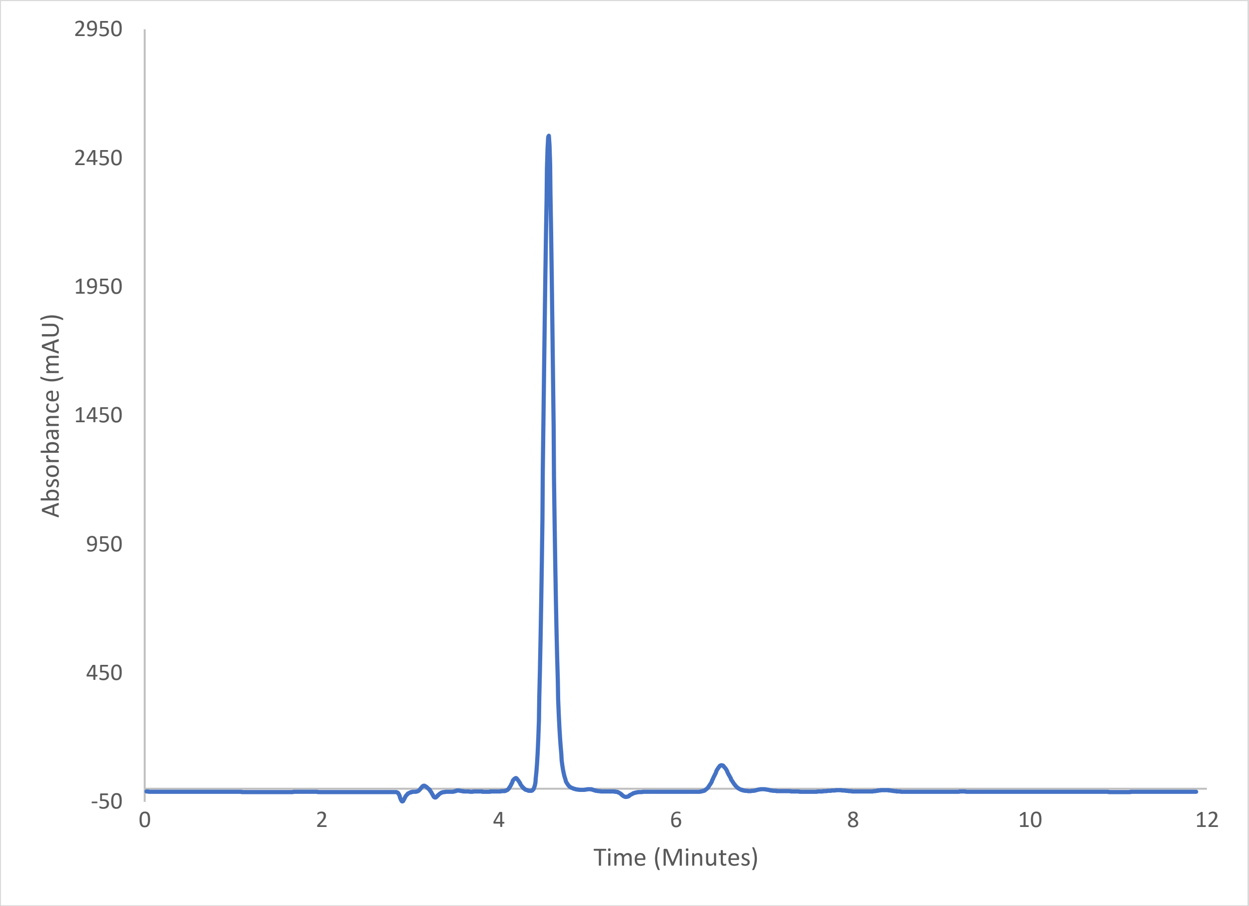

Conditions: Chiralpak AD-H, 98:2 hexanes/IPA, flow rate 1mL/min, λ=254 nm.

#	Time	Area	Height	Width	Area%	Symmetry
1	4.562	21105.2	2559.1	0.1375	94.731	0.945
2	6.517	1173.9	97.3	0.2011	5.269	0.821

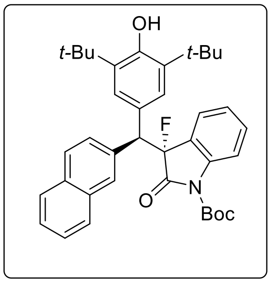

Figure S97. Chiral HPLC separation of a racemic mixture of compound **13** (*dr*=9:1).

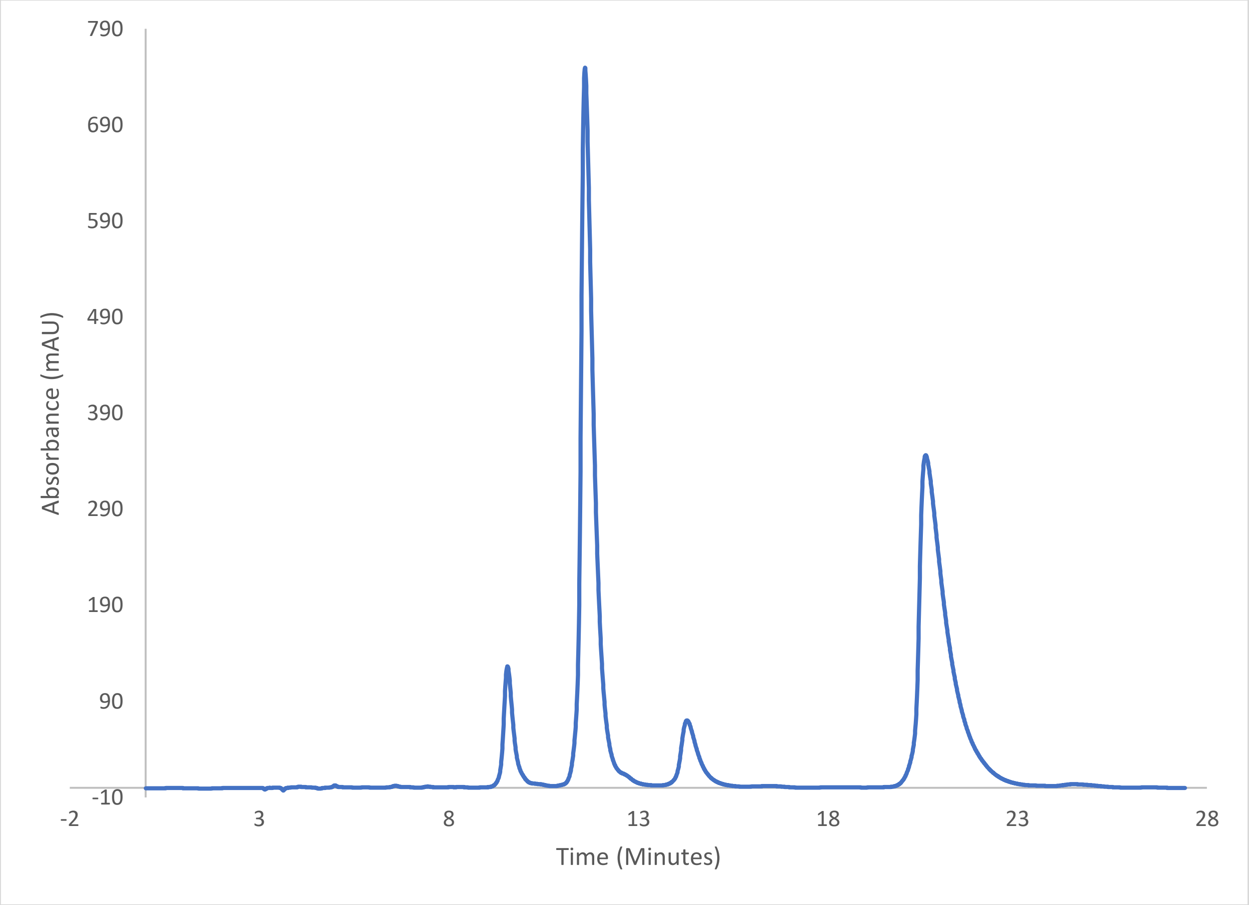

Conditions: (*S,S*)-Whelk-O 1, 98.5:1.5 hexanes/IPA, flow rate 1mL/min, λ=254 nm.

#	Time	Area	Height	Width	Area%	Symmetry
1	9.546	2401.2	125.5	0.2812	5.819	0.591
2	11.59	18530.9	747.9	0.3571	44.904	0.443
3	14.282	2203.3	68.2	0.4727	5.339	0.528
4	20.578	18132.4	345.4	0.7464	43.938	0.299

Figure S98. Chiral HPLC separation of the asymmetric reaction product, compound **13** (*ee*=93%, *dr*=53:1).

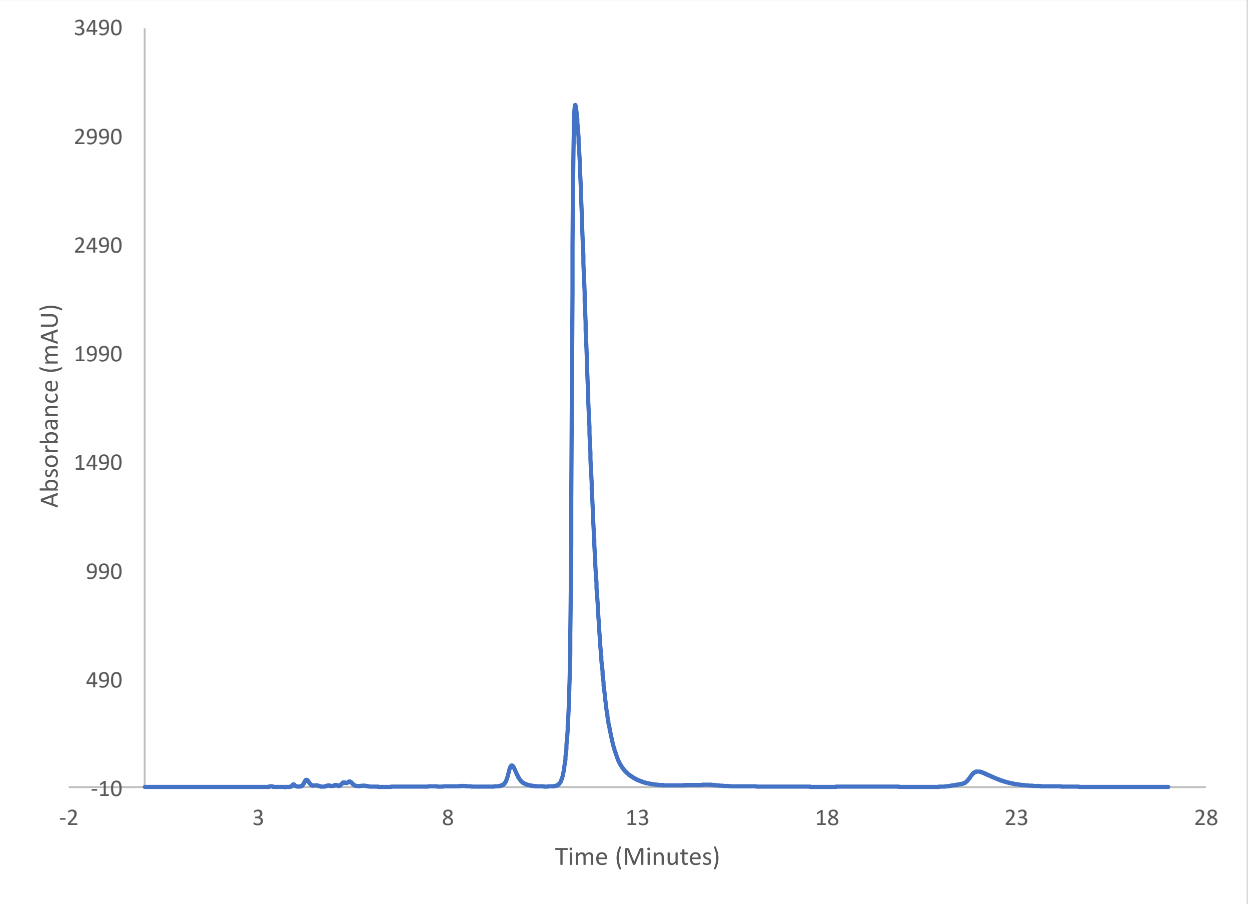

Conditions: (*S,S*)-Whelk-O 1, 98.5:1.5 hexanes/IPA, flow rate 1mL/min, λ=254 nm.

#	Time	Area	Height	Width	Area%	Symmetry
1	11.359	106765.5	3134.6	0.4914	96.310	0.23
2	21.978	4090.7	71.2	0.8021	3.690	0.459

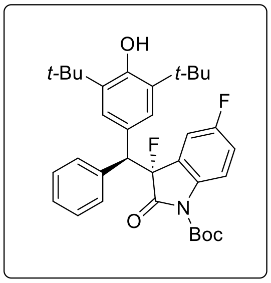

Figure S99. Chiral HPLC separation of a racemic mixture of compound **14** (*dr*=5:1).

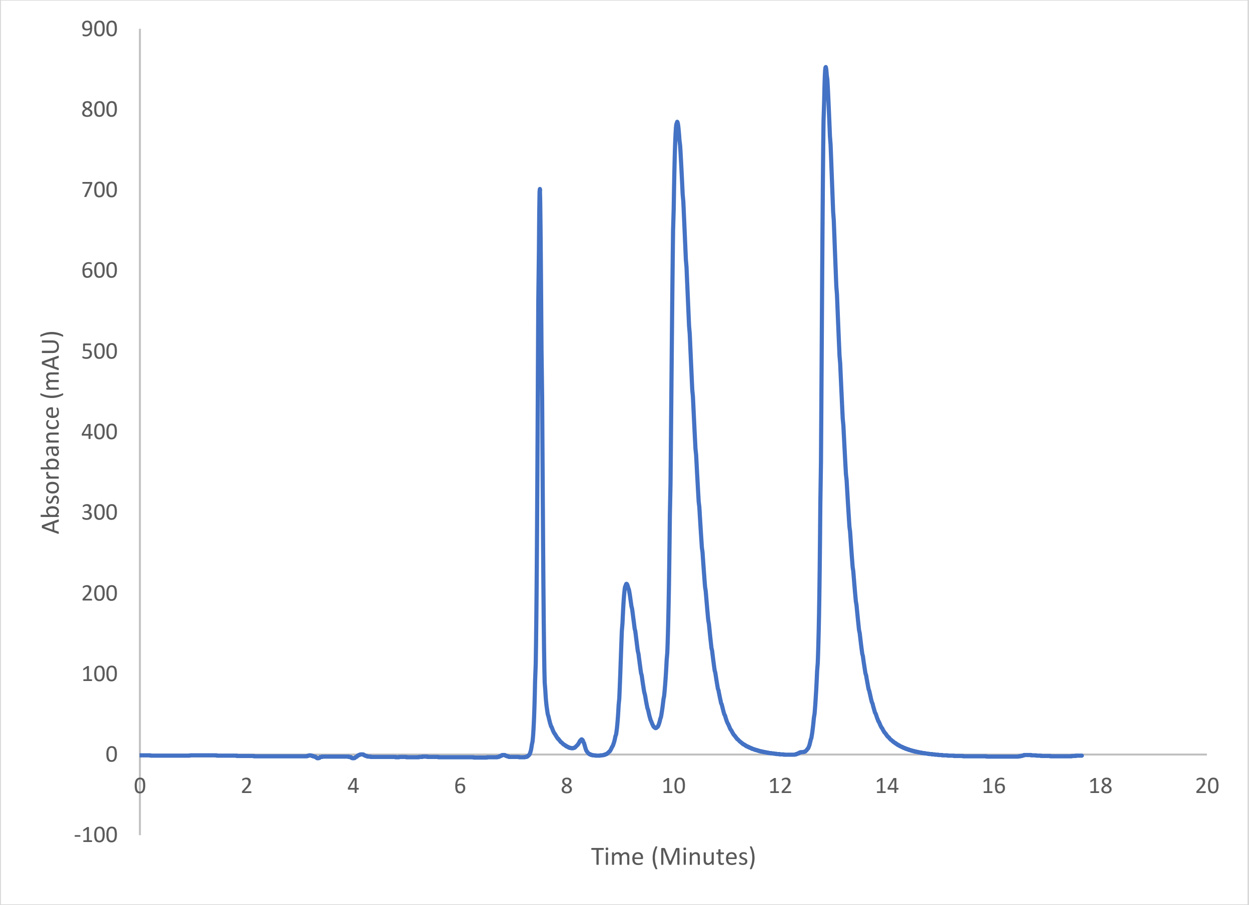

Conditions: (*S,S*)-Whelk-O 1, 99.5:0.5 hexanes/IPA, flow rate 1mL/min, λ=254 nm.

#	Time	Area	Height	Width	Area%	Symmetry
1	7.492	5267.1	705.1	0.1112	8.640	0.646
2	9.12	5273.5	212.8	0.3711	8.650	0.433
3	10.069	25253.9	785.5	0.4611	41.425	0.331
4	12.853	25168	853.5	0.406	41.284	0.241

Figure S100. Chiral HPLC separation of the asymmetric reaction product, compound **14** (*ee*=85%, *dr*=13:1).

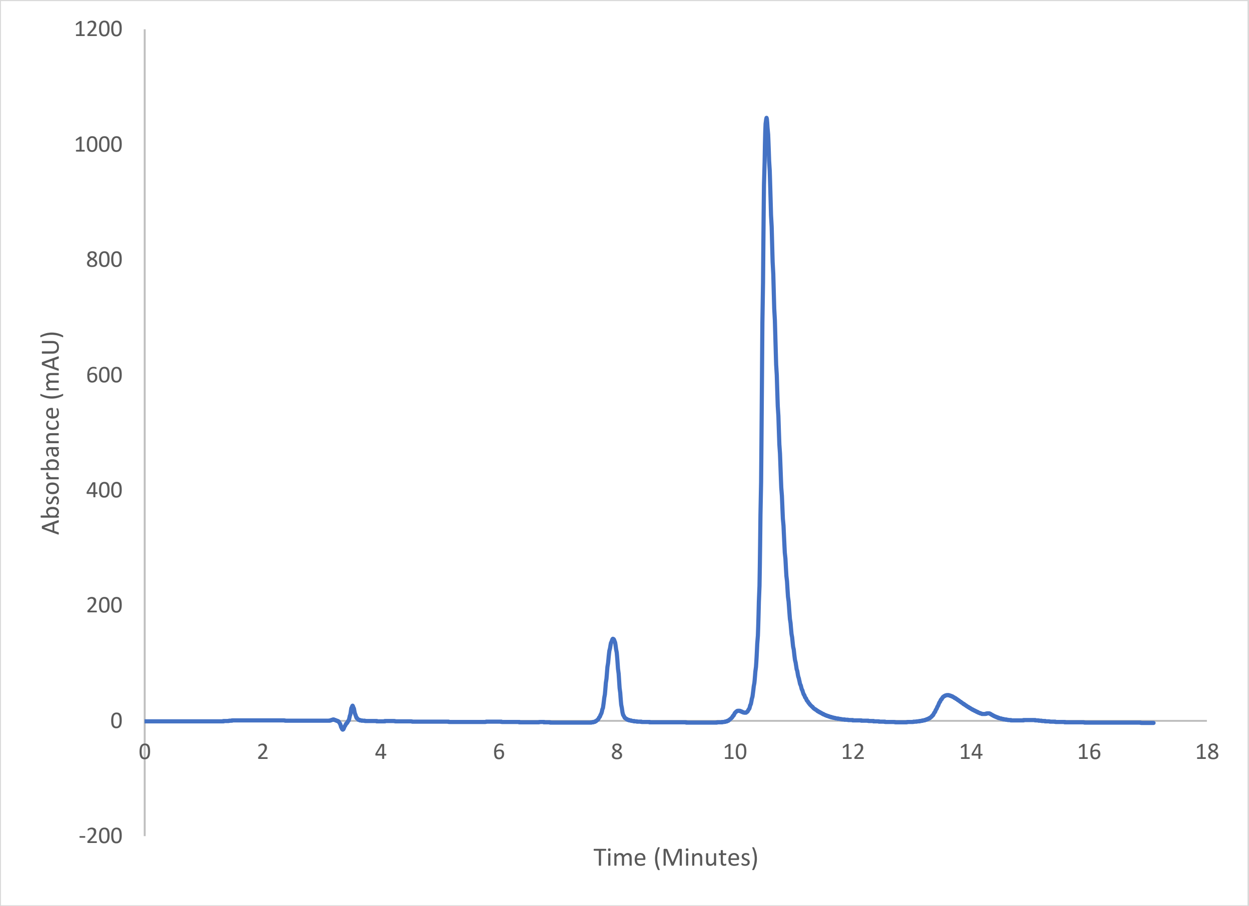

Conditions: (*S,S*)-Whelk-O 1, 99.5:0.5 hexanes/IPA, flow rate 1mL/min, λ=254 nm.

#	Time	Area	Height	Width	Area%	Symmetry
1	10.535	22043.7	1049.1	0.2971	92.654	0.369
2	13.604	1747.6	47.1	0.6186	7.346	0.504

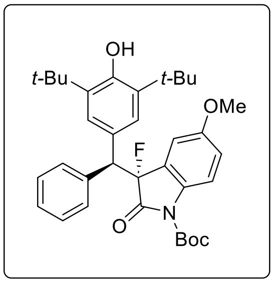

Figure S101. Chiral HPLC separation of a racemic mixture of compound **15** (*dr*=11:1).

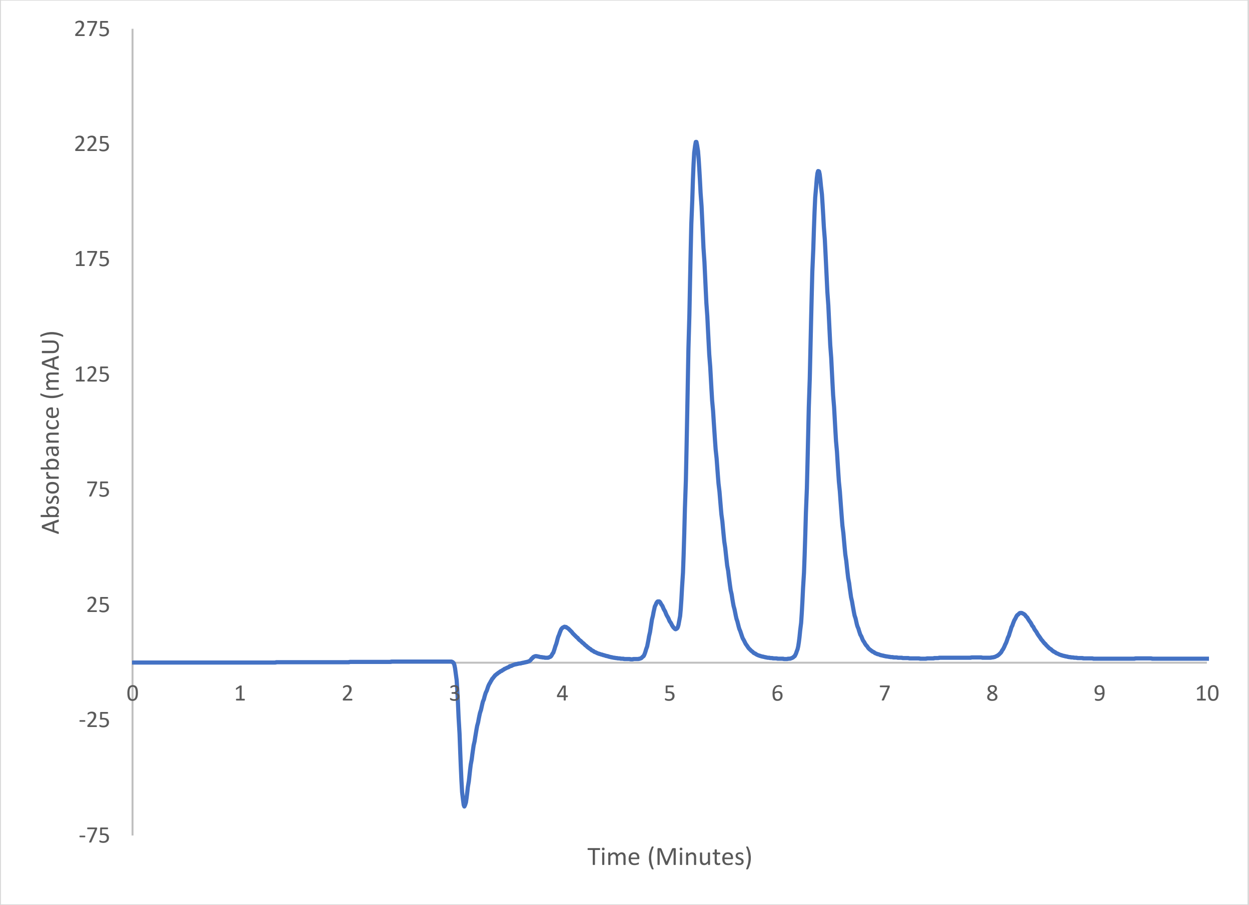

Conditions: Chiralpak IA, 98:2 hexanes/IPA, flow rate 1mL/min, λ=254 nm.

#	Time	Area	Height	Width	Area%	Symmetry
1	4.893	302.4	25.1	0.1805	3.981	0.63
2	5.244	3550.2	224.6	0.2264	46.742	0.459
3	6.383	3386.5	211.8	0.2366	44.586	0.528
4	8.269	356.3	19.5	0.2767	4.691	0.62

Figure S102. Chiral HPLC separation of the asymmetric reaction product, compound **15** (*ee*=60%, *dr*=34:1).

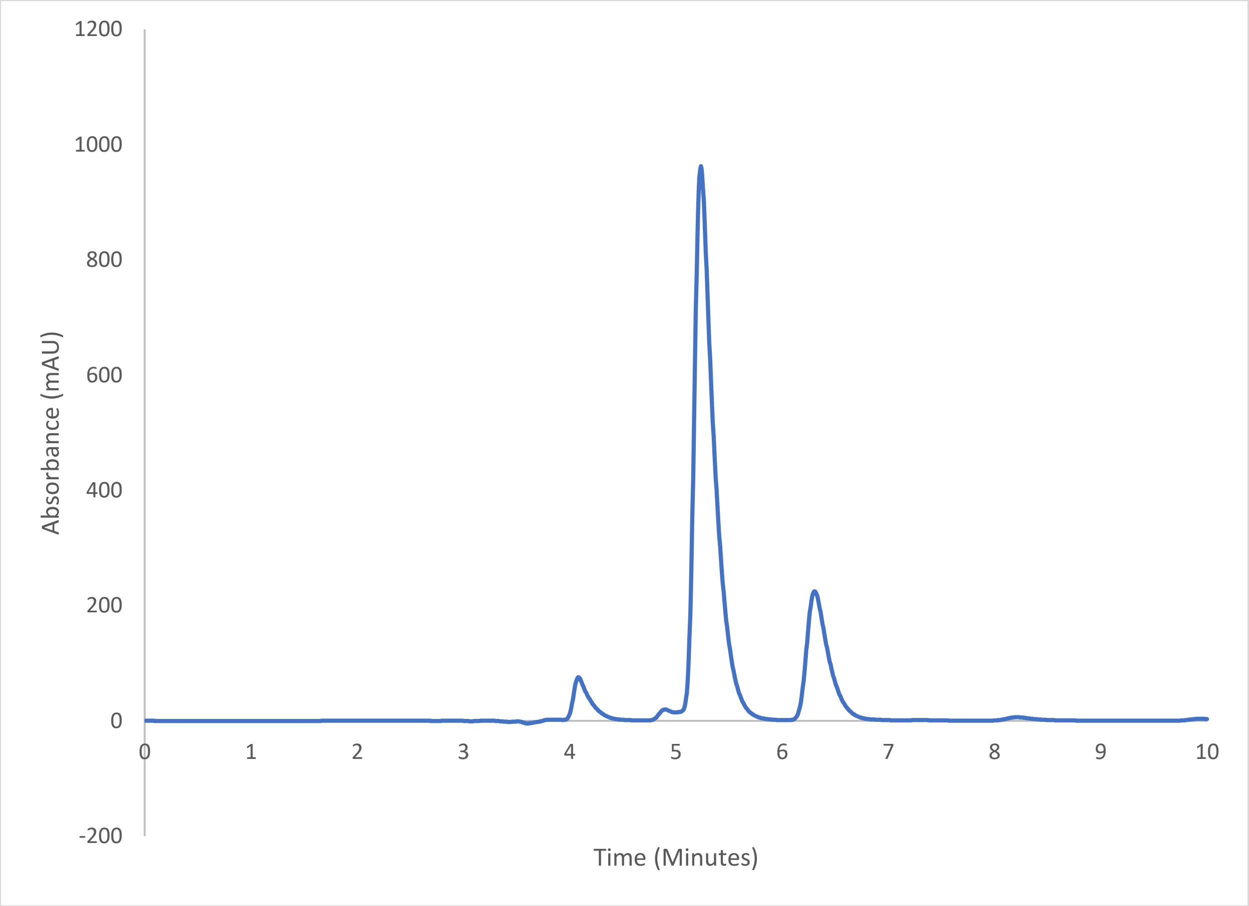

Conditions: Chiralpak IA, 98:2 hexanes/IPA, flow rate 1mL/min, λ=254 nm.

#	Time	Area	Height	Width	Area%	Symmetry
1	5.235	12733.3	961.4	0.1905	79.795	0.468
2	6.306	3224.3	223.9	0.212	20.205	0.529

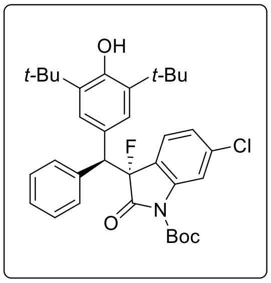

Figure S103. Chiral HPLC separation of a racemic mixture of compound **16** (*dr*=3:1).

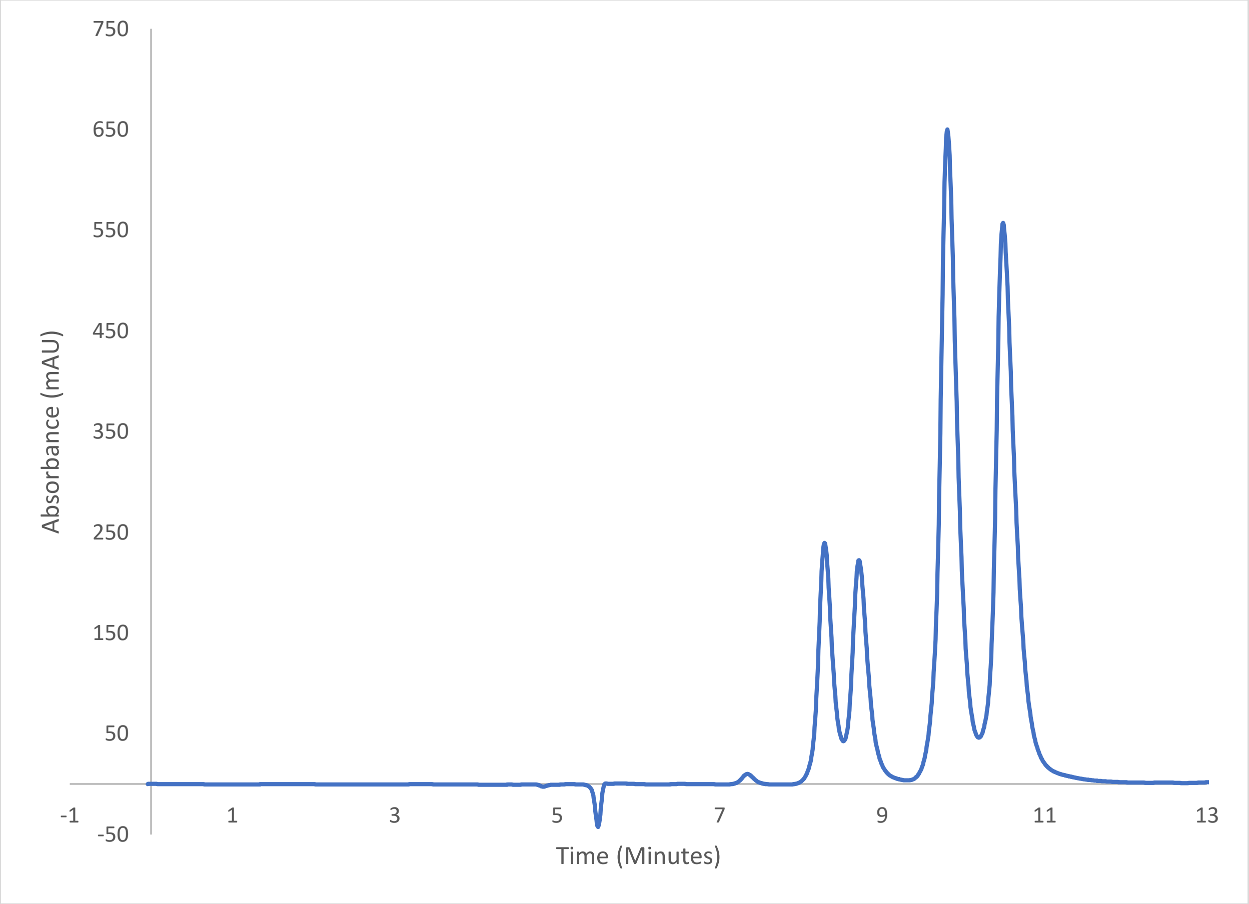

Conditions: (*S,S*)-Whelk-O 1, 99:1 hexanes/IPA, flow rate 0.65 mL/min, λ=254 nm.

#	Time	Area	Height	Width	Area%	Symmetry
1	8.292	3015.8	238.7	0.1874	11.416	0.788
2	8.715	3121.7	220.4	0.2092	11.817	0.729
3	9.804	10154.8	646.8	0.2292	38.442	0.701
4	10.489	10123.8	554.6	0.267	38.324	0.579

Figure S104. Chiral HPLC separation of the asymmetric reaction product, compound **16** (*ee*=71%, *dr*=24:1). Y-axis adjusted by +120 mAU.

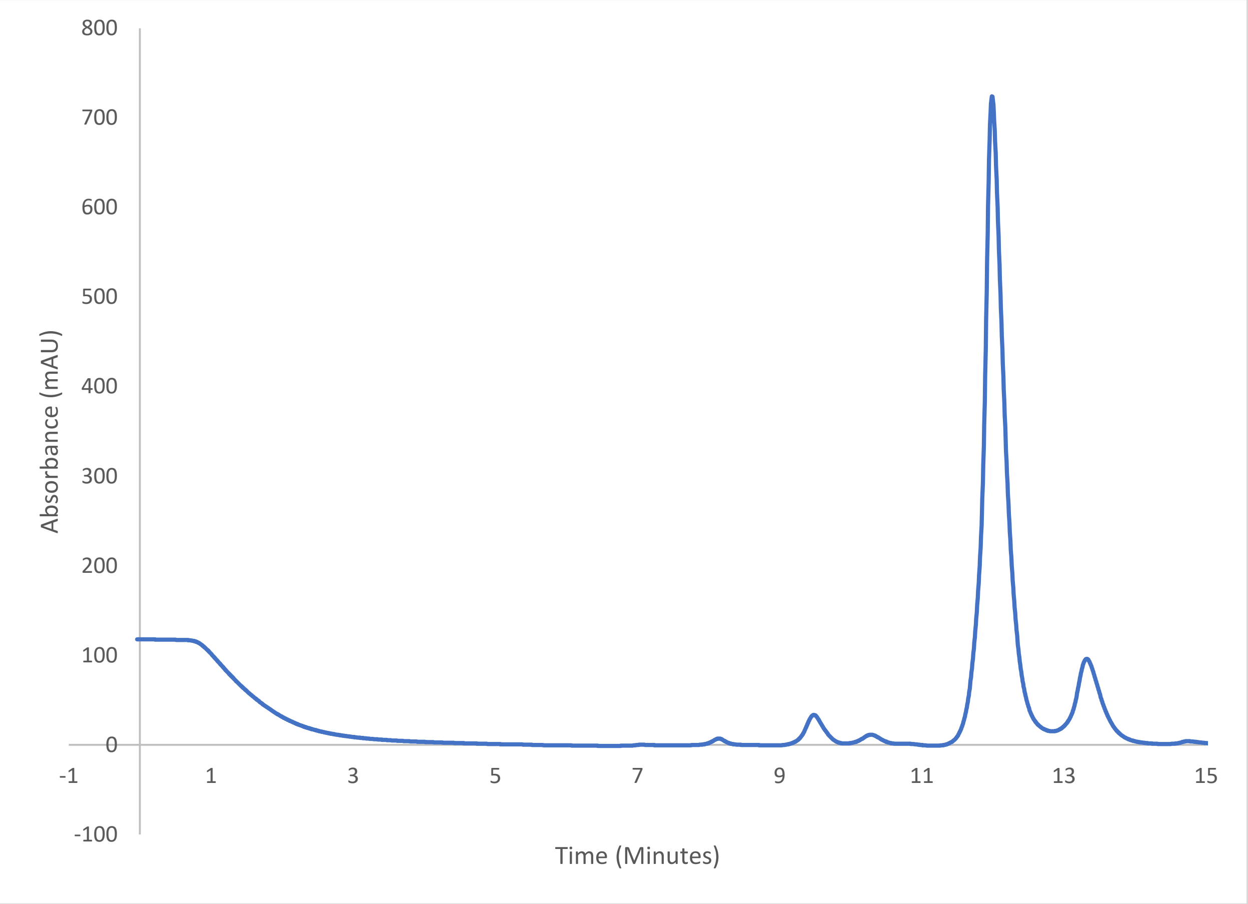

Conditions: (*S,S*)-Whelk-O 1, 99:1 hexanes/IPA, flow rate 0.65 mL/min, λ=254 nm.

#	Time	Area	Height	Width	Area%	Symmetry
1	11.99	15867.5	724.9	0.3074	85.511	0.644
2	13.321	2688.7	95.8	0.4016	14.489	0.802

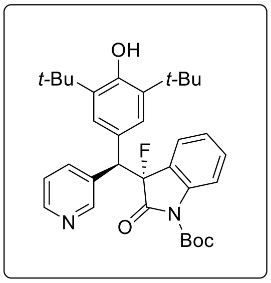

Figure S105. Chiral HPLC separation of a racemic mixture of compound **17** (*dr*=6:1).

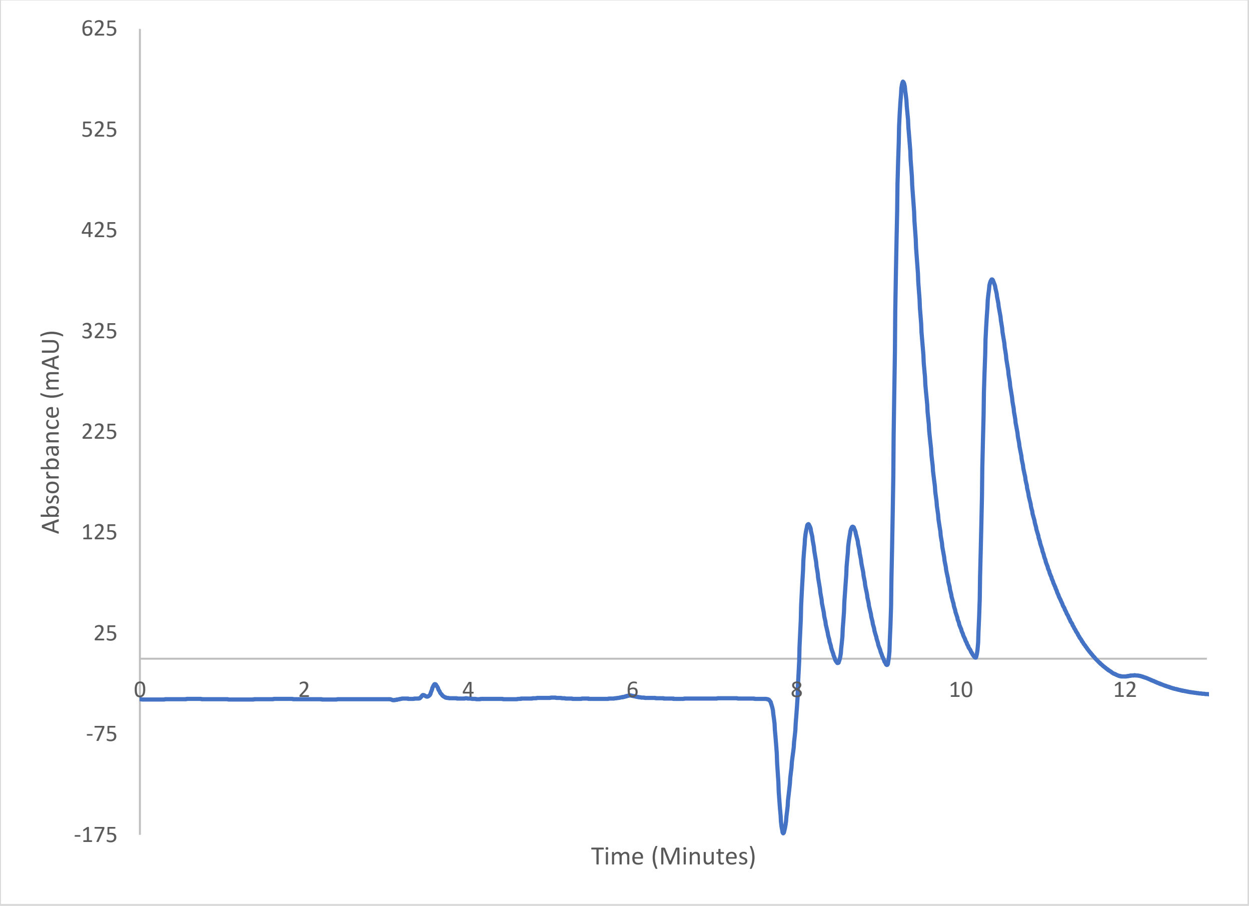

*y-axis adjusted by -40 mAU

Conditions: Chiralpak-IB, 98:2 hexanes/IPA, flow rate 1.0 mL/min, λ=254 nm.

#	Time	Area	Height	Width	Area%	Symmetry
1	8.143	2158.7	146.7	0.2452	6.438	0.434
2	8.683	2571.5	144.2	0.2972	7.669	0.492
3	9.297	14521.2	584.9	0.4138	43.305	0.331
4	10.379	14281.1	384.9	0.6184	42.589	0.225

Figure S106. Chiral HPLC separation of the asymmetric reaction product, compound **17** (*ee*=64%, *dr*=19:1).

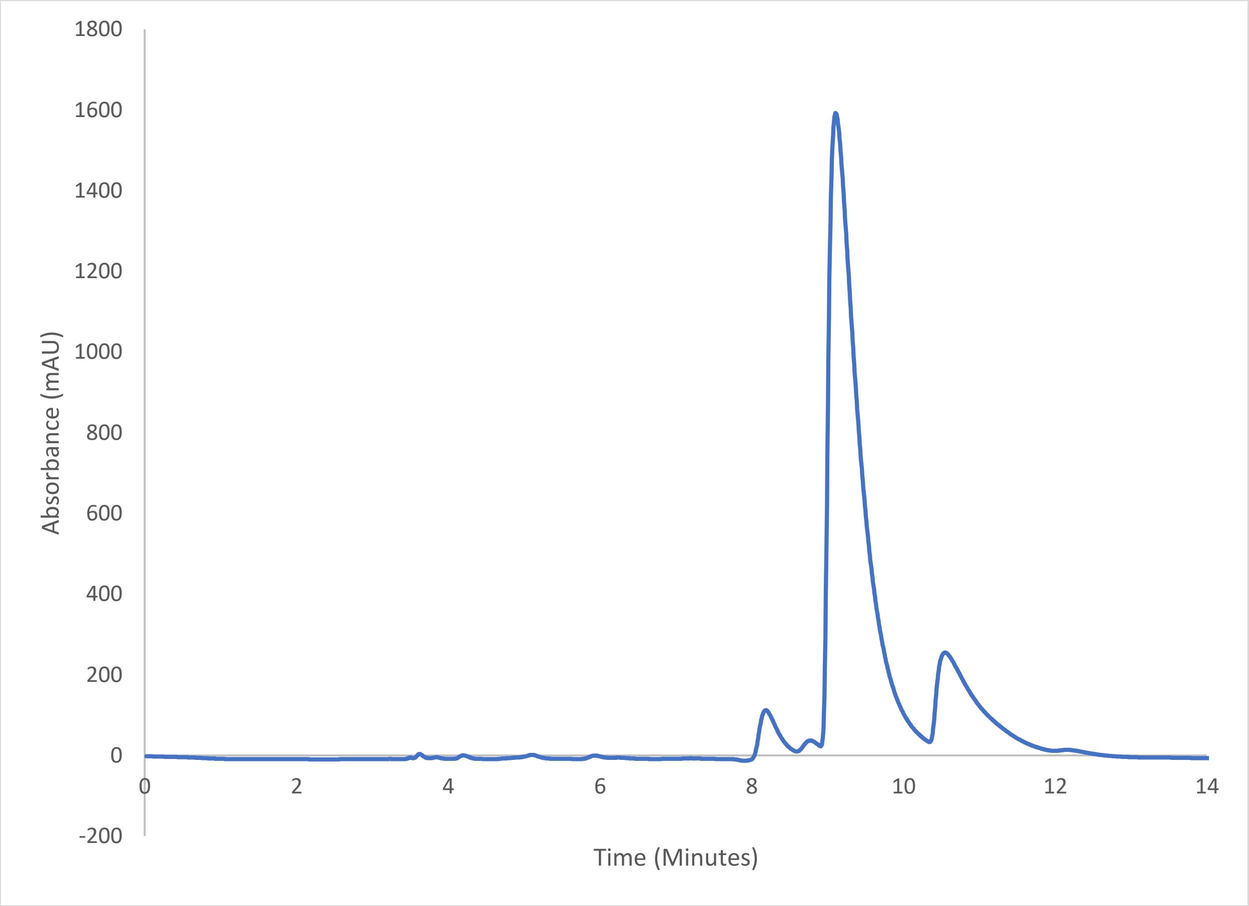

Conditions: Chiralpak-IB, 98:2 hexanes/IPA, flow rate 1.0 mL/min, λ=254 nm.

#	Time	Area	Height	Width	Area%	Symmetry
1	9.107	45558.8	1584.3	0.4155	82.010	0.262
2	10.548	9994.2	251.3	0.554	17.990	0.221

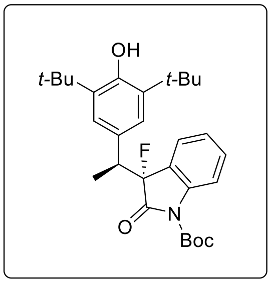

Figure S107. Chiral HPLC separation of a racemic mixture of compound **18** (*dr*=2:1).

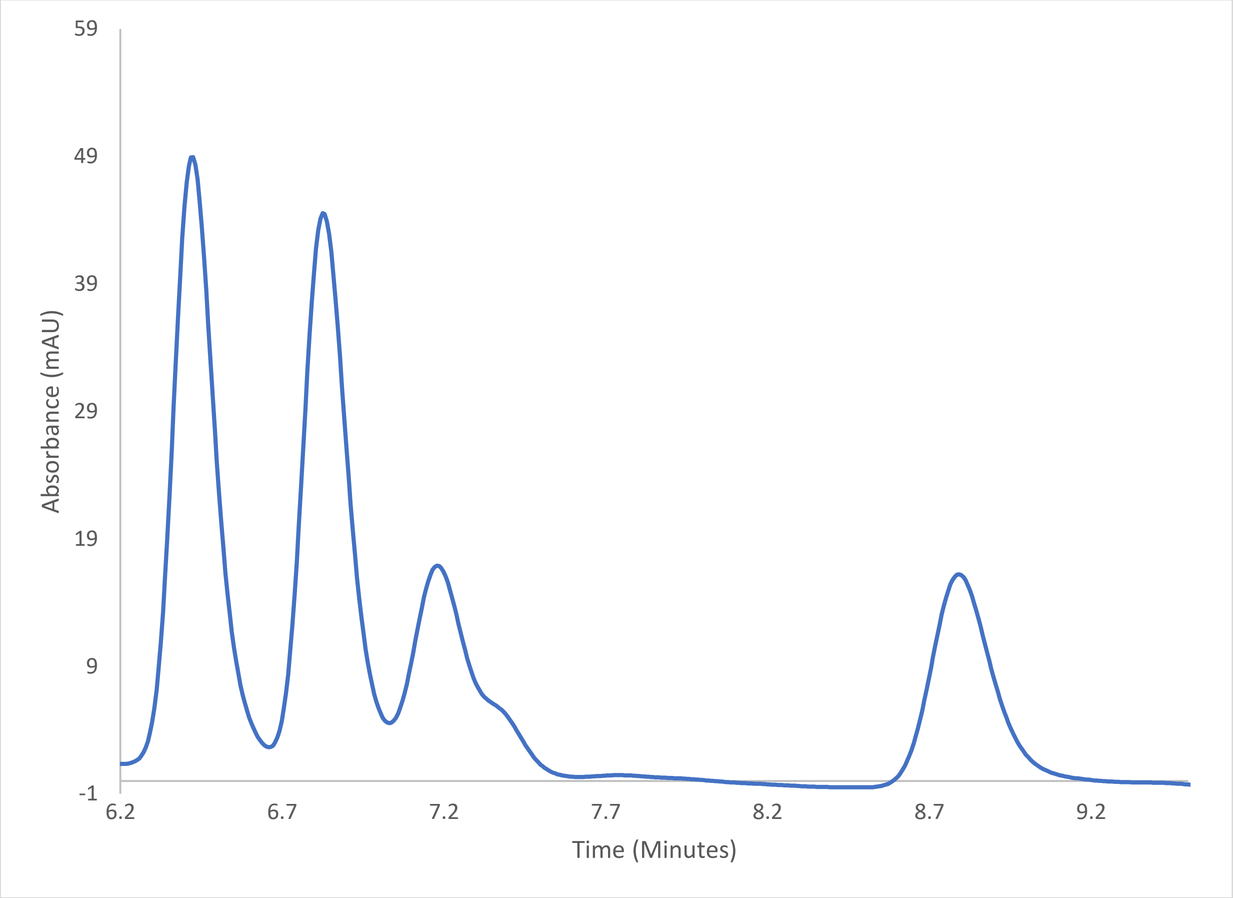

*y-axis adjusted by +10 units

Conditions: Chiralpak-IC, 98:2 hexanes/IPA, flow rate 0.75 mL/min, λ=254 nm.

#	Time	Area	Height	Width	Area%	Symmetry
1	6.422	426	46.9	0.1513	33.633	0.773
2	6.827	425.7	43.3	0.1638	33.612	0.793
3	7.18	203.7	15.9	0.214	16.082	0.616
4	8.791	211.2	16.4	0.2151	16.673	0.78

Figure S108. Chiral HPLC separation of the asymmetric reaction product, compound **18** (*ee*=31%, *dr*=3:1).

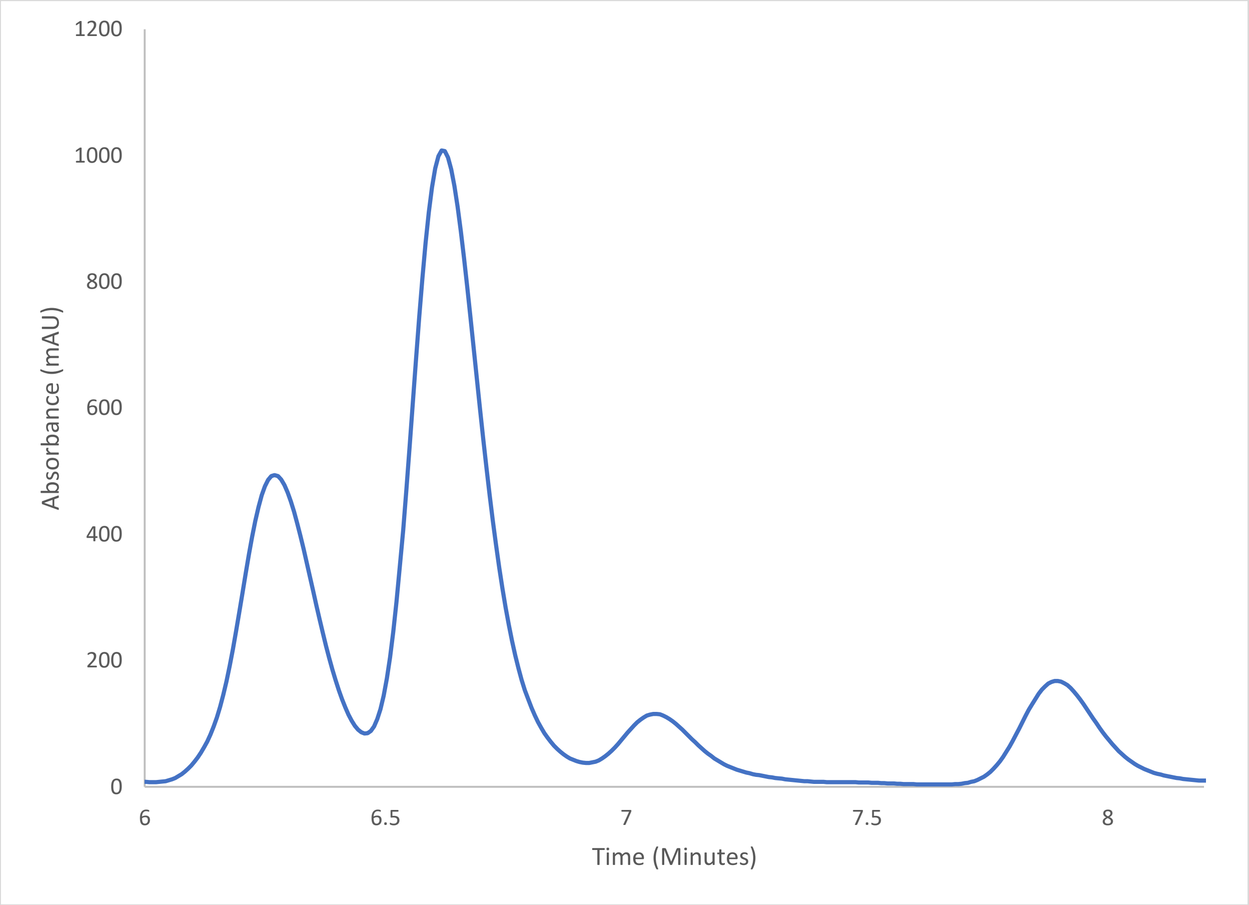

Conditions: Chiralpak-IC, 98:2 hexanes/IPA, flow rate 0.75 mL/min, λ=254 nm.

#	Time	Area	Height	Width	Area%	Symmetry
1	6.27	5358.1	478.3	0.1747	34.293	0.84
2	6.619	10266.5	981.4	0.162	65.707	0.759

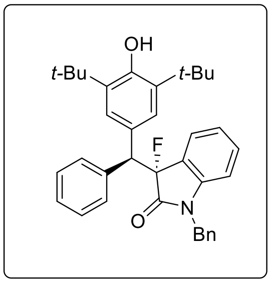

Figure S109. Chiral HPLC separation of a racemic mixture of compound **19** (*dr*=2:1).

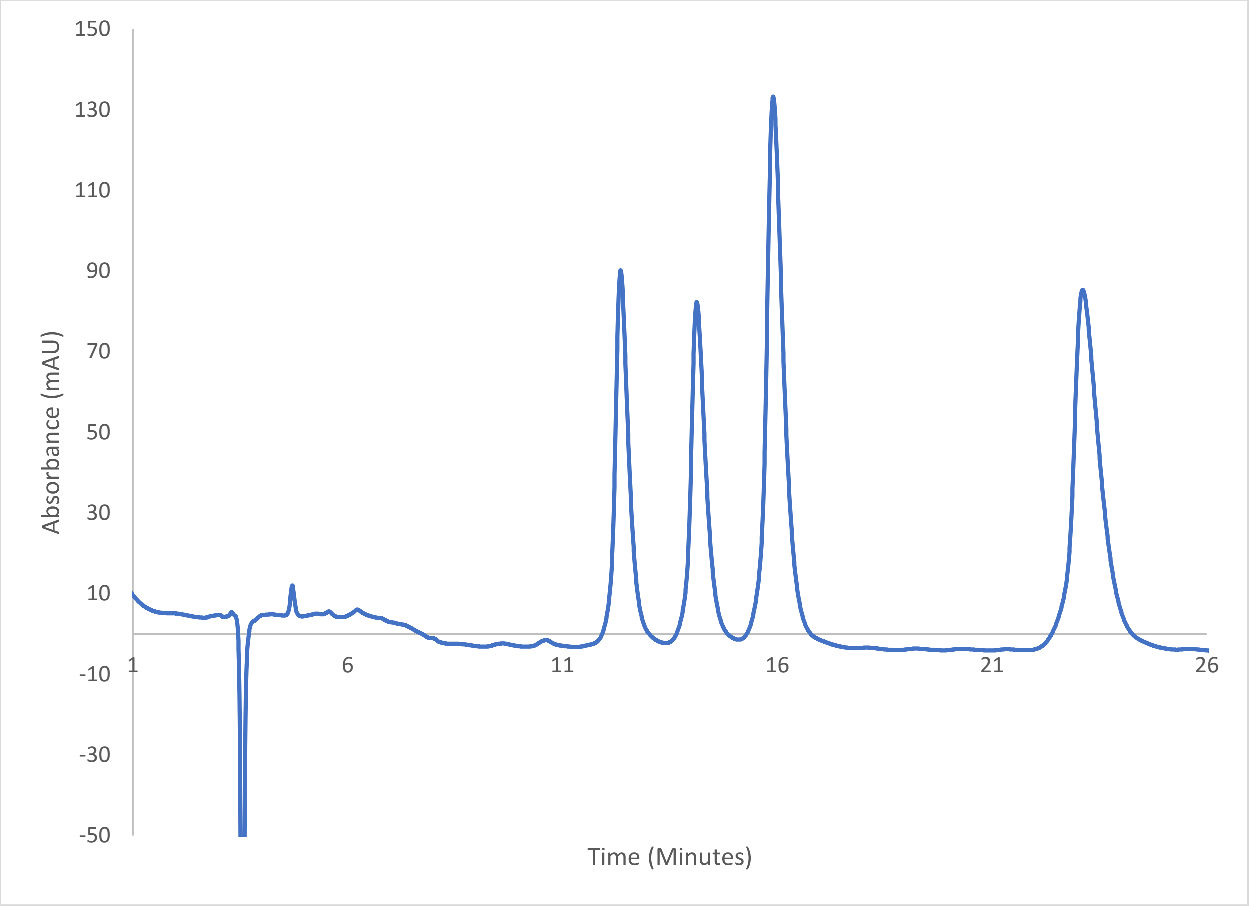

Conditions: (*S,S*)-Whelk-O 1, 98:2 hexanes/IPA, flow rate 1.0 mL/min, λ=254 nm.

#	Time	Area	Height	Width	Area%	Symmetry
1	12.354	2169.9	92.9	0.3424	17.634	0.669
2	14.127	2109	84.2	0.3661	17.139	0.651
3	15.906	3953.1	135.3	0.4288	32.125	0.625
4	23.112	4073.4	89.4	0.6777	33.103	0.554

Figure S110. Chiral HPLC separation of the asymmetric reaction product, compound **19** (*ee*=33% (major), 24% (minor)), *dr*=1.3:1).

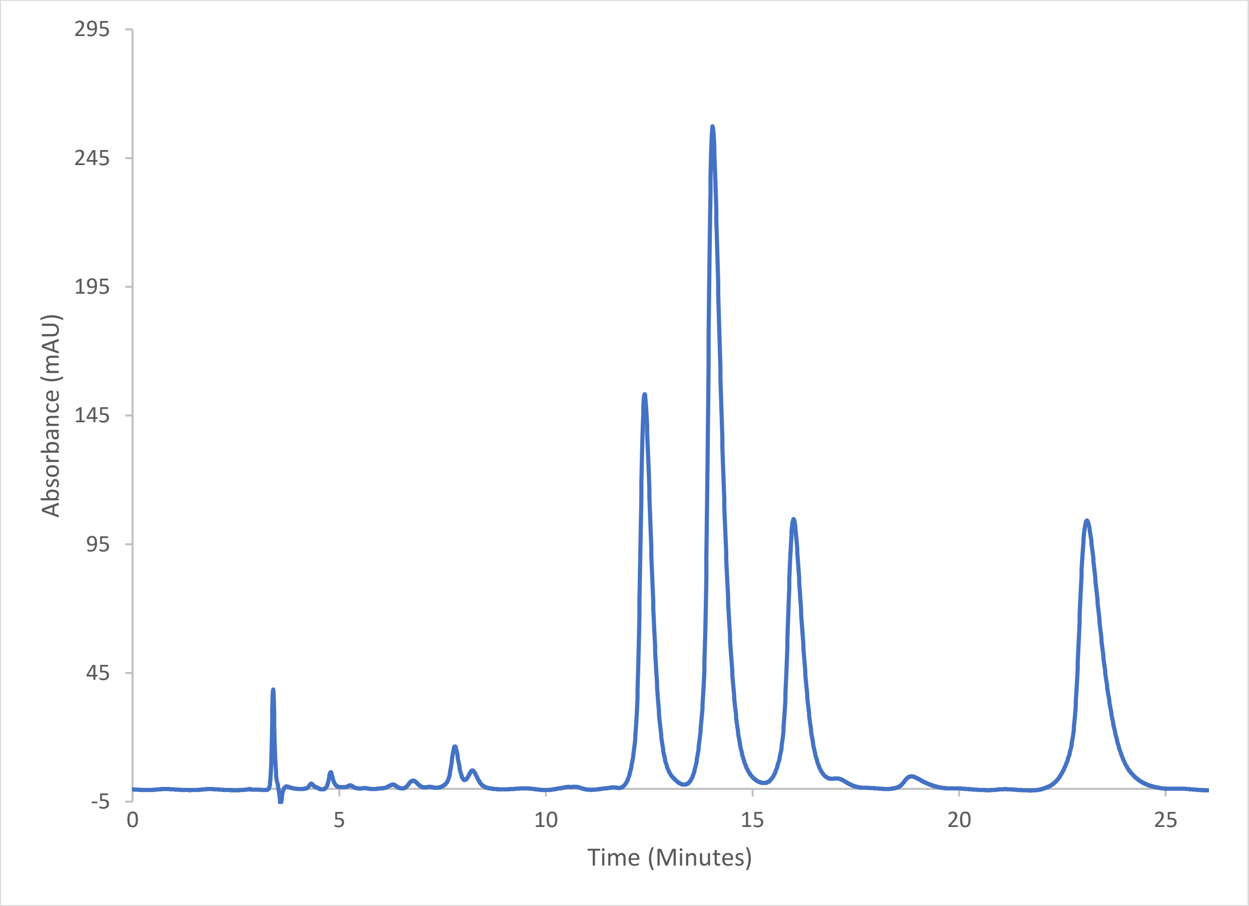

Conditions: (*S,S*)-Whelk-O 1, 98:2 hexanes/IPA, flow rate 1.0 mL/min, λ=254 nm.

#	Time	Area	Height	Width	Area%	Symmetry
1	12.391	3360.5	152.5	0.325	33.486	0.602
2	14.036	6674.9	255.5	0.3768	66.514	0.484

#	Time	Area	Height	Width	Area%	Symmetry
1	15.994	2777.8	101.9	0.4042	37.787	0.639
2	23.094	4573.5	104.6	0.632	62.213	0.545

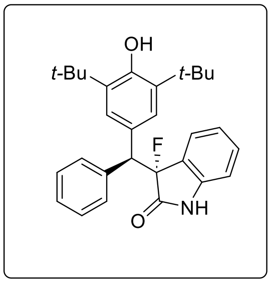

Figure S111. Chiral HPLC separation of a racemic mixture of compound **21** (*dr*= 3:1).

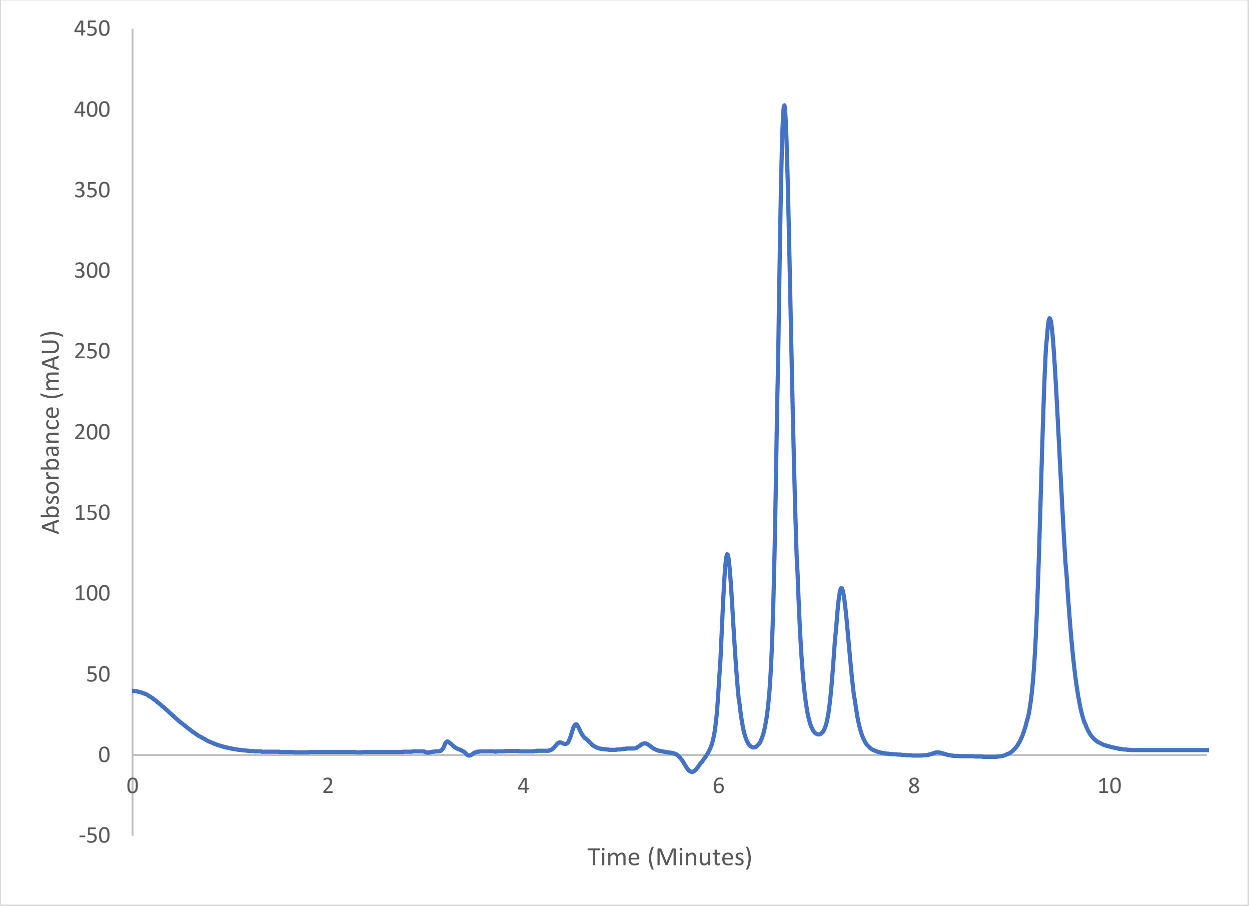

*Baseline adjusted by +40 mAU

Conditions: (*S,S*)-Whelk-O 1, 95:5 hexanes/IPA, flow rate 1.0 mL/min, λ=254 nm.

#	Time	Area	Height	Width	Area%	Symmetry
1	6.088	1567.4	133.3	0.1732	12.242	0.846
2	6.671	4961.9	409.2	0.1795	38.755	0.778
3	7.257	1548.7	107.3	0.2104	12.096	0.813
4	9.388	4725.2	270.2	0.26	36.906	0.668

Figure S112. Chiral HPLC separation of the asymmetric reaction product, compound **21** (*ee*= 94%, *dr*=48:1).

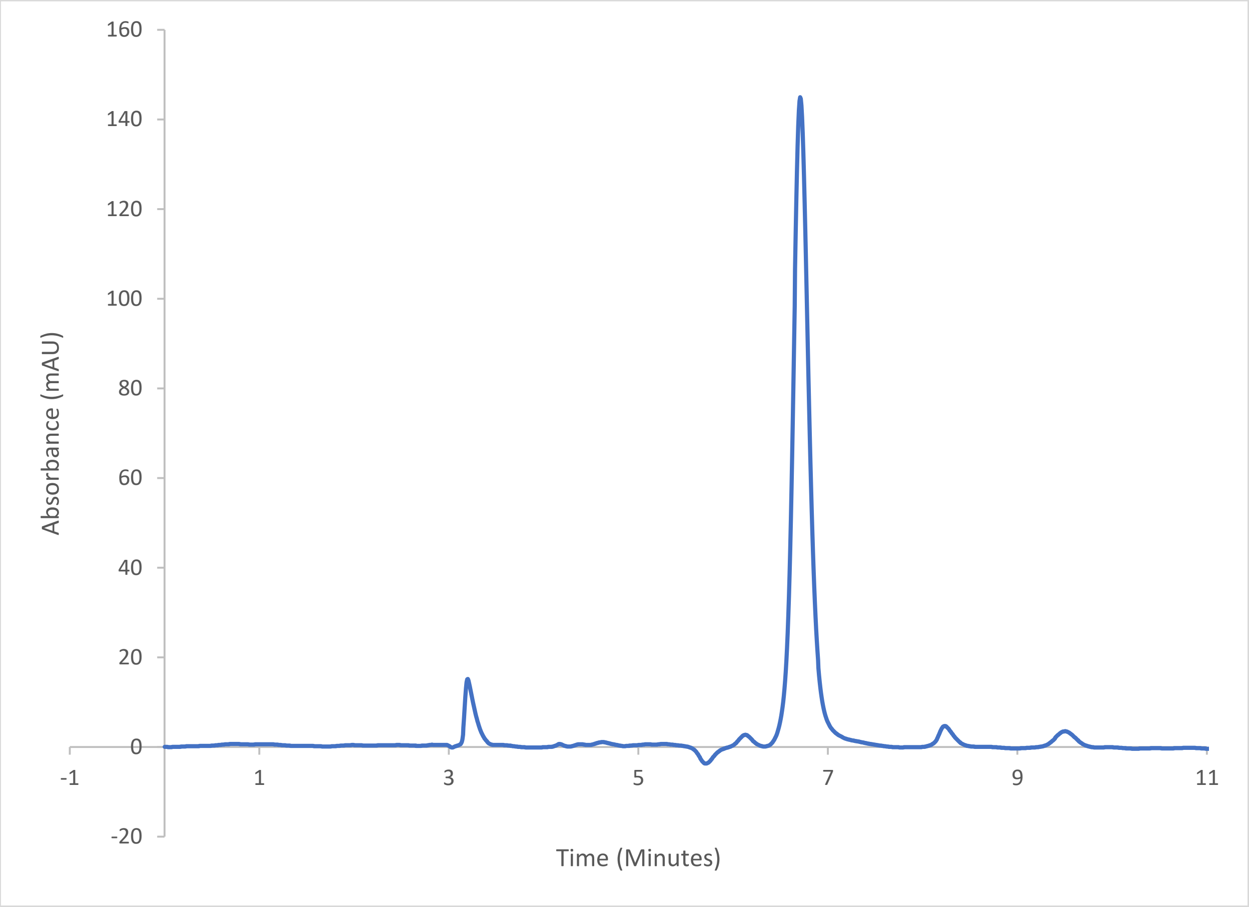

Conditions: (*S,S*)-Whelk-O 1, 95:5 hexanes/IPA, flow rate 1.0 mL/min, λ=254 nm.

#	Time	Area	Height	Width	Area%	Symmetry
1	6.709	1925.7	147	0.1929	96.972	0.773
2	9.501	60.1	3.7	0.25	3.028	1.039

9. References

[1]Ding, R.; Wolf, C. Organocatalytic Asymmetric Synthesis of α-Oxetanyl and α-Azetidinyl Tertiary Alkyl Fluorides and Chlorides. *Org. Lett.* **2018**, *20*, 892–895.

[2] Moskowitz, M.; Balaraman, K.; Wolf, C.; Organocatalytic Stereoselective Synthesis of Fluorinated 3,3′-Linked Bisoxindoles. *J. Org. Chem.* **2018**, *83*, 1661–1666.

[3]Kaluvu, B.; Wolf, C. Catalytic Enantioselective and Diastereoselective Allylic Alkylation with Fluoroenolates: Efficient Access to C3-Fluorinated and All-Carbon Quaternary Oxindoles *Angew. Chem. Int. Ed.* **2017**, *56,* 1390-1395.

[4]Wu, Q.; Guo, J.; Huang, G.; Chan, A. S. C.; Weng, J.; Lu, G. Visible Light-promoted Radical Cross-coupling of para-Quinone Methides with N-Substituted Anilines: An Efficient Approach to 2,2-Diarylethylamines. *Org. Biomol. Chem*. **2020**, *18*, 860-864.

[5] Zhu, J.; Xu, M.; Gong, B.; Lin, A.; Gao, S. (Z)-Selective Synthesis of Bromofluoroalkenes via the $TMSCF_2Br$-Mediated Tandem Reaction with para-Quinone Methides. *Org. Lett.* **2023**, *25*, 18, 3271–3275.

[6]Yan, Y.; Hao, J.; Xie, F.; Han, F.; Jing, L.; Han, P. Magnesium-Mediated Umpolung Carboxylation of p-Quinone Methides with CO_2. *J. Org. Chem.* **2023**, *88*, 14640–14648.

[7]Gaikwad, R.; Savekar, A. T.; Waghmode, S. B. Metal-Free Approach for Oxa-spirocyclohexadienones through [3 + 2]/[4 + 2] ipso-Cyclization of para-Quinone Methides with Halo Alcohols. *J. Org. Chem.* **2023**, *88*, 9987–10001.

[8]Xie, K.; Zhang, Z.; Li, X. Bismuth Triflate-Catalyzed Vinylogous Nucleophilic 1,6-Conjugate Addition of para-Quinone Methides with 3-Propenyl-2-silyloxyindoles. *Org. Lett.* **2017**, *19*, 6708–6711.

[9]Deng, Y.; Zhang. X.; Yu, K.; Yan, X.; Du, J.; Huang, H.; Fan, C. Bifunctional tertiary Amine-squaramide Catalyzed Asymmetric Catalytic 1,6-Conjugate Addition/aromatization of para-Quinone Methides with Oxindoles. *Chem. Commun.* **2016**, 52, 4183-4186.

[10] Zhao, K.; Zhi, Y.; Wang, A.; Enders, D. Asymmetric Organocatalytic Synthesis of 3-Diarylmethine-Substituted Oxindoles Bearing a Quaternary Stereocenter via 1,6-Conjugate Addition to *para*-Quinone Methides. *ACS Catal.* **2016**, *6*, 657–660.

[11]Ali, A.; Jajoria, R.; Harit, H. K.; Singh, R. P. Diastereoselective 1,6-Addition of α-Phosphonyloxy Enolates to para-Quinone Methides. *J. Org. Chem.* **2022**, *87*, 5213-5228.

[12]Luo, C.; Lu, W.; Wang, G.; Zhang, Z.; Li, H.; Han, P.; Yang, D.; Jing, L,; Wang, C. Photocatalytic Synthesis of Diarylmethyl Silanes via 1,6-Conjugate Addition of Silyl Radicals to *p*-Quinone Methides. *J. Org. Chem.* **2022**, *87*, 3567-3576.
